# Supplementary figures and images for: Overcoming the cytoplasmic retention of GDOWN1 modulates global transcription and facilitates stress adaptation (part 1 of 2)
Source: eLife. 2022 Dec 7;11:e79116. doi: 10.7554/eLife.79116 (PMC9728996; doi:10.7554/eLife.79116)

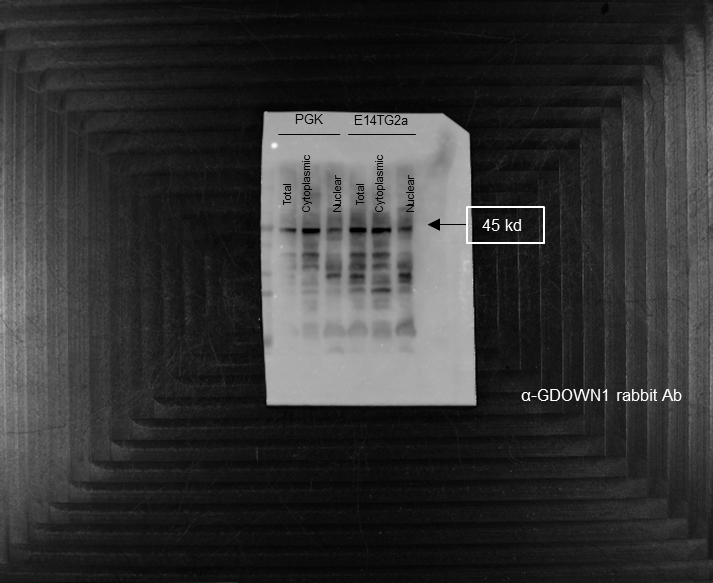

Supplement: Figure 1—source data 1. [file elife-79116-fig1-data1.zip › Figure 1-source data 1/+Label/Fig 1B-GDOWN1 rabbit antibody (For E14TG2a).tif]

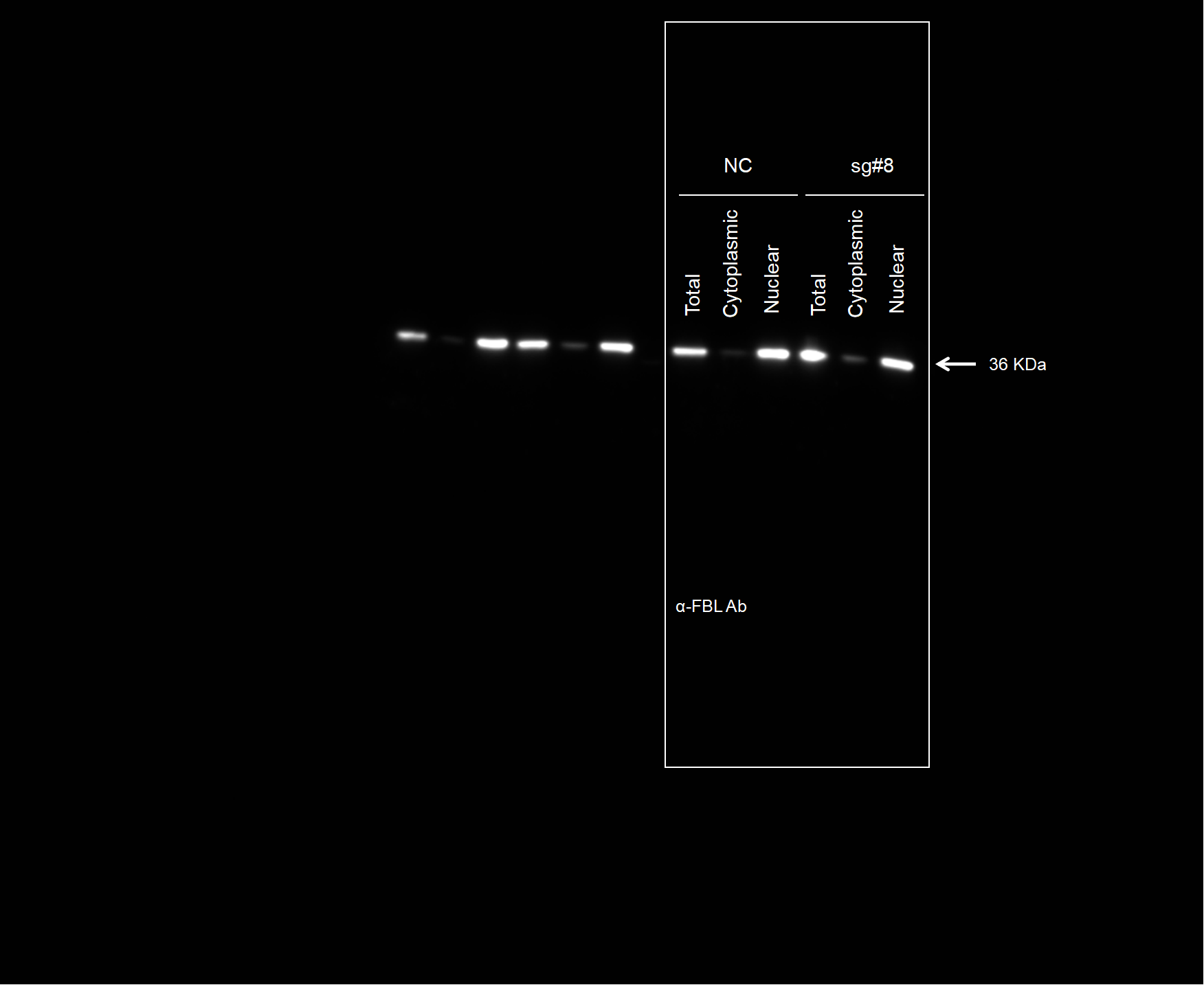

Supplement: Figure 1—source data 1. [file elife-79116-fig1-data1.zip › Figure 1-source data 1/+Label/Fig 1B-GDOWN1 FBL antibody (for KO sg#8), Right.Tif.tif]

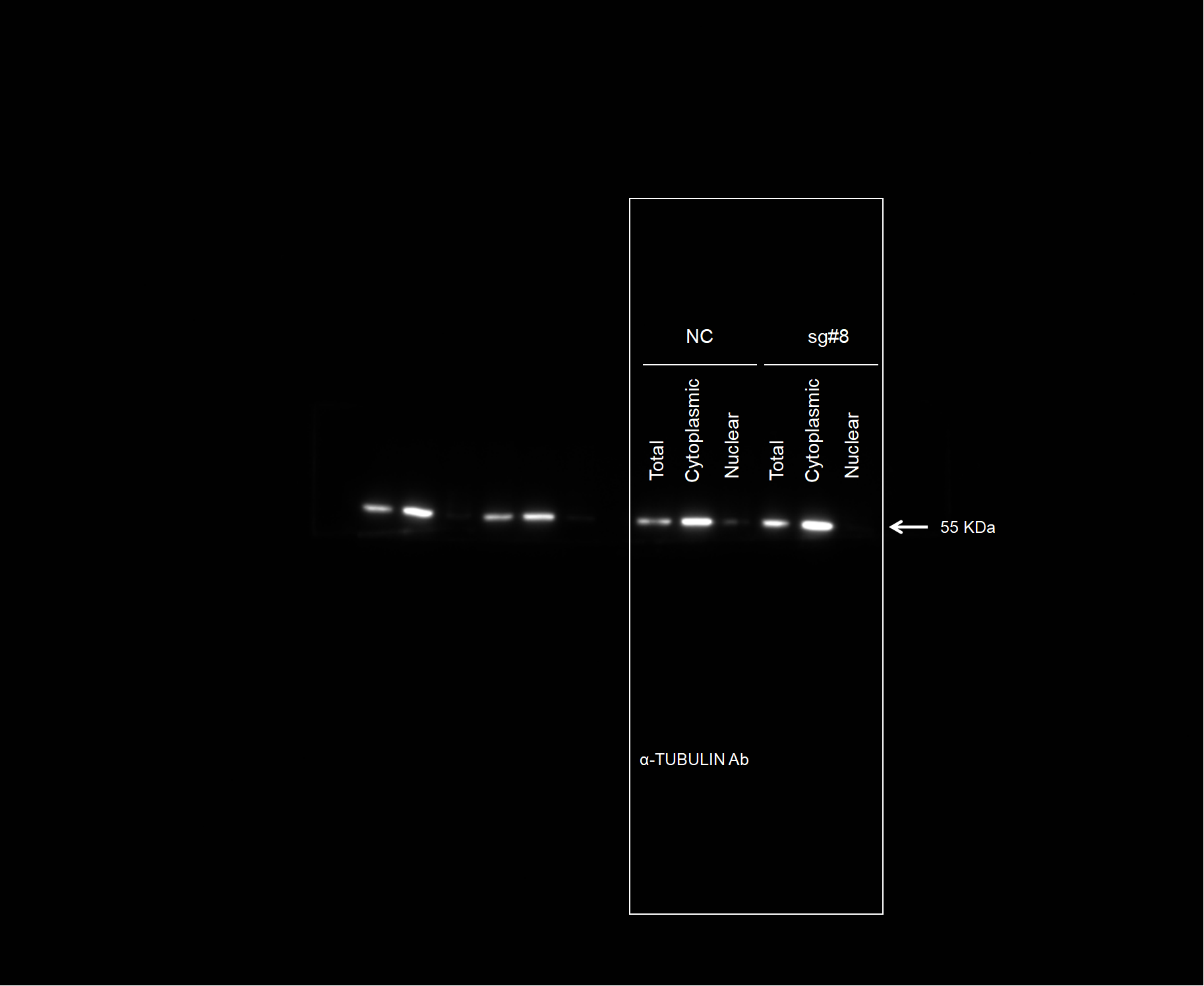

Supplement: Figure 1—source data 1. [file elife-79116-fig1-data1.zip › Figure 1-source data 1/+Label/Fig 1B-GDOWN1 TUBULIN antibody (for KO sg#8), Right.Tif.tif]

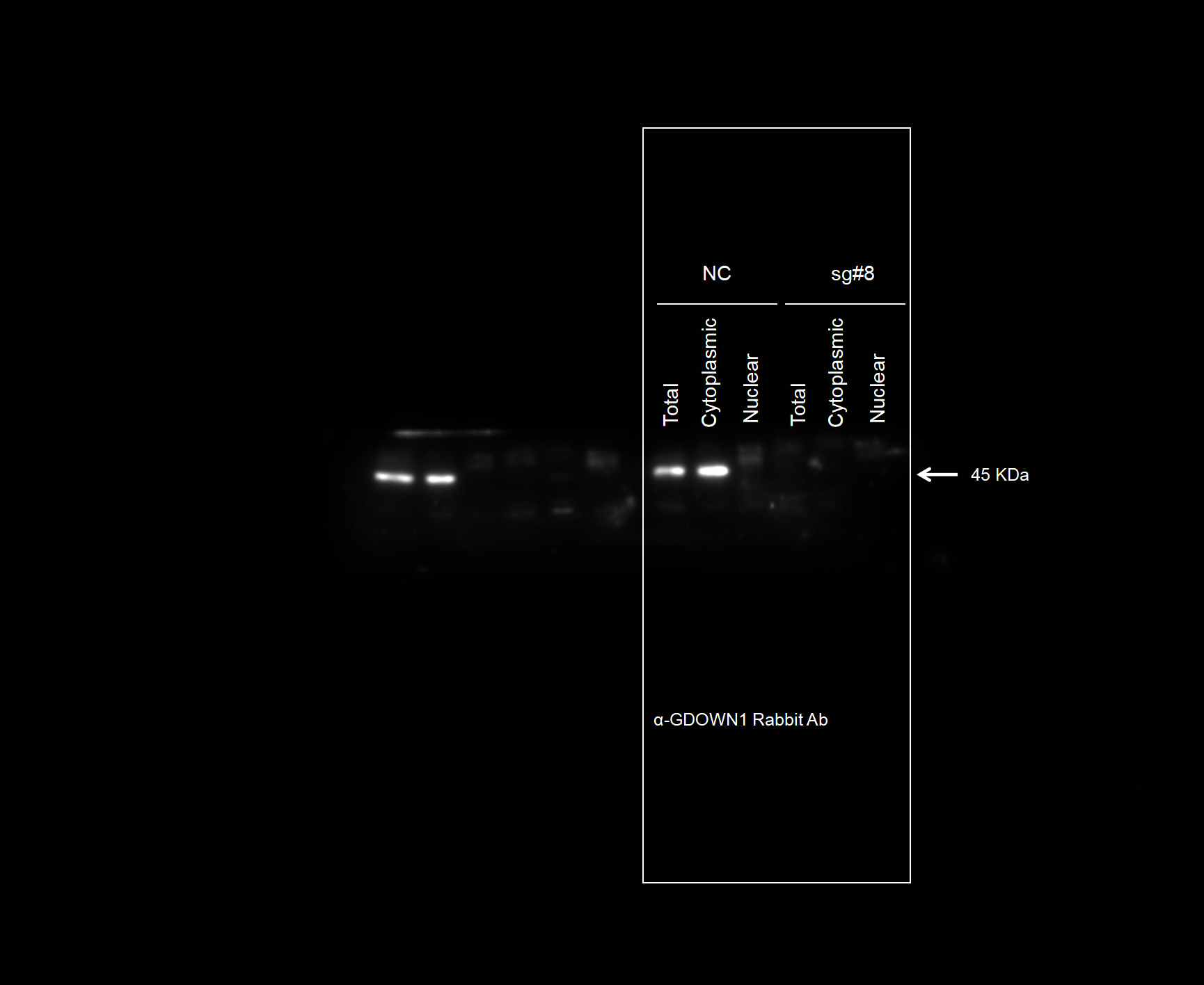

Supplement: Figure 1—source data 1. [file elife-79116-fig1-data1.zip › Figure 1-source data 1/+Label/Fig 1B-GDOWN1 rabbit antibody (for KO sg#8), Right.Tif.tif]

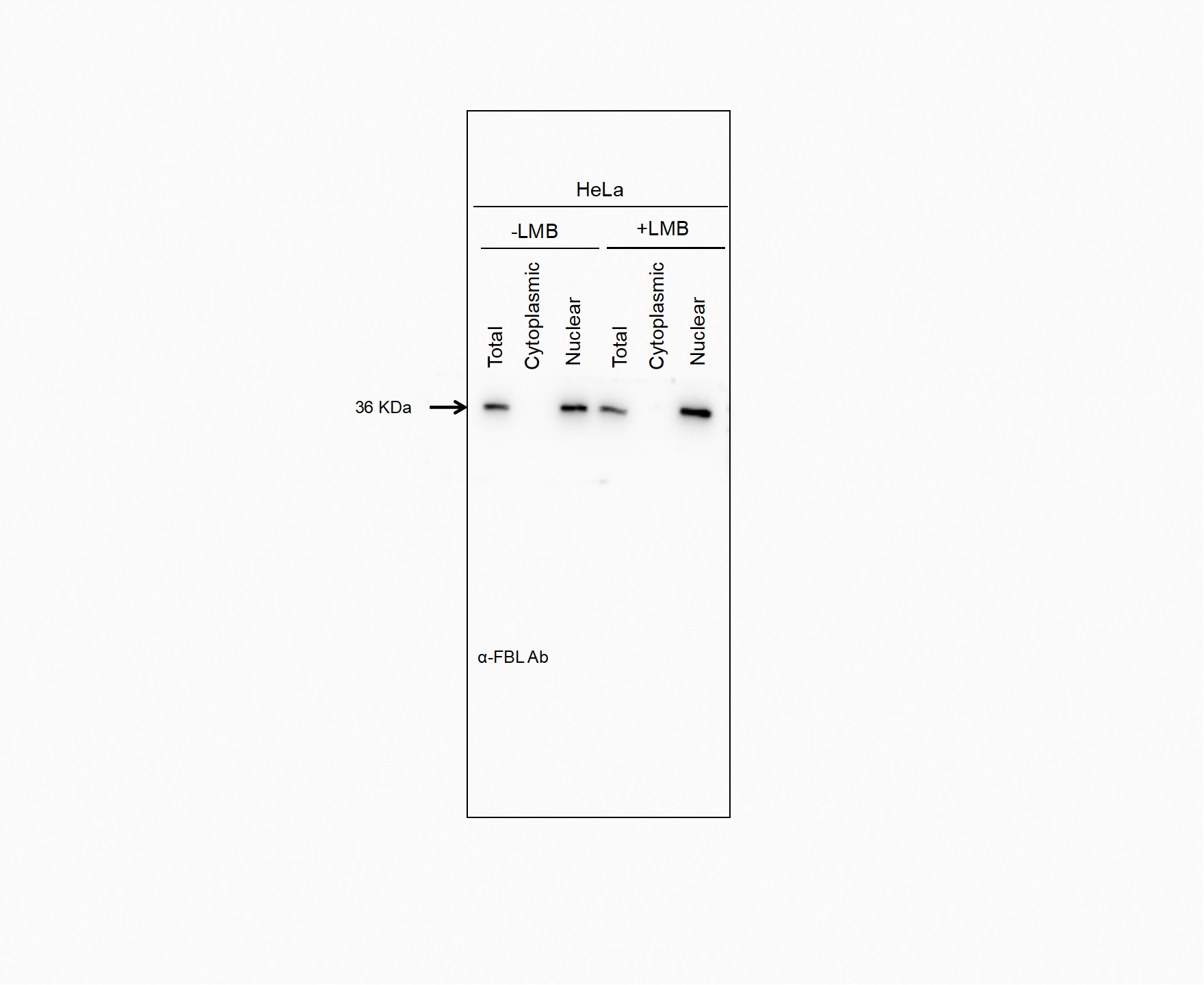

Supplement: Figure 1—source data 1. [file elife-79116-fig1-data1.zip › Figure 1-source data 1/+Label/Fig 1C-FBL antibody(For HeLa mock +LMB).Tif.tif]

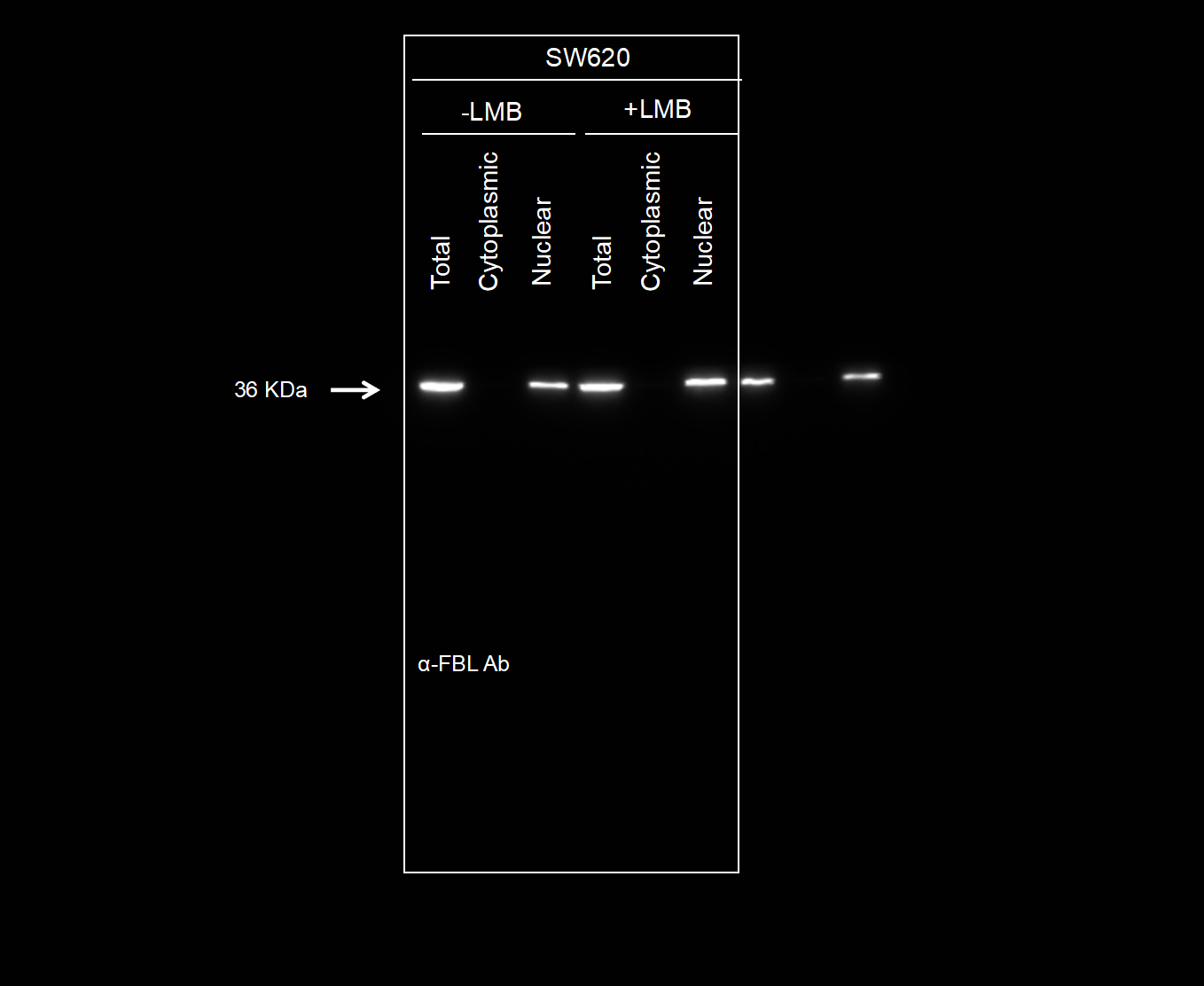

Supplement: Figure 1—source data 1. [file elife-79116-fig1-data1.zip › Figure 1-source data 1/+Label/Fig 1C-FBL antibody(For SW620 mock +LMB).Tif.tif]

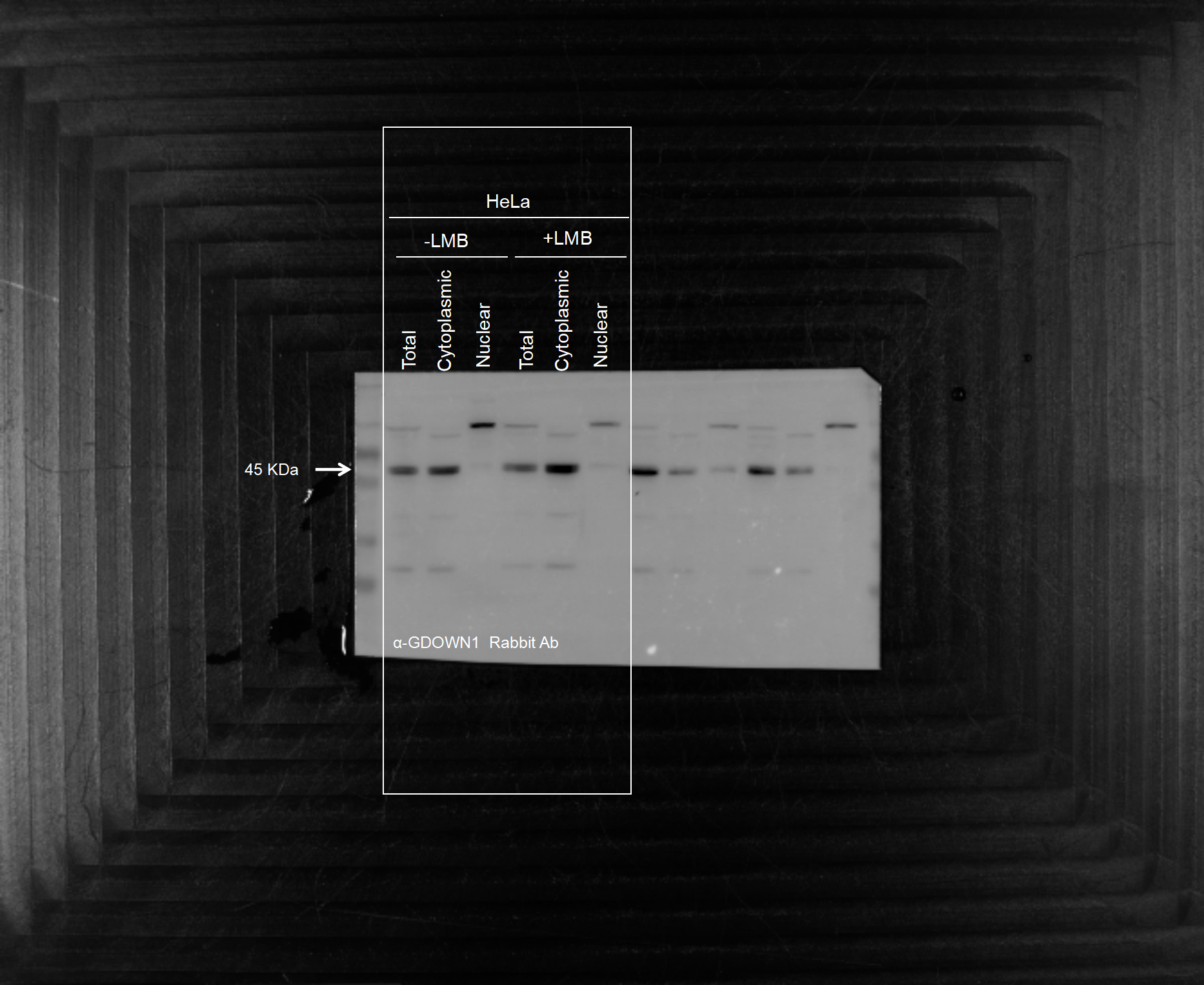

Supplement: Figure 1—source data 1. [file elife-79116-fig1-data1.zip › Figure 1-source data 1/+Label/Fig 1C-GDOWN1 rabbit antibody(For HeLa mock +LMB).Tif.tif]

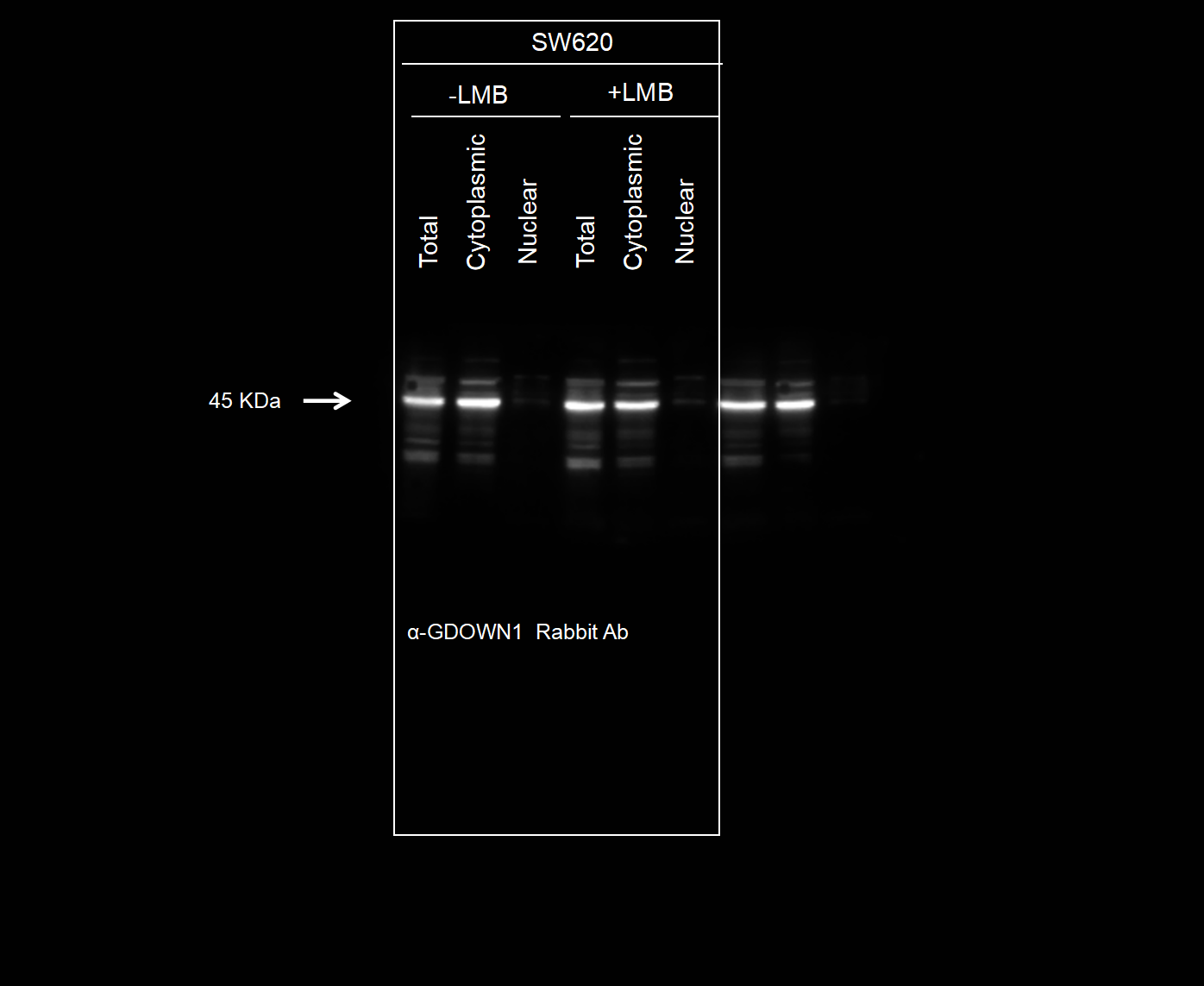

Supplement: Figure 1—source data 1. [file elife-79116-fig1-data1.zip › Figure 1-source data 1/+Label/Fig 1C-GDOWN1 rabbit antibody(For SW620 mock +LMB).Tif.tif]

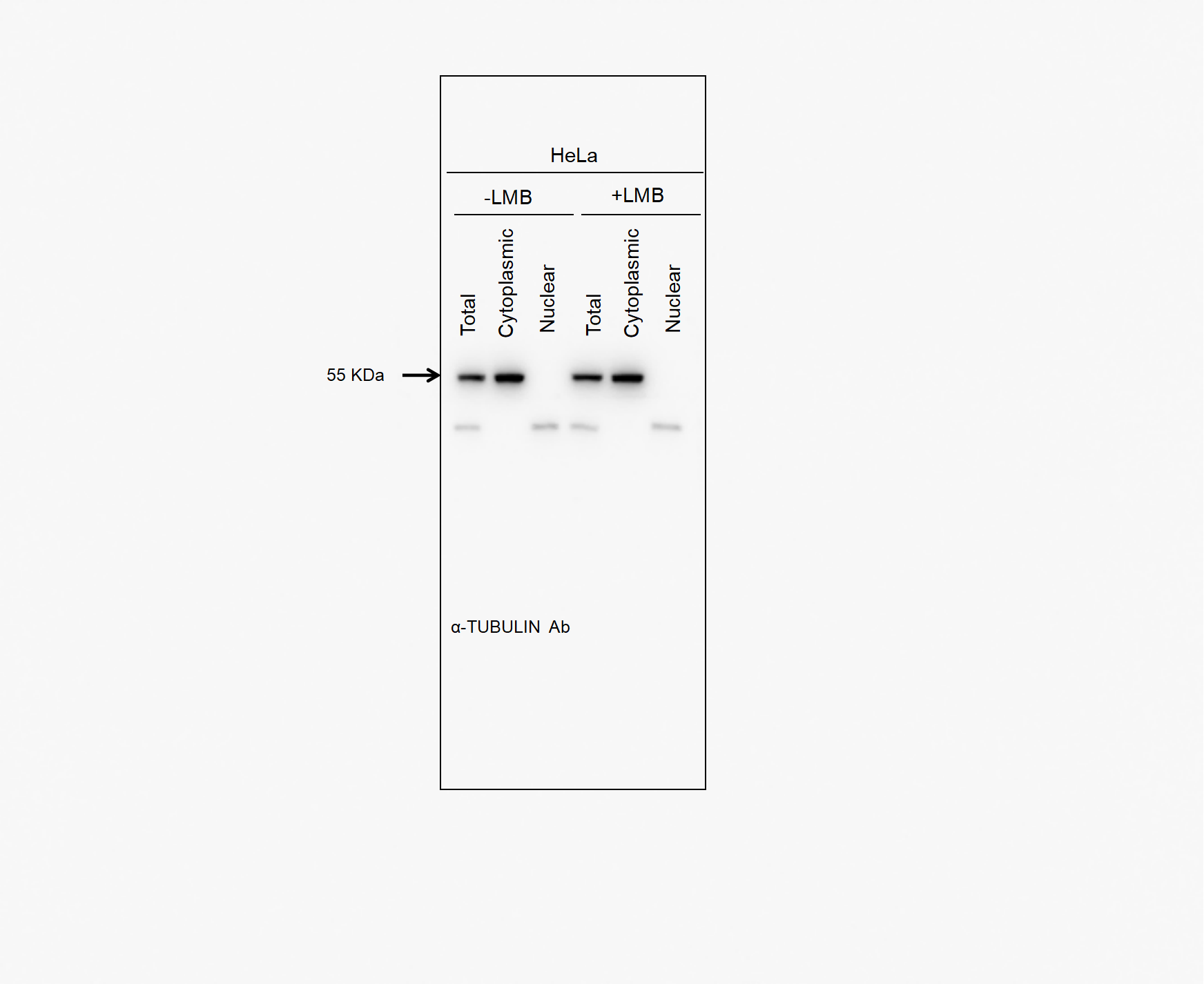

Supplement: Figure 1—source data 1. [file elife-79116-fig1-data1.zip › Figure 1-source data 1/+Label/Fig 1C-TUBULIN antibody(For HeLa mock +LMB).Tif.tif]

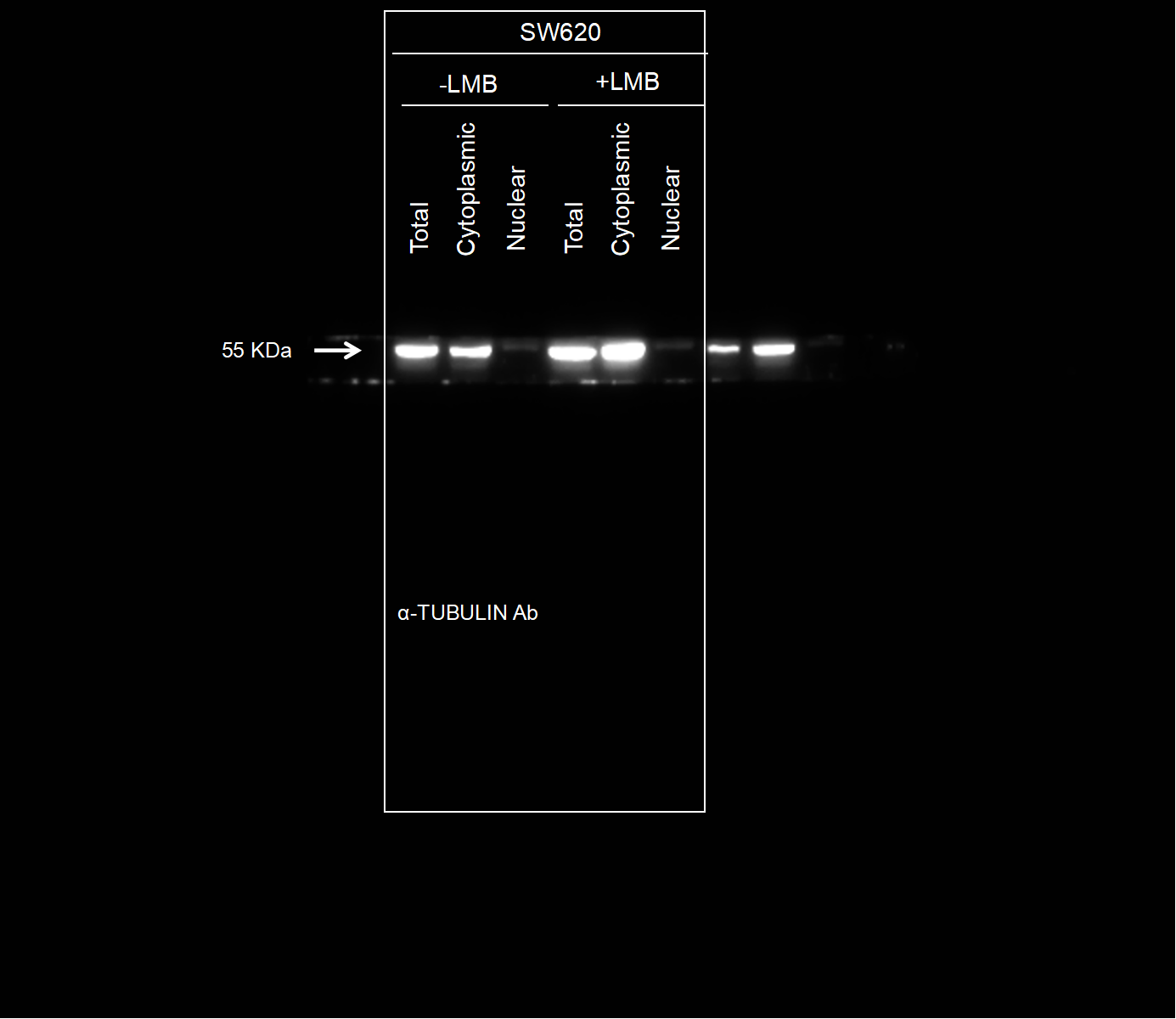

Supplement: Figure 1—source data 1. [file elife-79116-fig1-data1.zip › Figure 1-source data 1/+Label/Fig 1C-TUBULIN antibody(For SW620 mock +LMB).Tif.tif]

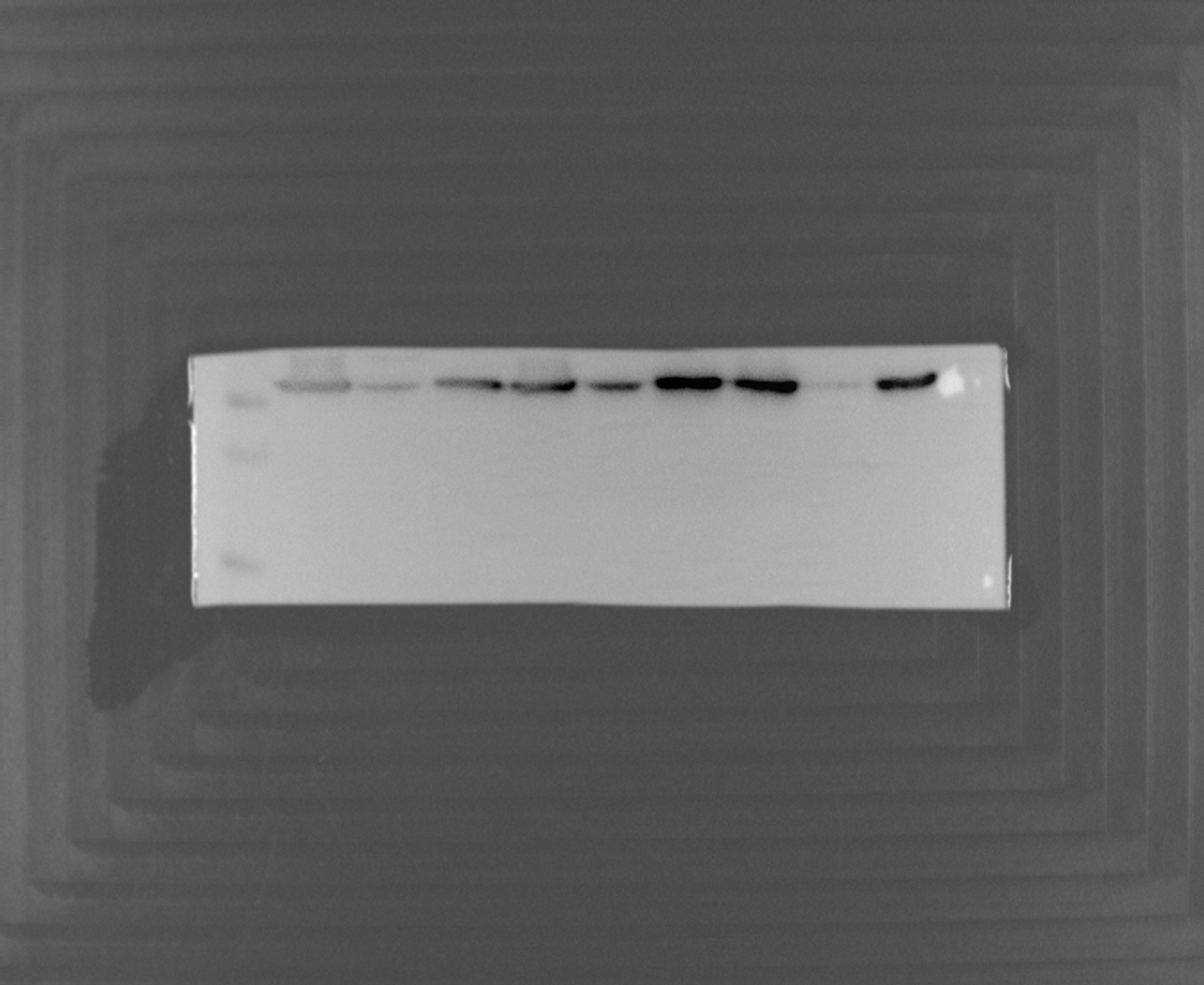

Supplement: Figure 1—source data 1. [file elife-79116-fig1-data1.zip › Figure 1-source data 1/unedited/Fig 1B-FBL antibody (For NIH3T3).Tif]

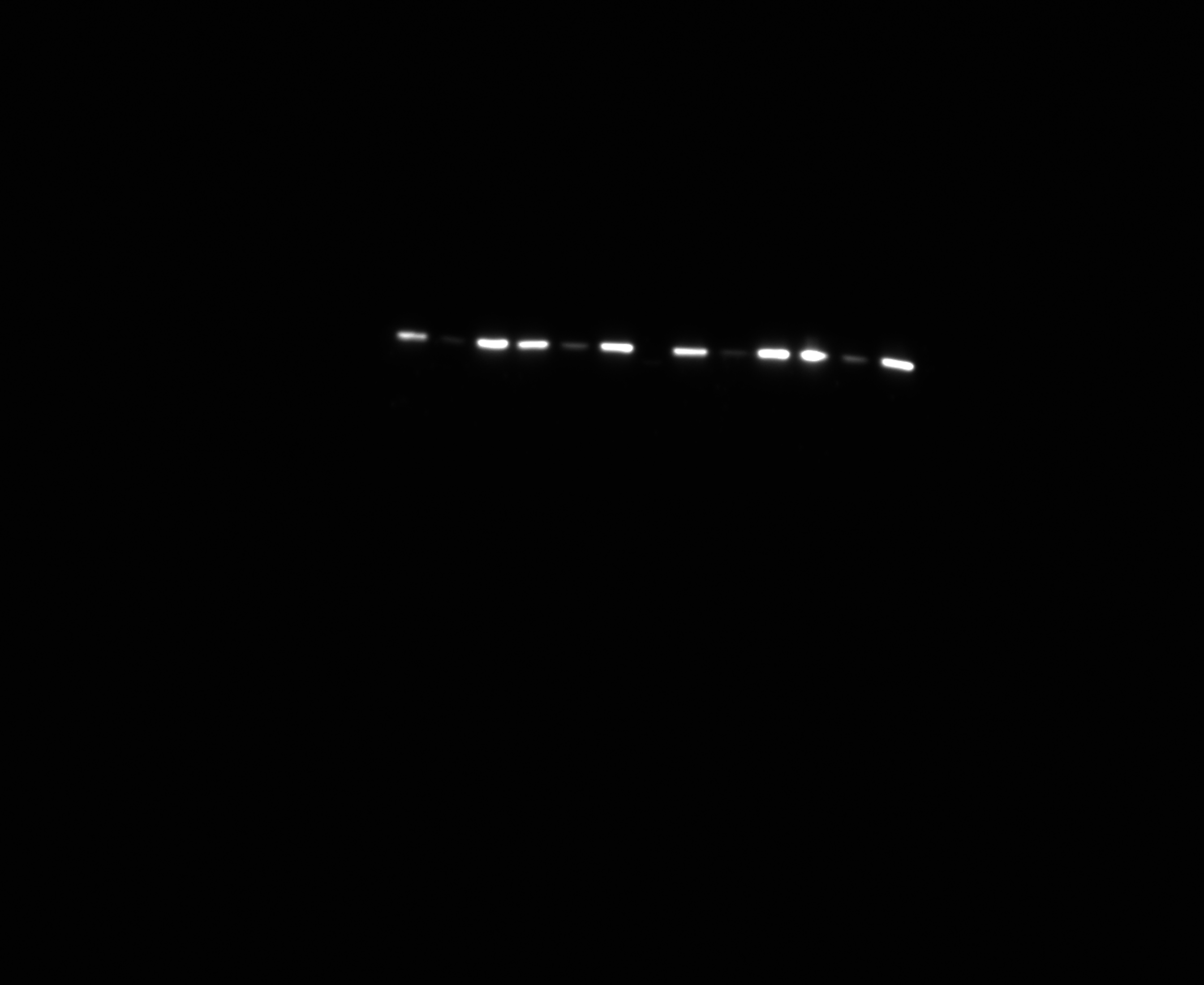

Supplement: Figure 1—source data 1. [file elife-79116-fig1-data1.zip › Figure 1-source data 1/unedited/Fig 1B-FBL antibody (for KO sg#8), Right.Tif]

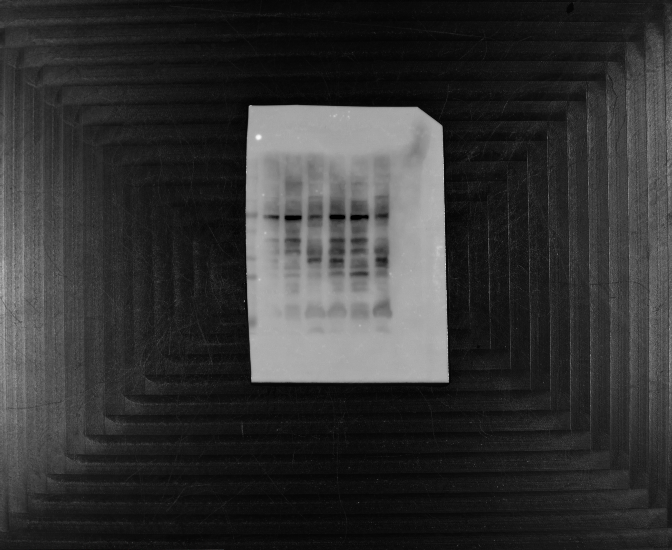

Supplement: Figure 1—source data 1. [file elife-79116-fig1-data1.zip › Figure 1-source data 1/unedited/Fig 1B-GDOWN1 rabbit antibody (For E14TG2a).Tif]

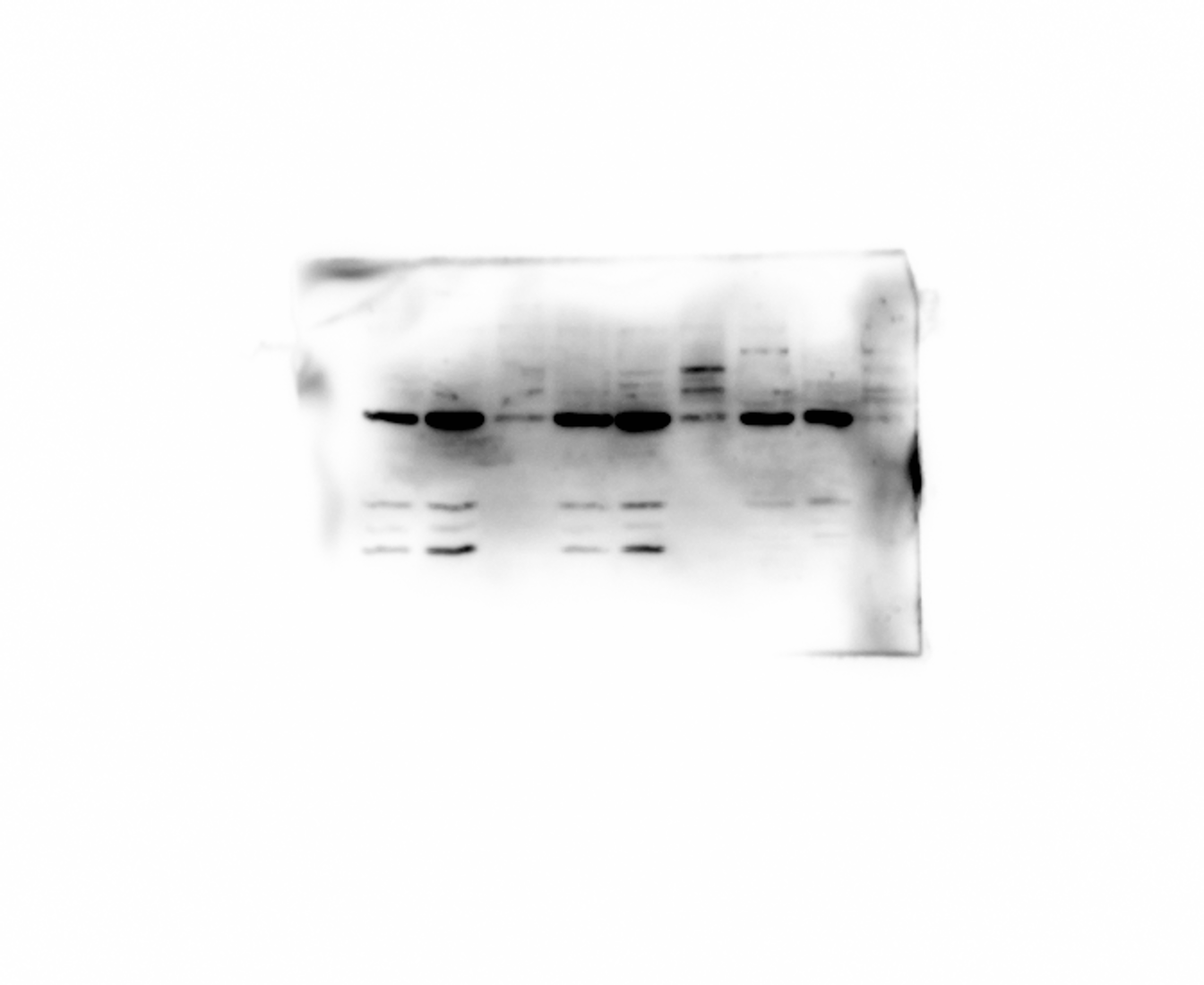

Supplement: Figure 1—source data 1. [file elife-79116-fig1-data1.zip › Figure 1-source data 1/unedited/Fig 1B-GDOWN1 rabbit antibody (For NIH3T3).Tif]

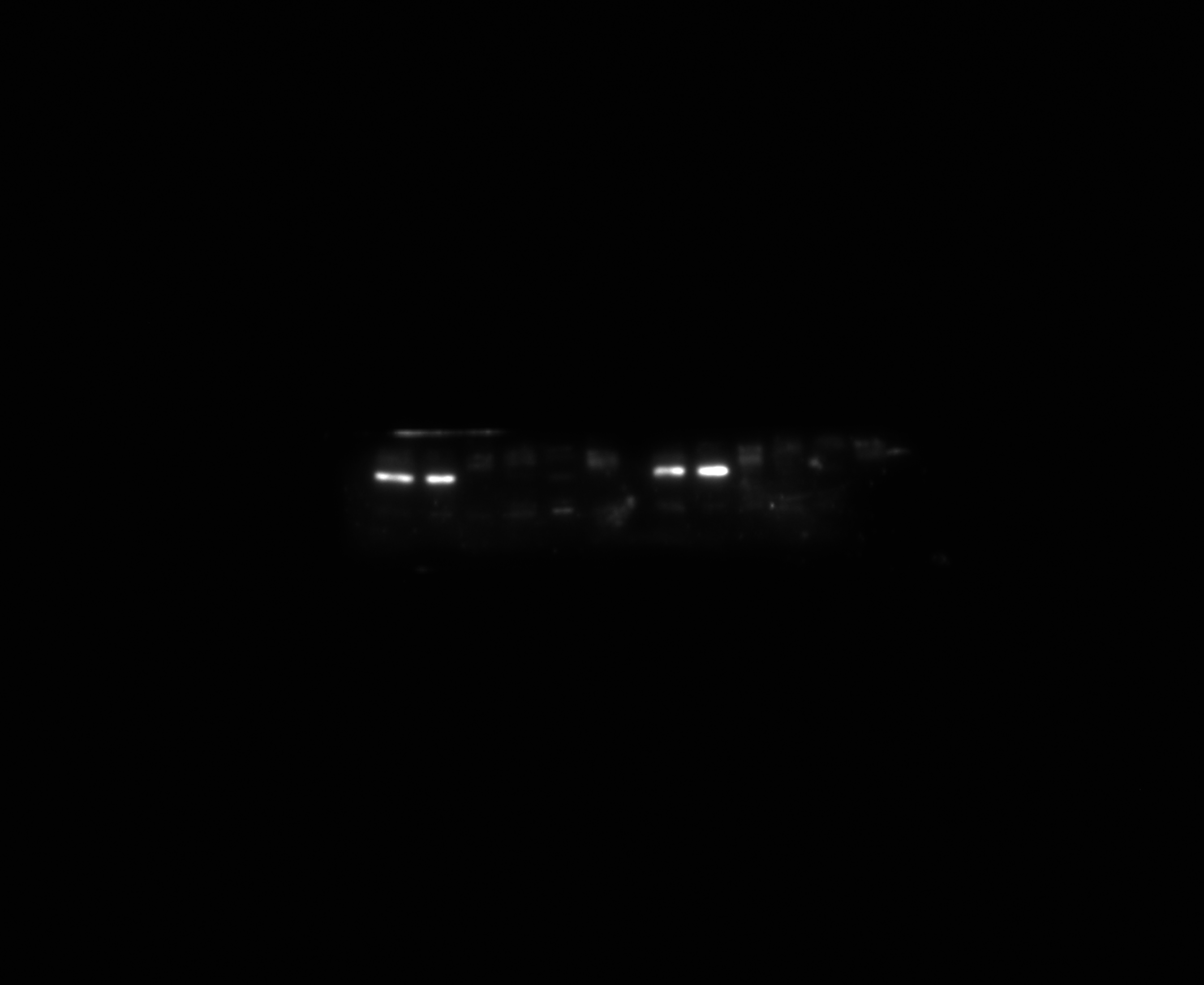

Supplement: Figure 1—source data 1. [file elife-79116-fig1-data1.zip › Figure 1-source data 1/unedited/Fig 1B-GDOWN1 rabbit antibody (for KO sg#8), Right.Tif]

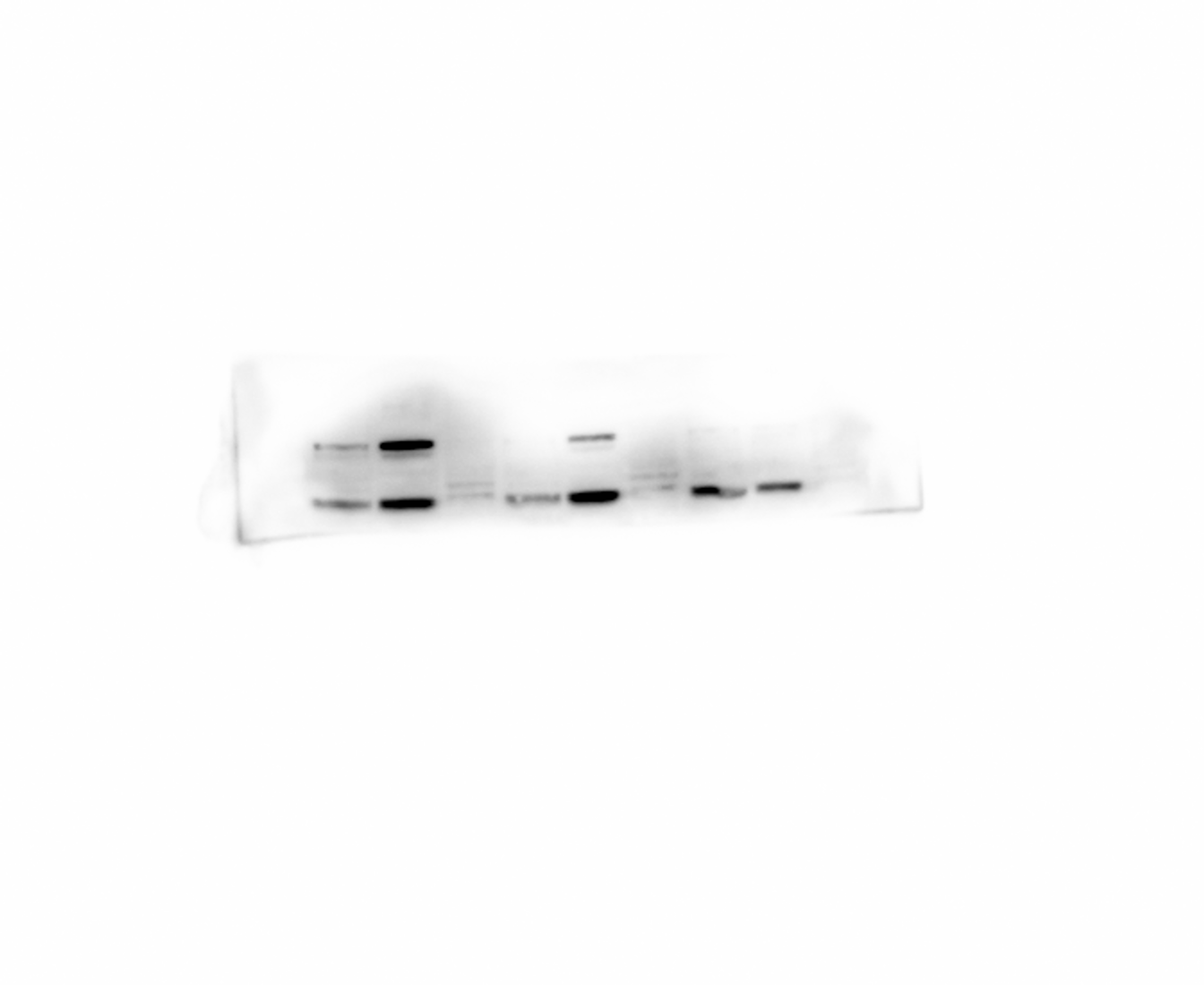

Supplement: Figure 1—source data 1. [file elife-79116-fig1-data1.zip › Figure 1-source data 1/unedited/Fig 1B-TUBULIN antibody (For NIH3T3).Tif]

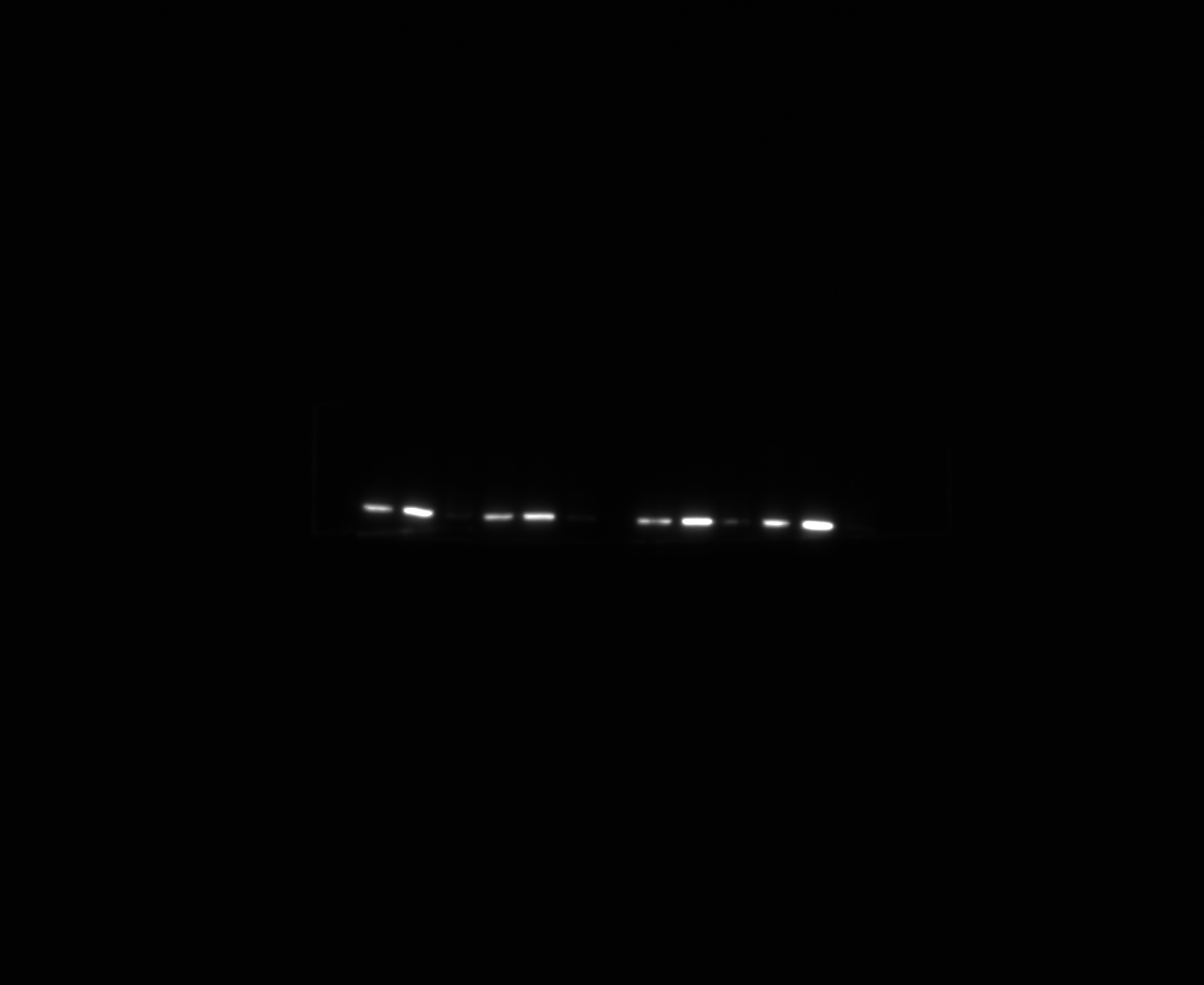

Supplement: Figure 1—source data 1. [file elife-79116-fig1-data1.zip › Figure 1-source data 1/unedited/Fig 1B-TUBULIN antibody (for KO sg#8), Right.Tif]

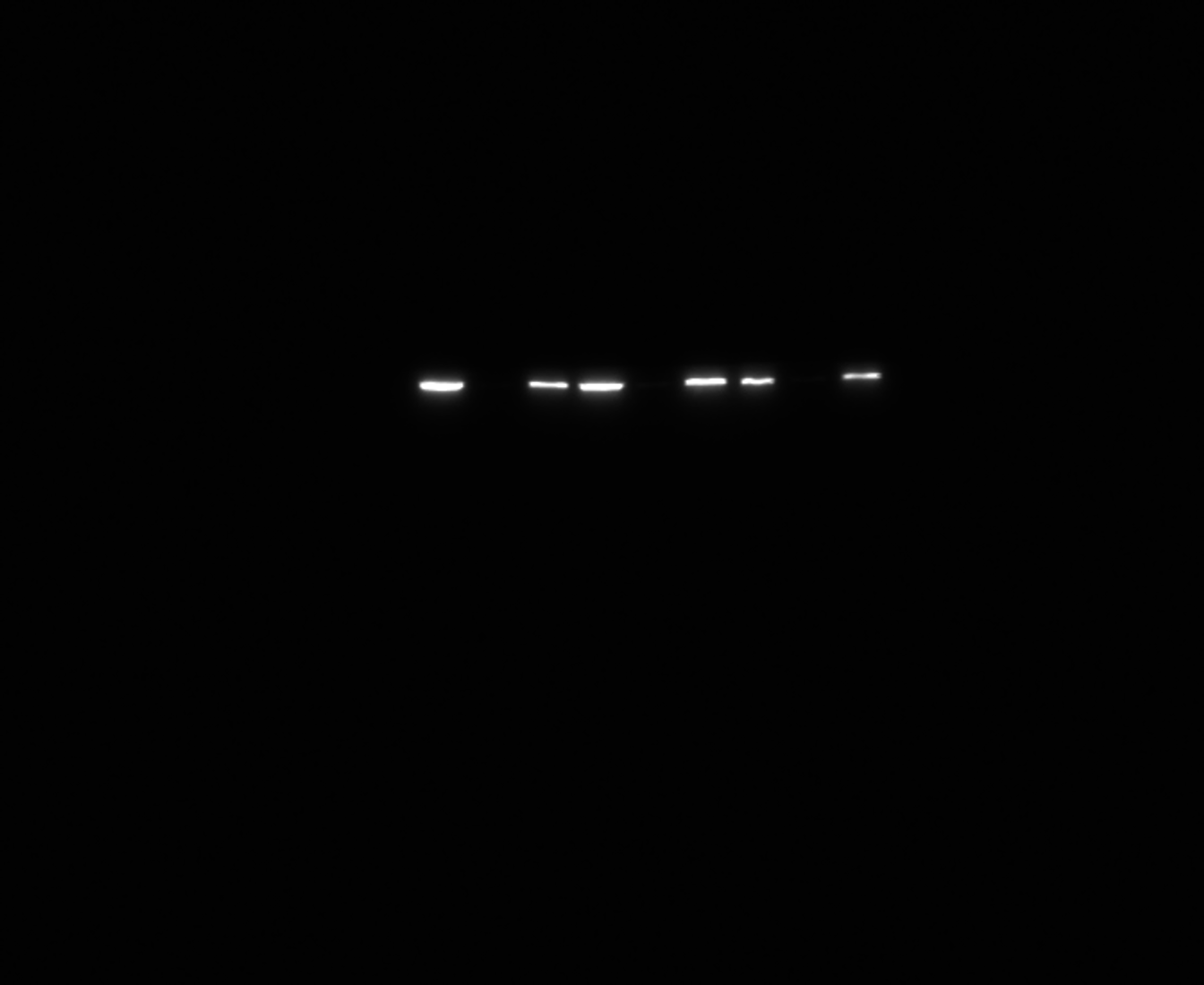

Supplement: Figure 1—source data 1. [file elife-79116-fig1-data1.zip › Figure 1-source data 1/unedited/Fig 1C-FBL antibody (For SW620 mock +LMB).Tif]

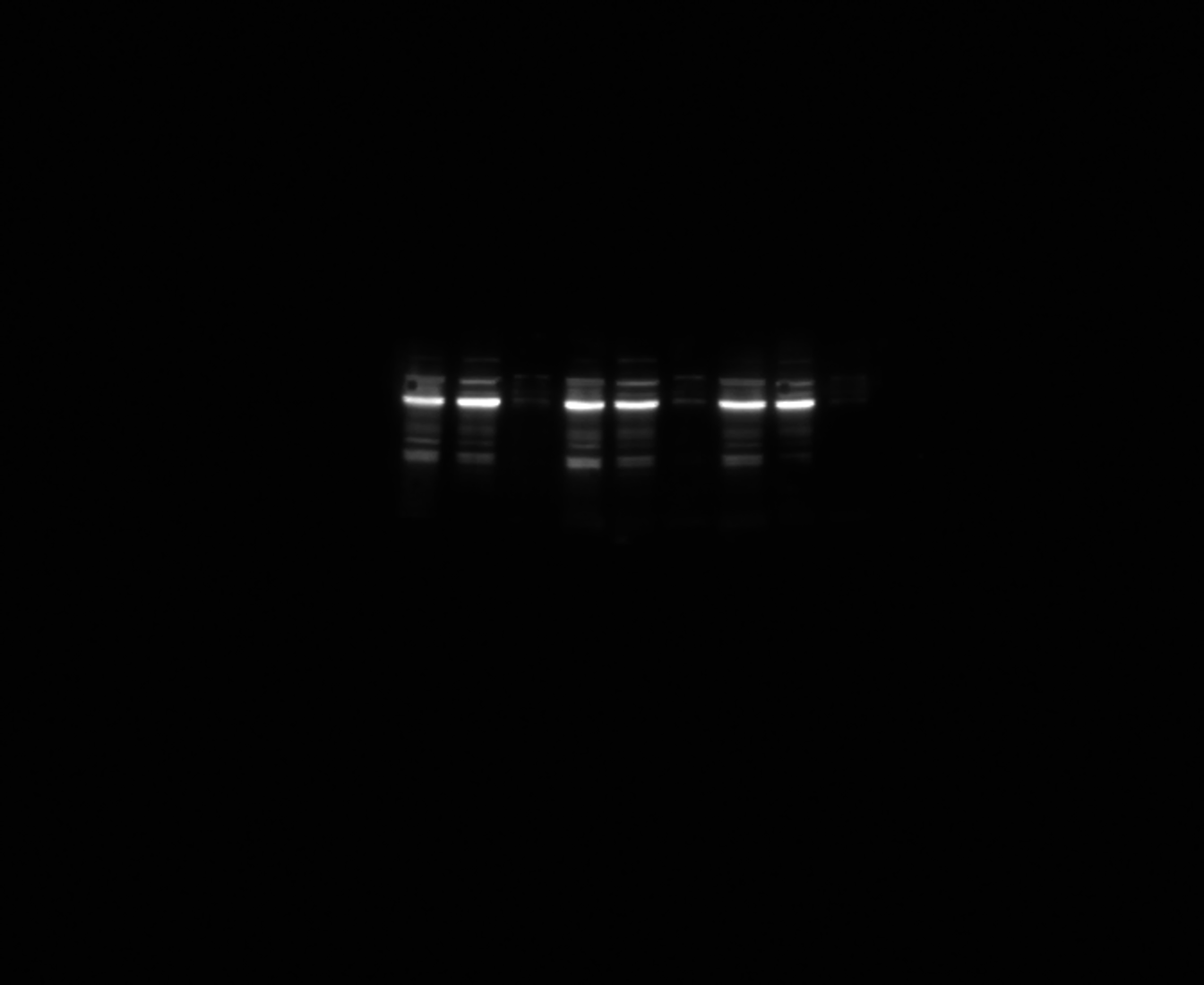

Supplement: Figure 1—source data 1. [file elife-79116-fig1-data1.zip › Figure 1-source data 1/unedited/Fig 1C-GDOWN1 antibody (For SW620 mock +LMB).Tif]

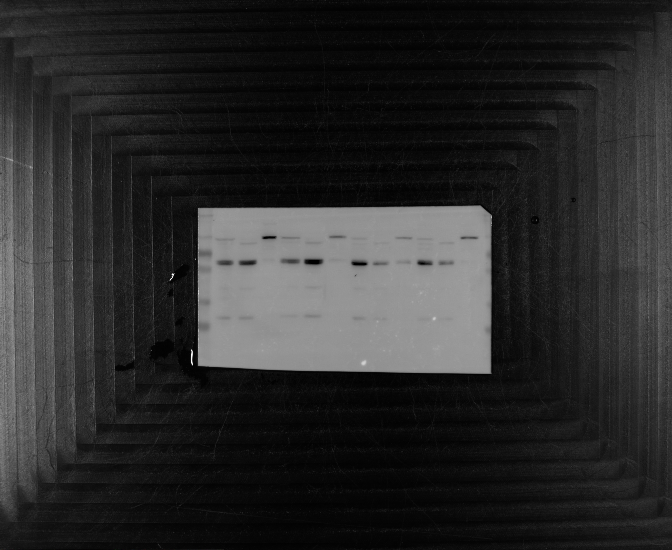

Supplement: Figure 1—source data 1. [file elife-79116-fig1-data1.zip › Figure 1-source data 1/unedited/Fig 1C-GDOWN1 rabbit antibody(For HeLa mock +LMB).Tif]

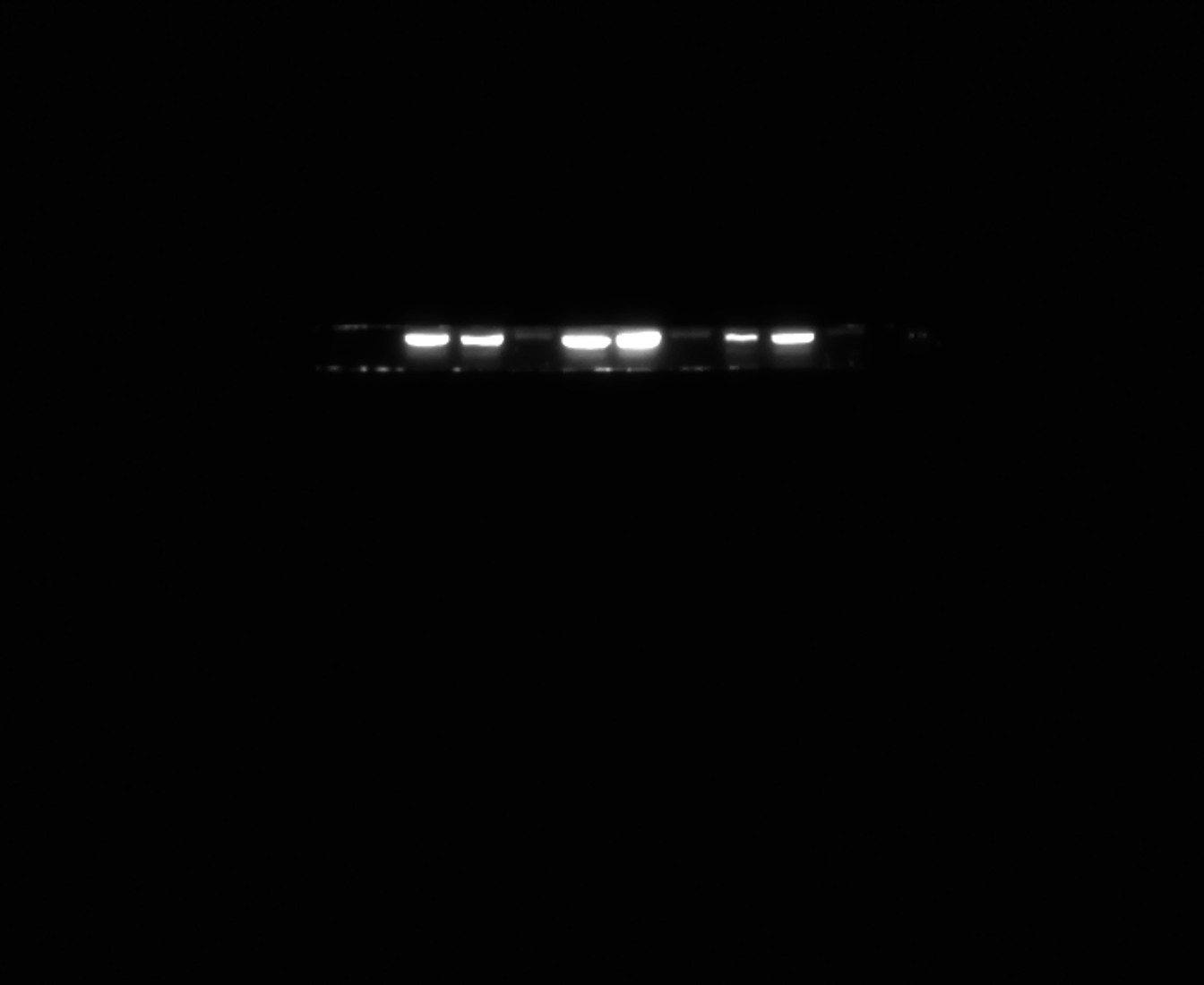

Supplement: Figure 1—source data 1. [file elife-79116-fig1-data1.zip › Figure 1-source data 1/unedited/Fig 1C-TUBULIN antibody (For SW620 mock +LMB).Tif]

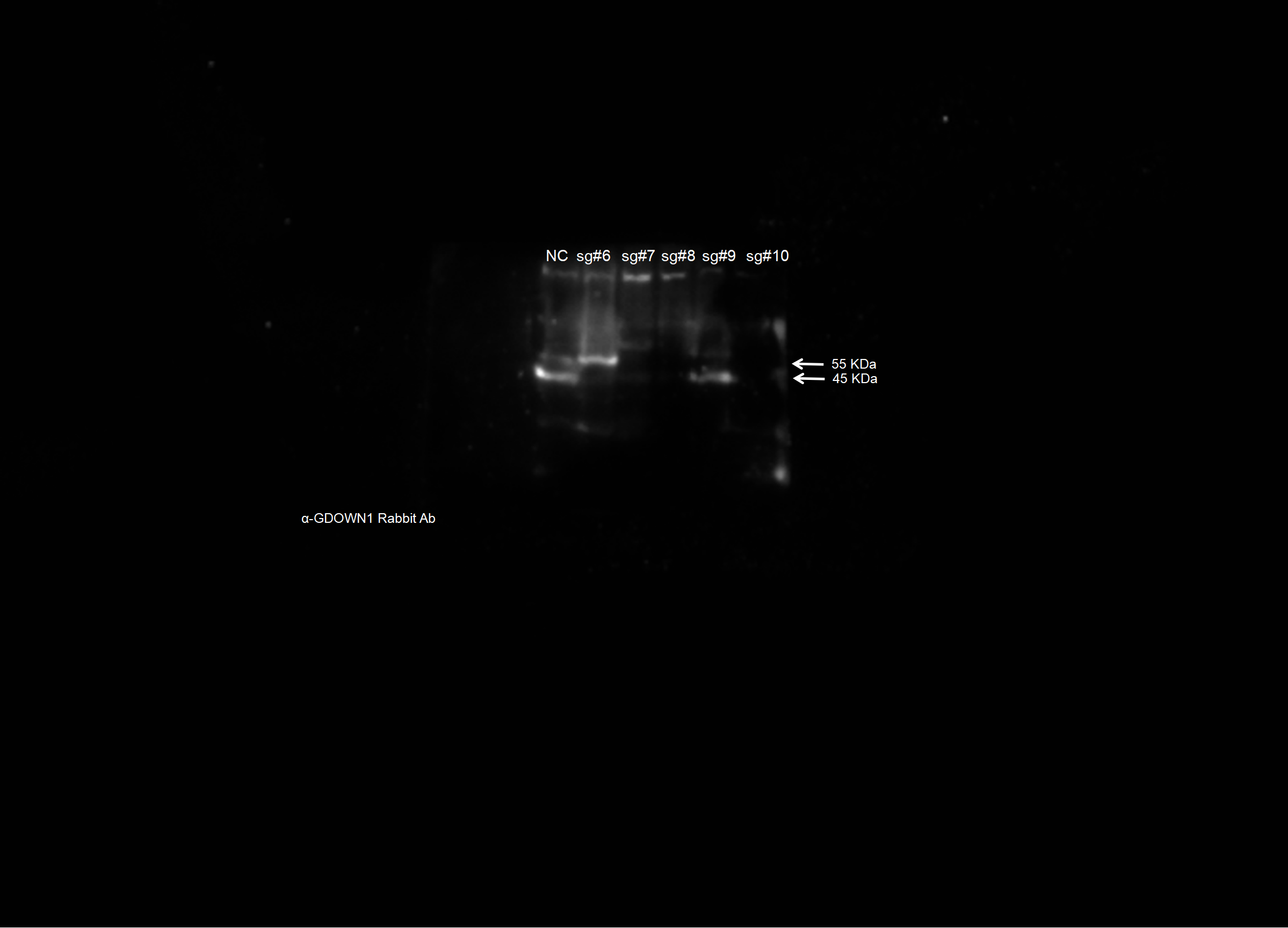

Supplement: Figure 1—figure supplement 1—source data 1. [file elife-79116-fig1-figsupp1-data1.zip › Figure 1-figure supplement 1-source data 1/+Label/Fig 1-Fig supplement 1A-GDOWN1 rabbit antibody(For sg#6.tif]

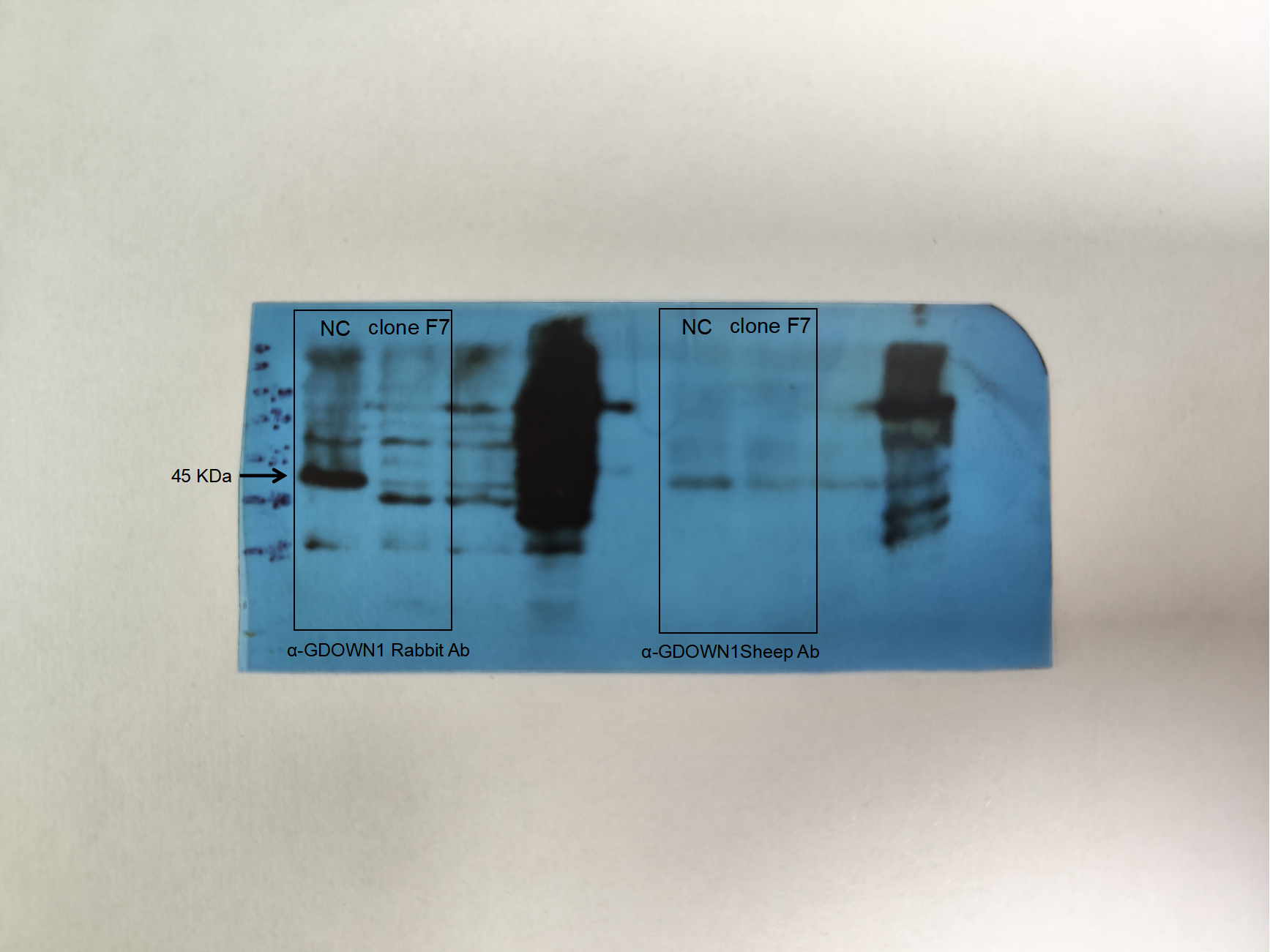

Supplement: Figure 1—figure supplement 1—source data 1. [file elife-79116-fig1-figsupp1-data1.zip › Figure 1-figure supplement 1-source data 1/+Label/Fig 1-Fig supplement 1A-GDOWN1 sheep and rabbit antibod.png]

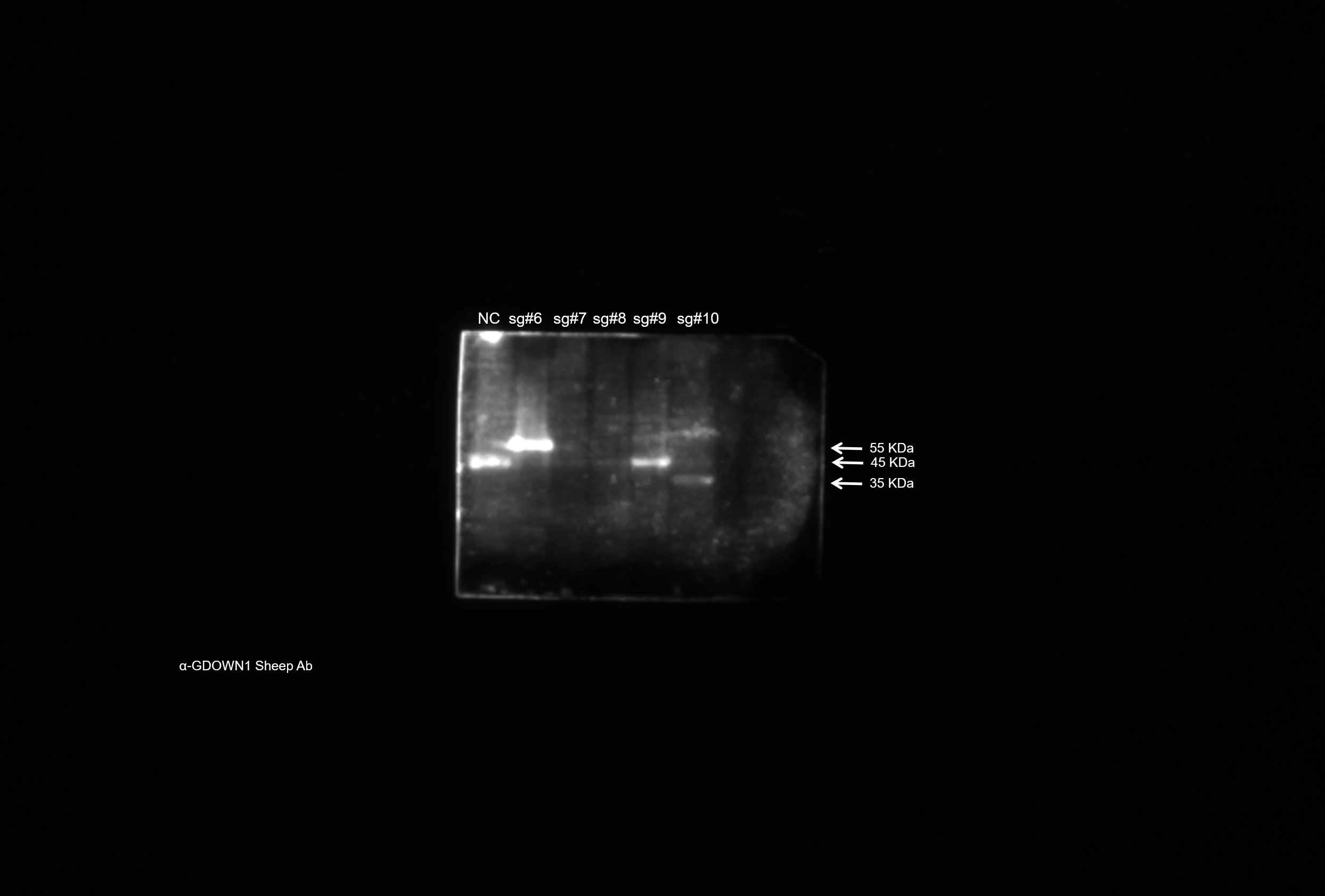

Supplement: Figure 1—figure supplement 1—source data 1. [file elife-79116-fig1-figsupp1-data1.zip › Figure 1-figure supplement 1-source data 1/+Label/Fig 1-Fig supplement 1A-GDOWN1 sheep antibody(For sg#6.tif]

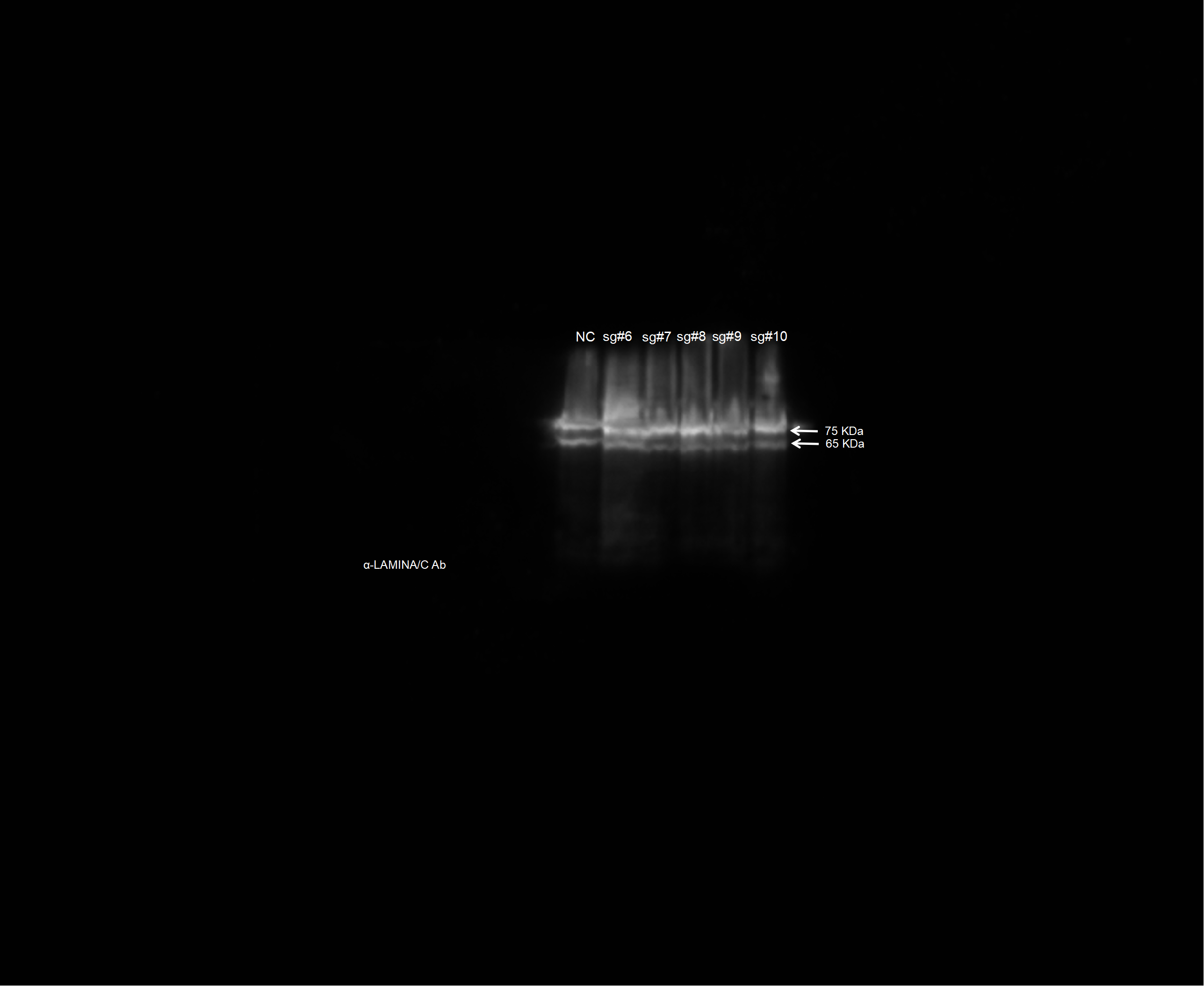

Supplement: Figure 1—figure supplement 1—source data 1. [file elife-79116-fig1-figsupp1-data1.zip › Figure 1-figure supplement 1-source data 1/+Label/Fig 1-Fig supplement 1A-LAMNA C antibody(For sg#6 7 8 9 10).Tif.tif]

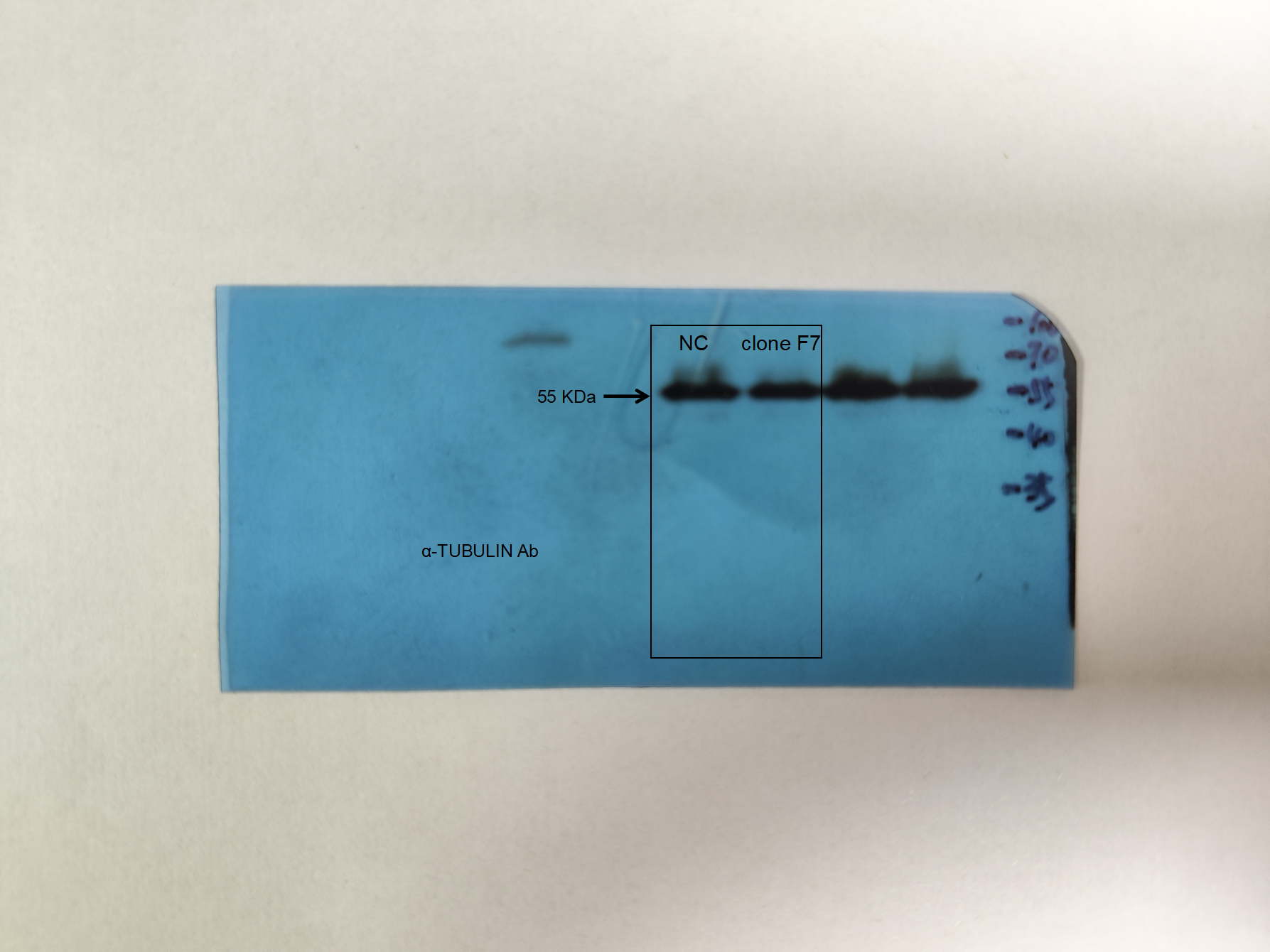

Supplement: Figure 1—figure supplement 1—source data 1. [file elife-79116-fig1-figsupp1-data1.zip › Figure 1-figure supplement 1-source data 1/+Label/Fig 1-Fig supplement 1A-TUBULIN (For clone F7).png]

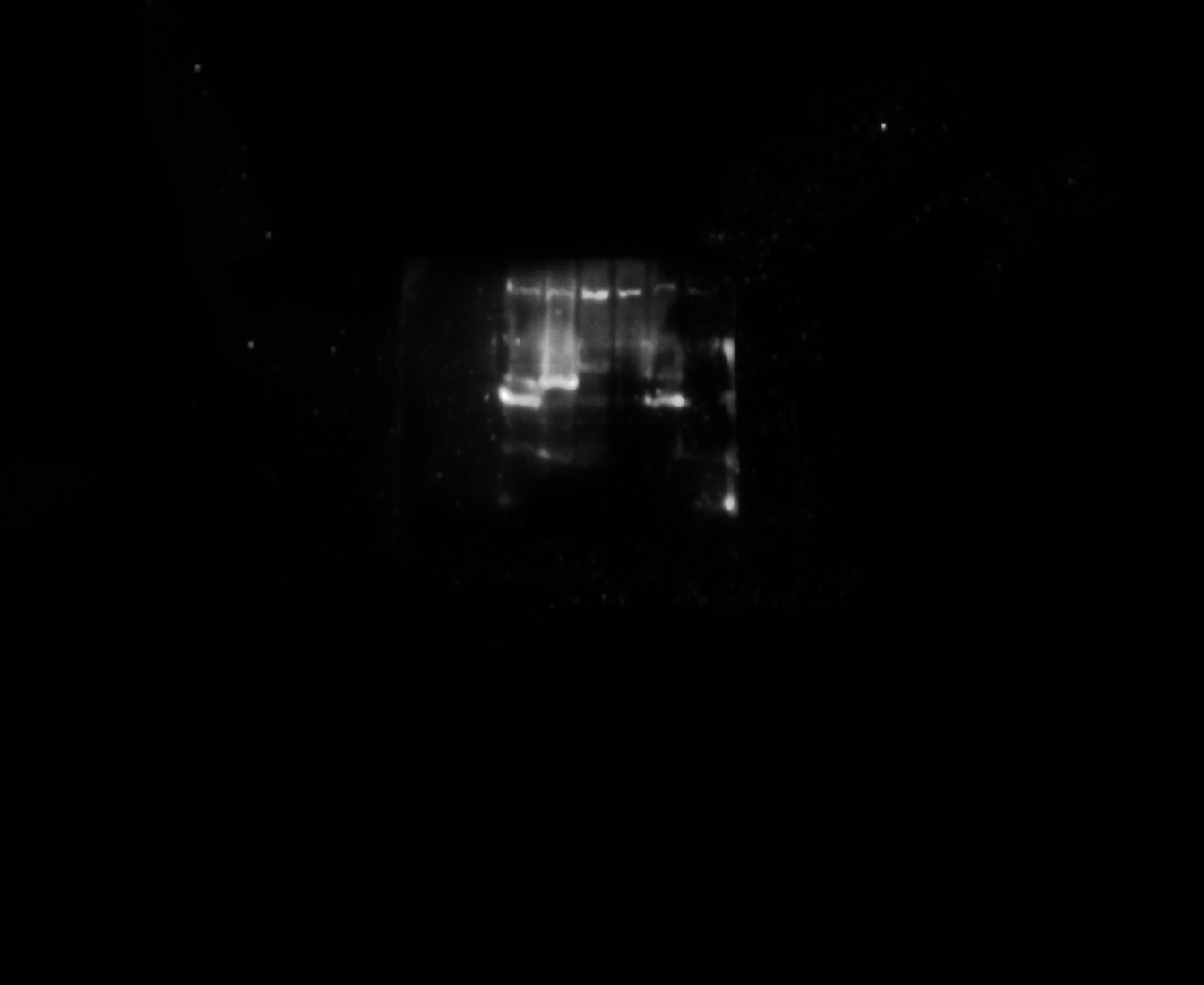

Supplement: Figure 1—figure supplement 1—source data 1. [file elife-79116-fig1-figsupp1-data1.zip › Figure 1-figure supplement 1-source data 1/Unedited/Fig 1-Fig supplement 1A-GDOWN1 rabbit antibody(For sg#6 7 8 9 10).Tif]

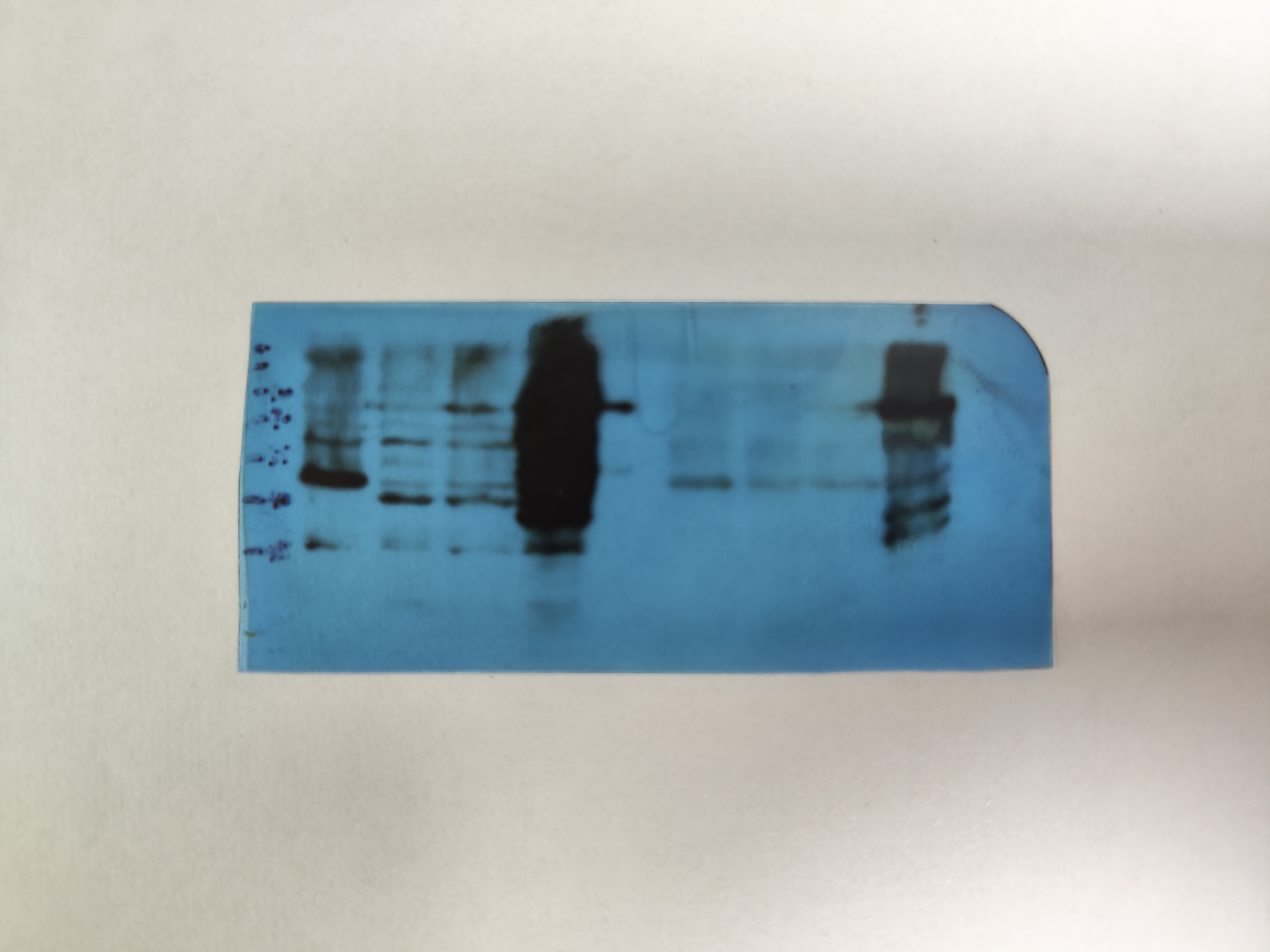

Supplement: Figure 1—figure supplement 1—source data 1. [file elife-79116-fig1-figsupp1-data1.zip › Figure 1-figure supplement 1-source data 1/Unedited/Fig 1-Fig supplement 1A-GDOWN1 sheep and rabbit antibody (For clone F7).jpg]

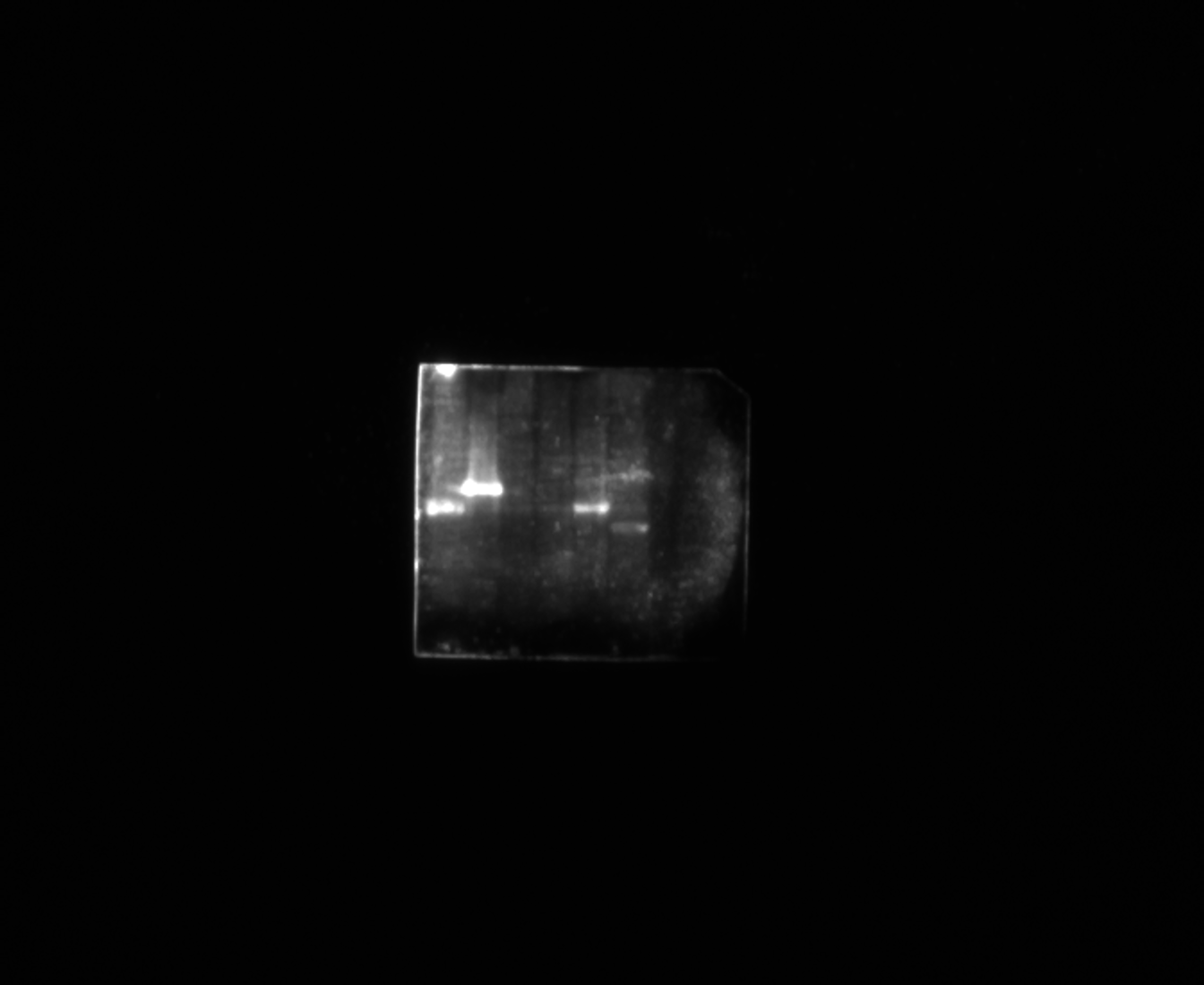

Supplement: Figure 1—figure supplement 1—source data 1. [file elife-79116-fig1-figsupp1-data1.zip › Figure 1-figure supplement 1-source data 1/Unedited/Fig 1-Fig supplement 1A-GDOWN1 sheep antibody(For sg#6 7 8 9 10).Tif]

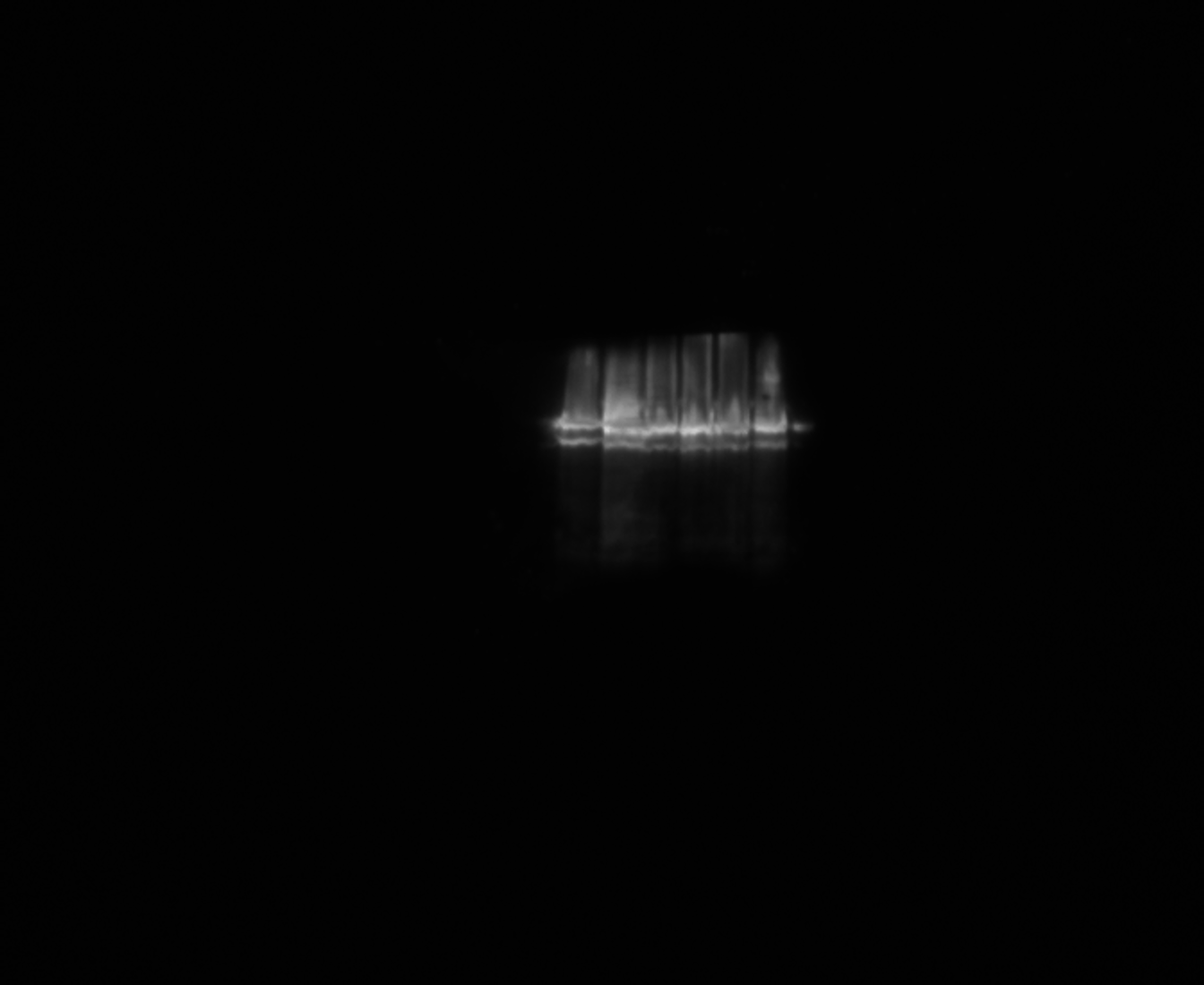

Supplement: Figure 1—figure supplement 1—source data 1. [file elife-79116-fig1-figsupp1-data1.zip › Figure 1-figure supplement 1-source data 1/Unedited/Fig 1-Fig supplement 1A-LAMINAC (For sg#6 7 8 9 10).Tif]

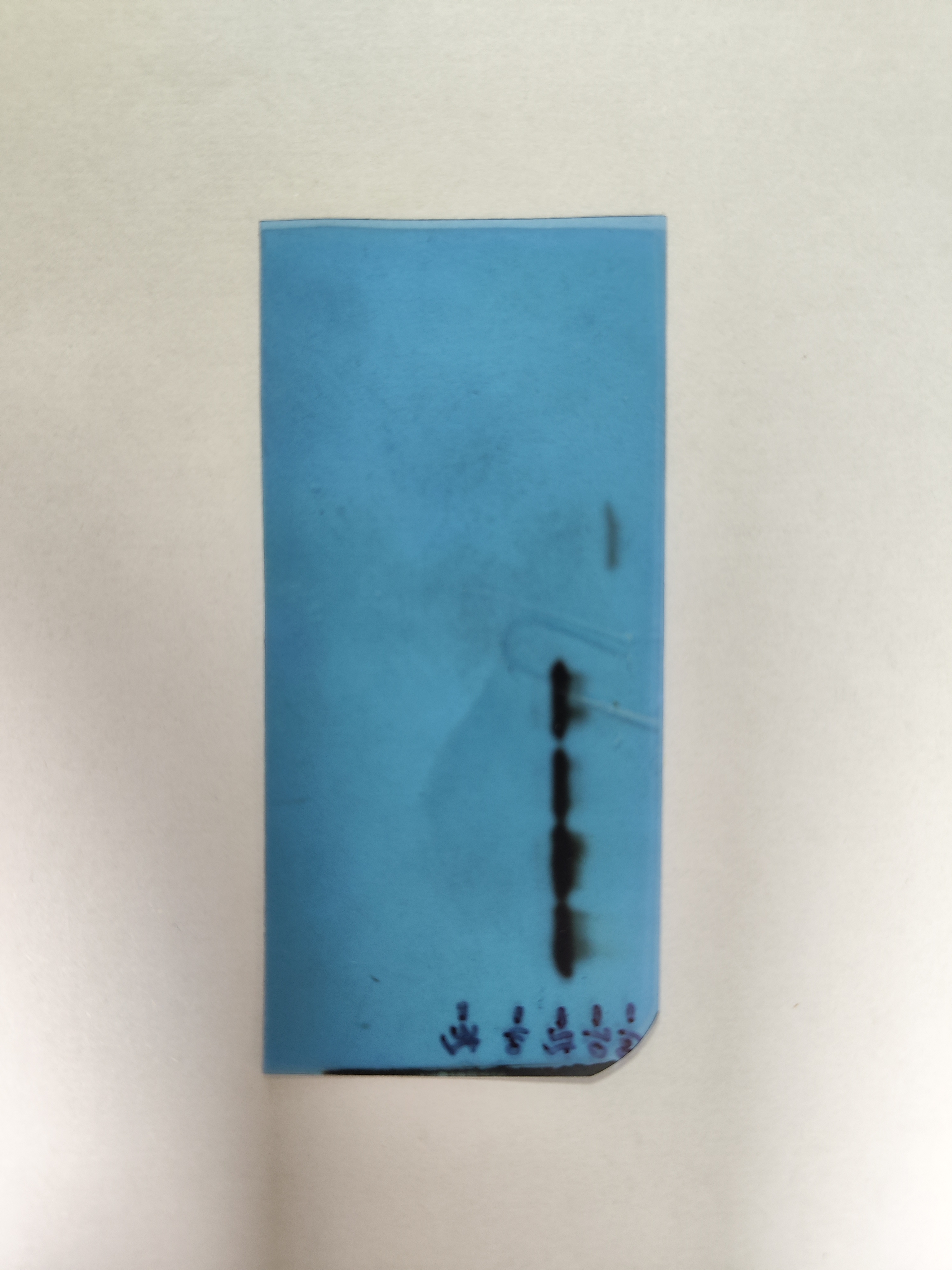

Supplement: Figure 1—figure supplement 1—source data 1. [file elife-79116-fig1-figsupp1-data1.zip › Figure 1-figure supplement 1-source data 1/Unedited/Fig 1-Fig supplement 1A-TUBULIN (For clone F7).jpg]

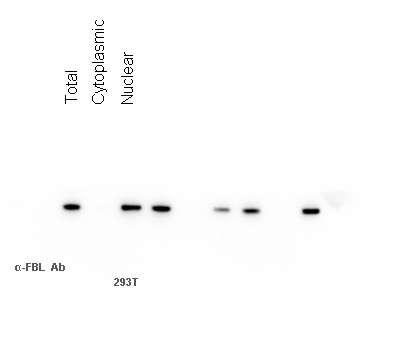

Supplement: Figure 1—figure supplement 1—source data 2. [file elife-79116-fig1-figsupp1-data2.zip › Figure 1-figure supplement 1-source data 2/+Label/Fig 1-Fig supplement 1B-FBL (293T), Left.tif]

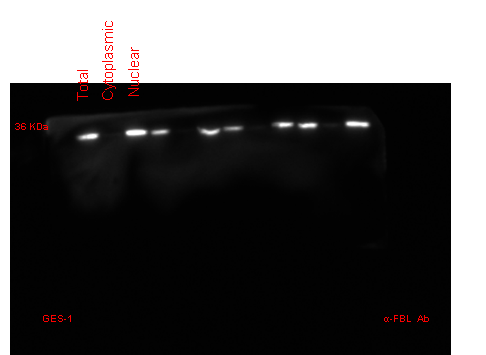

Supplement: Figure 1—figure supplement 1—source data 2. [file elife-79116-fig1-figsupp1-data2.zip › Figure 1-figure supplement 1-source data 2/+Label/Fig 1-Fig supplement 1B-FBL (GES-1), Left.tif]

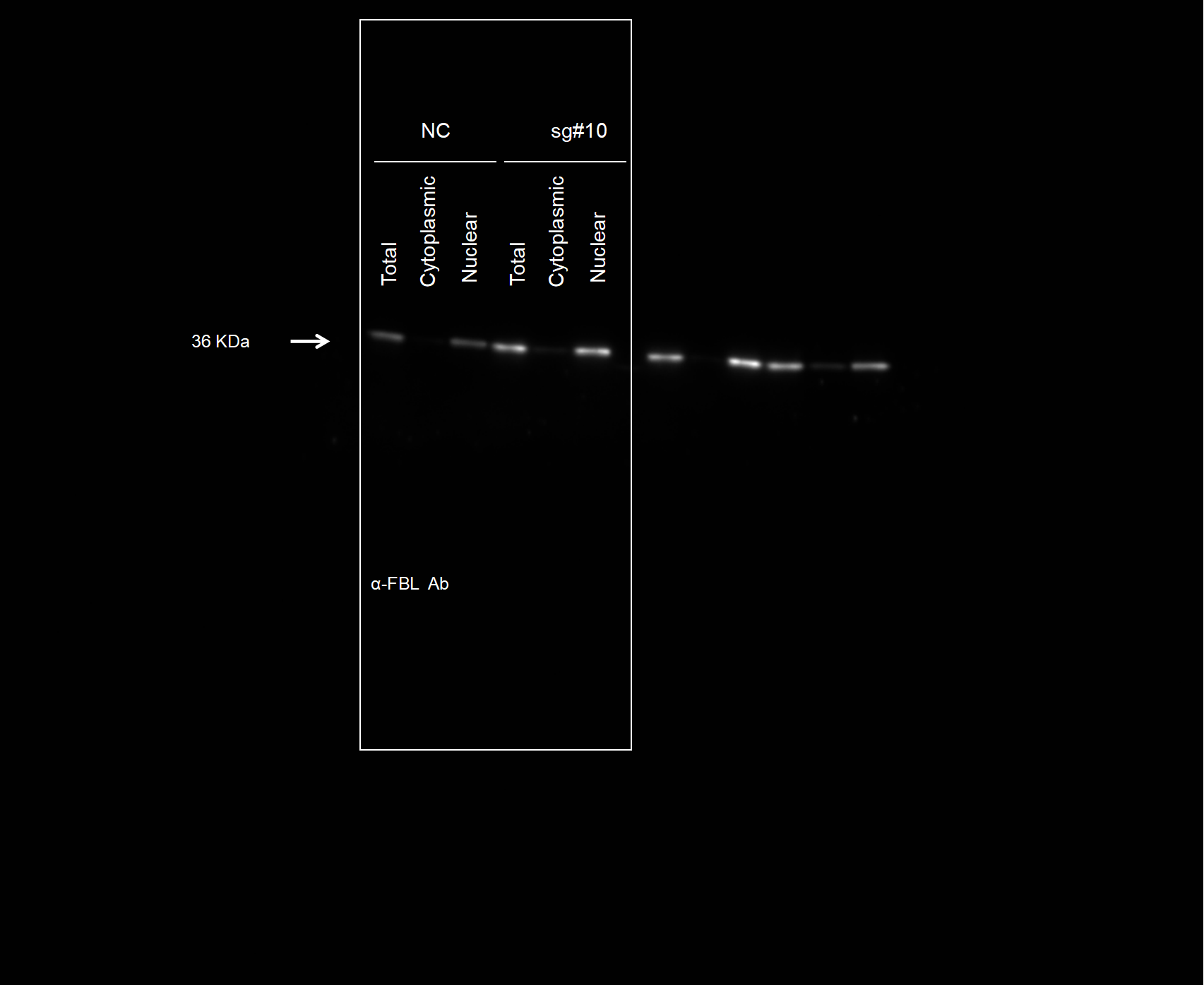

Supplement: Figure 1—figure supplement 1—source data 2. [file elife-79116-fig1-figsupp1-data2.zip › Figure 1-figure supplement 1-source data 2/+Label/Fig 1-Fig supplement 1B-FBL (KO sg#10), Left.Tif.tif]

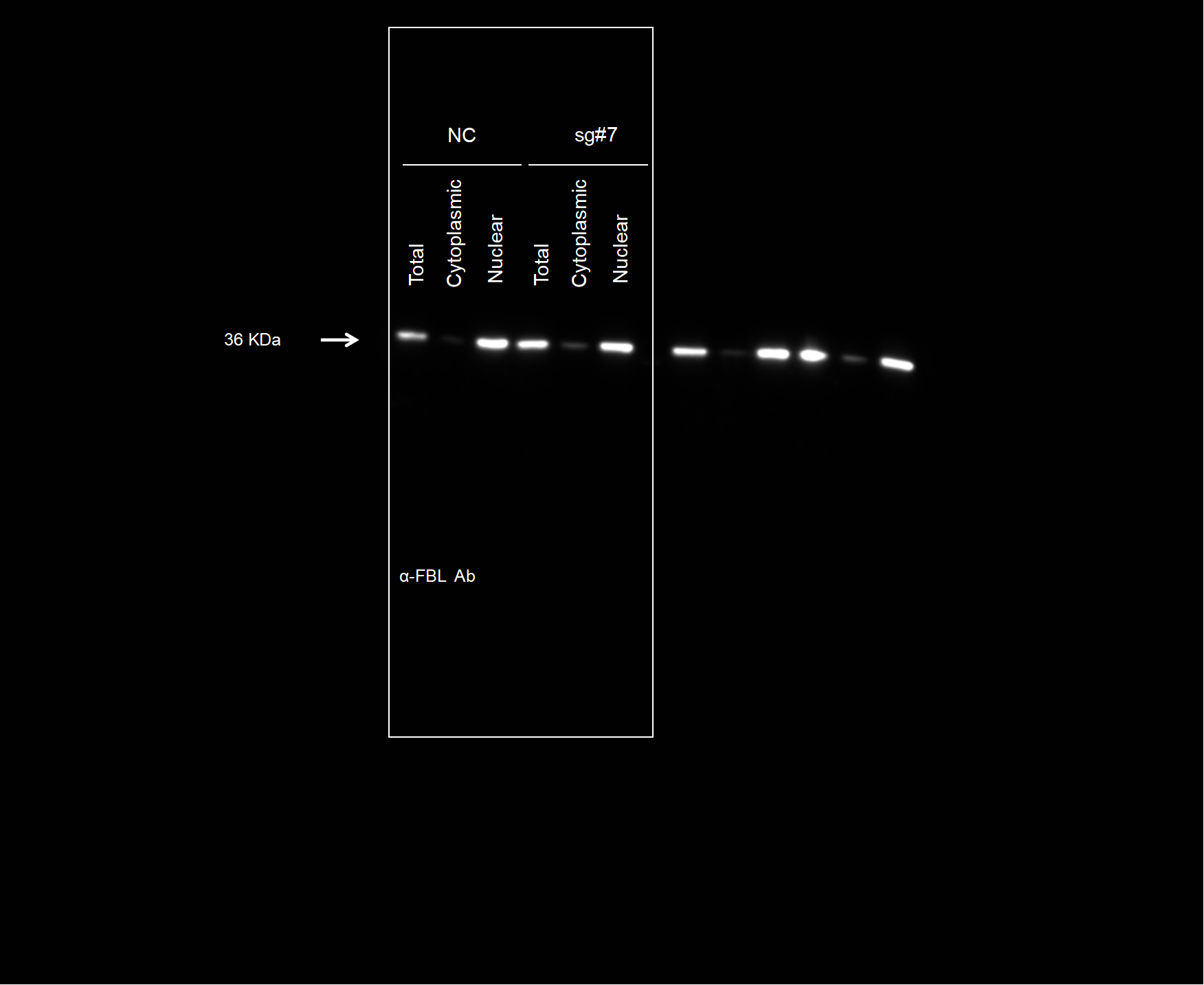

Supplement: Figure 1—figure supplement 1—source data 2. [file elife-79116-fig1-figsupp1-data2.zip › Figure 1-figure supplement 1-source data 2/+Label/Fig 1-Fig supplement 1B-FBL (KO sg#7), Left.Tif.tif]

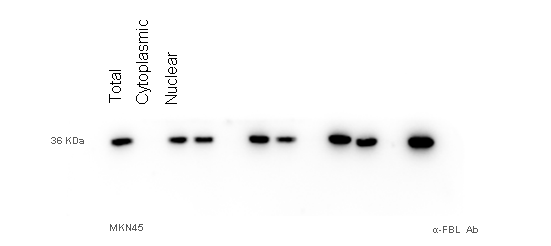

Supplement: Figure 1—figure supplement 1—source data 2. [file elife-79116-fig1-figsupp1-data2.zip › Figure 1-figure supplement 1-source data 2/+Label/Fig 1-Fig supplement 1B-FBL (MKN-45), Left.tif]

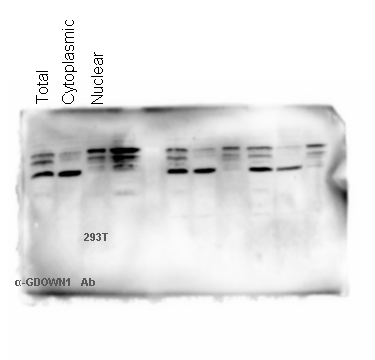

Supplement: Figure 1—figure supplement 1—source data 2. [file elife-79116-fig1-figsupp1-data2.zip › Figure 1-figure supplement 1-source data 2/+Label/Fig 1-Fig supplement 1B-GDOWN1 (293T), Left.tif]

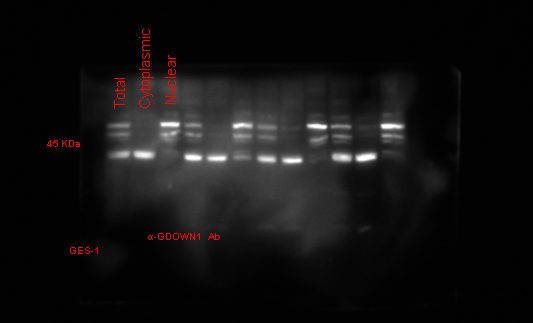

Supplement: Figure 1—figure supplement 1—source data 2. [file elife-79116-fig1-figsupp1-data2.zip › Figure 1-figure supplement 1-source data 2/+Label/Fig 1-Fig supplement 1B-GDOWN1 (GES-1), Left.tif]

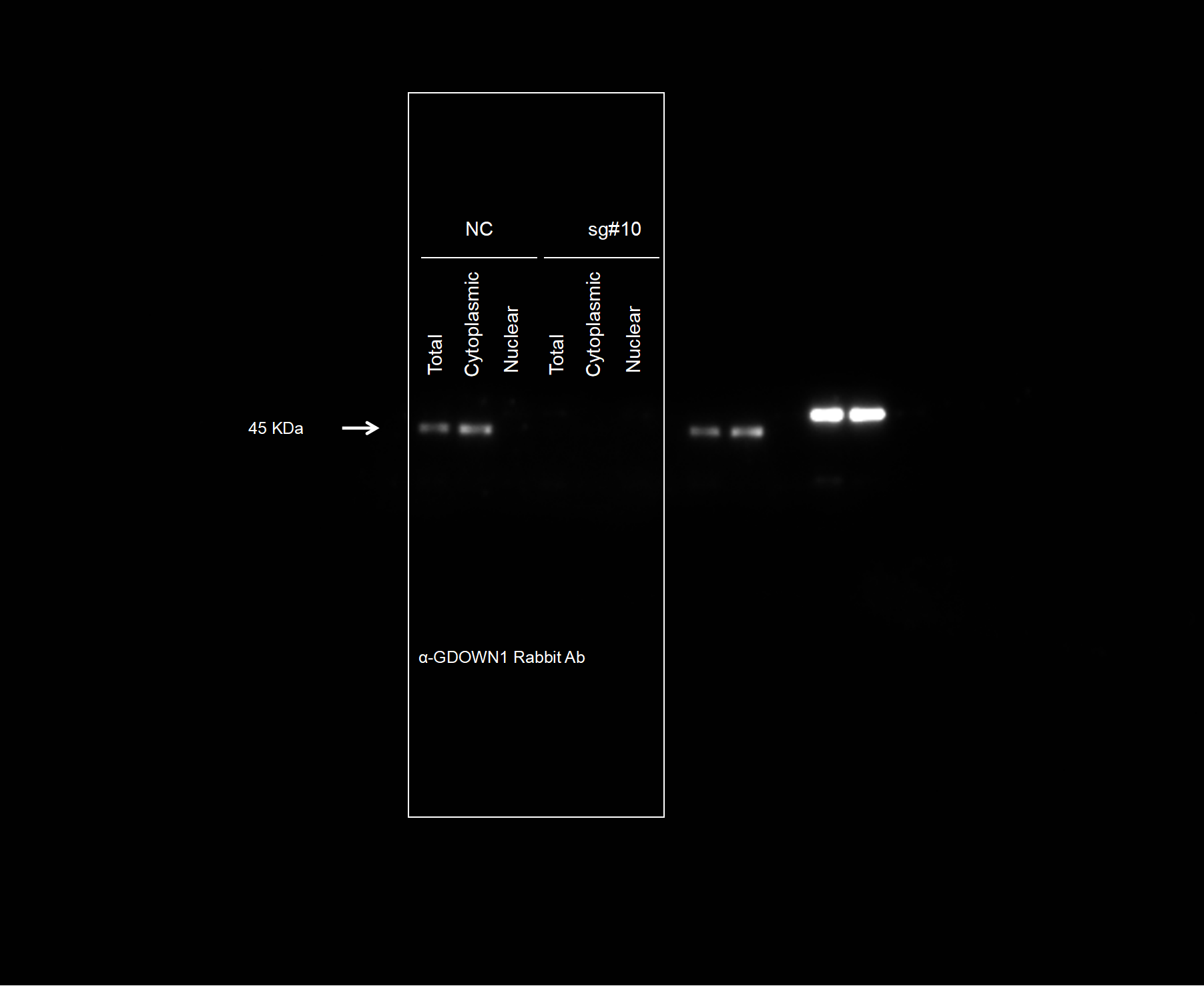

Supplement: Figure 1—figure supplement 1—source data 2. [file elife-79116-fig1-figsupp1-data2.zip › Figure 1-figure supplement 1-source data 2/+Label/Fig 1-Fig supplement 1B-GDOWN1 (KO sg#10), Left.Tif.tif]

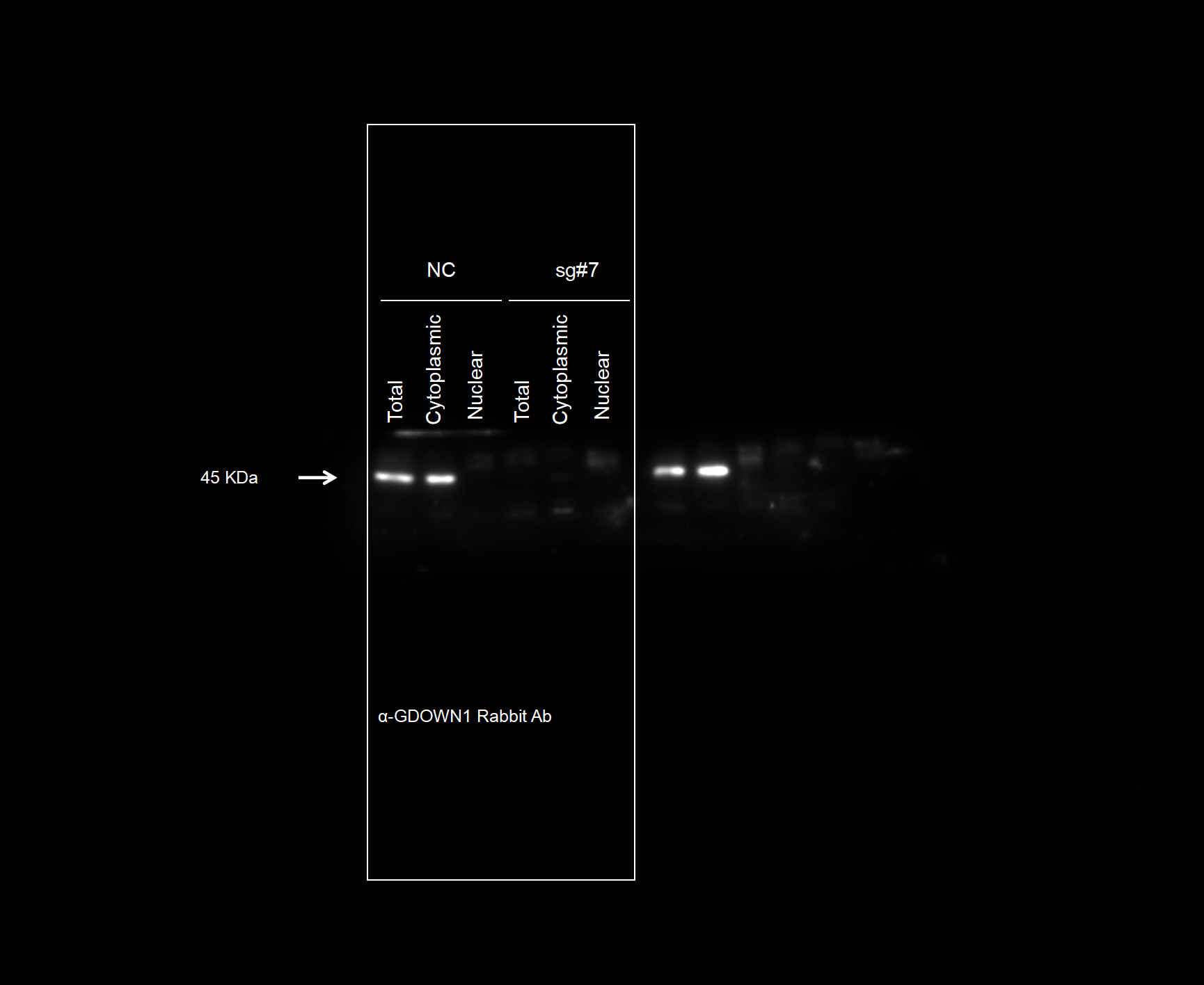

Supplement: Figure 1—figure supplement 1—source data 2. [file elife-79116-fig1-figsupp1-data2.zip › Figure 1-figure supplement 1-source data 2/+Label/Fig 1-Fig supplement 1B-GDOWN1 (KO sg#7), Left.Tif.tif]

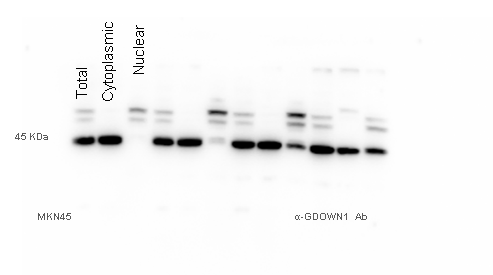

Supplement: Figure 1—figure supplement 1—source data 2. [file elife-79116-fig1-figsupp1-data2.zip › Figure 1-figure supplement 1-source data 2/+Label/Fig 1-Fig supplement 1B-GDOWN1 (MKN-45), Left.tif]

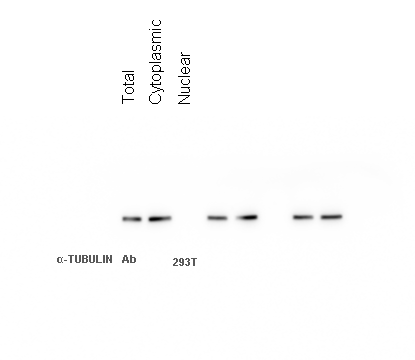

Supplement: Figure 1—figure supplement 1—source data 2. [file elife-79116-fig1-figsupp1-data2.zip › Figure 1-figure supplement 1-source data 2/+Label/Fig 1-Fig supplement 1B-TUBULIN (293T), Left.tif]

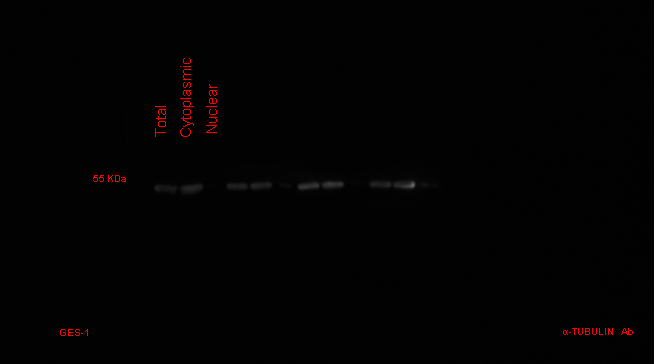

Supplement: Figure 1—figure supplement 1—source data 2. [file elife-79116-fig1-figsupp1-data2.zip › Figure 1-figure supplement 1-source data 2/+Label/Fig 1-Fig supplement 1B-TUBULIN (GES-1), Left.tif]

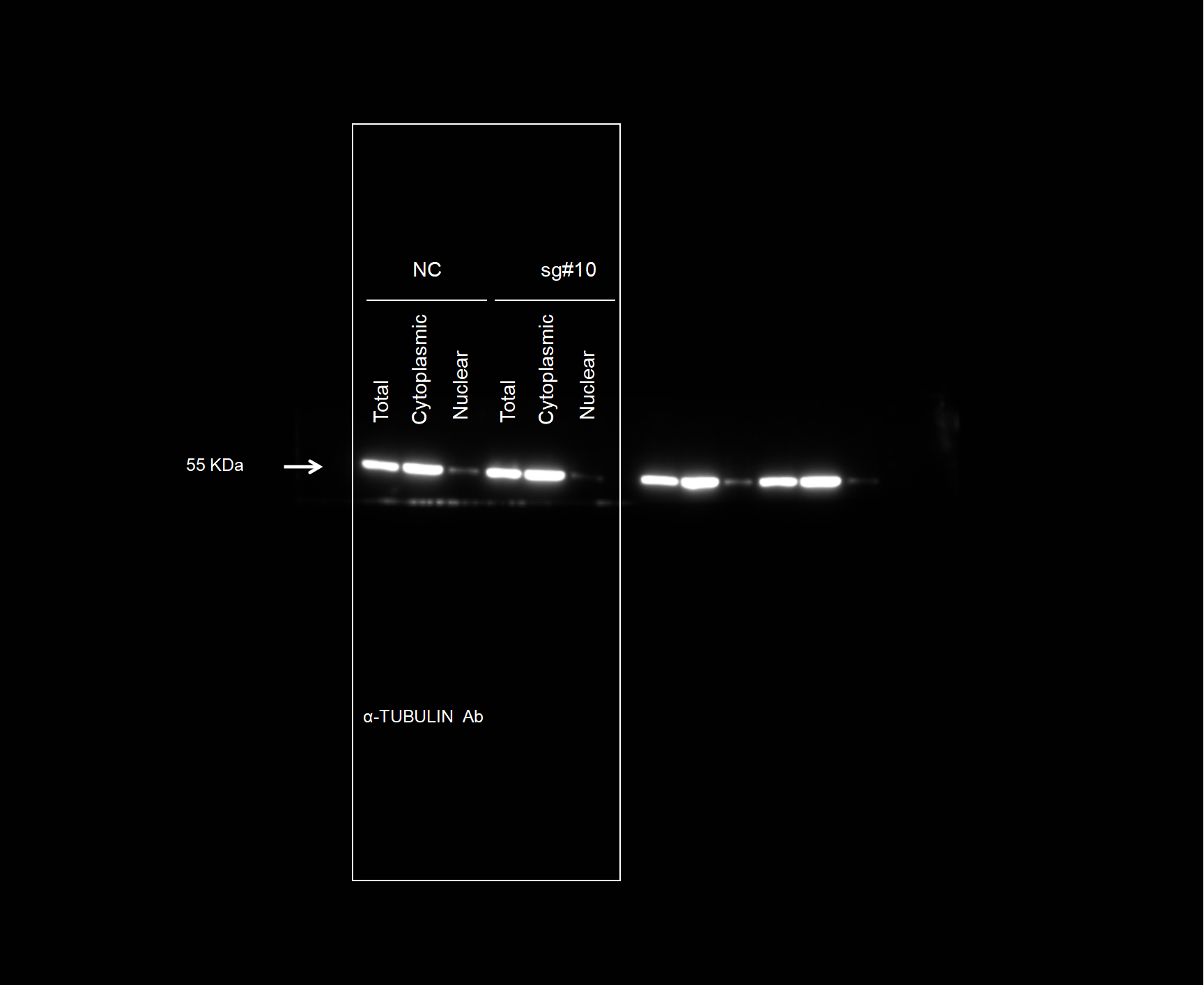

Supplement: Figure 1—figure supplement 1—source data 2. [file elife-79116-fig1-figsupp1-data2.zip › Figure 1-figure supplement 1-source data 2/+Label/Fig 1-Fig supplement 1B-TUBULIN (KO sg#10), Left.Tif.tif]

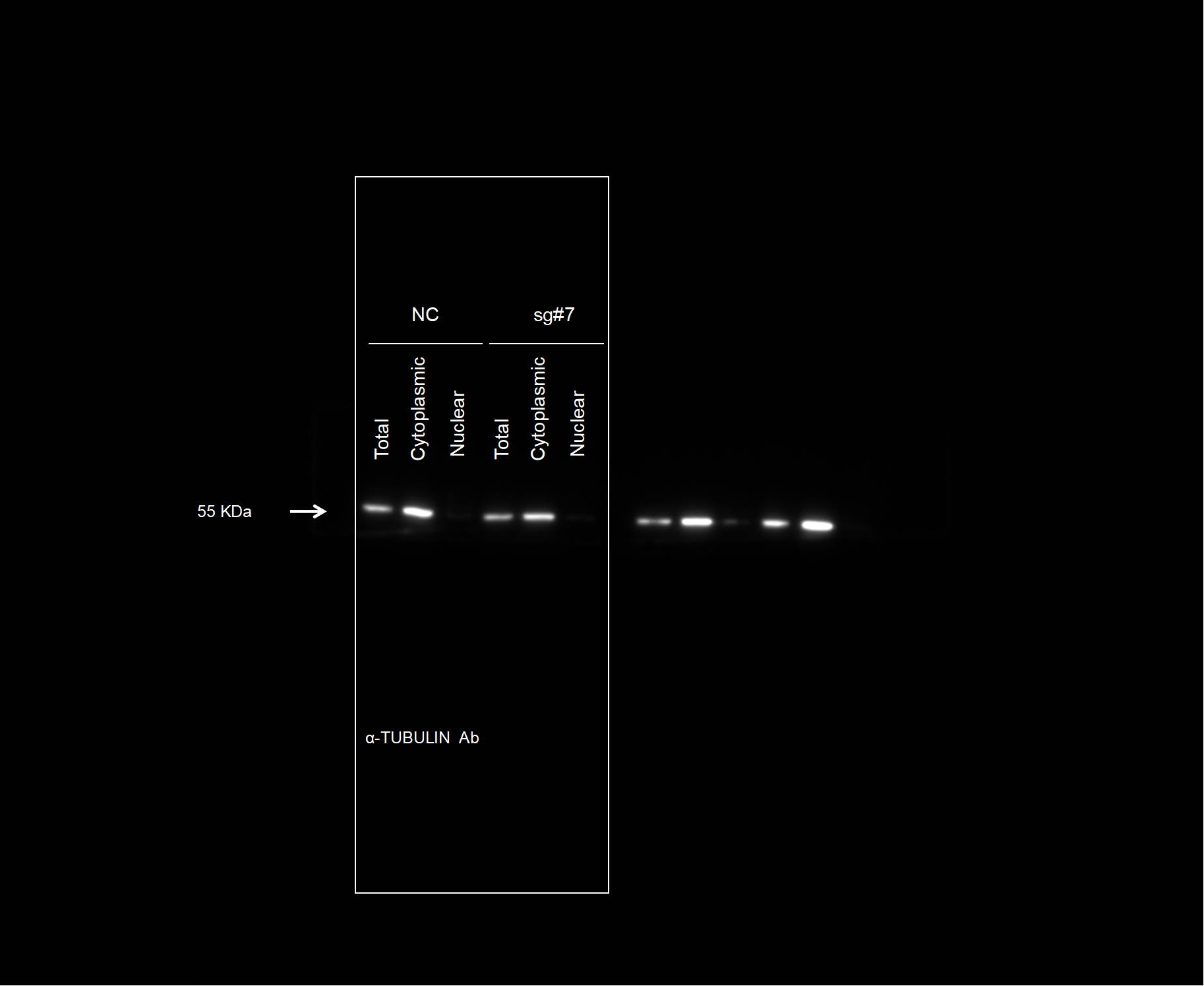

Supplement: Figure 1—figure supplement 1—source data 2. [file elife-79116-fig1-figsupp1-data2.zip › Figure 1-figure supplement 1-source data 2/+Label/Fig 1-Fig supplement 1B-TUBULIN (KO sg#7), Left.Tif.tif]

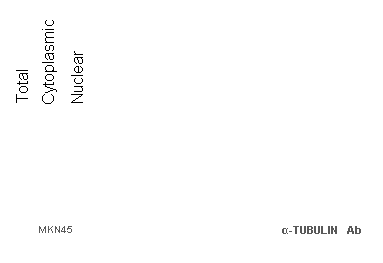

Supplement: Figure 1—figure supplement 1—source data 2. [file elife-79116-fig1-figsupp1-data2.zip › Figure 1-figure supplement 1-source data 2/+Label/Fig 1-Fig supplement 1B-TUBULIN (MKN-45), Left.tif]

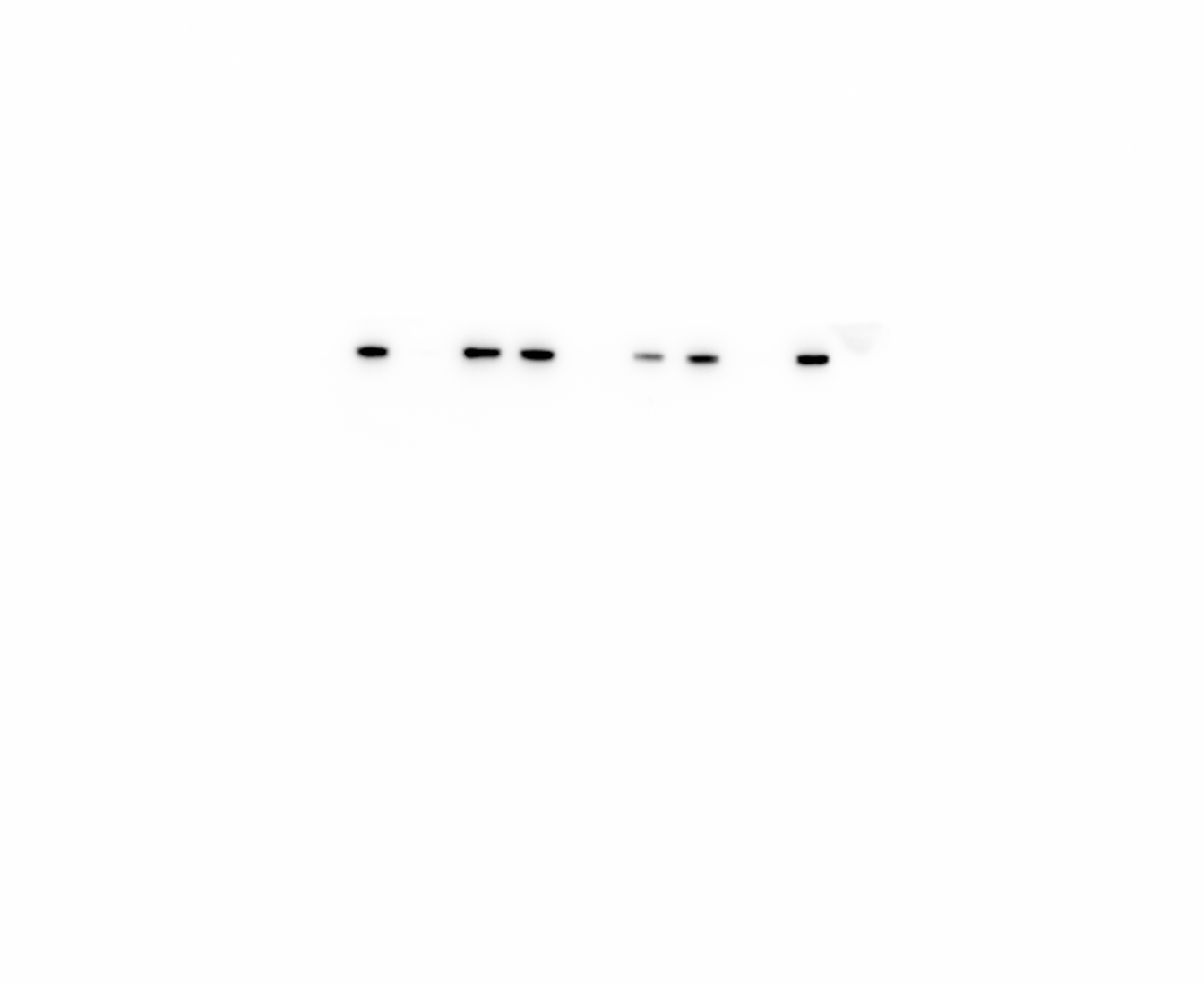

Supplement: Figure 1—figure supplement 1—source data 2. [file elife-79116-fig1-figsupp1-data2.zip › Figure 1-figure supplement 1-source data 2/unedited/Fig 1-Fig supplement 1B-FBL (293T), Left.Tif]

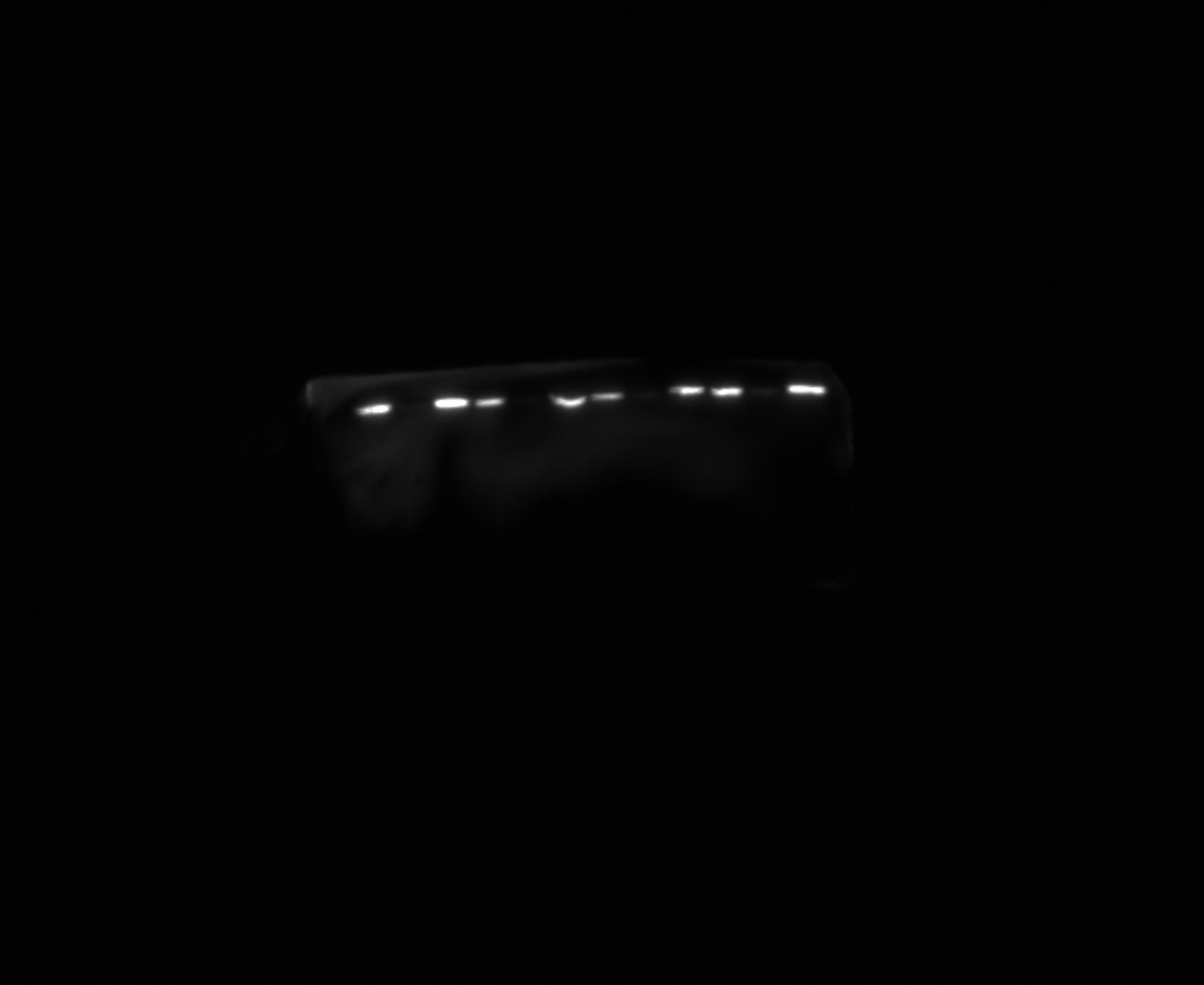

Supplement: Figure 1—figure supplement 1—source data 2. [file elife-79116-fig1-figsupp1-data2.zip › Figure 1-figure supplement 1-source data 2/unedited/Fig 1-Fig supplement 1B-FBL (GES-1), Left.Tif]

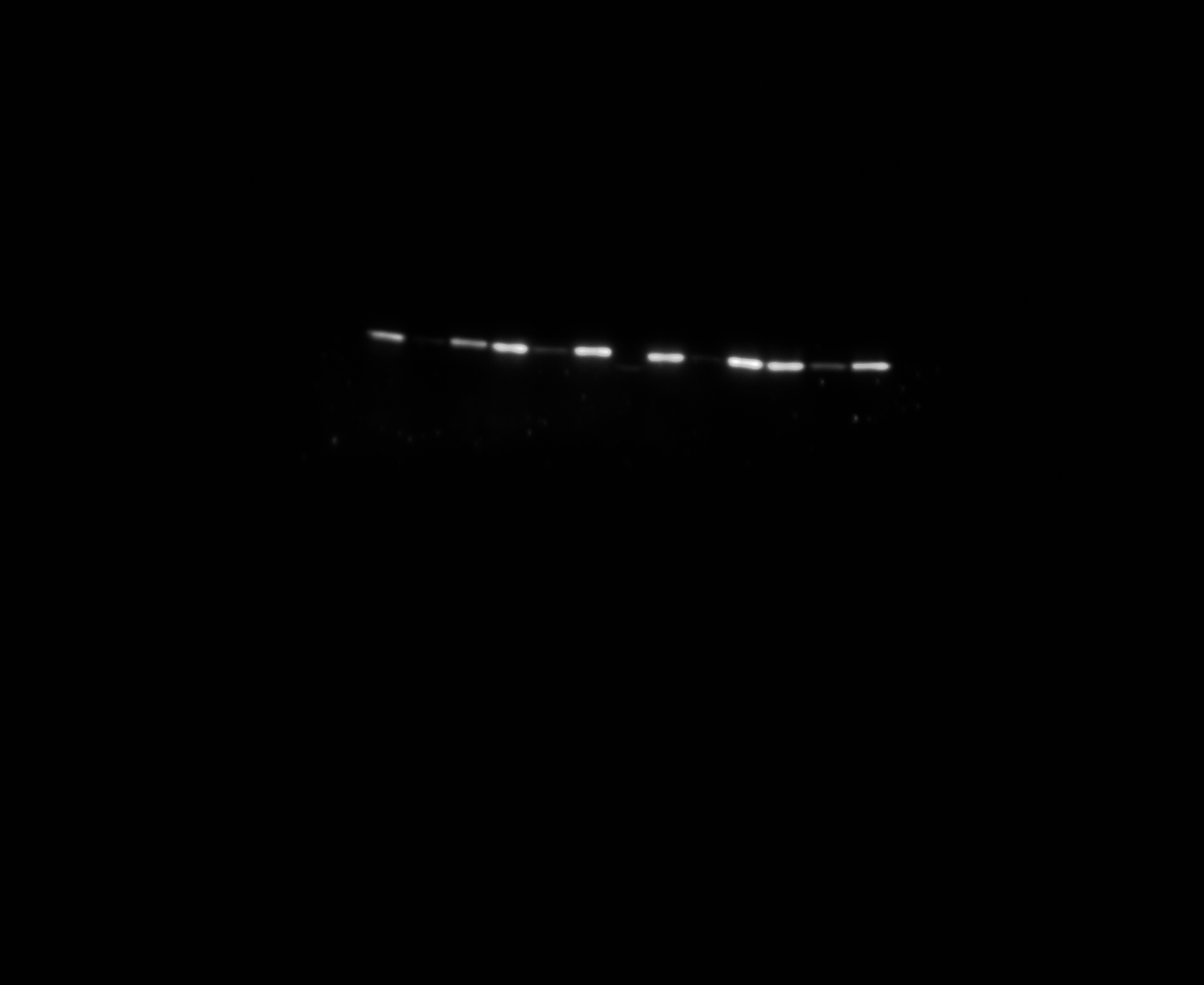

Supplement: Figure 1—figure supplement 1—source data 2. [file elife-79116-fig1-figsupp1-data2.zip › Figure 1-figure supplement 1-source data 2/unedited/Fig 1-Fig supplement 1B-FBL (KO sg#10), Left.Tif]

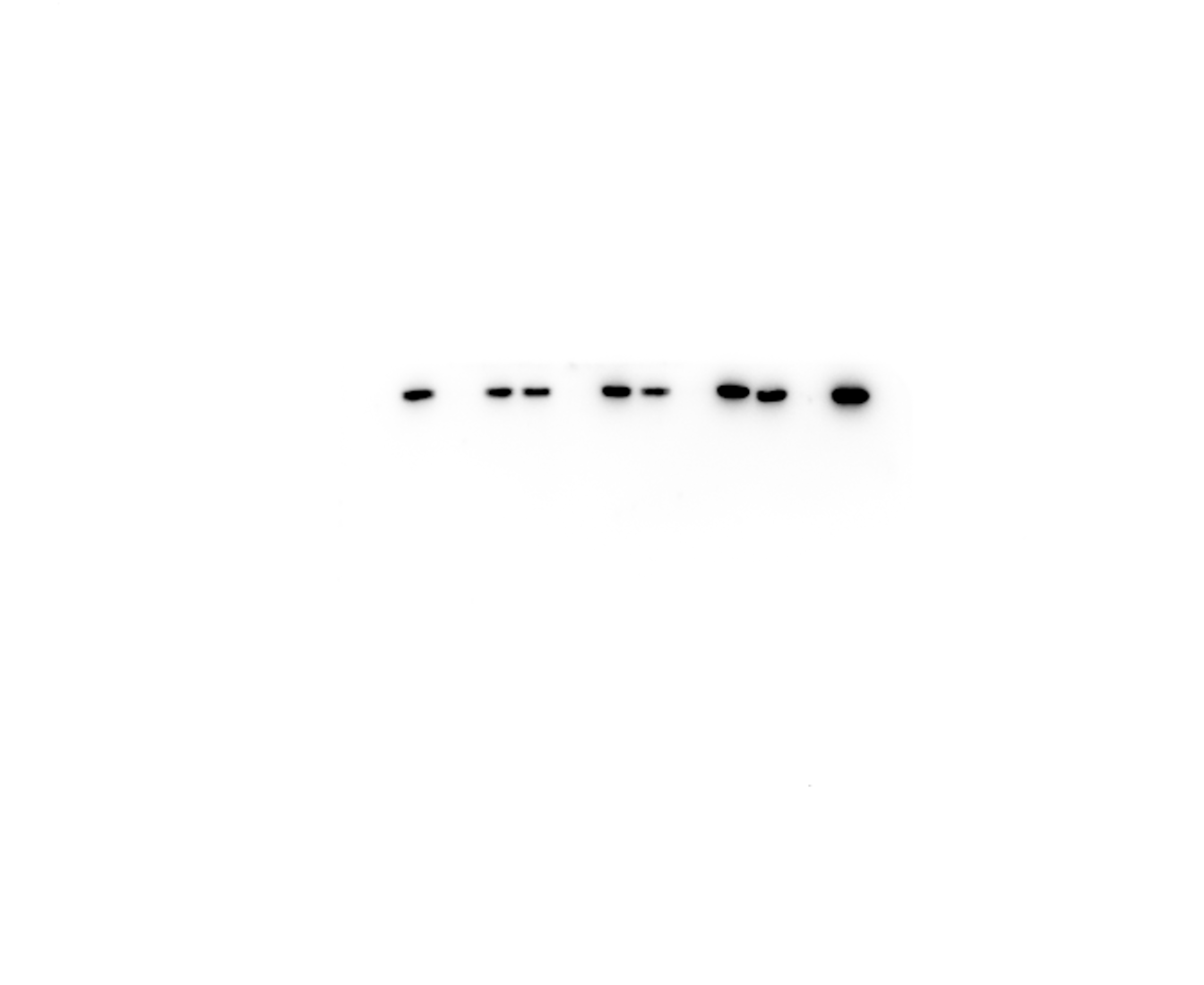

Supplement: Figure 1—figure supplement 1—source data 2. [file elife-79116-fig1-figsupp1-data2.zip › Figure 1-figure supplement 1-source data 2/unedited/Fig 1-Fig supplement 1B-FBL (MKN-45), Left.Tif]

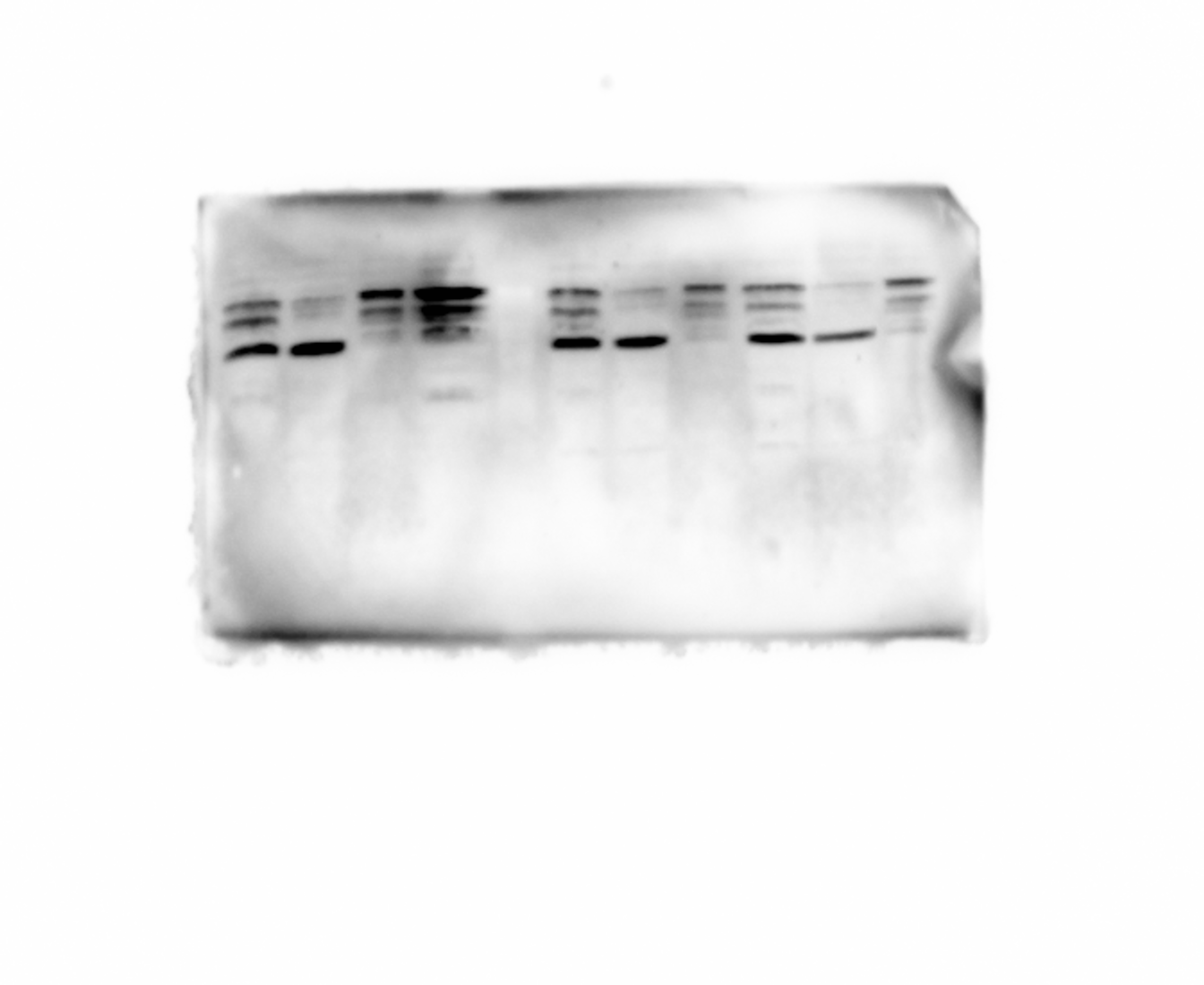

Supplement: Figure 1—figure supplement 1—source data 2. [file elife-79116-fig1-figsupp1-data2.zip › Figure 1-figure supplement 1-source data 2/unedited/Fig 1-Fig supplement 1B-GDOWN1 (293T), Left.Tif]

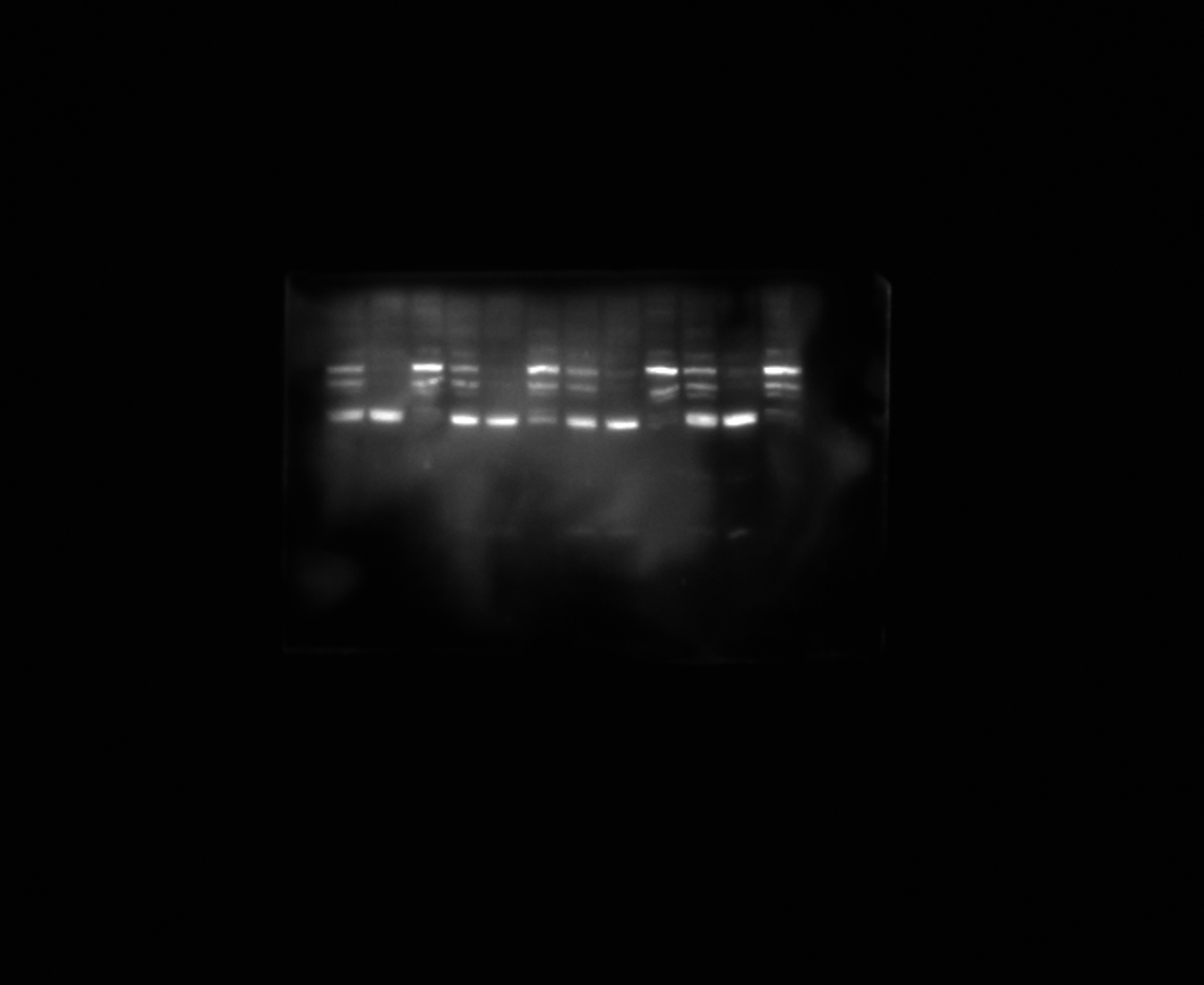

Supplement: Figure 1—figure supplement 1—source data 2. [file elife-79116-fig1-figsupp1-data2.zip › Figure 1-figure supplement 1-source data 2/unedited/Fig 1-Fig supplement 1B-GDOWN1 (GES-1), Left.Tif]

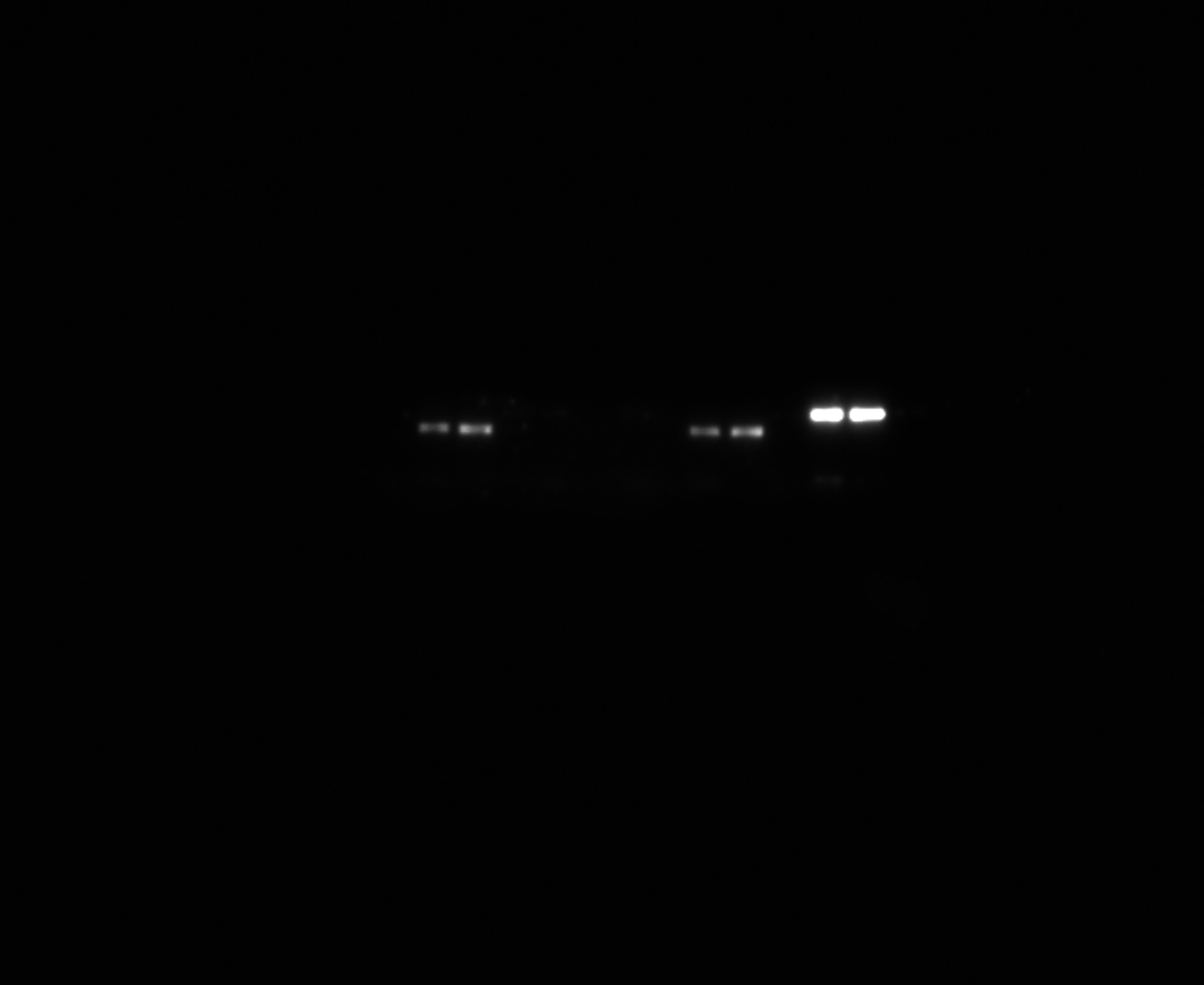

Supplement: Figure 1—figure supplement 1—source data 2. [file elife-79116-fig1-figsupp1-data2.zip › Figure 1-figure supplement 1-source data 2/unedited/Fig 1-Fig supplement 1B-GDOWN1 (KO sg#10), Left.Tif]

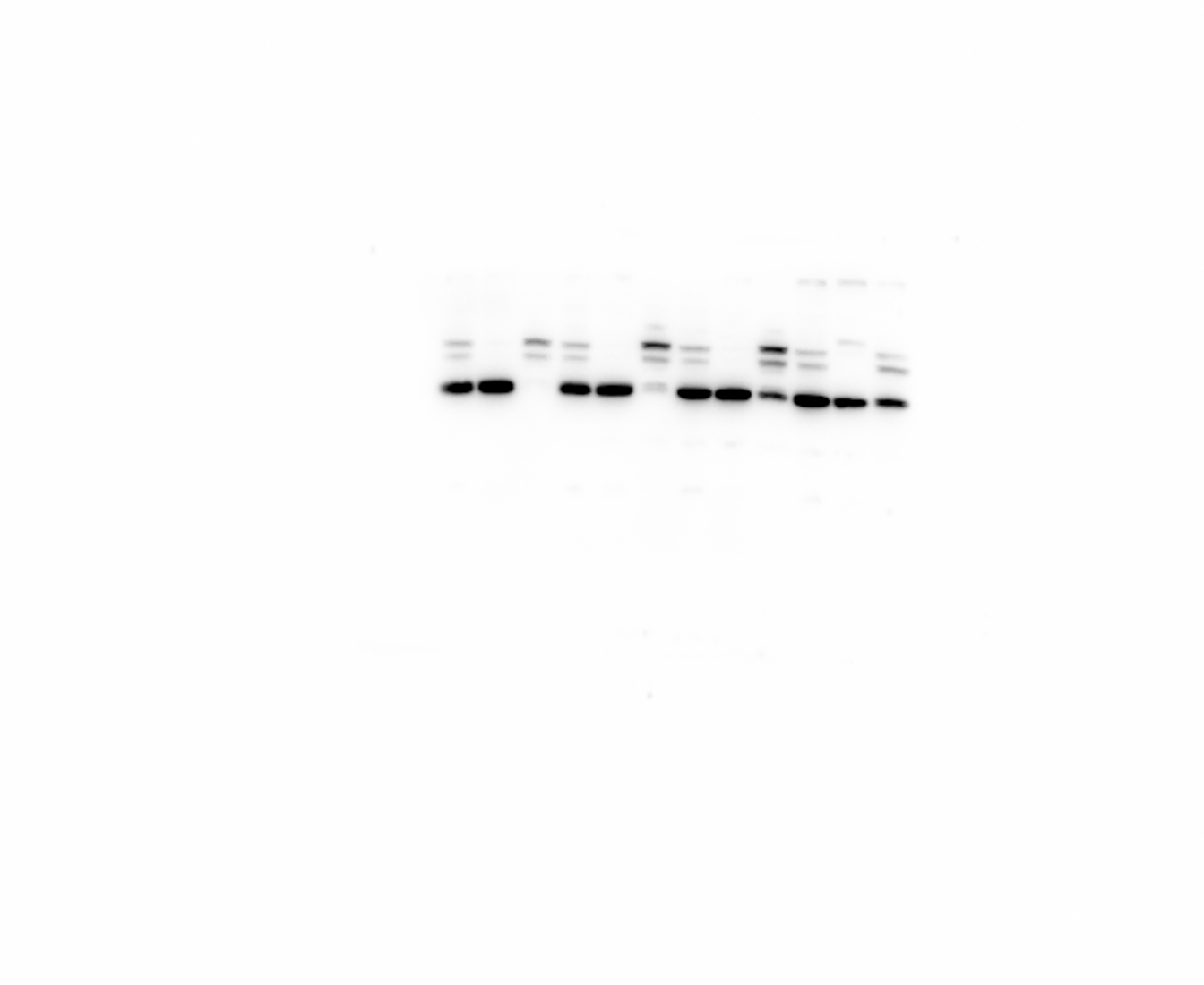

Supplement: Figure 1—figure supplement 1—source data 2. [file elife-79116-fig1-figsupp1-data2.zip › Figure 1-figure supplement 1-source data 2/unedited/Fig 1-Fig supplement 1B-GDOWN1 (MKN-45), Left.Tif]

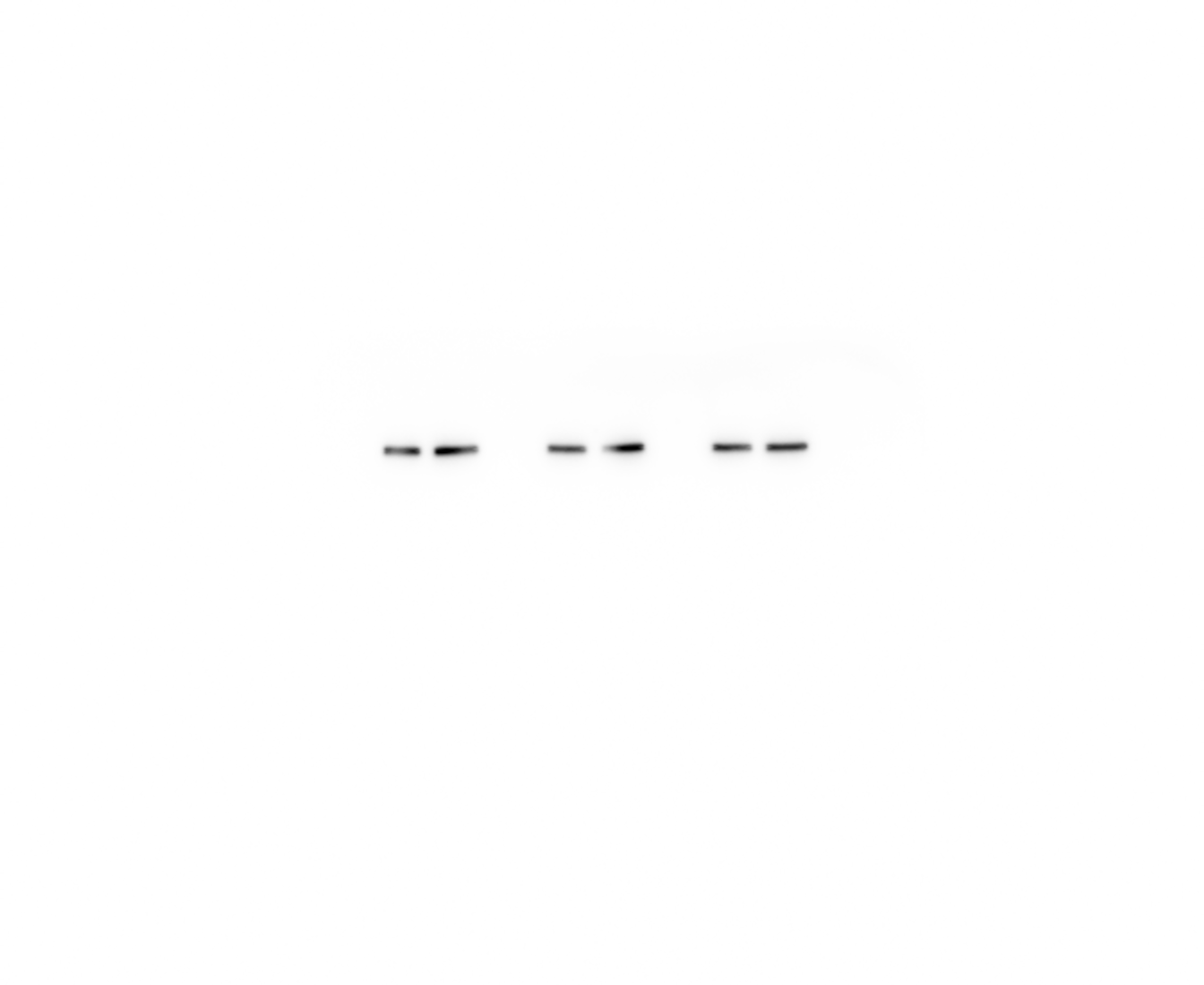

Supplement: Figure 1—figure supplement 1—source data 2. [file elife-79116-fig1-figsupp1-data2.zip › Figure 1-figure supplement 1-source data 2/unedited/Fig 1-Fig supplement 1B-TUBULIN (293T), Left.Tif]

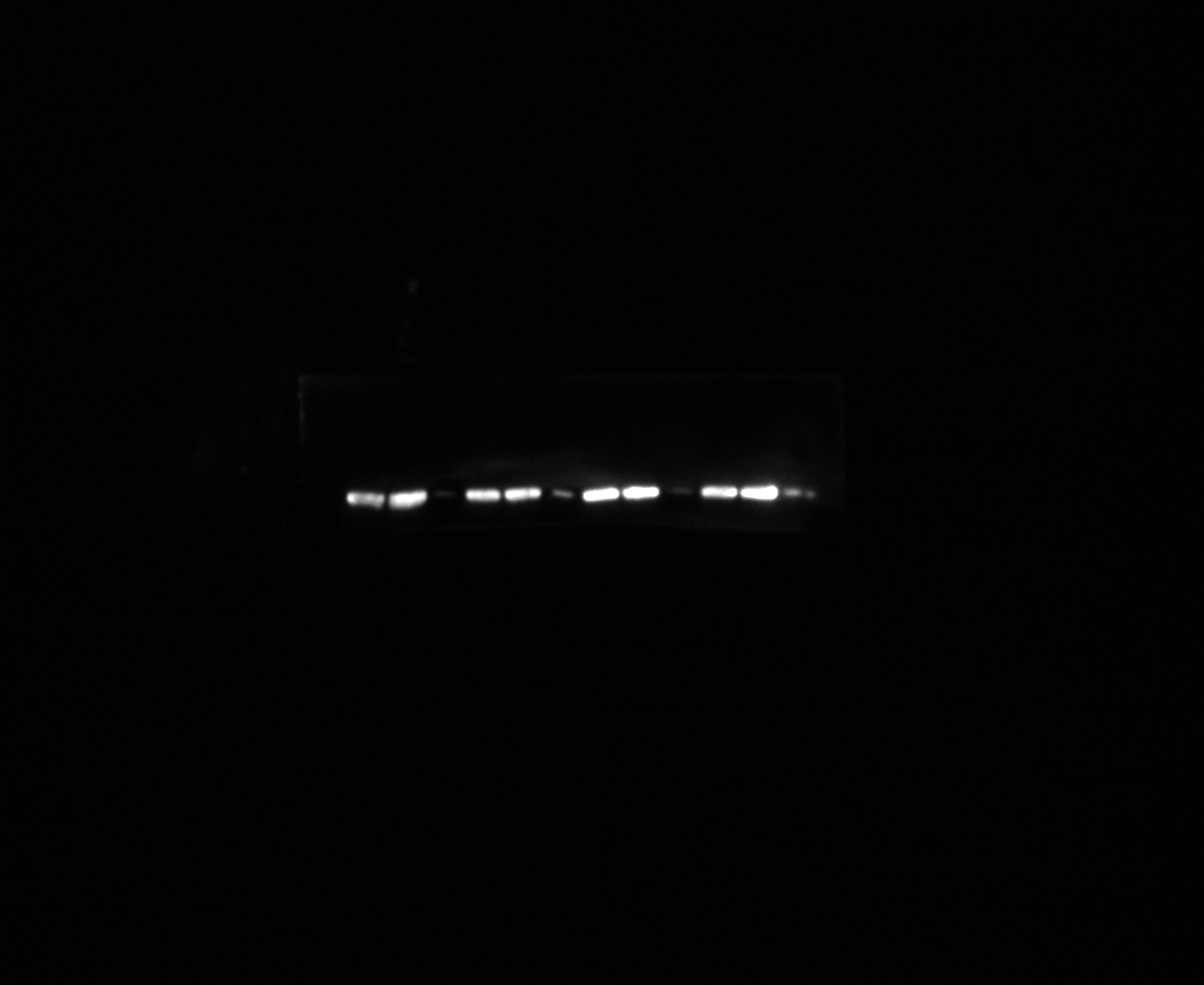

Supplement: Figure 1—figure supplement 1—source data 2. [file elife-79116-fig1-figsupp1-data2.zip › Figure 1-figure supplement 1-source data 2/unedited/Fig 1-Fig supplement 1B-TUBULIN (GES-1), Left.Tif]

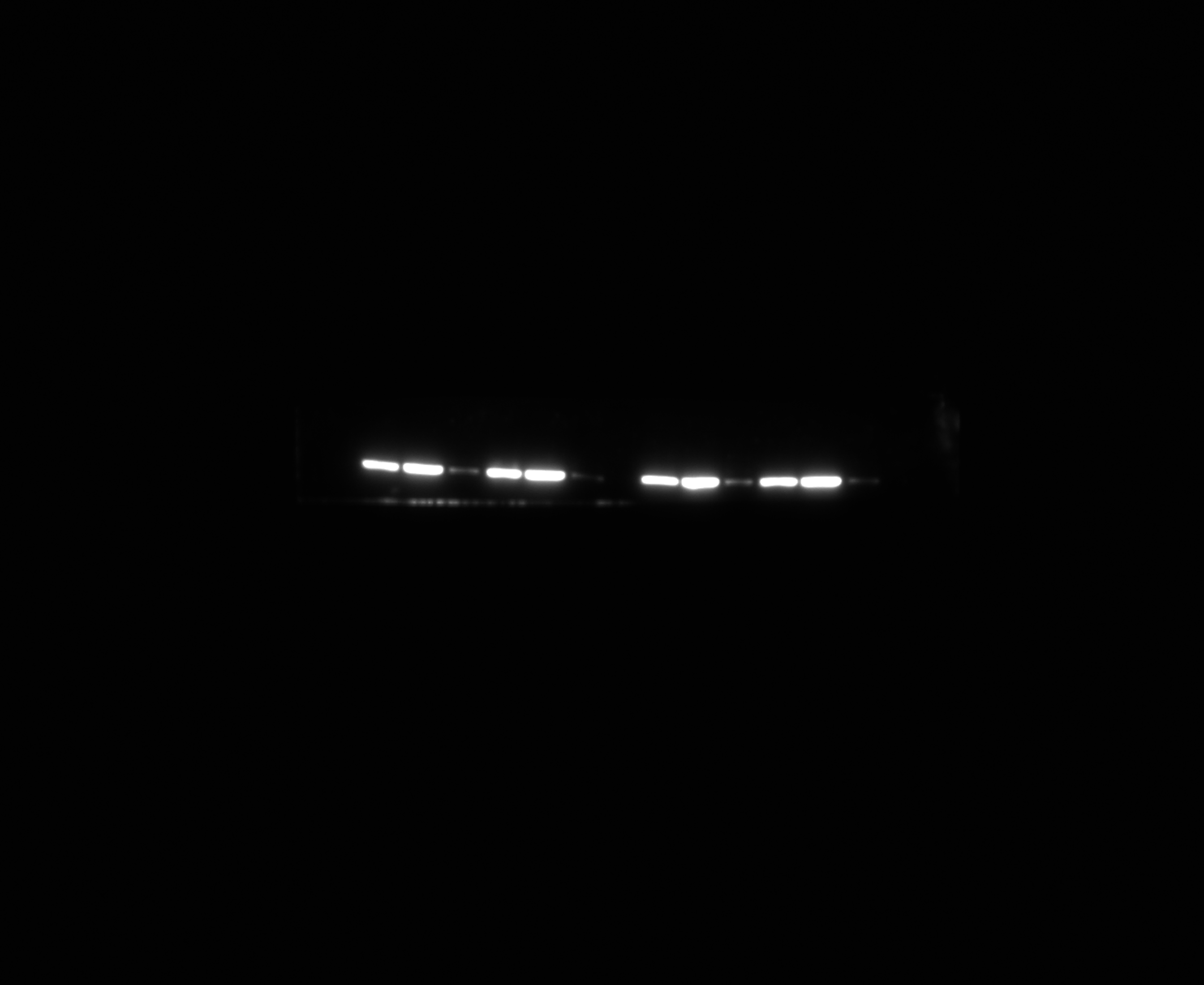

Supplement: Figure 1—figure supplement 1—source data 2. [file elife-79116-fig1-figsupp1-data2.zip › Figure 1-figure supplement 1-source data 2/unedited/Fig 1-Fig supplement 1B-TUBULIN (KO sg#10), Left.Tif]

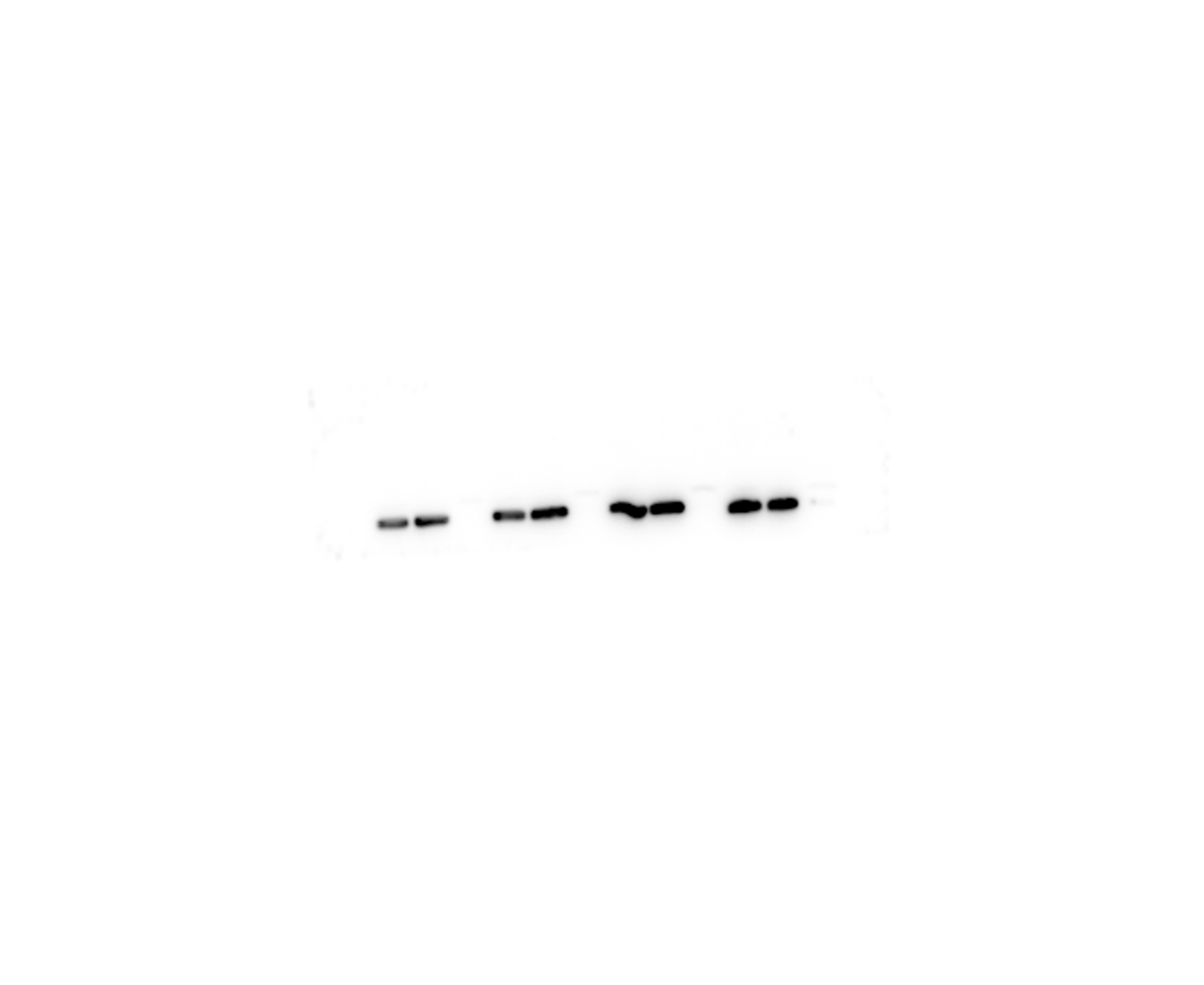

Supplement: Figure 1—figure supplement 1—source data 2. [file elife-79116-fig1-figsupp1-data2.zip › Figure 1-figure supplement 1-source data 2/unedited/Fig 1-Fig supplement 1B-TUBULIN (MKN-45), Left.Tif]

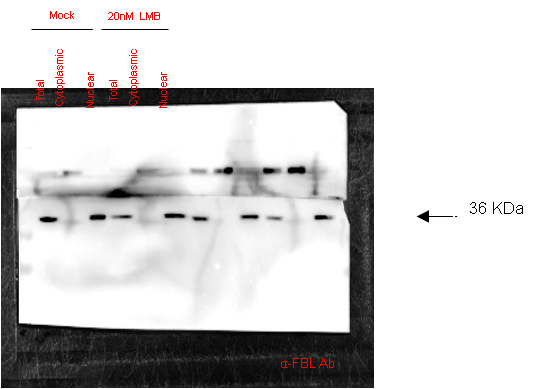

Supplement: Figure 1—figure supplement 1—source data 3. [file elife-79116-fig1-figsupp1-data3.zip › Figure 1-figure supplement 1-source data 3/+Label/Fig1-Fig supplement 1C-FBL antibody(for HeLa mock +LMB).tif]

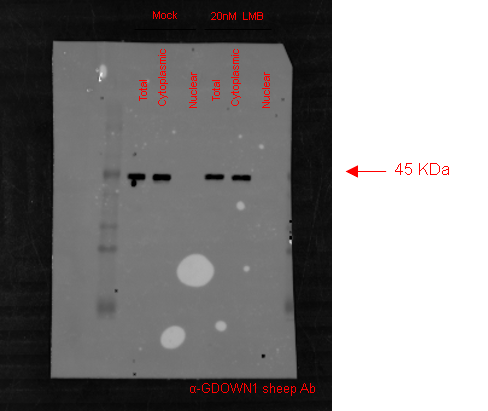

Supplement: Figure 1—figure supplement 1—source data 3. [file elife-79116-fig1-figsupp1-data3.zip › Figure 1-figure supplement 1-source data 3/+Label/Fig1-Fig supplement 1C-GDOWN1-sheep antibody(for HeLa mock +LMB).tif]

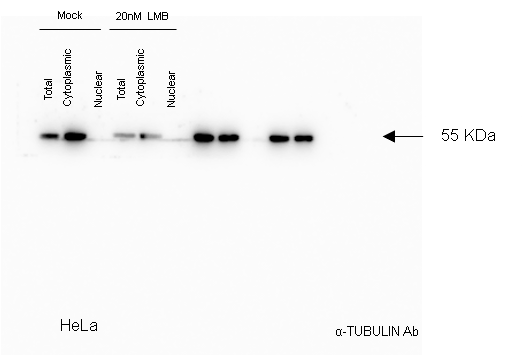

Supplement: Figure 1—figure supplement 1—source data 3. [file elife-79116-fig1-figsupp1-data3.zip › Figure 1-figure supplement 1-source data 3/+Label/Fig1-Fig supplement 1C-TUBULIN antibody (for HeLa mock +LMB).tif]

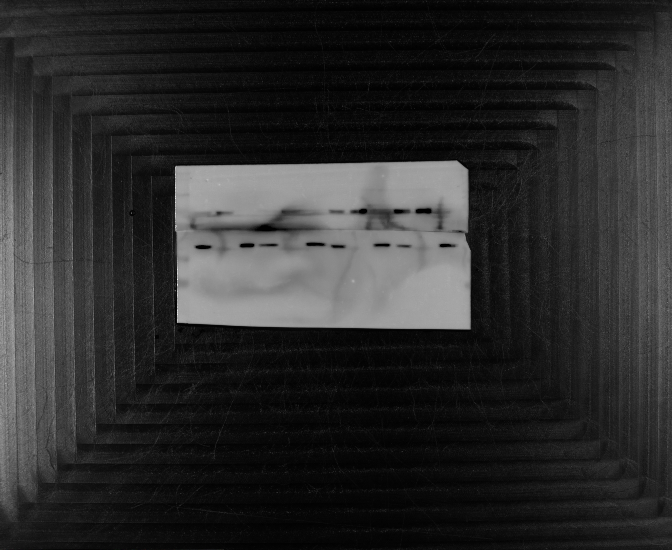

Supplement: Figure 1—figure supplement 1—source data 3. [file elife-79116-fig1-figsupp1-data3.zip › Figure 1-figure supplement 1-source data 3/Unedited/Fig1-Fig supplement 1C-FBL antibody(for HeLa mock +LMB).Tif]

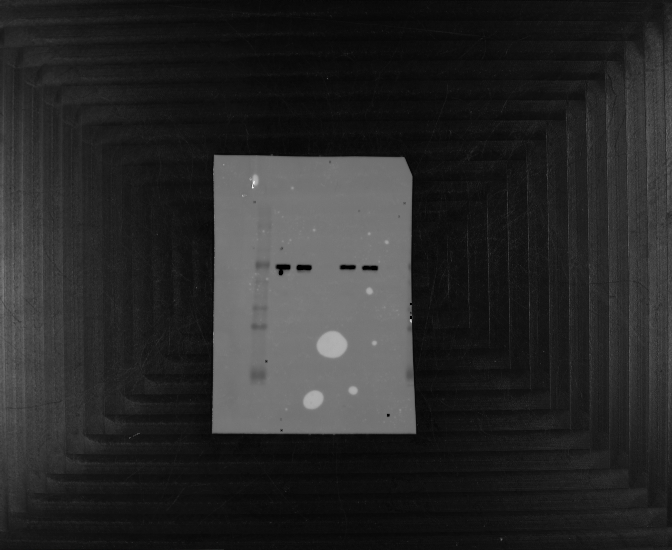

Supplement: Figure 1—figure supplement 1—source data 3. [file elife-79116-fig1-figsupp1-data3.zip › Figure 1-figure supplement 1-source data 3/Unedited/Fig1-Fig supplement 1C-GDOWN1-sheep antibody(for HeLa mock +LMB).Tif]

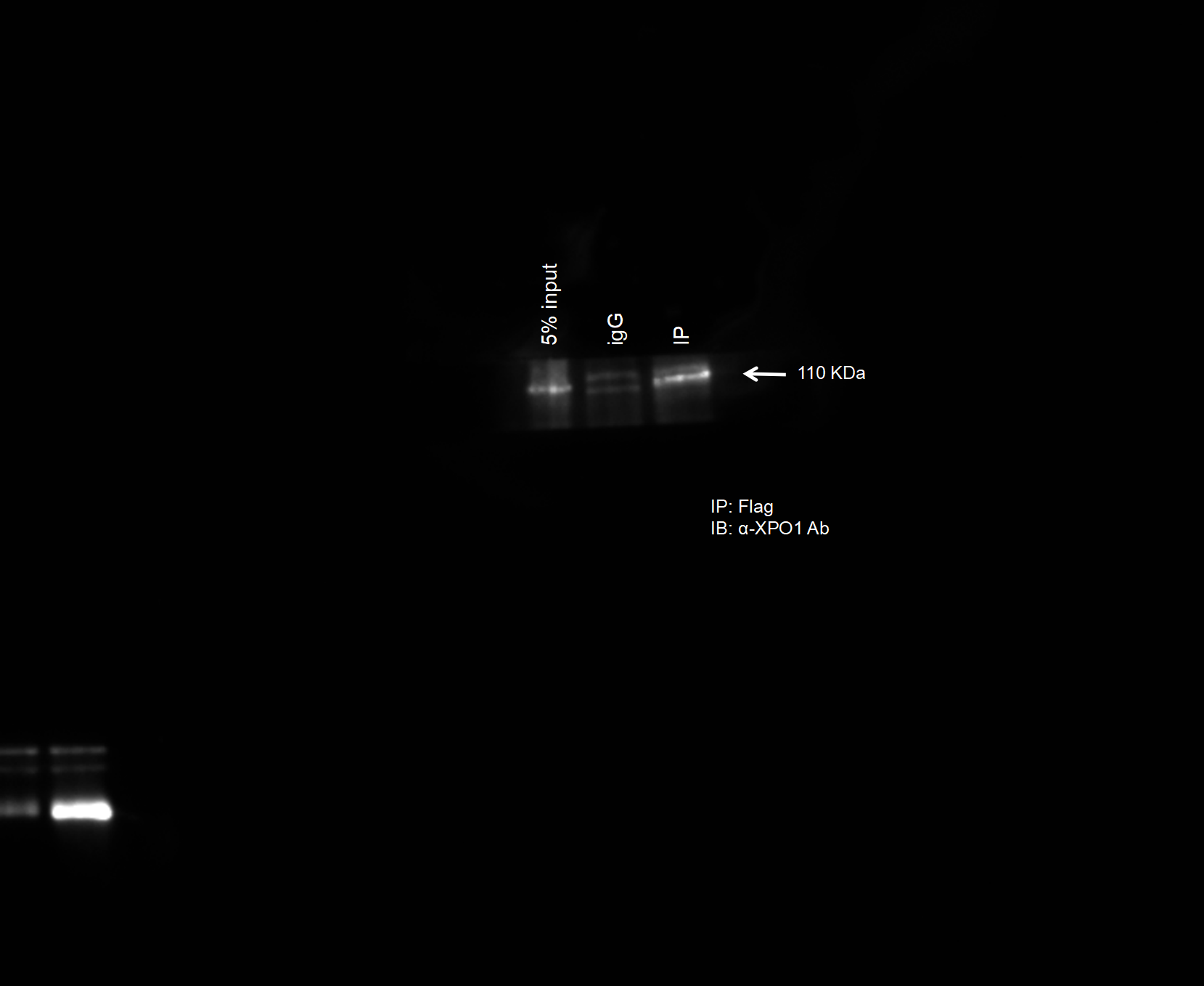

Supplement: Figure 2—source data 2. [file elife-79116-fig2-data2.zip › Figure 2-source data 2/+Label/Fig 2C-CRM1 antibody.tif]

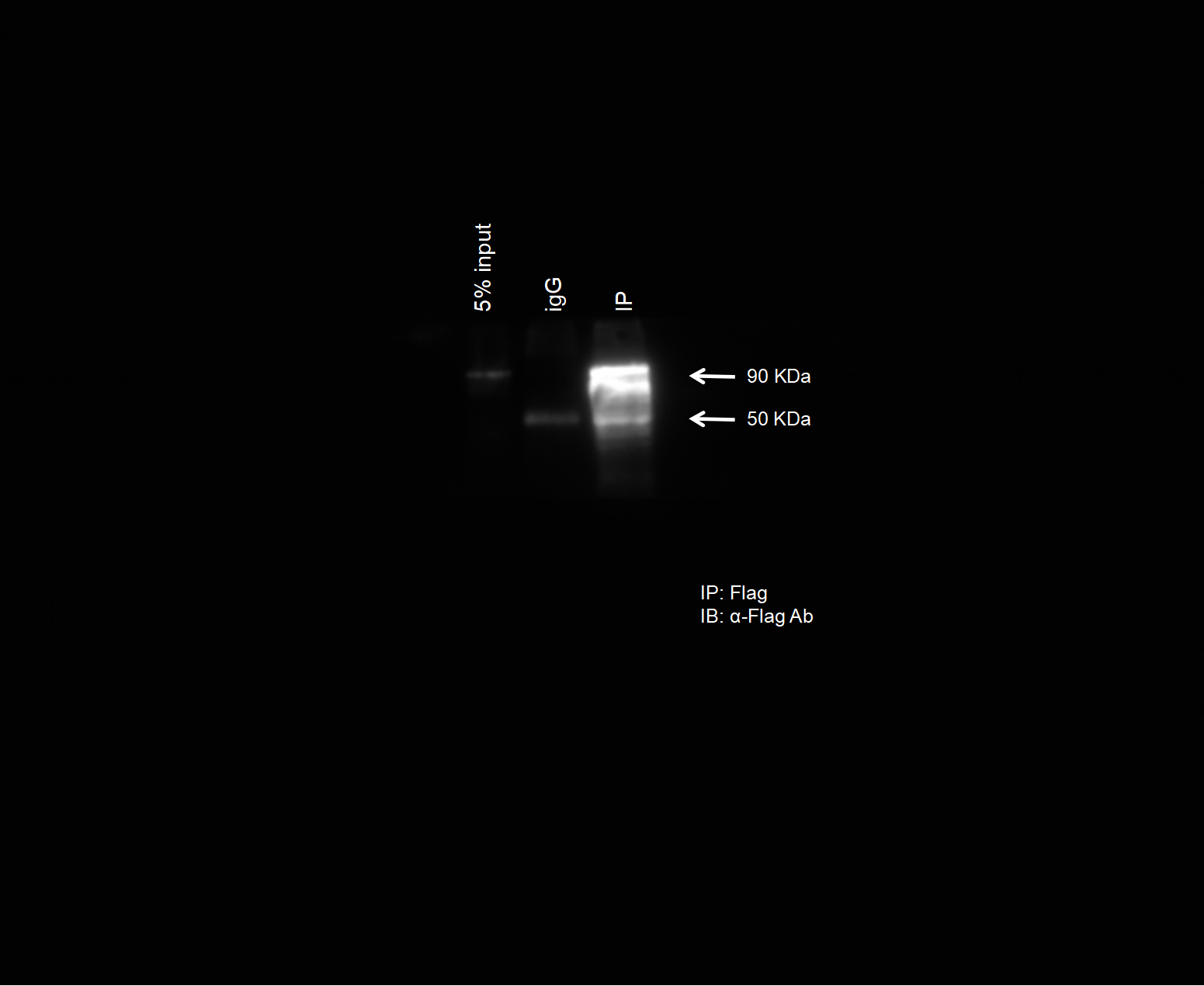

Supplement: Figure 2—source data 2. [file elife-79116-fig2-data2.zip › Figure 2-source data 2/+Label/Fig 2C-Flag antibody.tif]

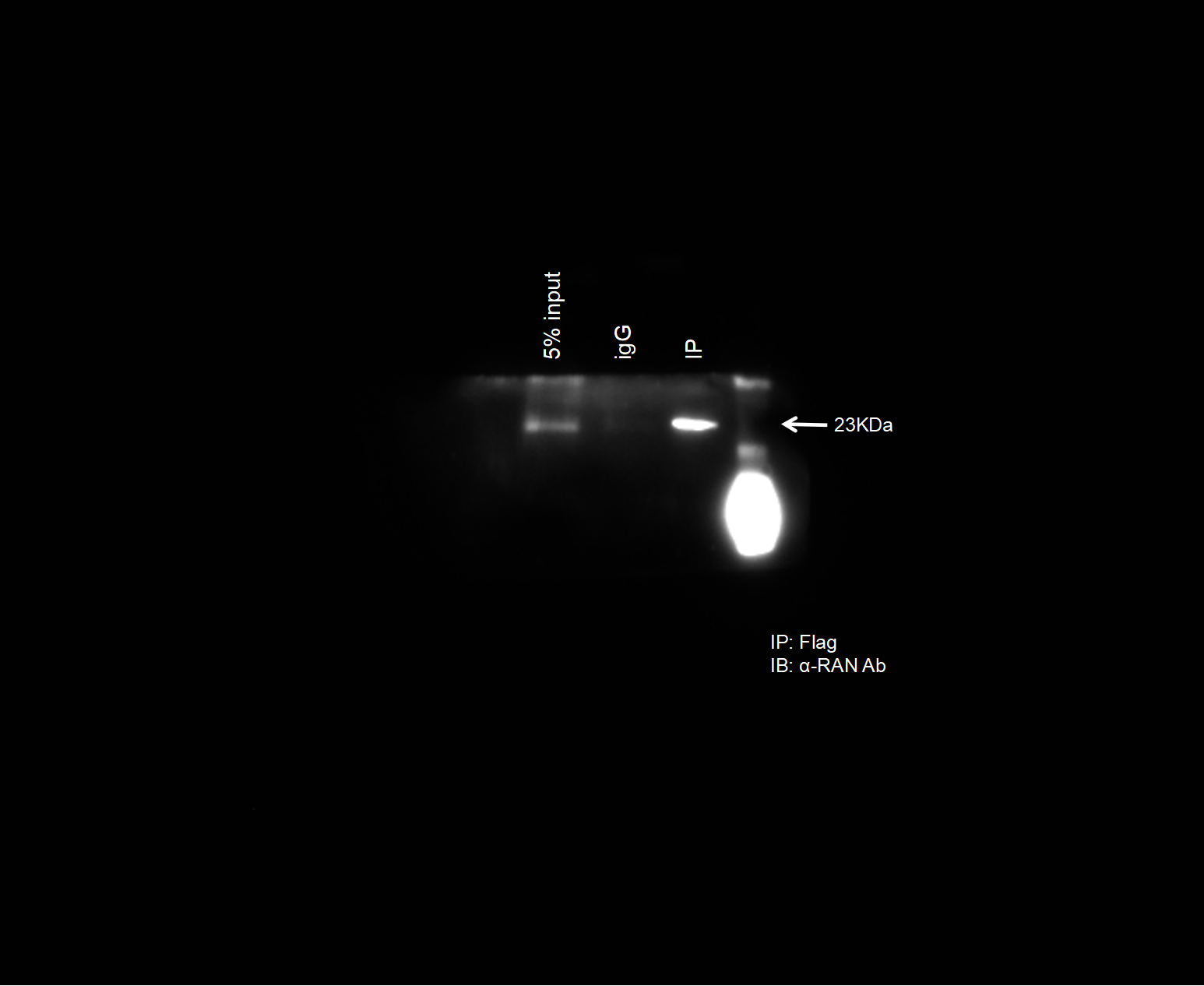

Supplement: Figure 2—source data 2. [file elife-79116-fig2-data2.zip › Figure 2-source data 2/+Label/Fig 2C-RAN antibody.tif]

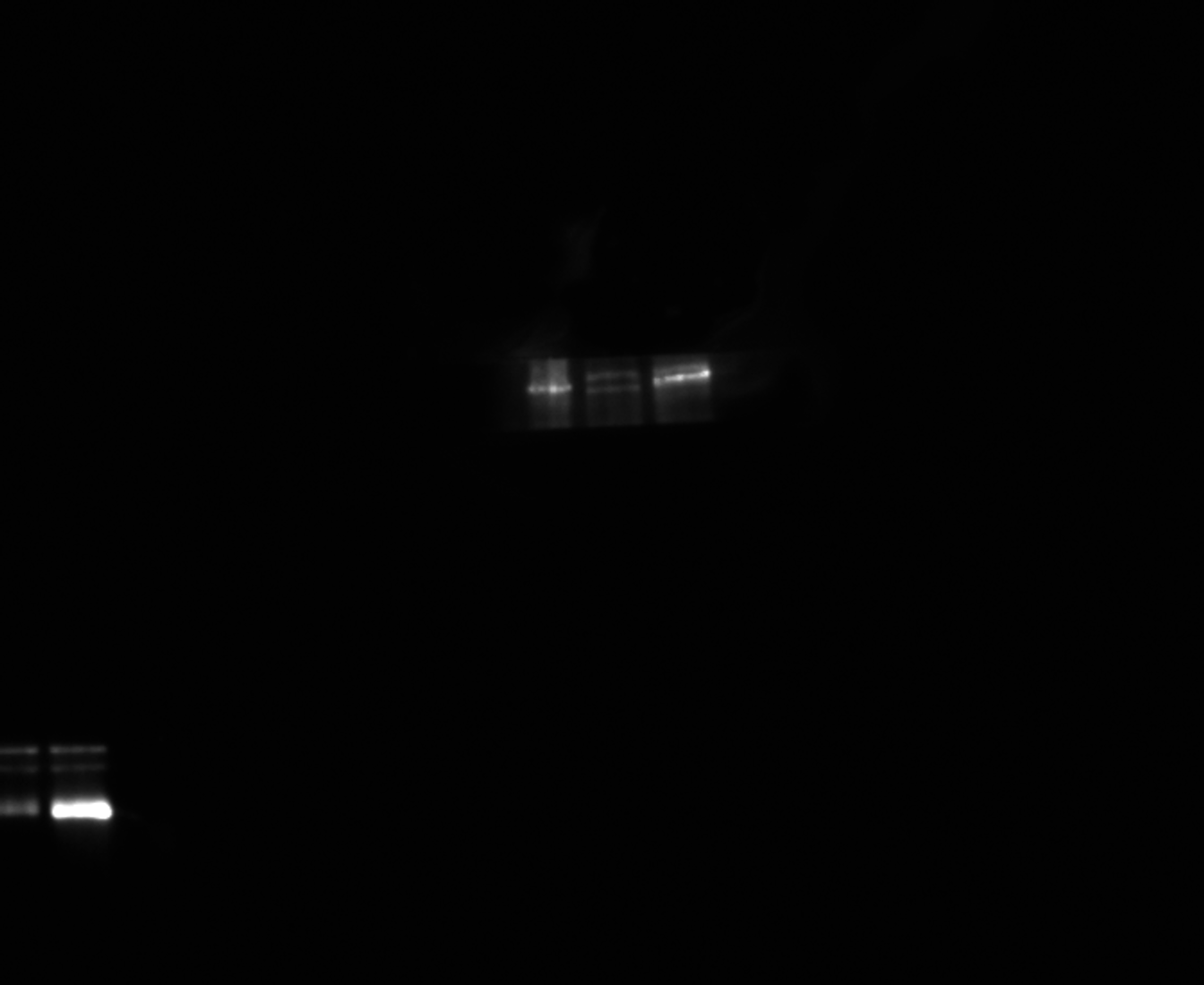

Supplement: Figure 2—source data 2. [file elife-79116-fig2-data2.zip › Figure 2-source data 2/Unedited/Fig 2C-CRM1 antibody.Tif]

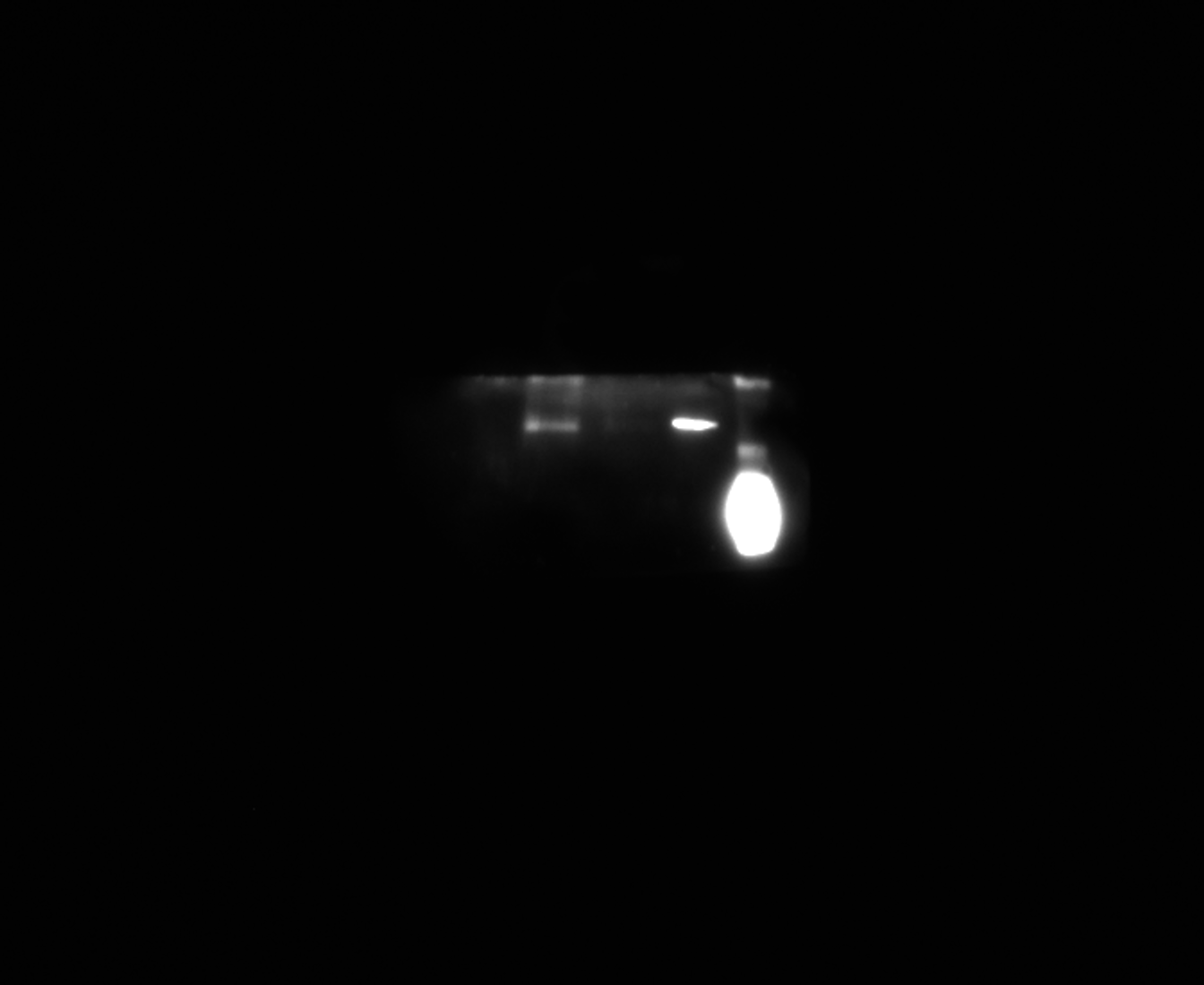

Supplement: Figure 2—source data 2. [file elife-79116-fig2-data2.zip › Figure 2-source data 2/Unedited/Fig 2C-RAN antibody.Tif]

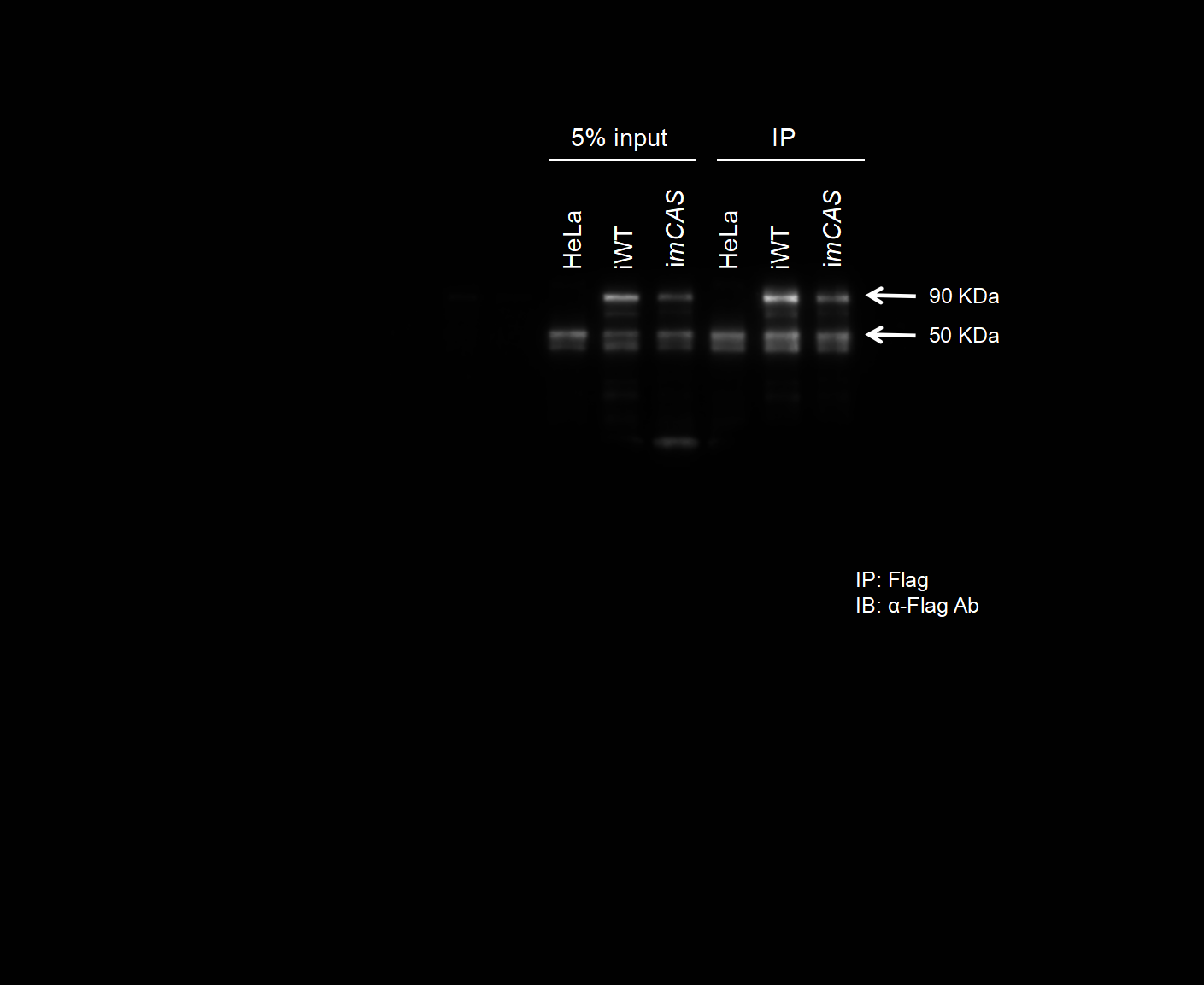

Supplement: Figure 3—source data 1. [file elife-79116-fig3-data1.zip › Figure 3-source data 1/+Label/Fig 3E-Flag antibody.Tif.tif]

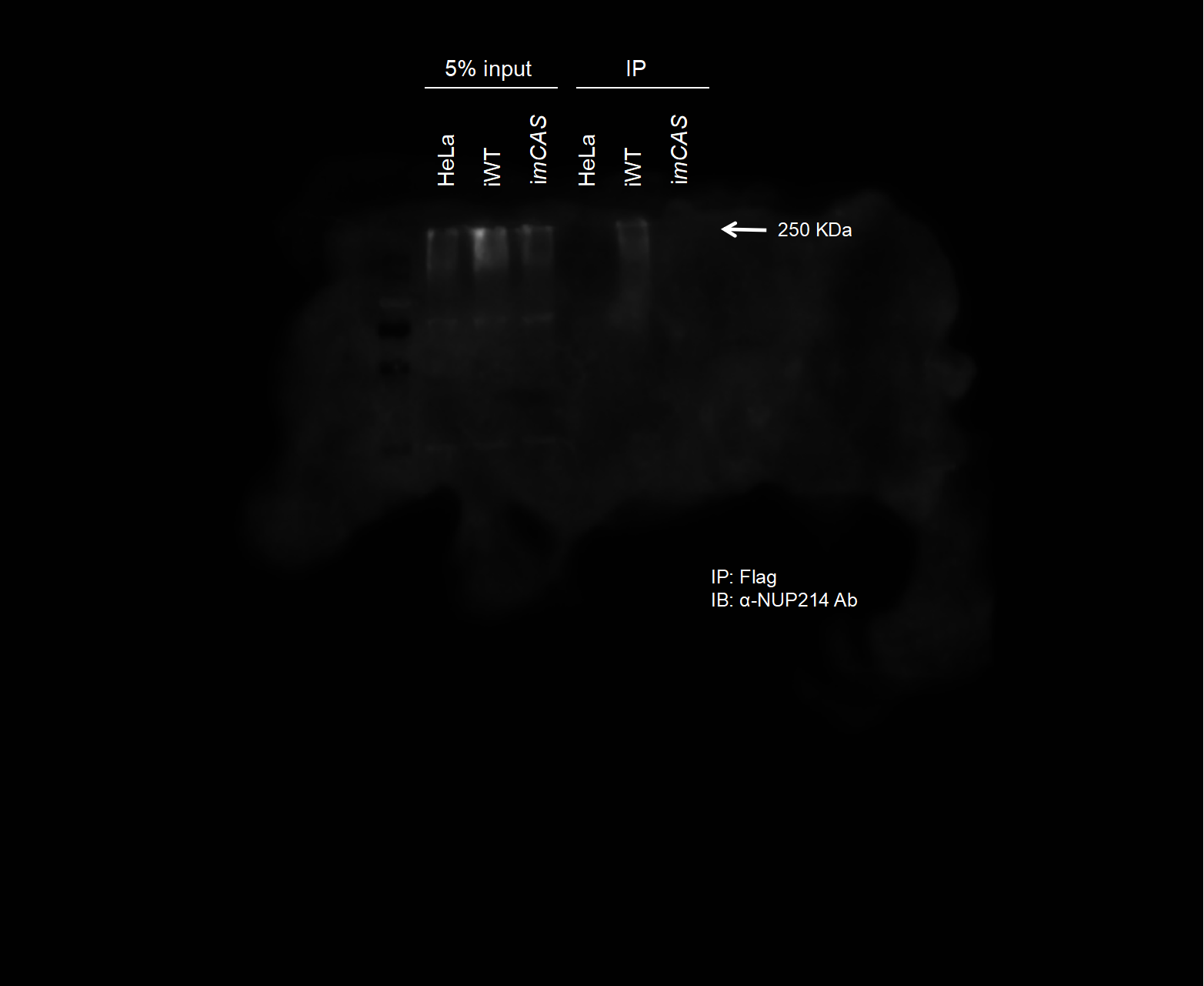

Supplement: Figure 3—source data 1. [file elife-79116-fig3-data1.zip › Figure 3-source data 1/+Label/Fig 3E-NUP214 antibody.Tif.tif]

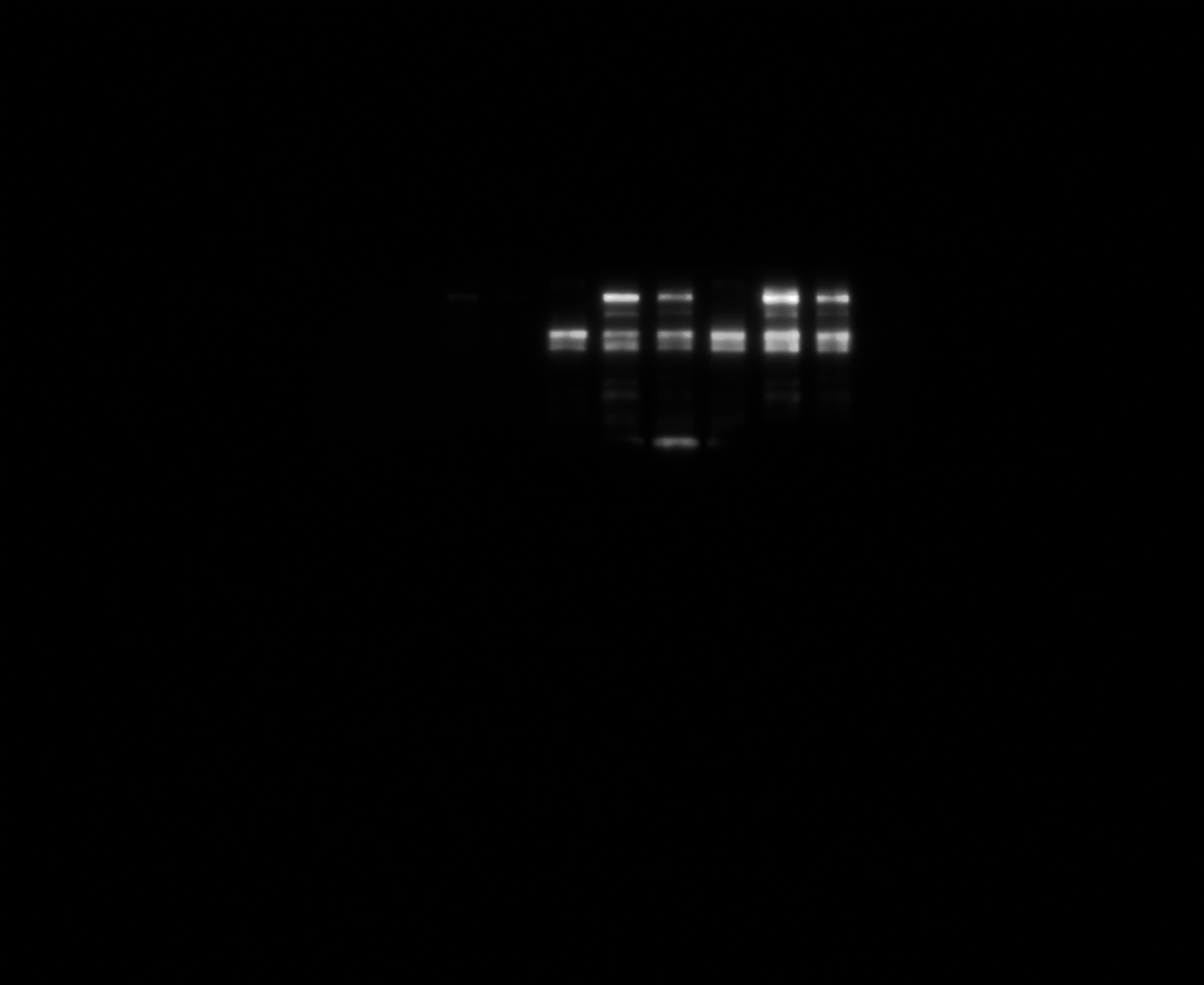

Supplement: Figure 3—source data 1. [file elife-79116-fig3-data1.zip › Figure 3-source data 1/Unedited/Fig 3E-Flag antibody.Tif]

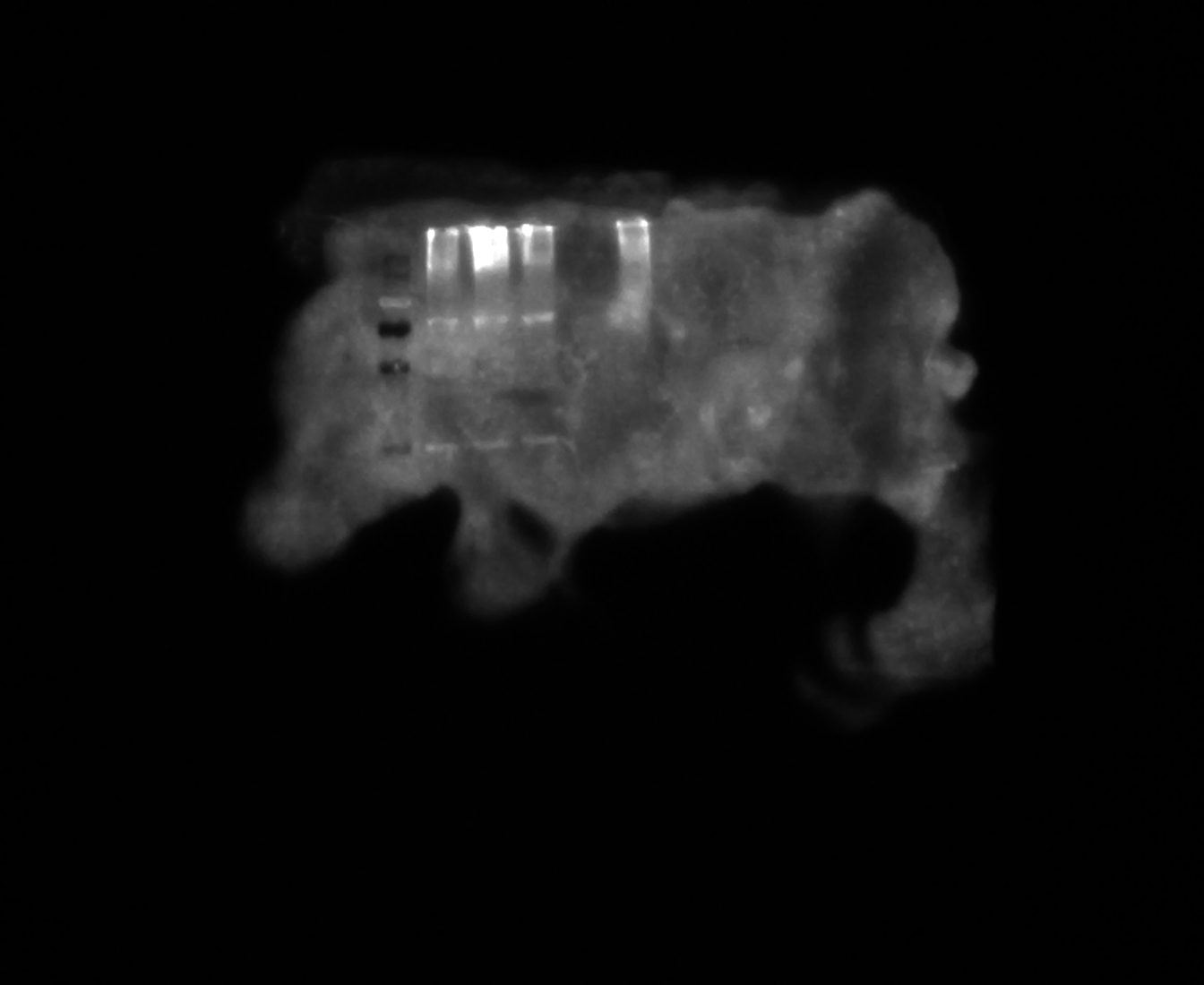

Supplement: Figure 3—source data 1. [file elife-79116-fig3-data1.zip › Figure 3-source data 1/Unedited/Fig 3E-NUP214 antibody.Tif]

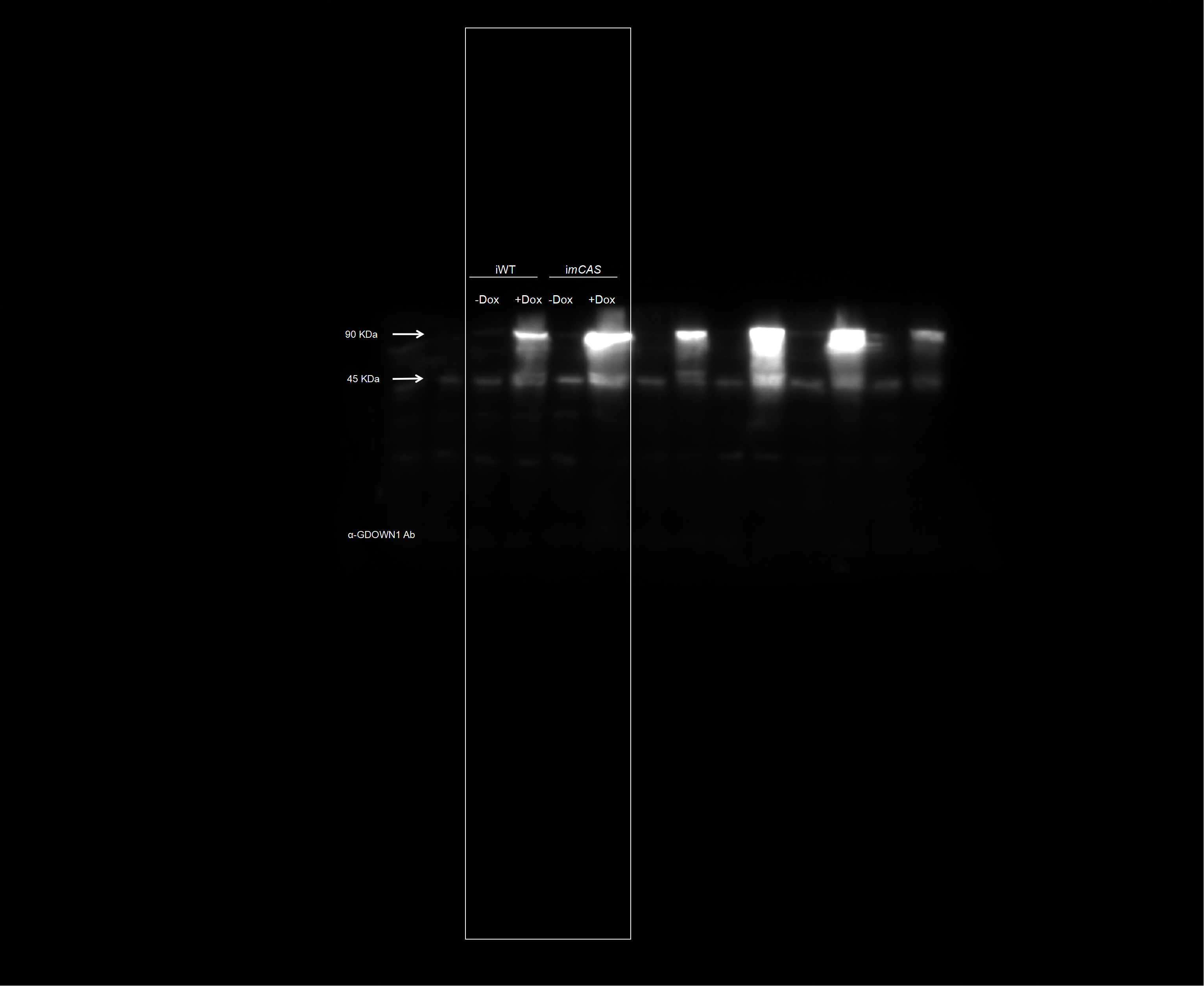

Supplement: Figure 3—figure supplement 2—source data 1. [file elife-79116-fig3-figsupp2-data1.zip › Figure 3-figure supplement 2-source data 1/+Label/Fig 3-Fig supplement 1D-GDOWN1 rabbit antibody.Tif.tif]

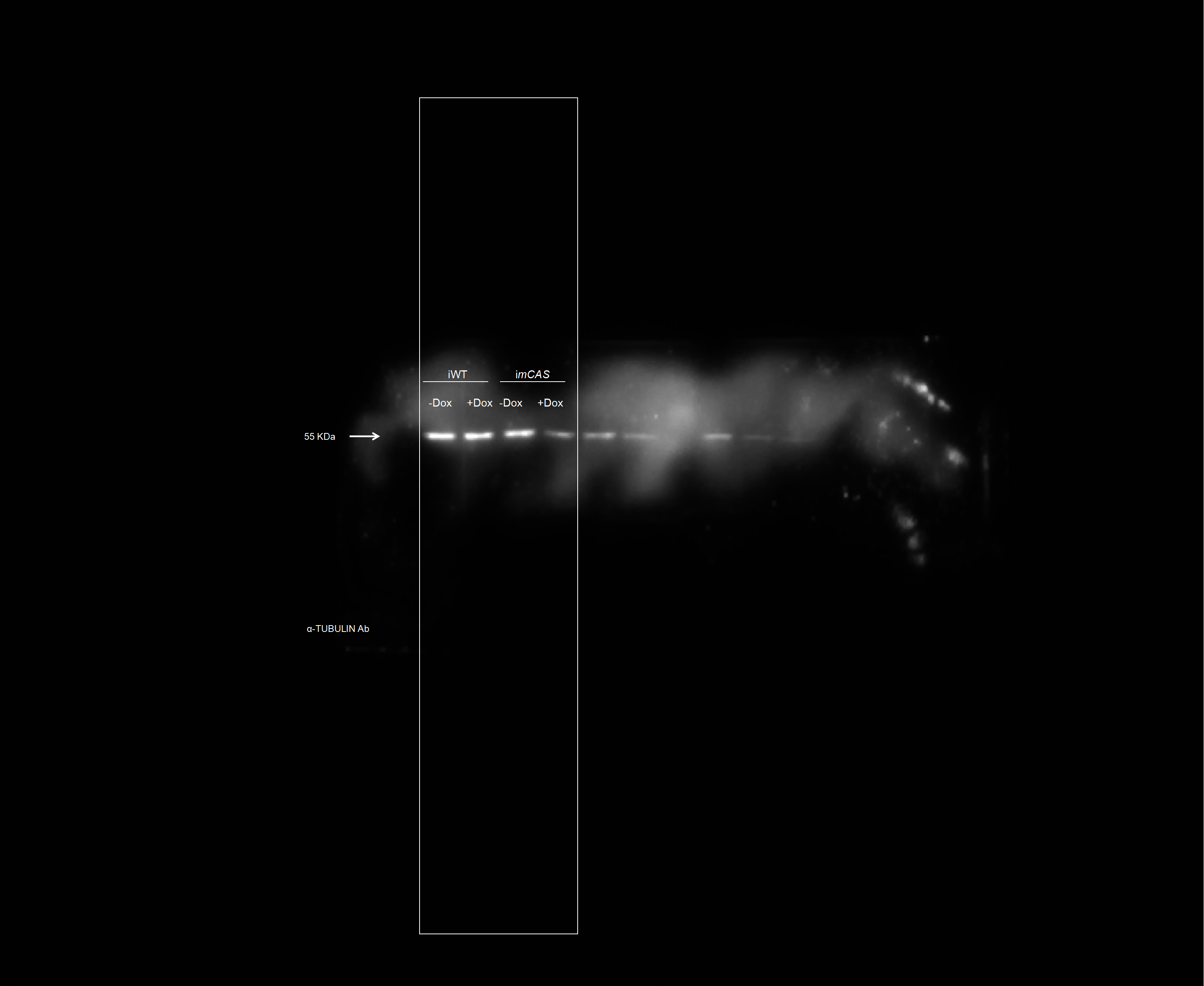

Supplement: Figure 3—figure supplement 2—source data 1. [file elife-79116-fig3-figsupp2-data1.zip › Figure 3-figure supplement 2-source data 1/+Label/Fig 3-Fig supplement 1D-TUBULIN antibody.Tif.tif]

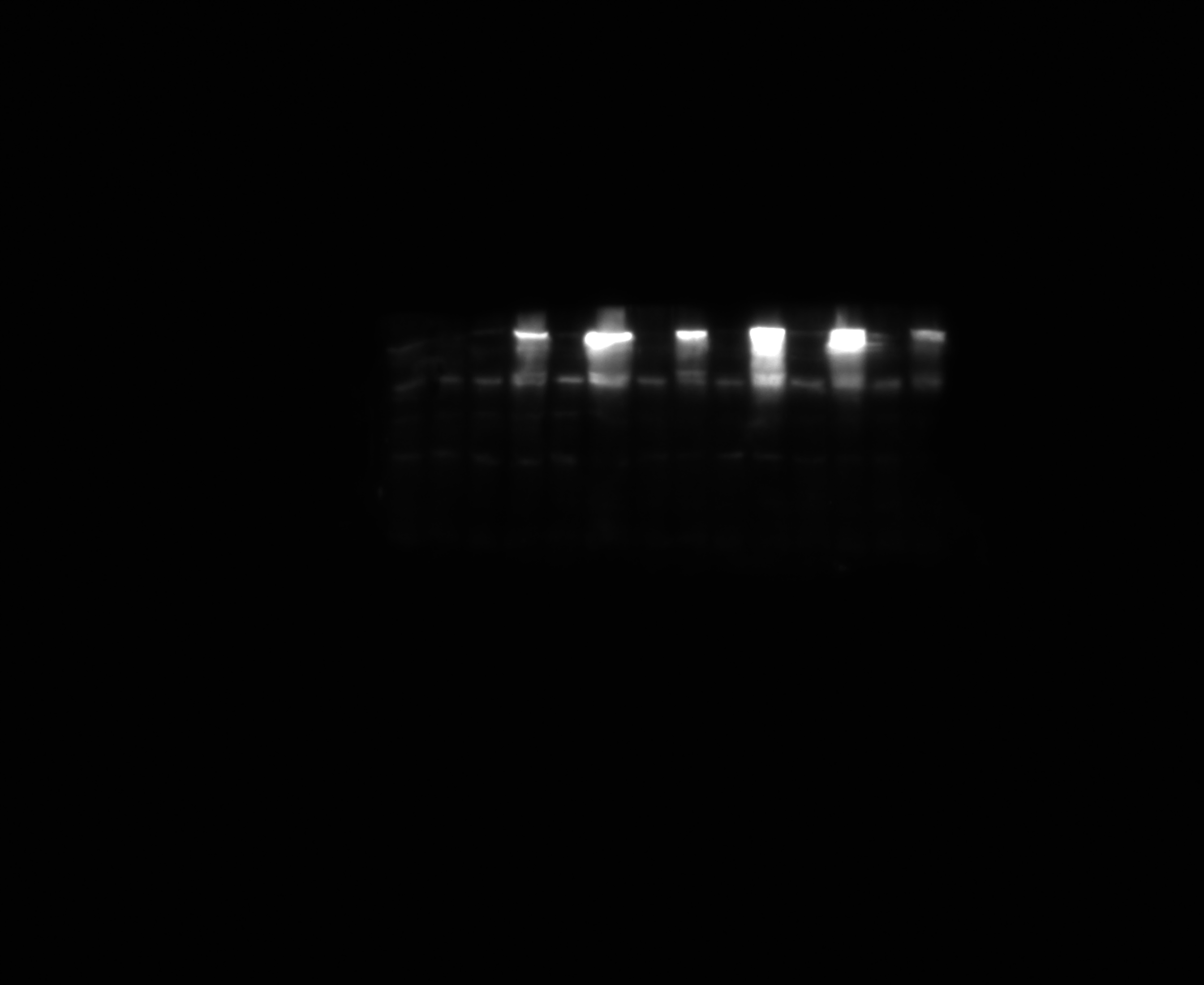

Supplement: Figure 3—figure supplement 2—source data 1. [file elife-79116-fig3-figsupp2-data1.zip › Figure 3-figure supplement 2-source data 1/Unedited/Fig 3-Fig supplement 1D-GDOWN1 rabbit antibody.Tif]

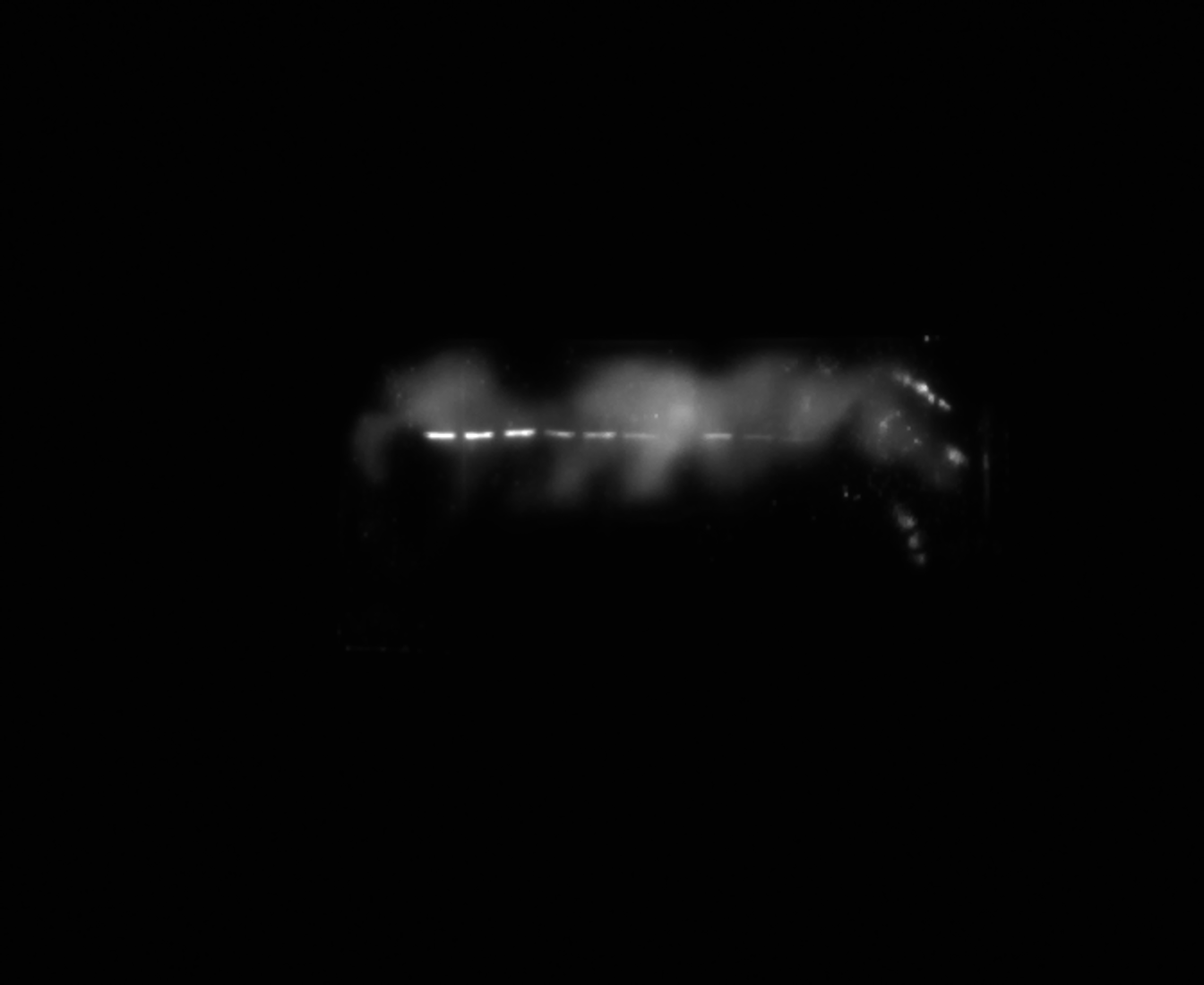

Supplement: Figure 3—figure supplement 2—source data 1. [file elife-79116-fig3-figsupp2-data1.zip › Figure 3-figure supplement 2-source data 1/Unedited/Fig 3-Fig supplement 1D-TUBULIN antibody.Tif]

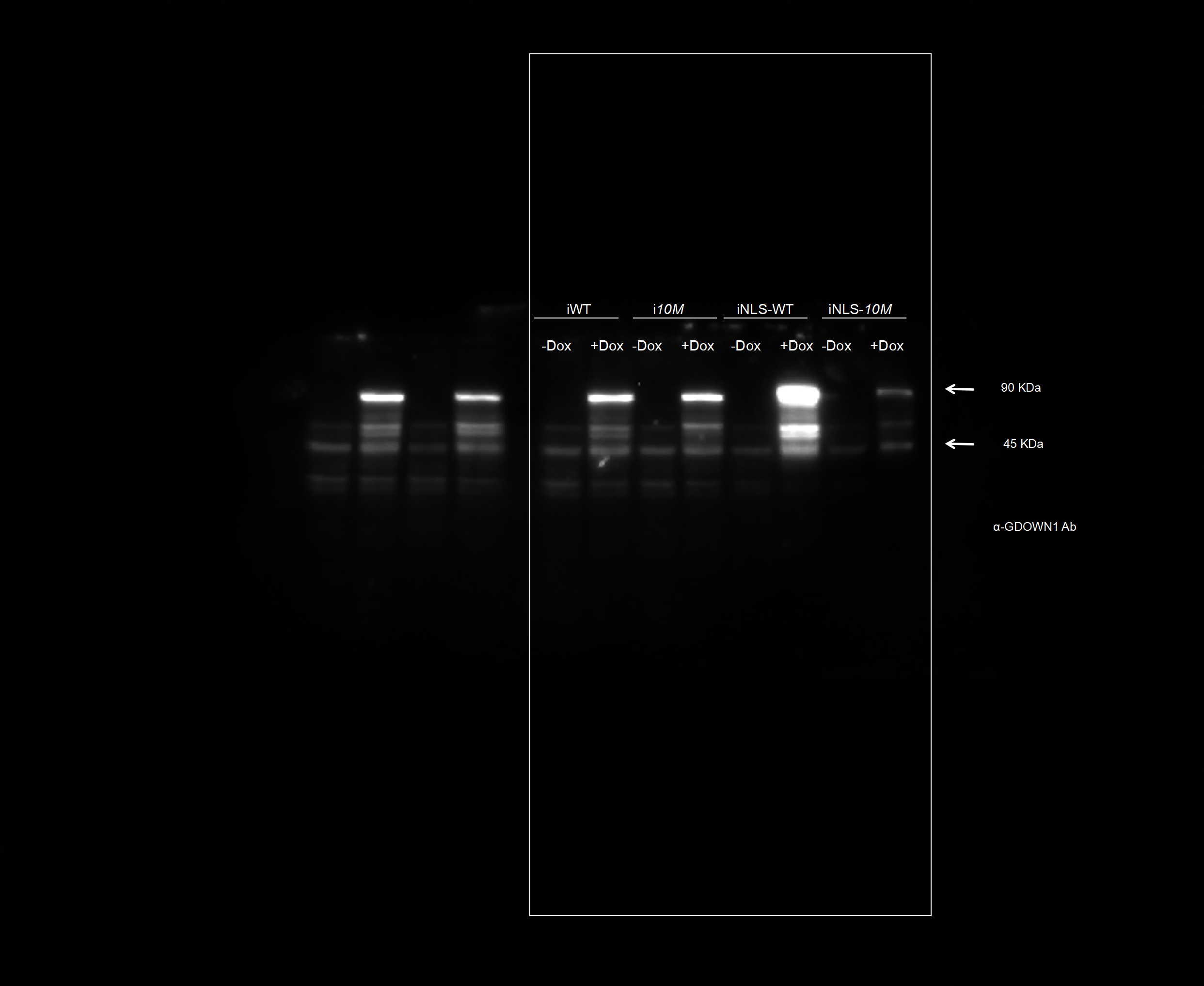

Supplement: Figure 5—figure supplement 1—source data 1. [file elife-79116-fig5-figsupp1-data1.zip › Figure 5-figure supplement 1-source data 1/+Label/Fig 5-Fig supplement 1A GDOWN1 Rabbit antibody.Tif.tif]

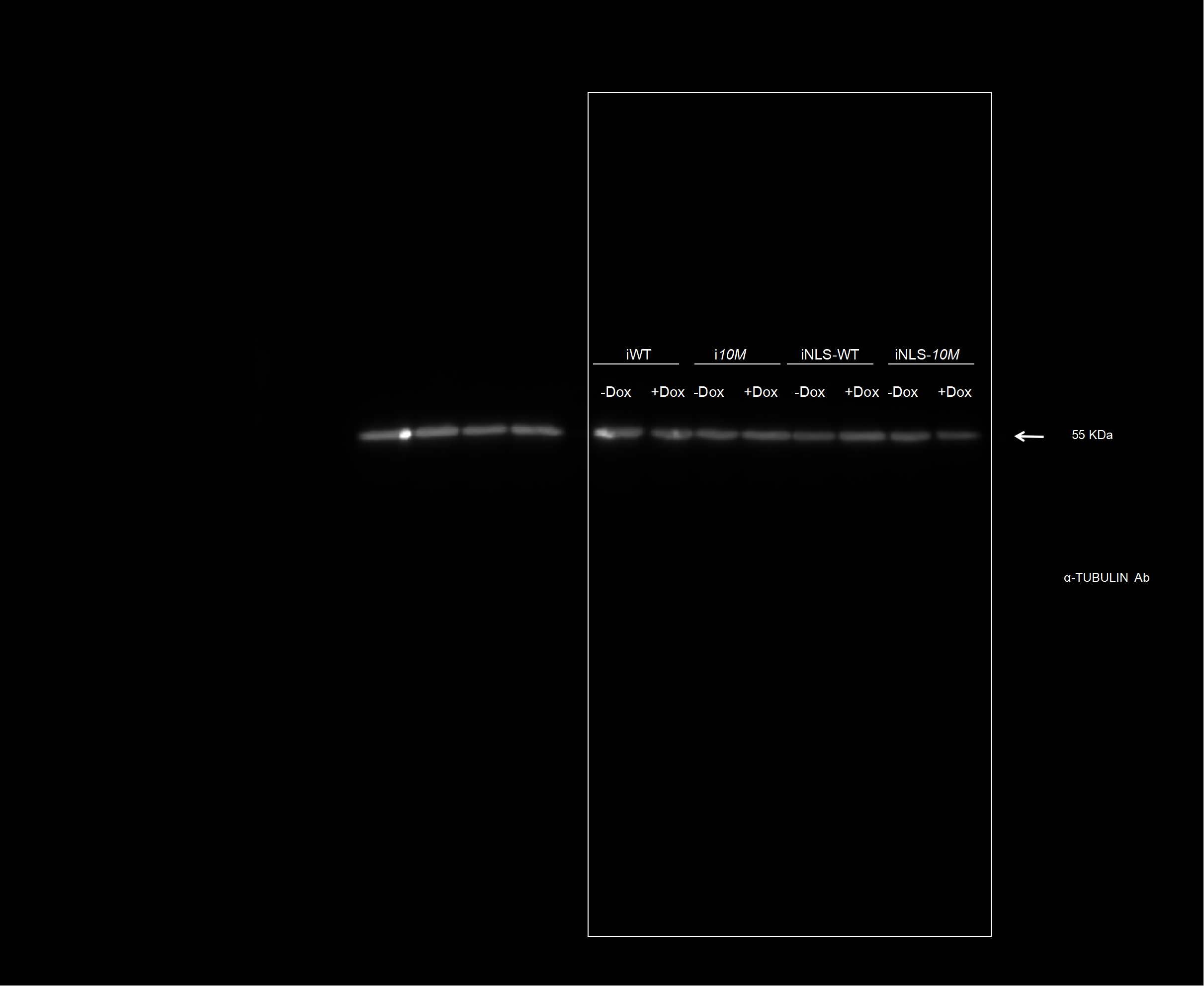

Supplement: Figure 5—figure supplement 1—source data 1. [file elife-79116-fig5-figsupp1-data1.zip › Figure 5-figure supplement 1-source data 1/+Label/Fig 5-Fig supplement 1A TUNULIN.Tif.tif]

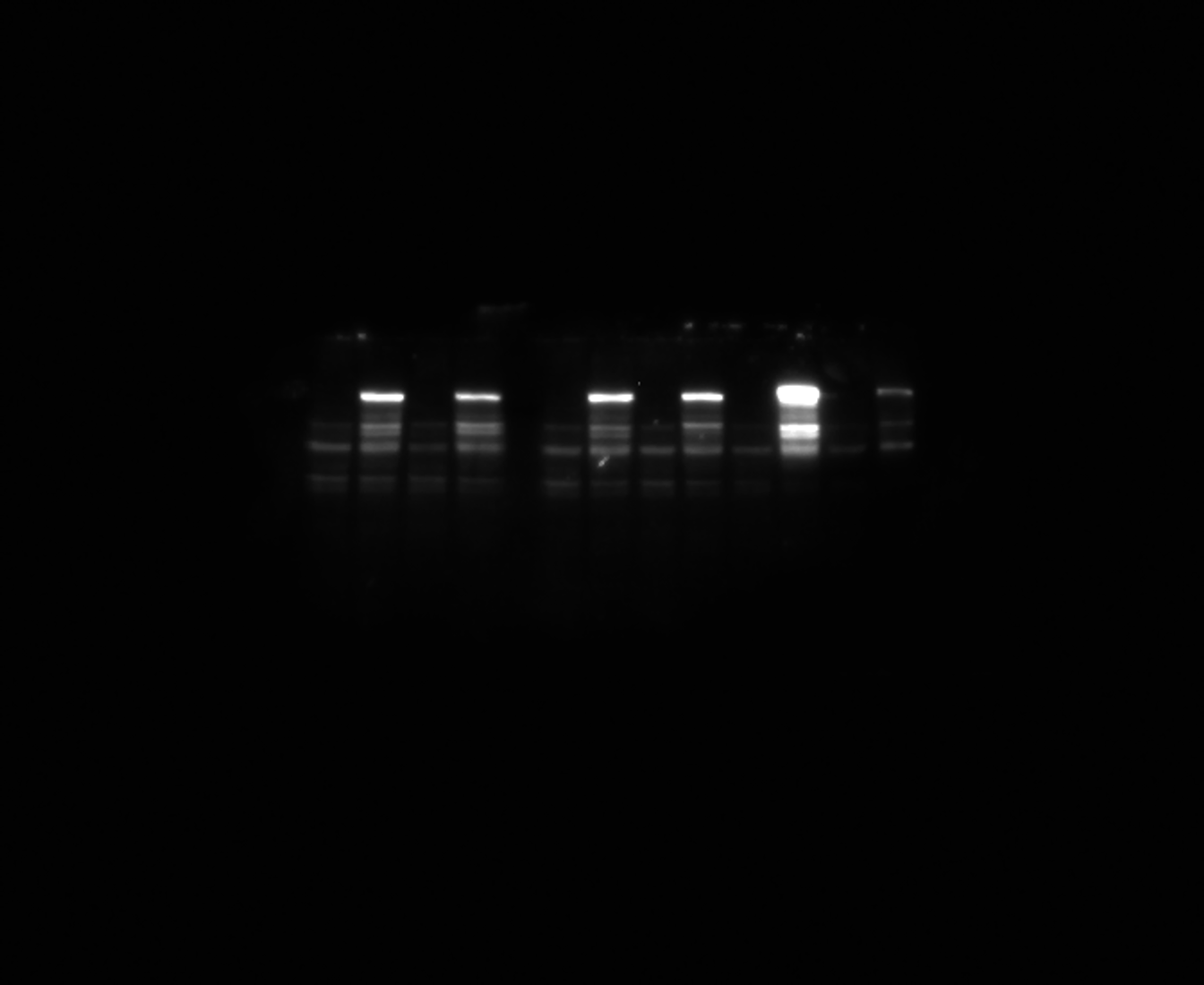

Supplement: Figure 5—figure supplement 1—source data 1. [file elife-79116-fig5-figsupp1-data1.zip › Figure 5-figure supplement 1-source data 1/Unedited/Fig 5-Fig supplement 1A GDOWN1 Rabbit antibody.Tif]

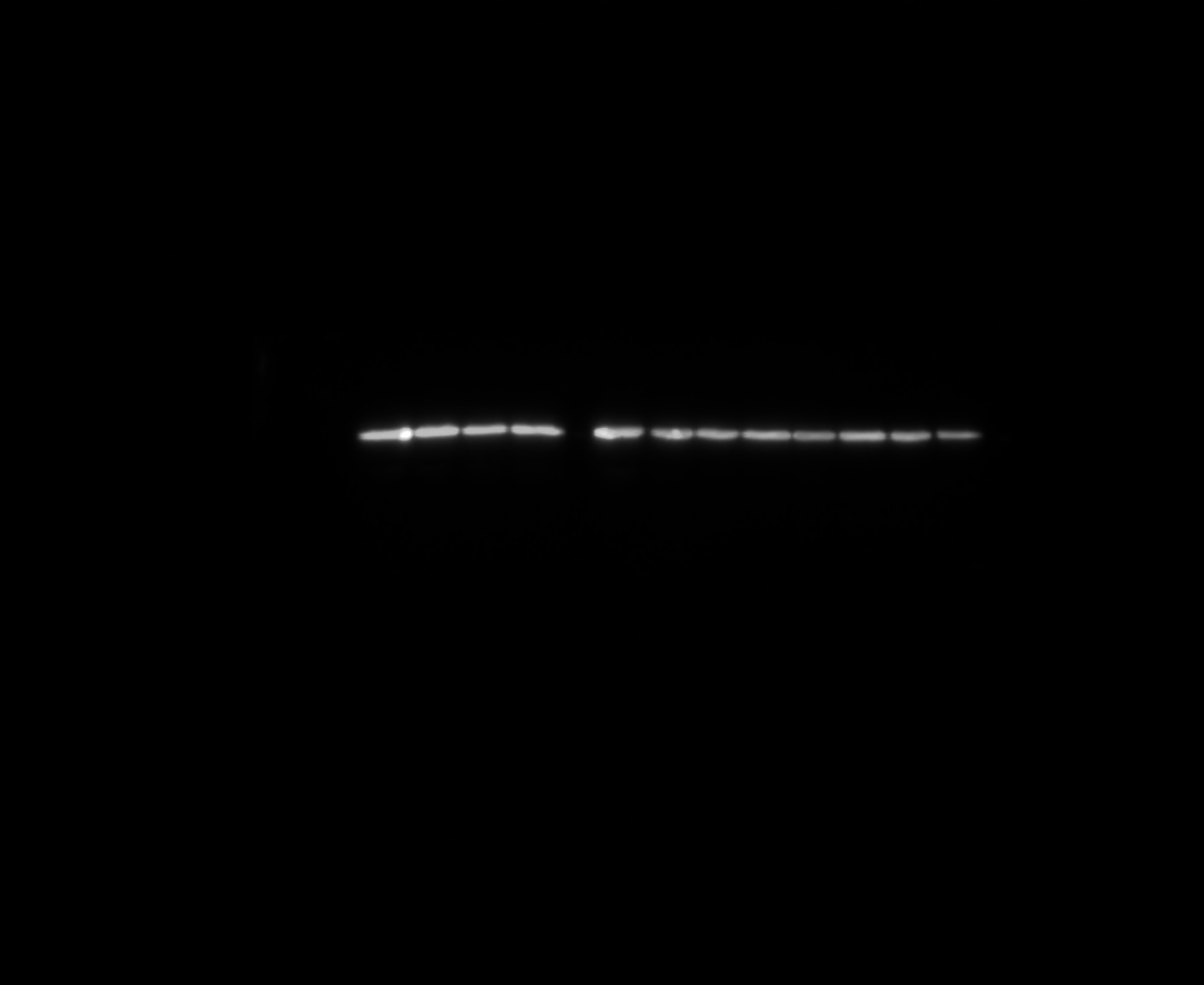

Supplement: Figure 5—figure supplement 1—source data 1. [file elife-79116-fig5-figsupp1-data1.zip › Figure 5-figure supplement 1-source data 1/Unedited/Fig 5-Fig supplement 1A TUBULIN antibody.Tif]

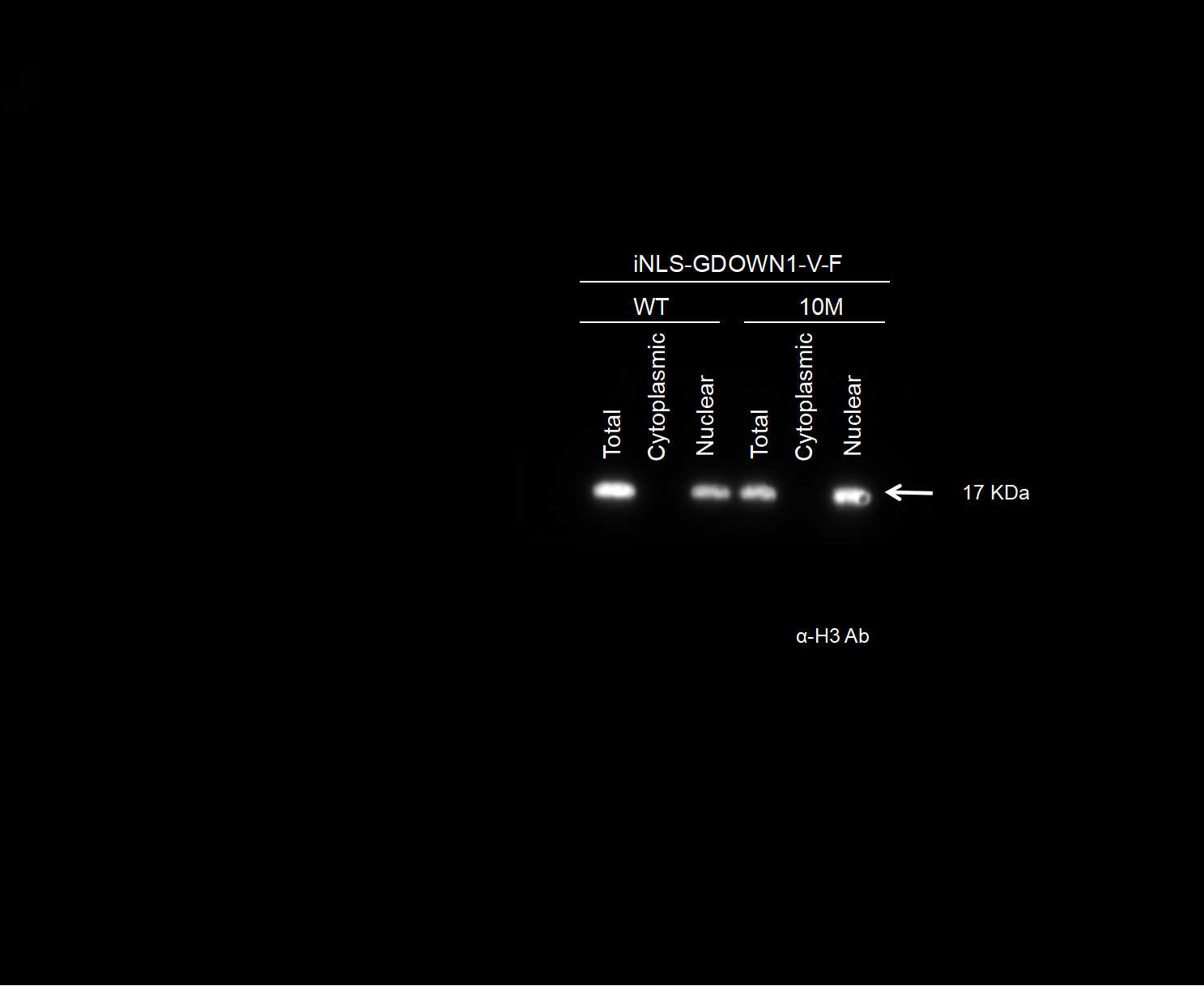

Supplement: Figure 6—source data 2. [file elife-79116-fig6-data2.zip › Figure 6-source data 2/+Label/Fig6B-H3 antibody (For NLS-WT, NLS-10M).tif]

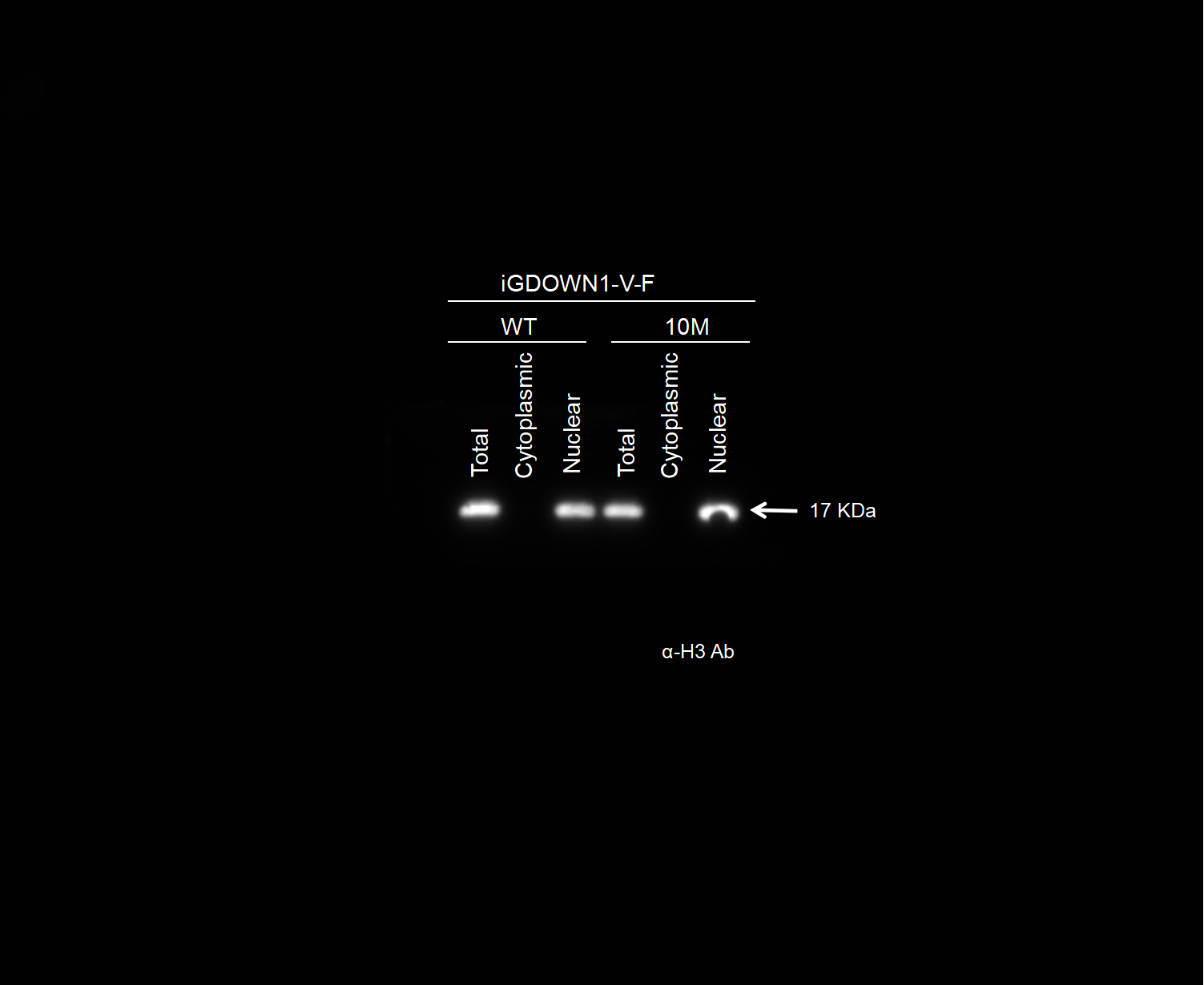

Supplement: Figure 6—source data 2. [file elife-79116-fig6-data2.zip › Figure 6-source data 2/+Label/Fig6B-H3 antibody (For WT, 10M).tif]

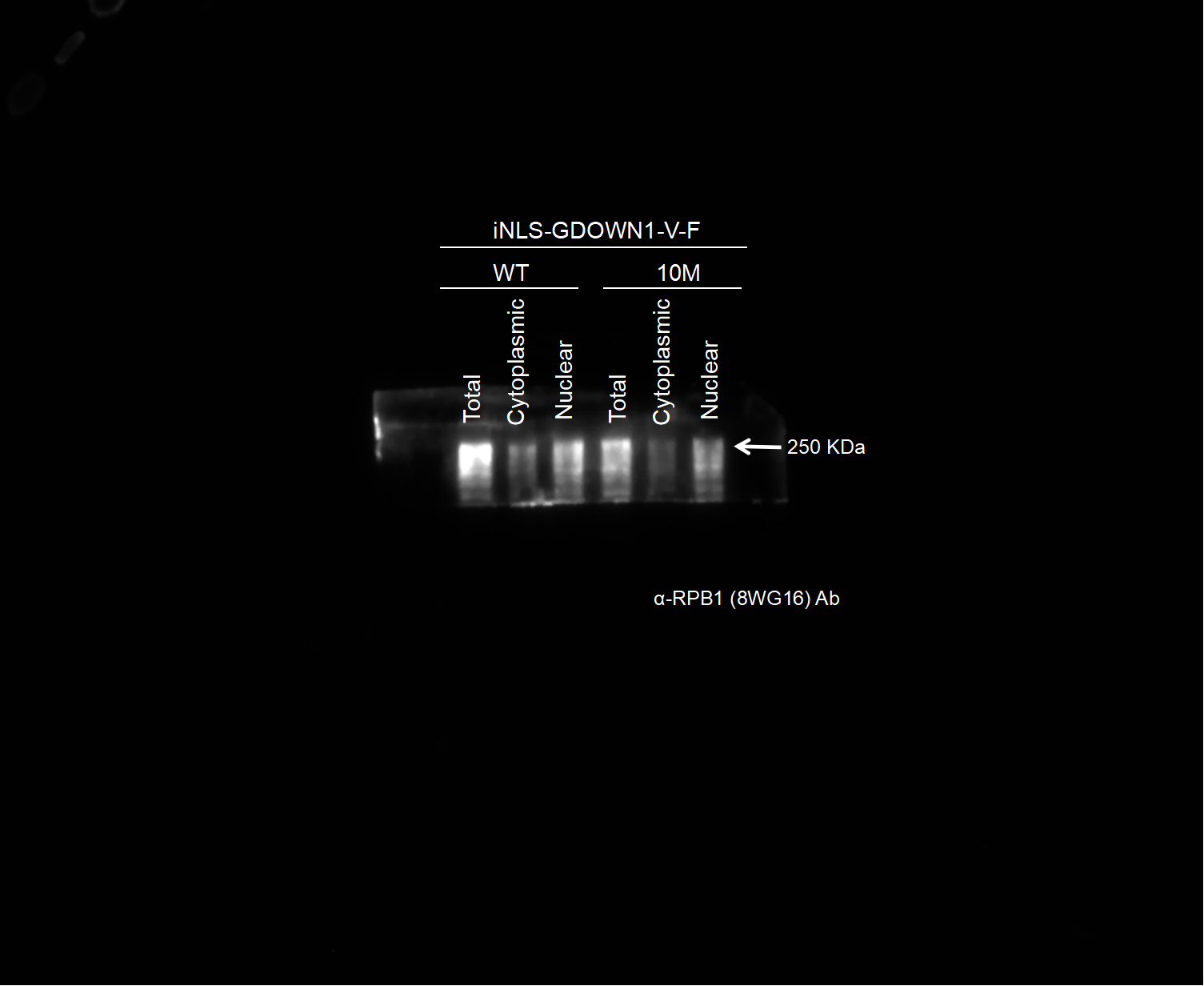

Supplement: Figure 6—source data 2. [file elife-79116-fig6-data2.zip › Figure 6-source data 2/+Label/Fig6B-RPB1(NTD) antibody (For NLS-WT, NLS-10M).tif]

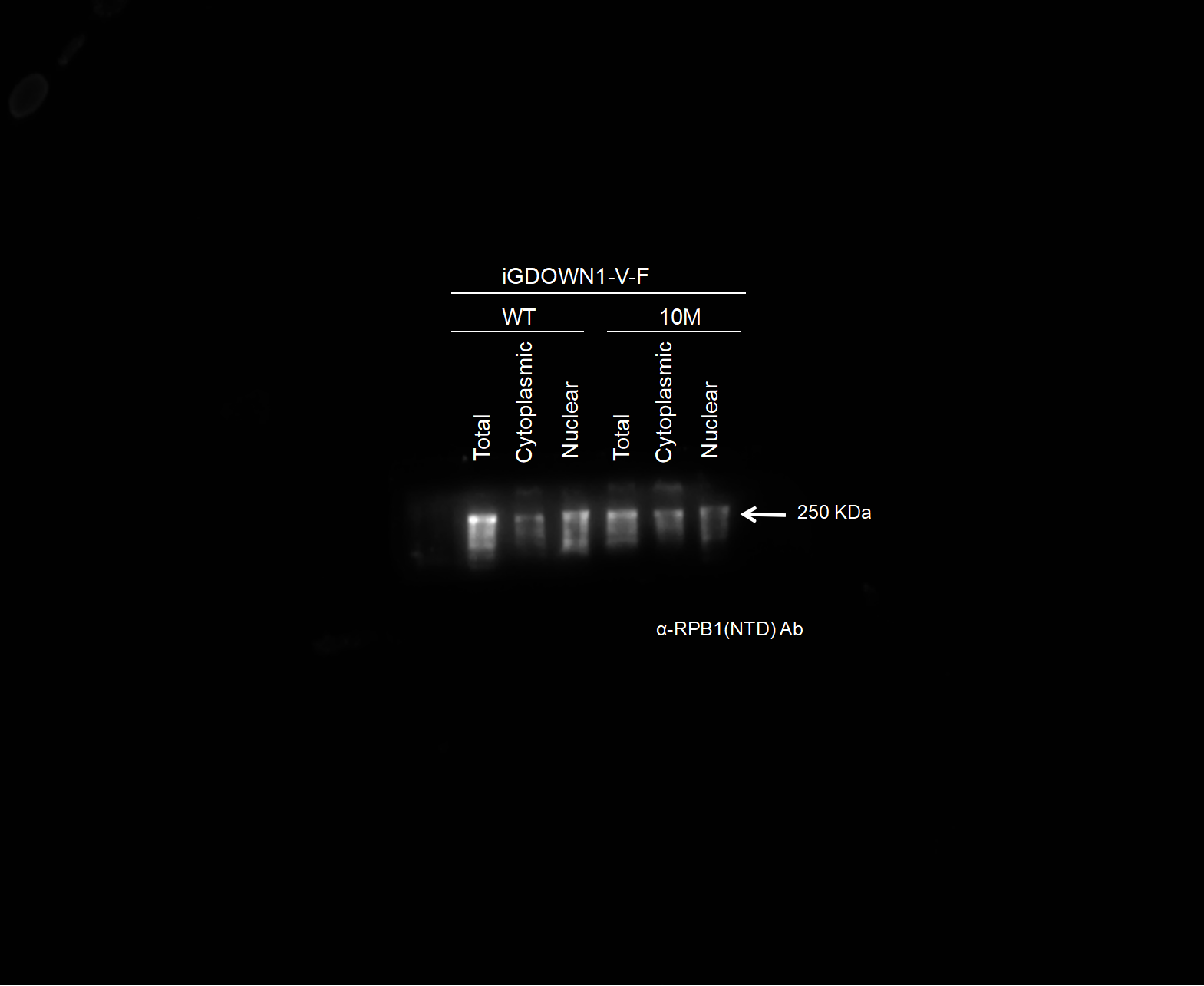

Supplement: Figure 6—source data 2. [file elife-79116-fig6-data2.zip › Figure 6-source data 2/+Label/Fig6B-RPB1(NTD) antibody (For WT, 10M).tif]

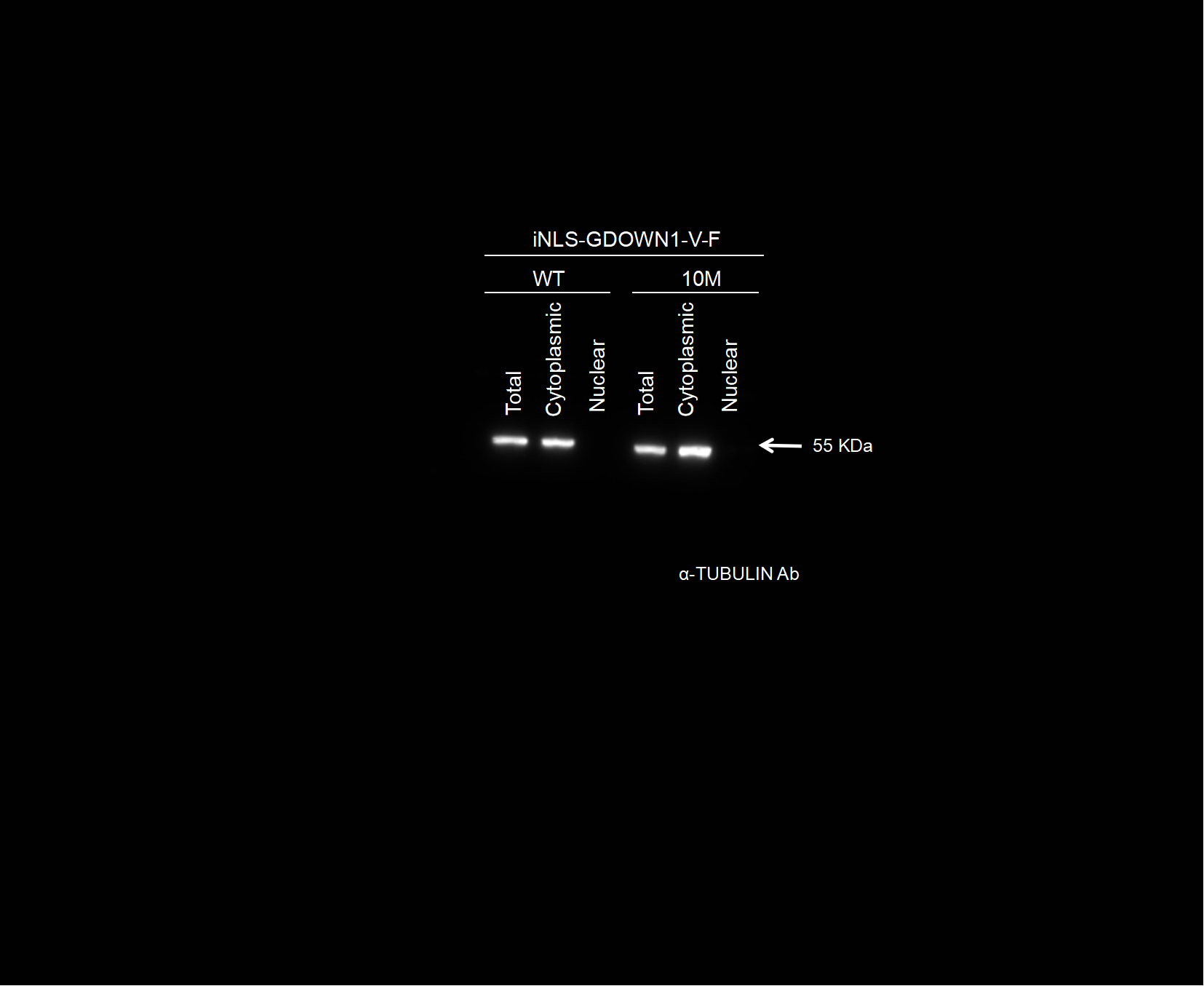

Supplement: Figure 6—source data 2. [file elife-79116-fig6-data2.zip › Figure 6-source data 2/+Label/Fig6B-TUBULIN antibody (For NLS-WT, NLS-10M).tif]

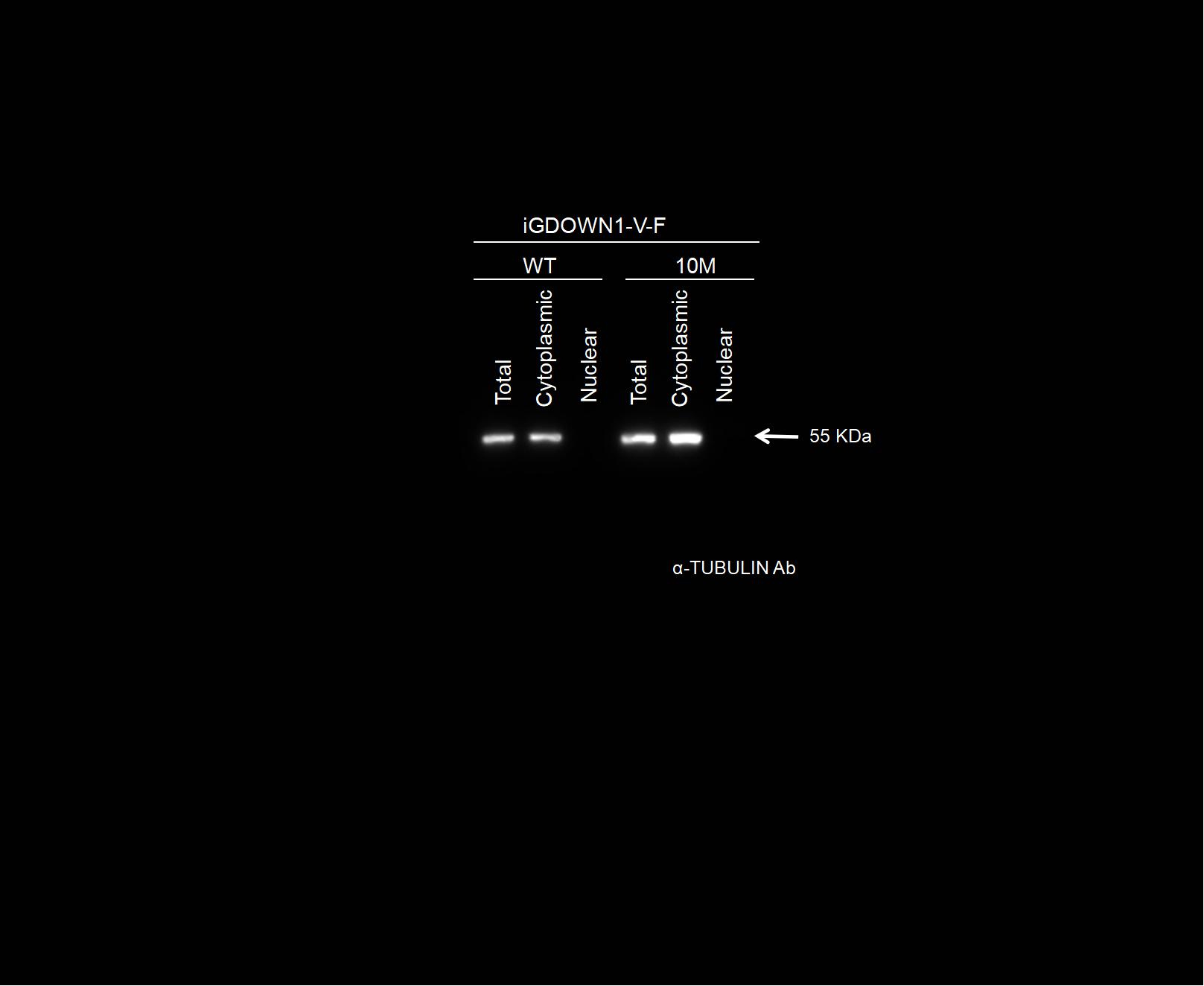

Supplement: Figure 6—source data 2. [file elife-79116-fig6-data2.zip › Figure 6-source data 2/+Label/Fig6B-TUBULIN antibody (For WT, 10M).tif]

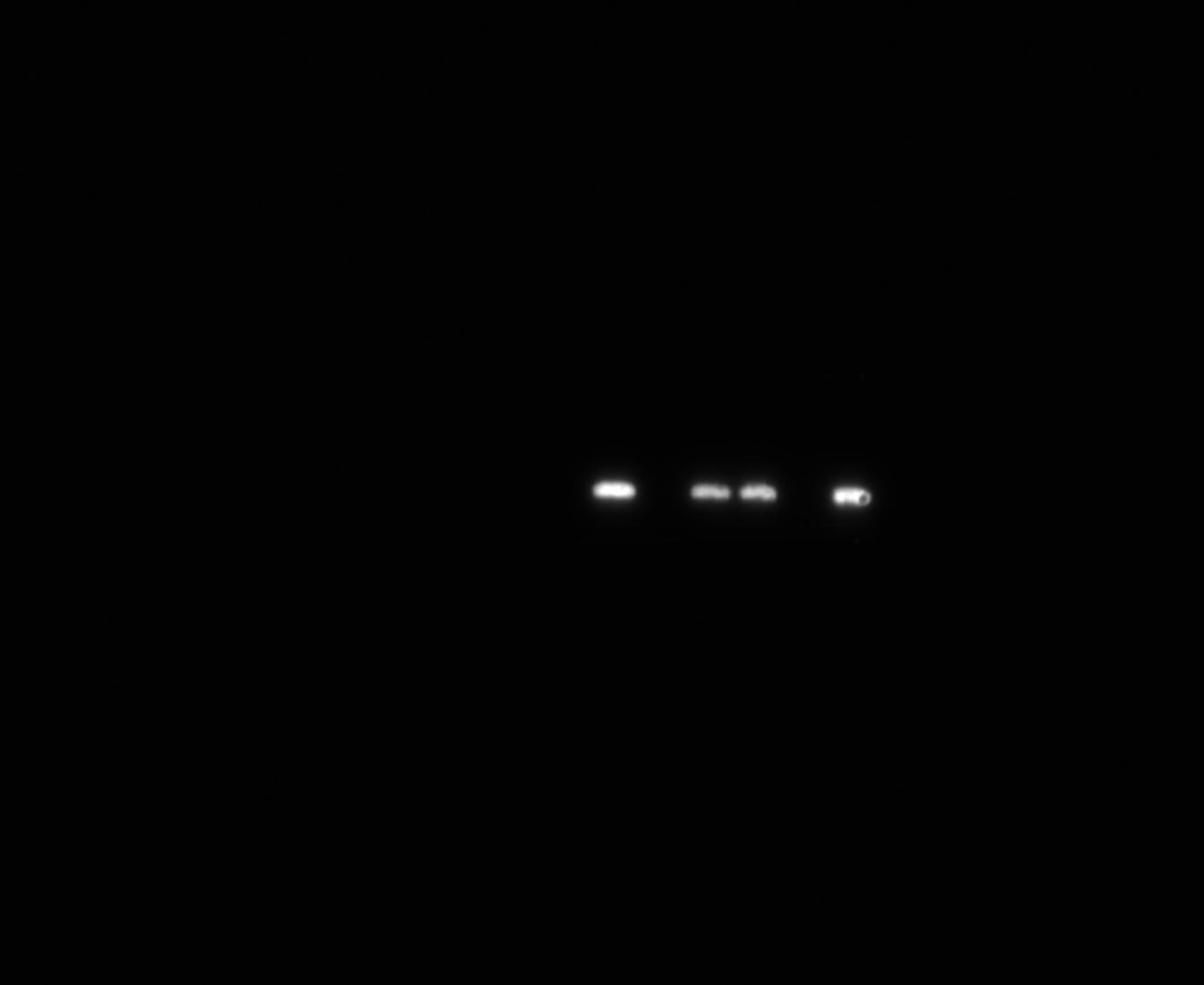

Supplement: Figure 6—source data 2. [file elife-79116-fig6-data2.zip › Figure 6-source data 2/Unedited/Fig6B-H3 antibody (For NLS-WT, NLS-10M).Tif]

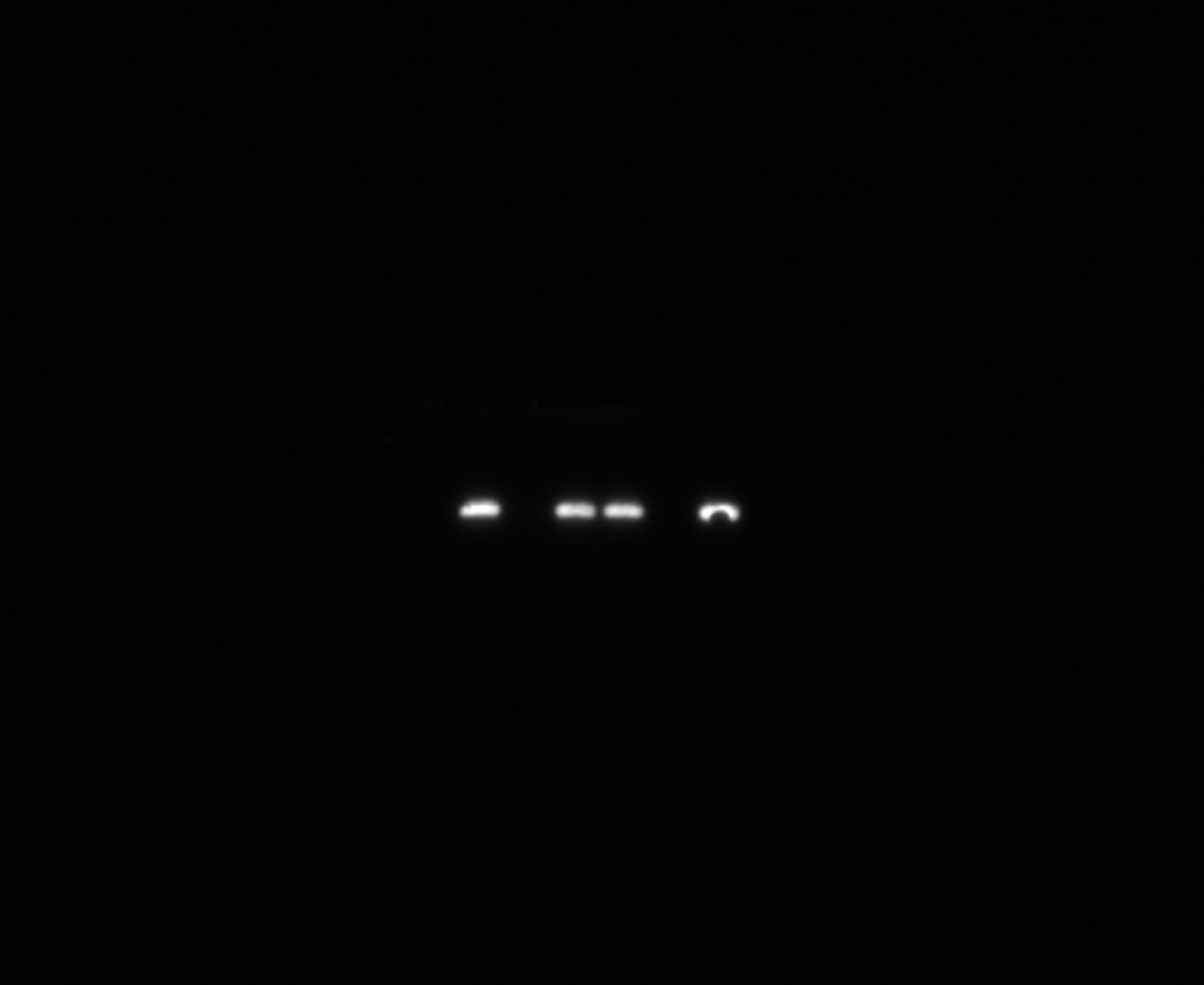

Supplement: Figure 6—source data 2. [file elife-79116-fig6-data2.zip › Figure 6-source data 2/Unedited/Fig6B-H3 antibody (For WT, 10M).Tif]

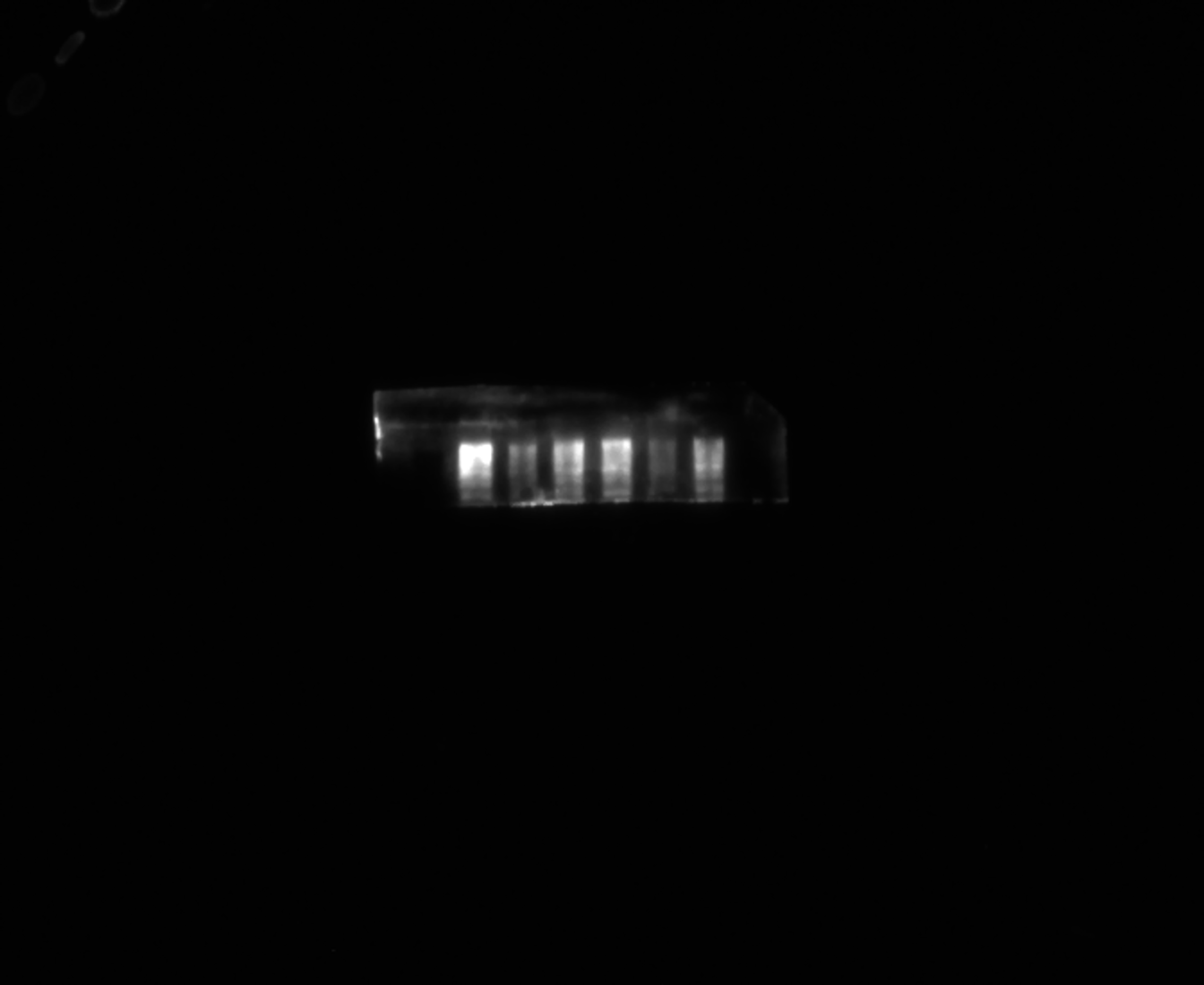

Supplement: Figure 6—source data 2. [file elife-79116-fig6-data2.zip › Figure 6-source data 2/Unedited/Fig6B-RPB1(NTD) antibody (For NLS-WT, NLS-10M).Tif]

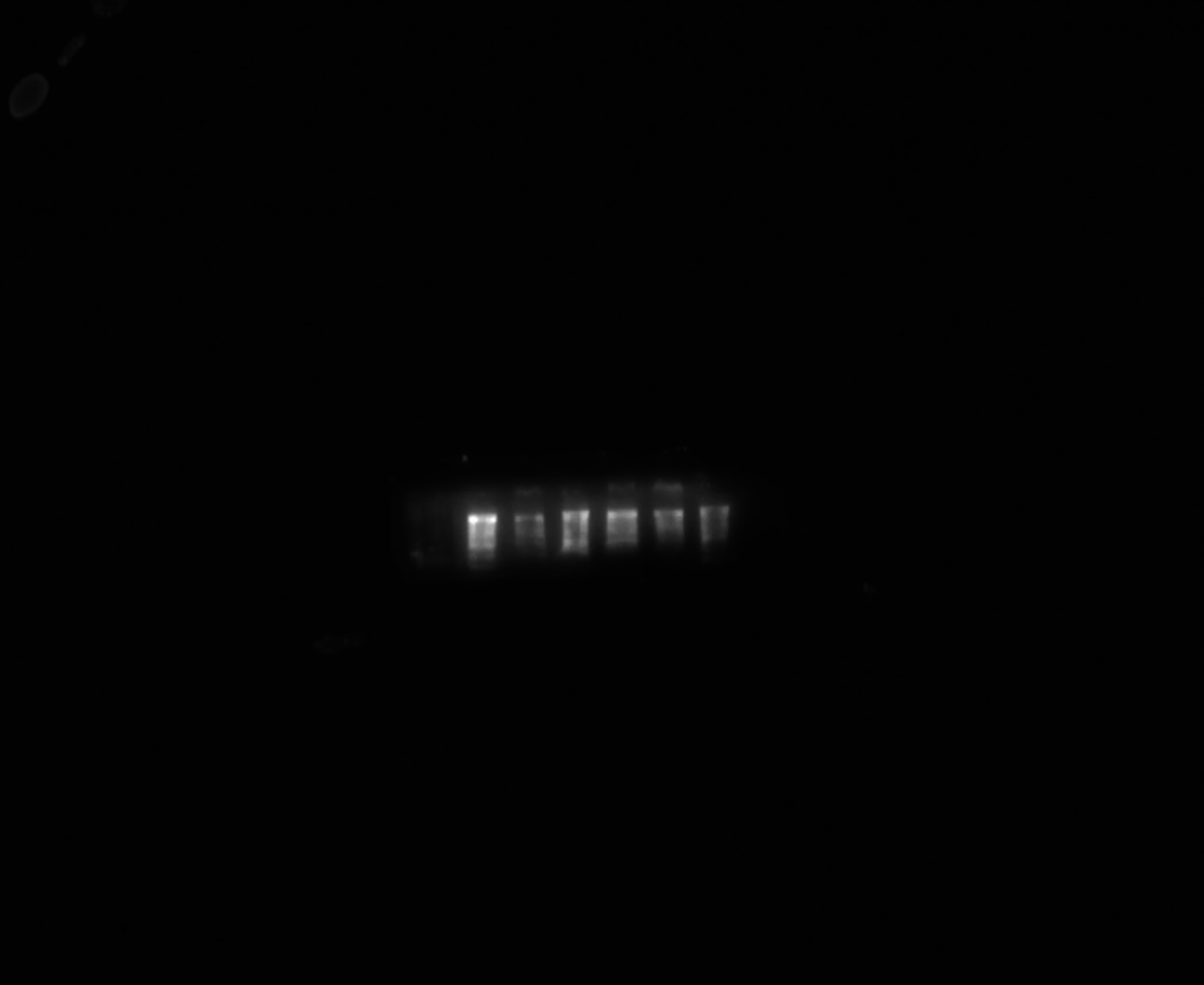

Supplement: Figure 6—source data 2. [file elife-79116-fig6-data2.zip › Figure 6-source data 2/Unedited/Fig6B-RPB1(NTD) antibody (For WT, 10M).Tif]

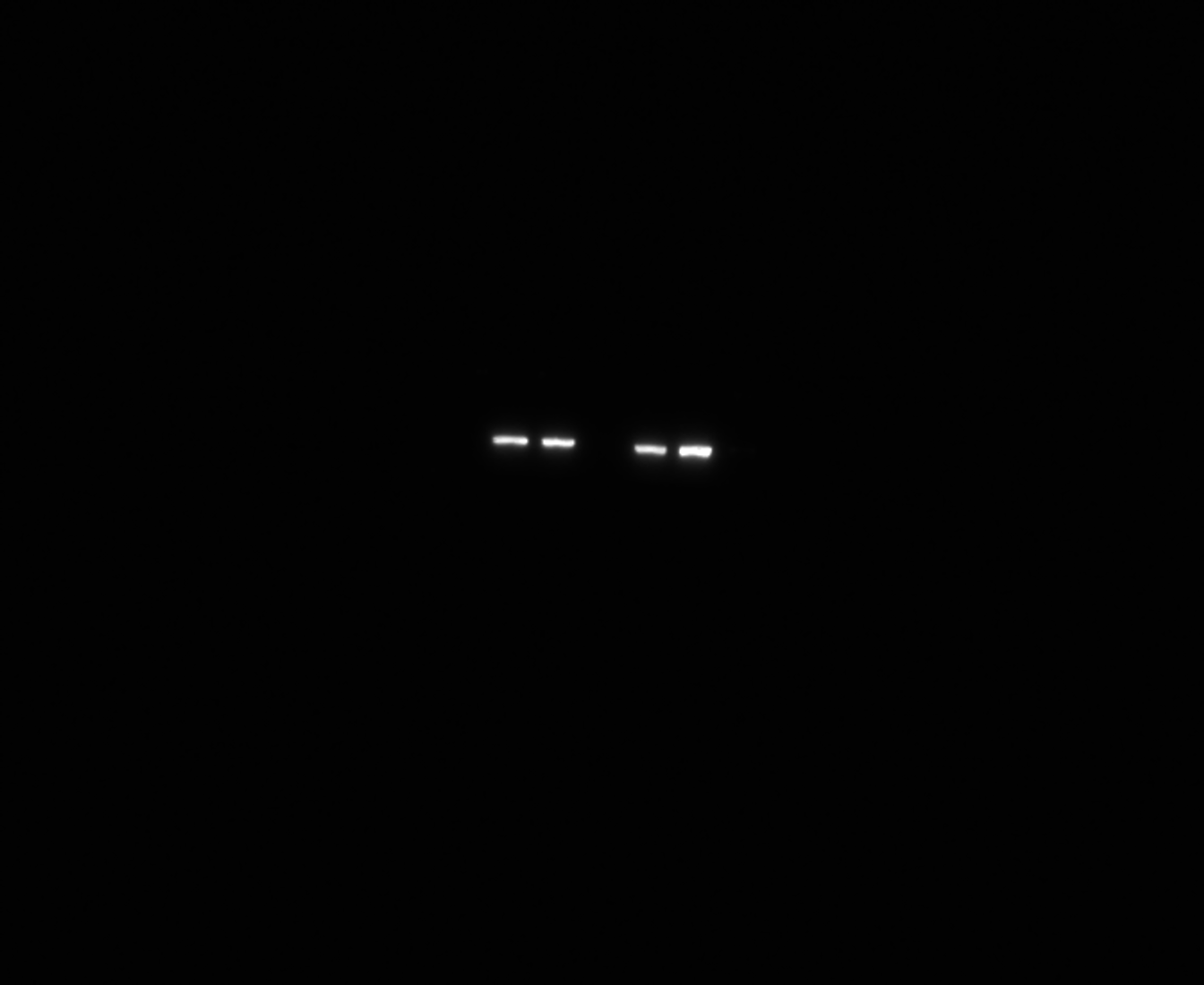

Supplement: Figure 6—source data 2. [file elife-79116-fig6-data2.zip › Figure 6-source data 2/Unedited/Fig6B-TUBULIN antibody (For NLS-WT, NLS-10M).Tif]

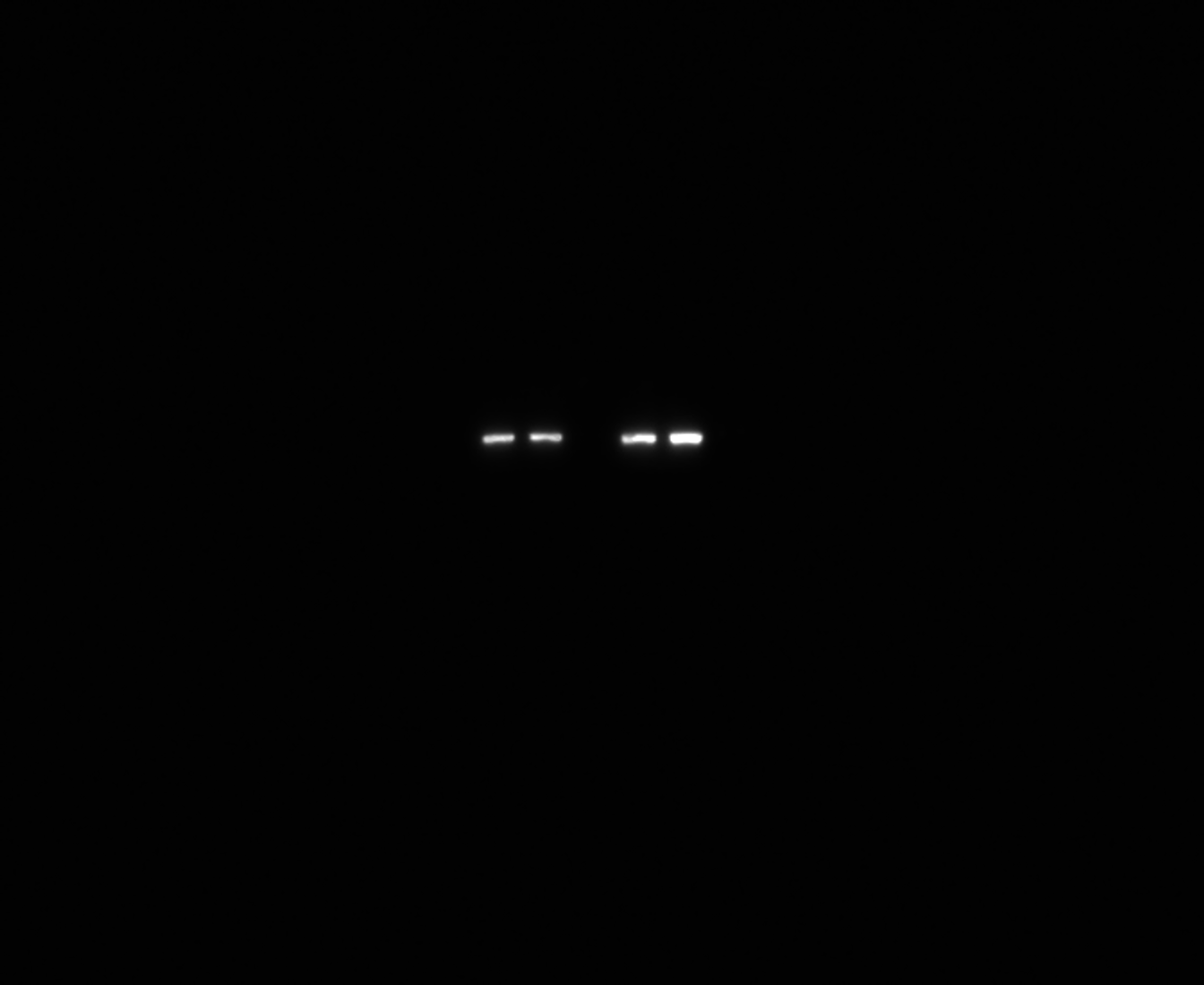

Supplement: Figure 6—source data 2. [file elife-79116-fig6-data2.zip › Figure 6-source data 2/Unedited/Fig6B-TUBULIN antibody (For WT, 10M).Tif]

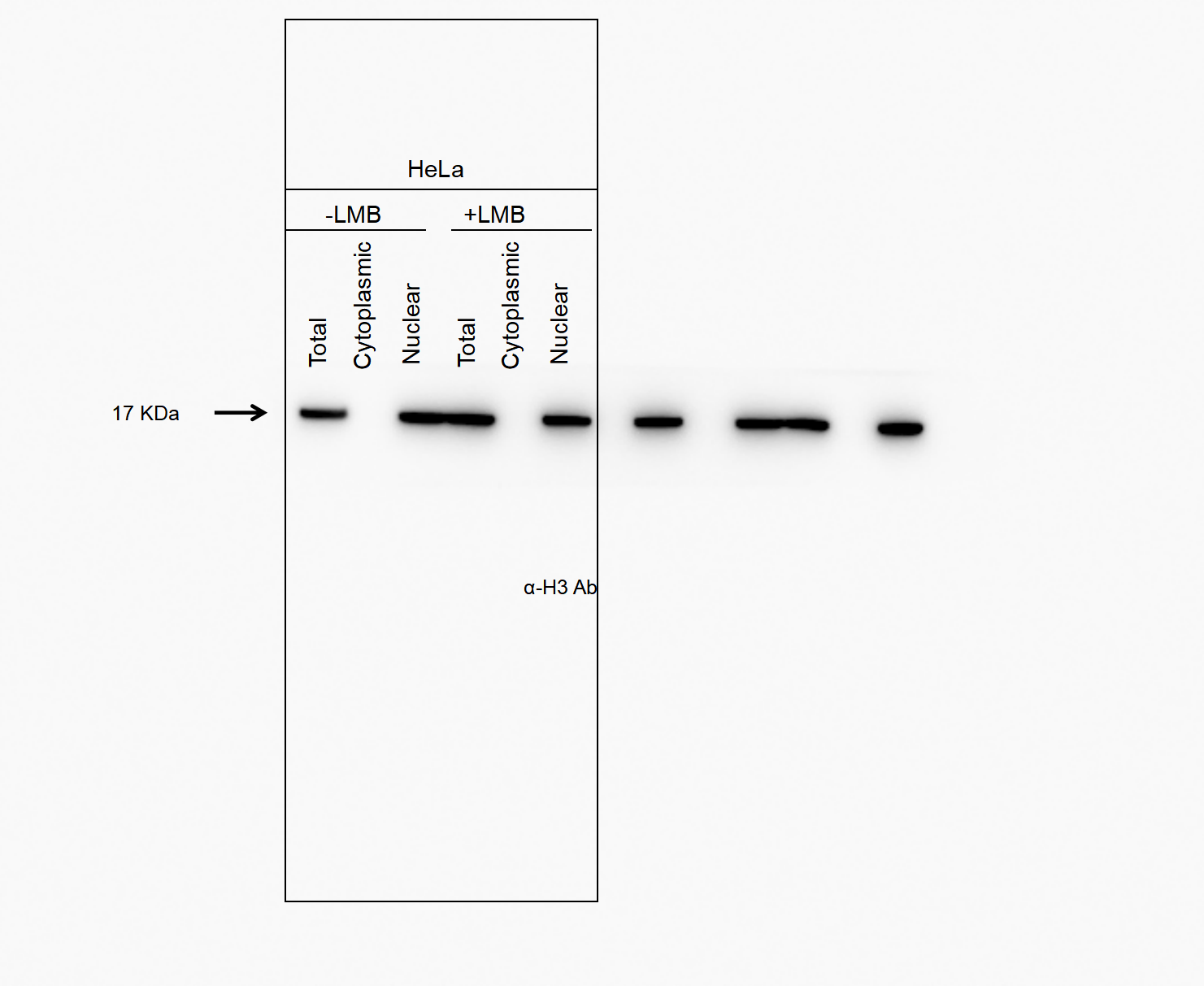

Supplement: Figure 6—figure supplement 1—source data 1. [file elife-79116-fig6-figsupp1-data1.zip › Figure 6-figure supplement 1-source data 1/+Label/Fig6-supple 1A-H3 antibody (For HeLa - or + LMB).Tif.tif]

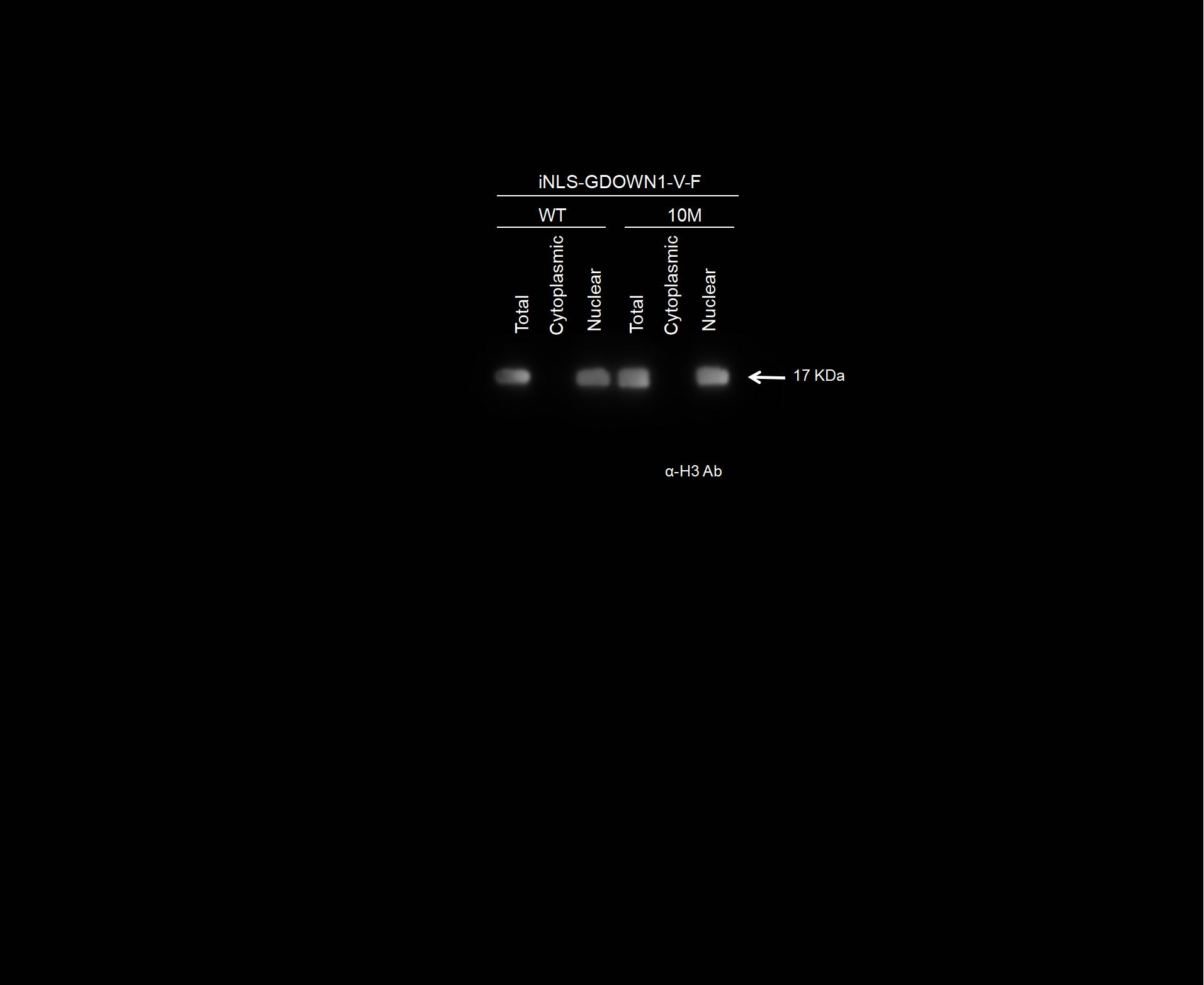

Supplement: Figure 6—figure supplement 1—source data 1. [file elife-79116-fig6-figsupp1-data1.zip › Figure 6-figure supplement 1-source data 1/+Label/Fig6-supple 1A-H3 antibody (For NLS-WT, NLS-10M).Tif.tif]

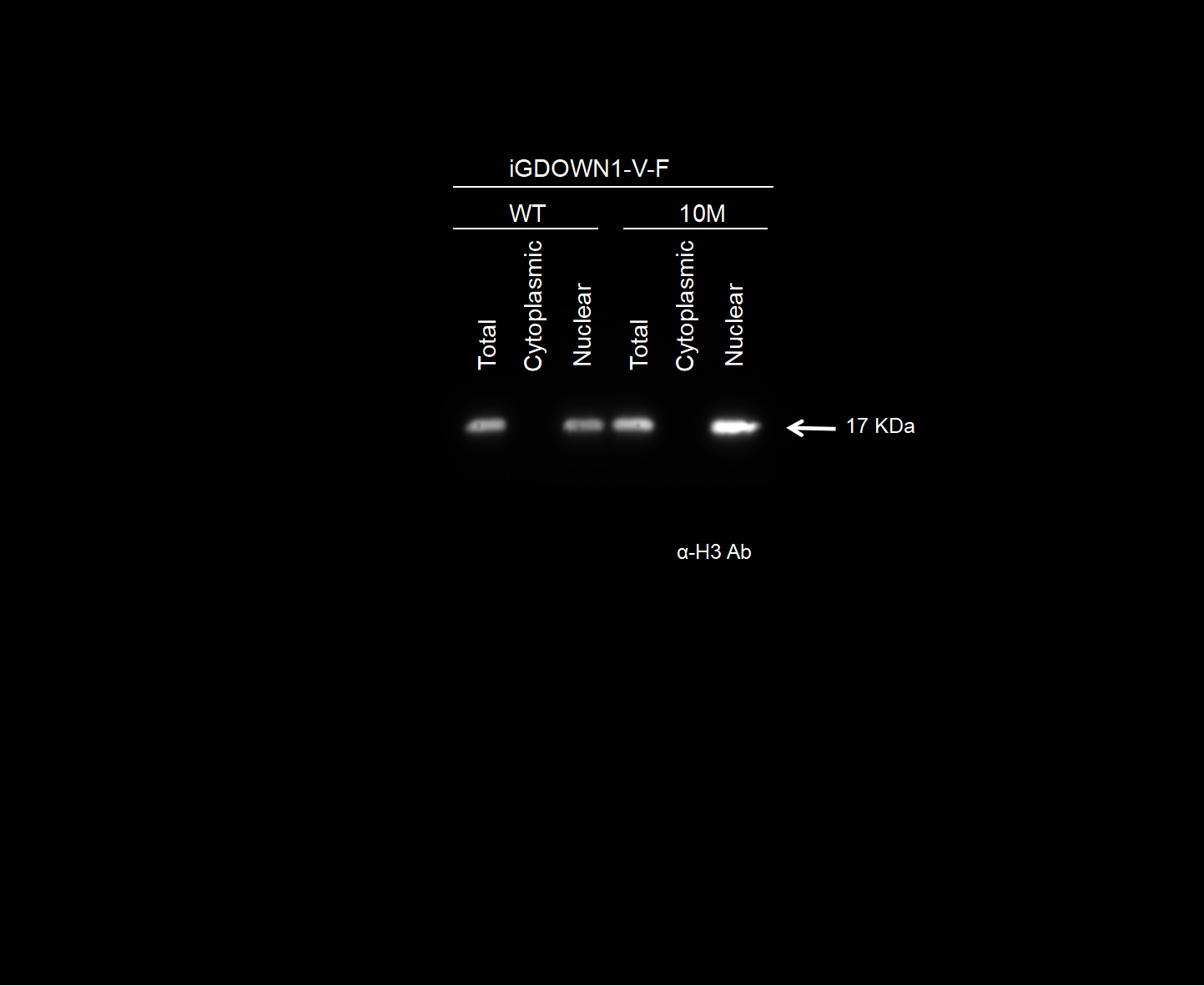

Supplement: Figure 6—figure supplement 1—source data 1. [file elife-79116-fig6-figsupp1-data1.zip › Figure 6-figure supplement 1-source data 1/+Label/Fig6-supple 1A-H3 antibody (For WT, 10M).Tif.tif]

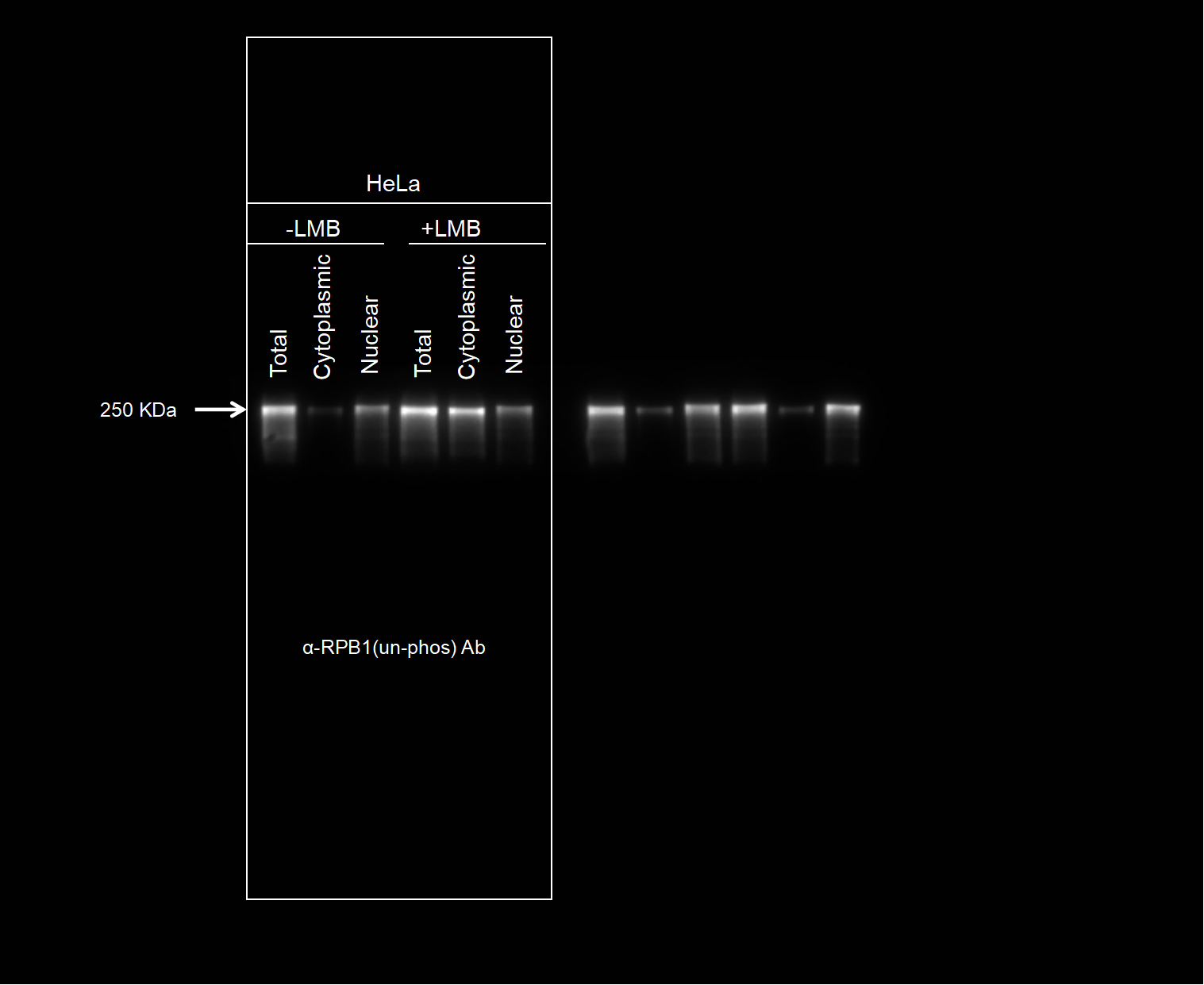

Supplement: Figure 6—figure supplement 1—source data 1. [file elife-79116-fig6-figsupp1-data1.zip › Figure 6-figure supplement 1-source data 1/+Label/Fig6-supple 1A-RPB1 (8WG16) antibody (For HeLa - or + LMB).Tif.tif]

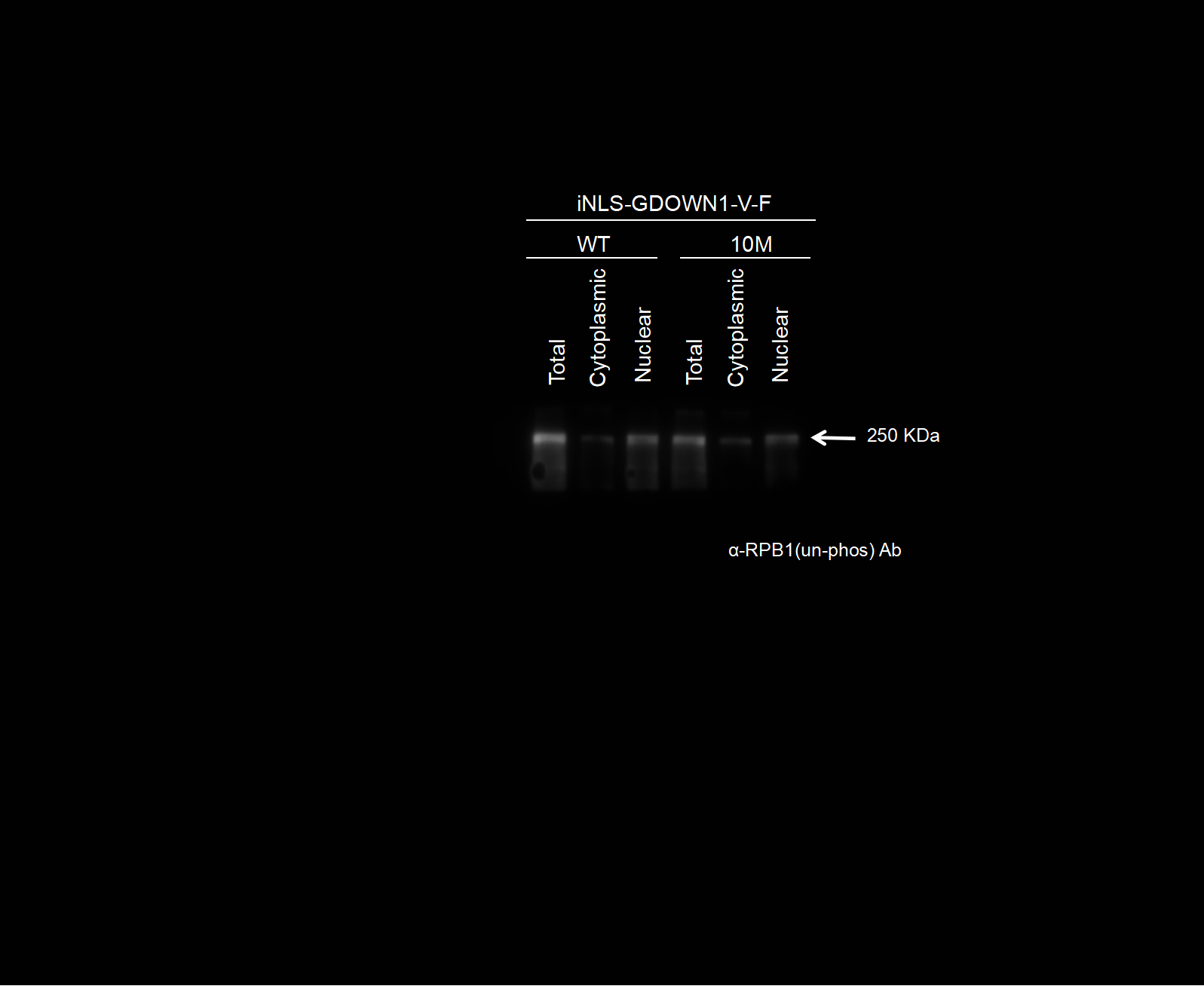

Supplement: Figure 6—figure supplement 1—source data 1. [file elife-79116-fig6-figsupp1-data1.zip › Figure 6-figure supplement 1-source data 1/+Label/Fig6-supple 1A-RPB1(8WG16) antibody (For NLS-WT, NLS-10M).Tif.tif]

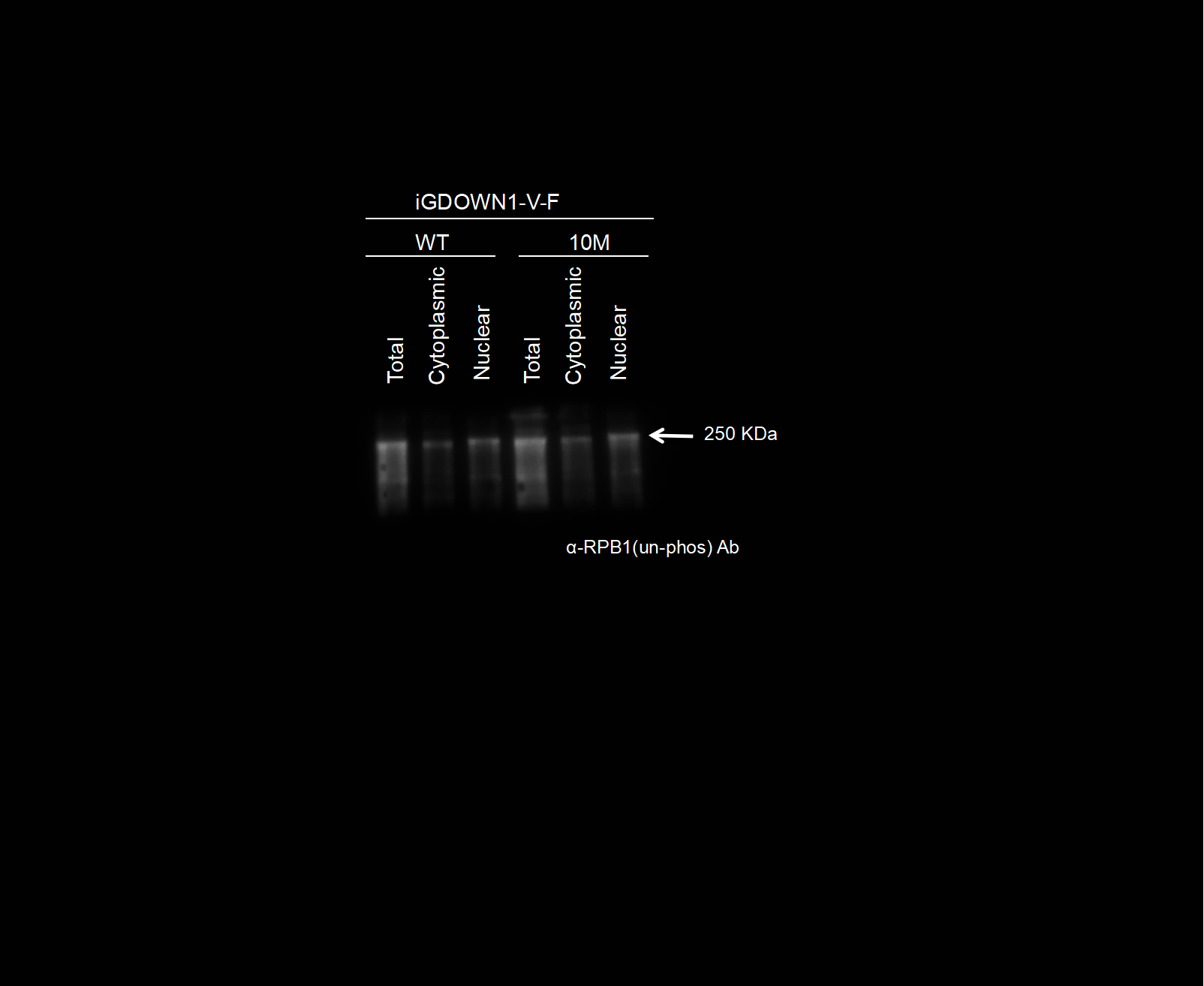

Supplement: Figure 6—figure supplement 1—source data 1. [file elife-79116-fig6-figsupp1-data1.zip › Figure 6-figure supplement 1-source data 1/+Label/Fig6-supple 1A-RPB1(8WG16) antibody (For WT, 10M).Tif.tif]

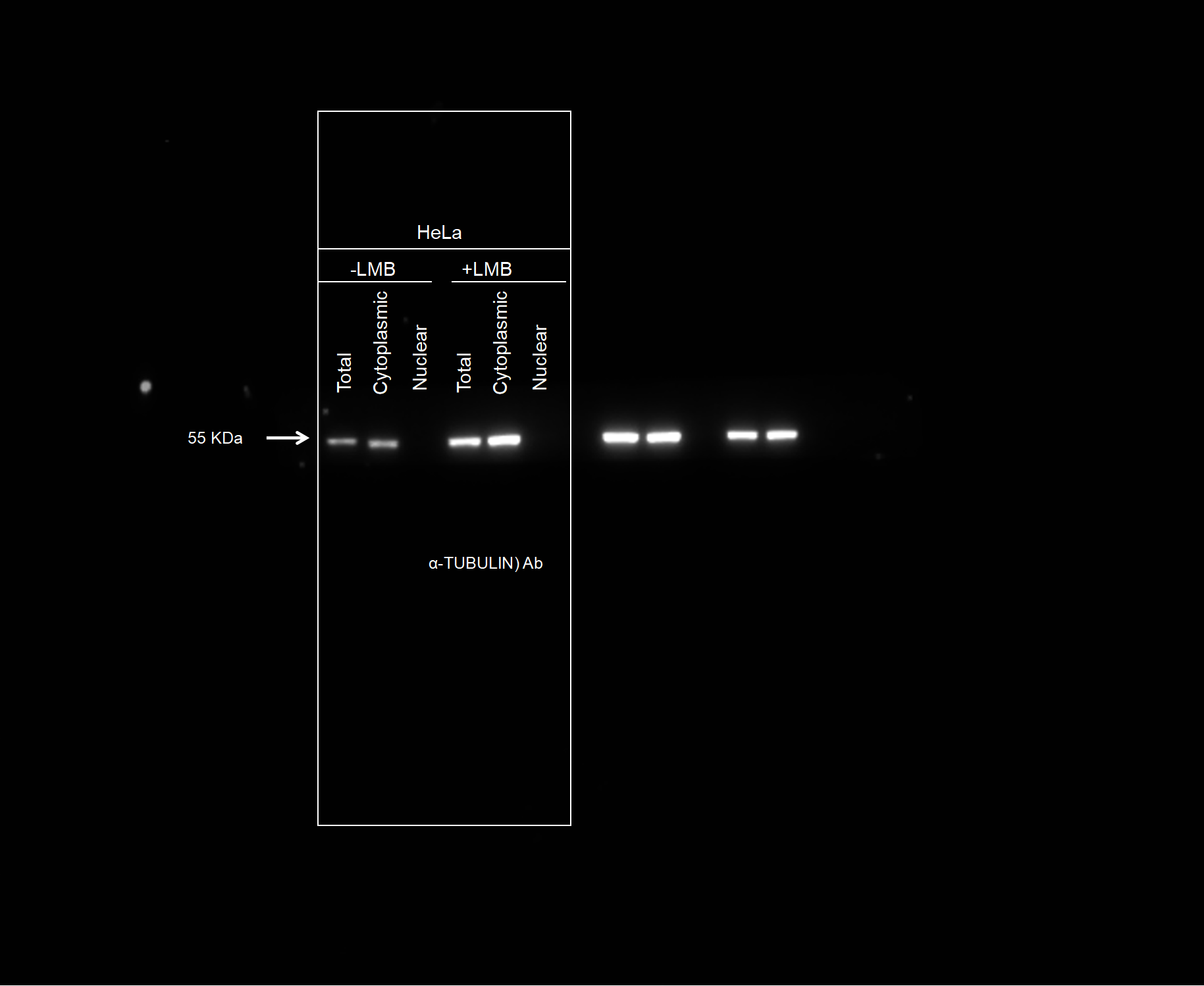

Supplement: Figure 6—figure supplement 1—source data 1. [file elife-79116-fig6-figsupp1-data1.zip › Figure 6-figure supplement 1-source data 1/+Label/Fig6-supple 1A-TUBULIN antibody (For HeLa - or + LMB).Tif.tif]

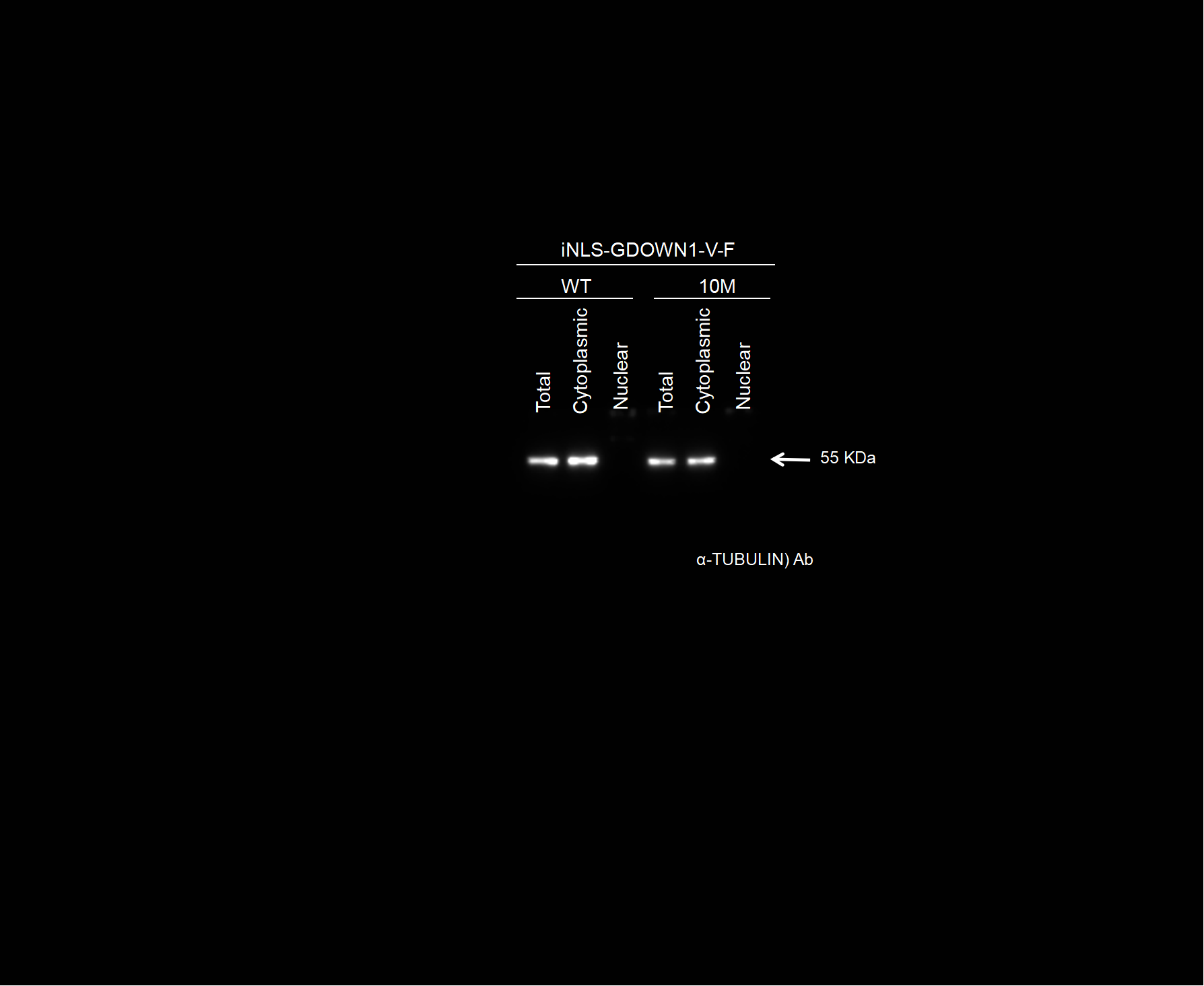

Supplement: Figure 6—figure supplement 1—source data 1. [file elife-79116-fig6-figsupp1-data1.zip › Figure 6-figure supplement 1-source data 1/+Label/Fig6-supple 1A-TUBULIN antibody (For NLS-WT, NLS-10M).Tif.tif]
