# Supplementary figures and images for: Overcoming the cytoplasmic retention of GDOWN1 modulates global transcription and facilitates stress adaptation (part 2 of 2)
Source: eLife. 2022 Dec 7;11:e79116. doi: 10.7554/eLife.79116 (PMC9728996; doi:10.7554/eLife.79116)

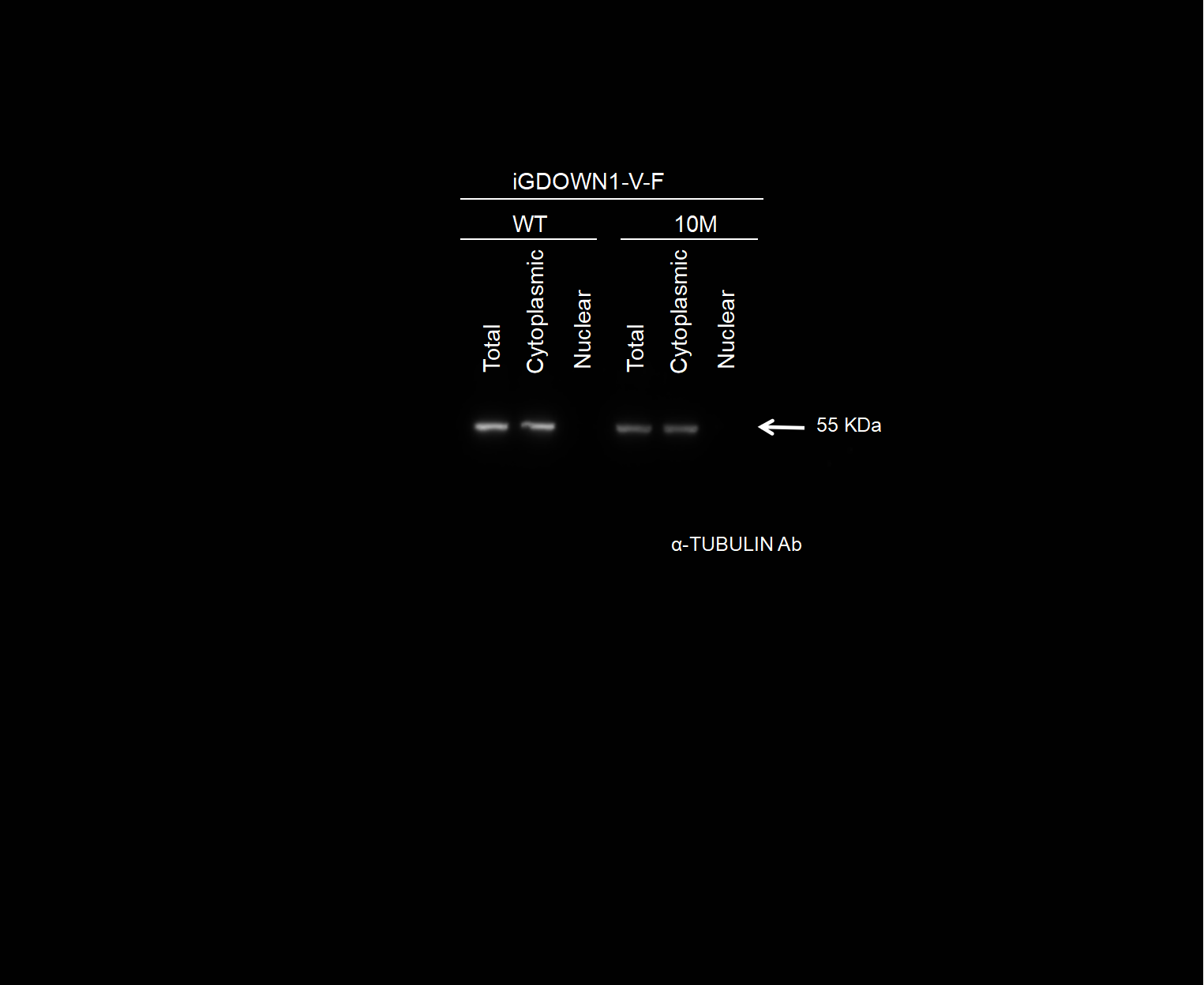

Supplement: Figure 6—figure supplement 1—source data 1. [file elife-79116-fig6-figsupp1-data1.zip › Figure 6-figure supplement 1-source data 1/+Label/Fig6-supple 1A-TUBULIN antibody (For WT, 10M).Tif.tif]

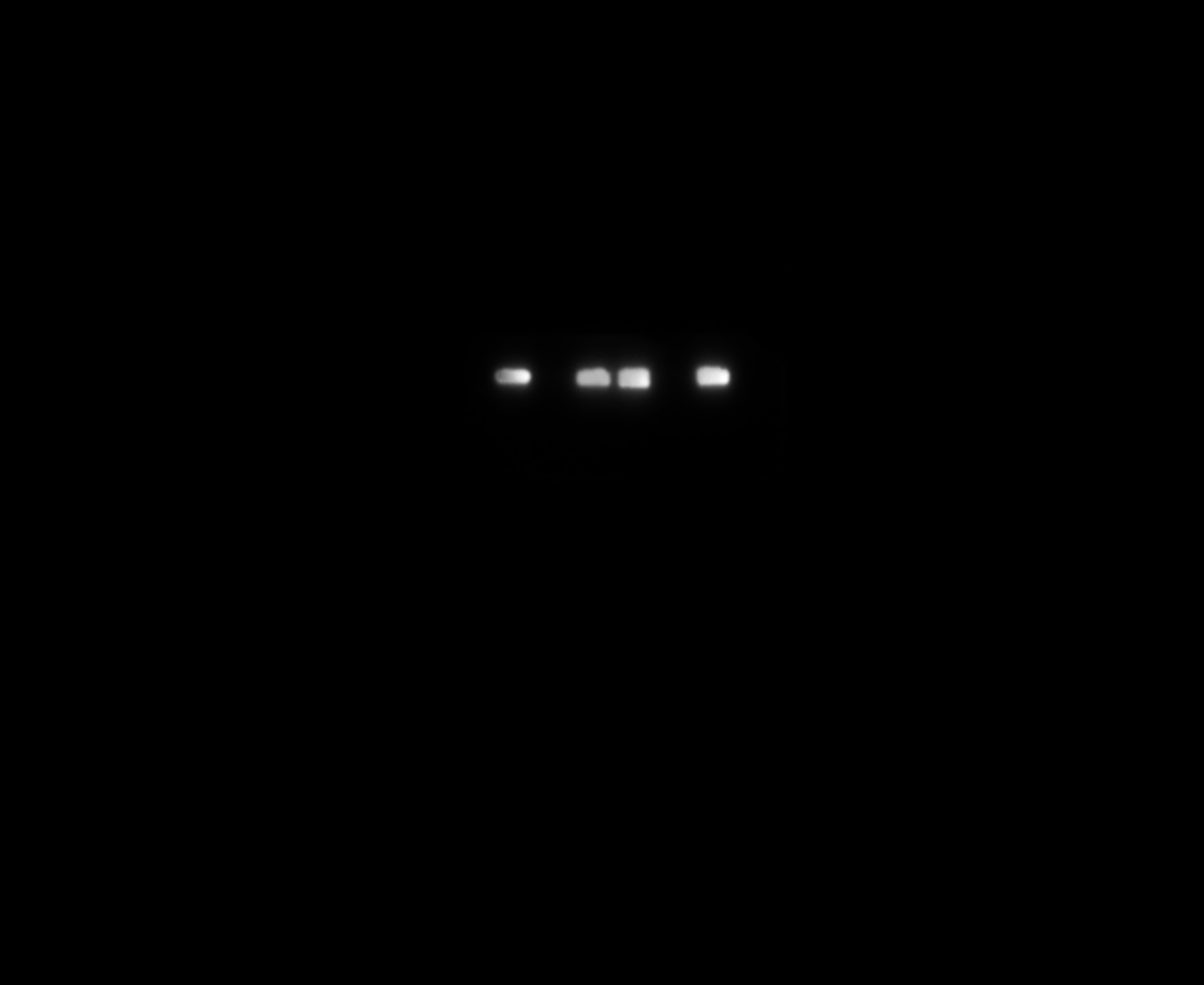

Supplement: Figure 6—figure supplement 1—source data 1. [file elife-79116-fig6-figsupp1-data1.zip › Figure 6-figure supplement 1-source data 1/Unedited/Fig6-Fig supplement 1A-H3 antibody (For NLS-WT, NLS-10M).Tif]

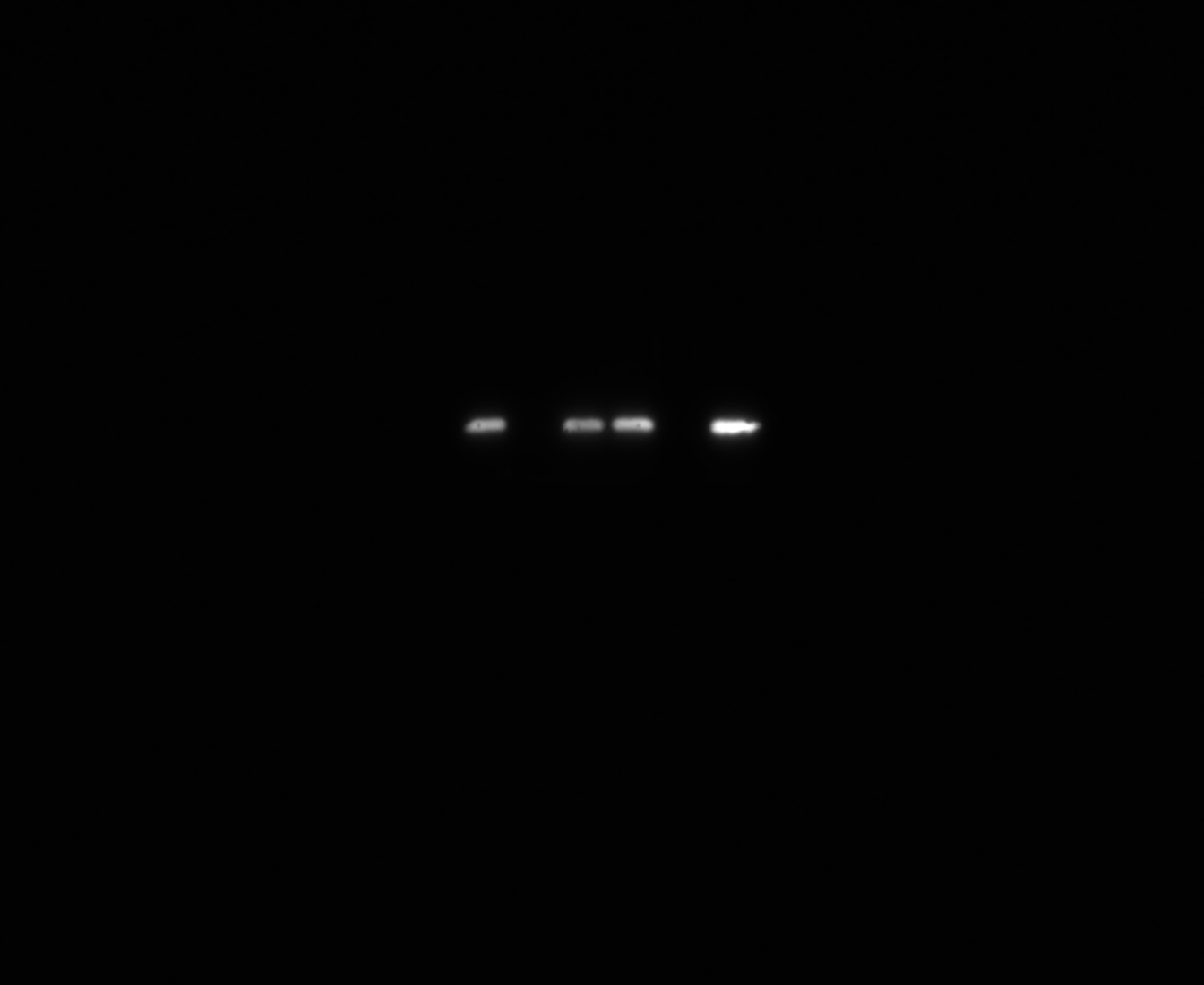

Supplement: Figure 6—figure supplement 1—source data 1. [file elife-79116-fig6-figsupp1-data1.zip › Figure 6-figure supplement 1-source data 1/Unedited/Fig6-Fig supplement 1A-H3 antibody (For WT, 10M).Tif]

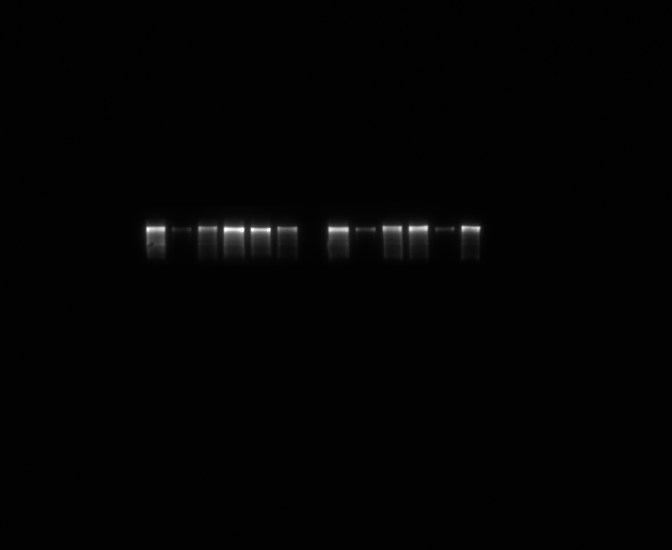

Supplement: Figure 6—figure supplement 1—source data 1. [file elife-79116-fig6-figsupp1-data1.zip › Figure 6-figure supplement 1-source data 1/Unedited/Fig6-supple 1A-RPB1 (8WG16) antibody (For HeLa - or + LMB).Tif]

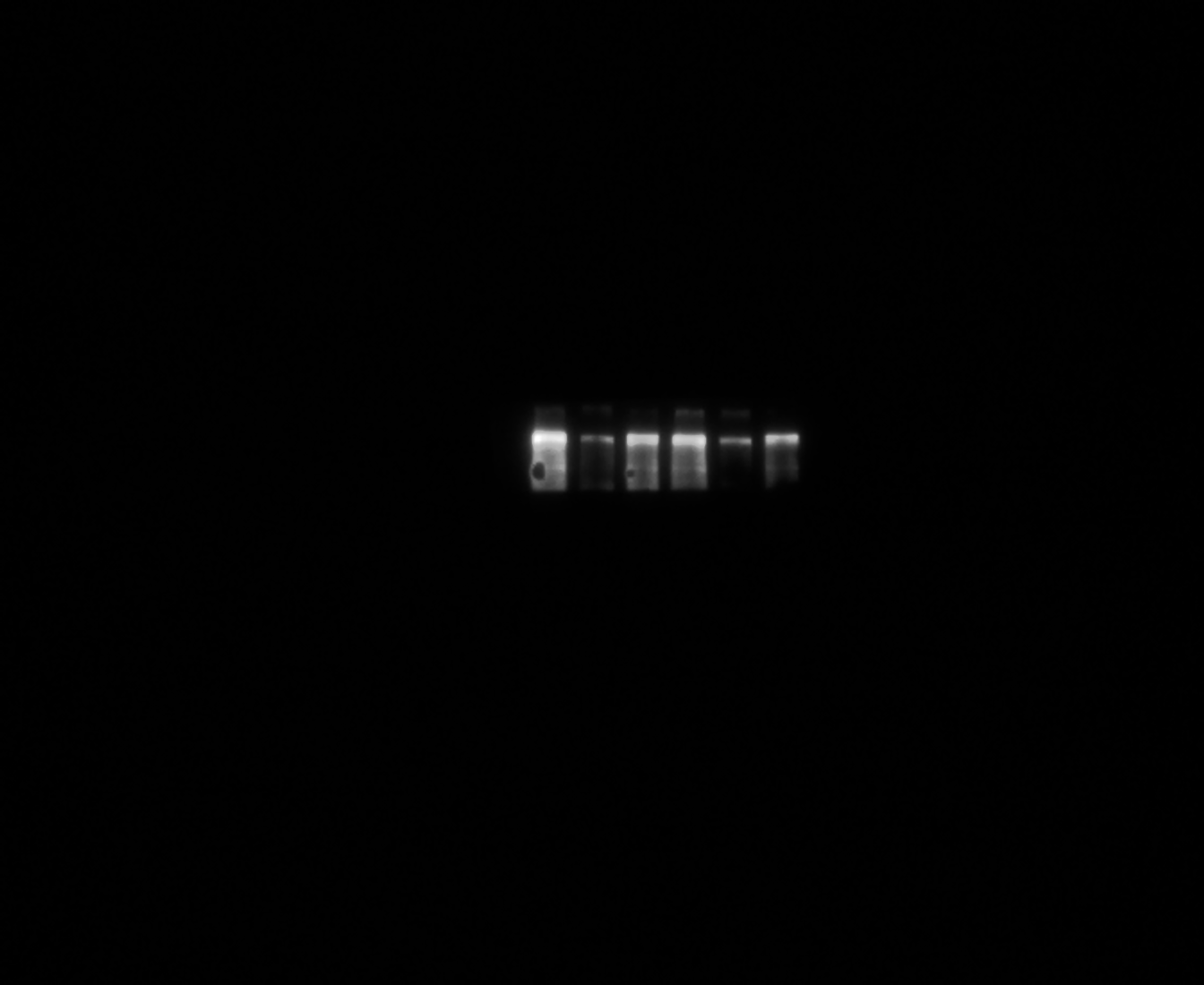

Supplement: Figure 6—figure supplement 1—source data 1. [file elife-79116-fig6-figsupp1-data1.zip › Figure 6-figure supplement 1-source data 1/Unedited/Fig6-supple 1A-RPB1(8WG16) antibody (For NLS-WT, NLS-10M).Tif]

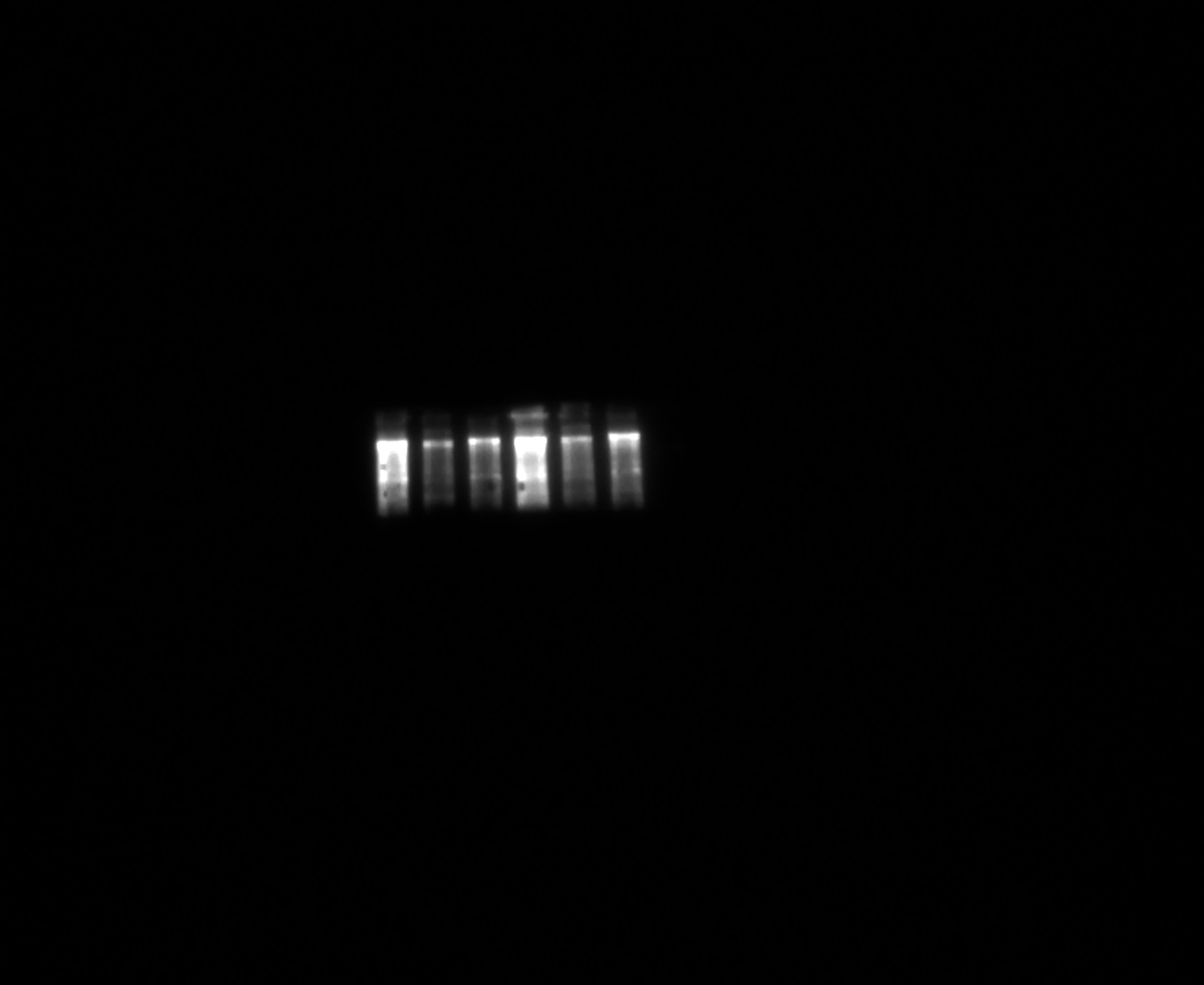

Supplement: Figure 6—figure supplement 1—source data 1. [file elife-79116-fig6-figsupp1-data1.zip › Figure 6-figure supplement 1-source data 1/Unedited/Fig6-supple 1A-RPB1(8WG16) antibody (For WT, 10M).Tif]

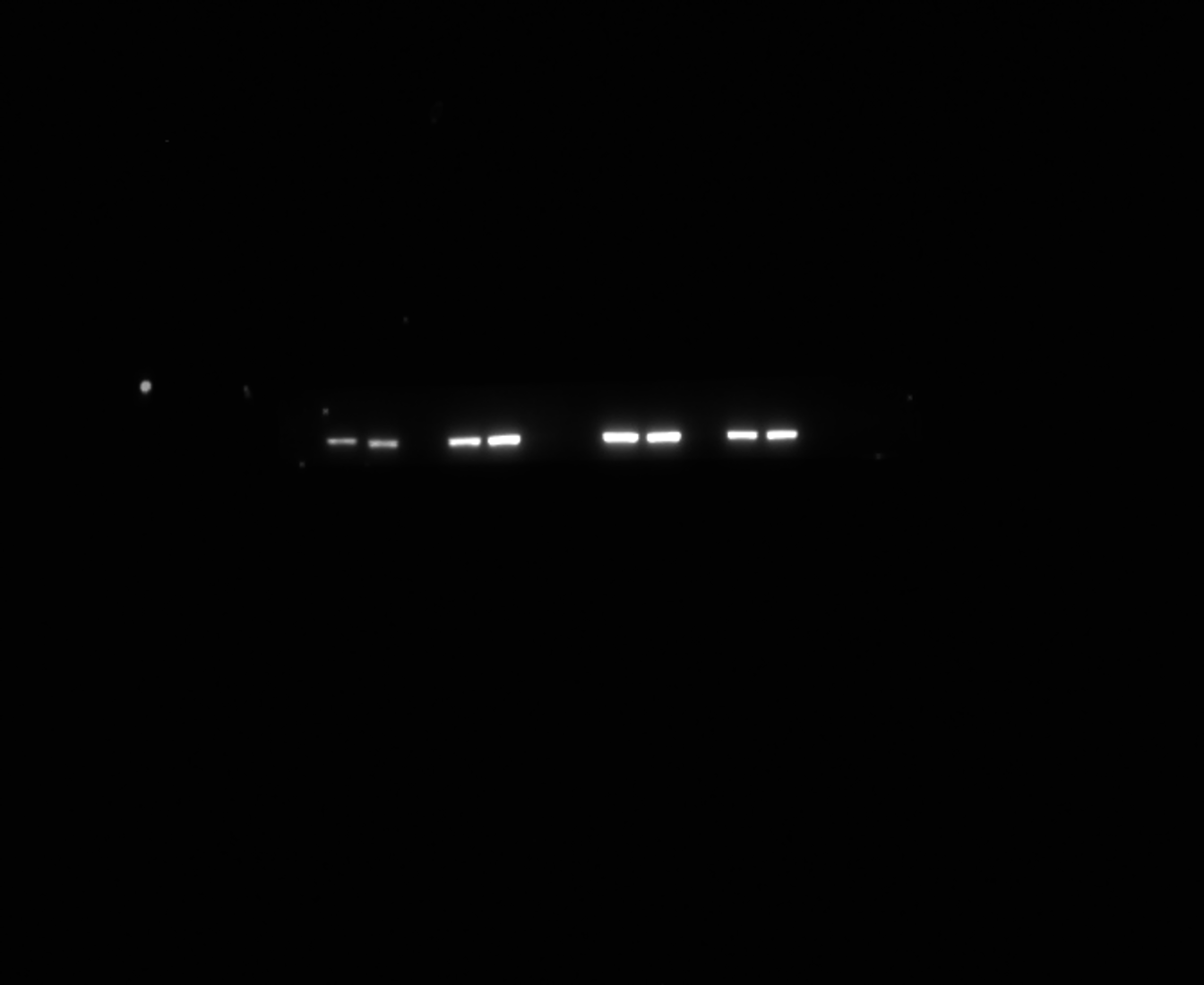

Supplement: Figure 6—figure supplement 1—source data 1. [file elife-79116-fig6-figsupp1-data1.zip › Figure 6-figure supplement 1-source data 1/Unedited/Fig6-supple 1A-TUBULIN antibody (For HeLa - or + LMB).Tif]

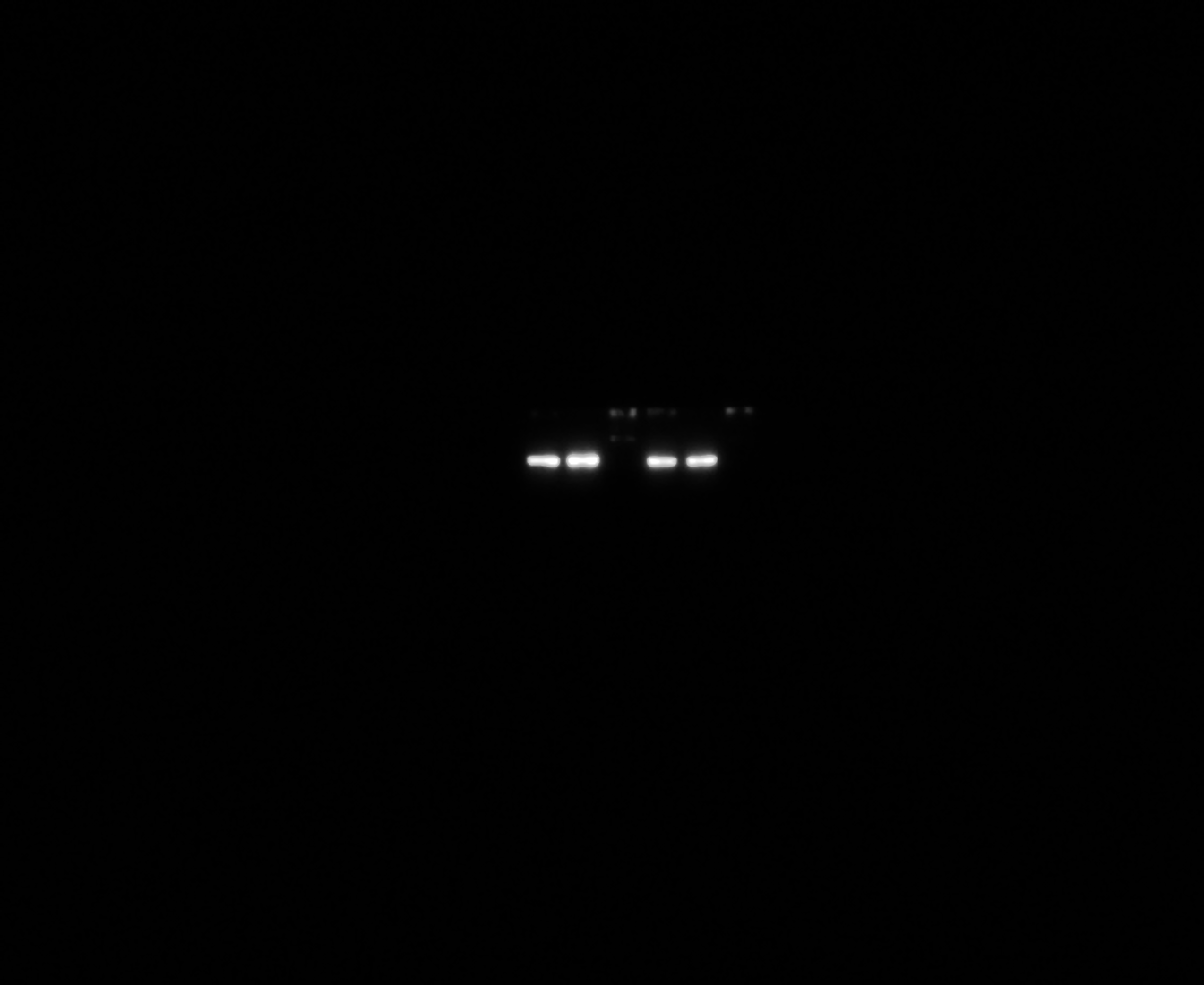

Supplement: Figure 6—figure supplement 1—source data 1. [file elife-79116-fig6-figsupp1-data1.zip › Figure 6-figure supplement 1-source data 1/Unedited/Fig6-supple 1A-TUBULIN antibody (For NLS-WT, NLS-10M).Tif]

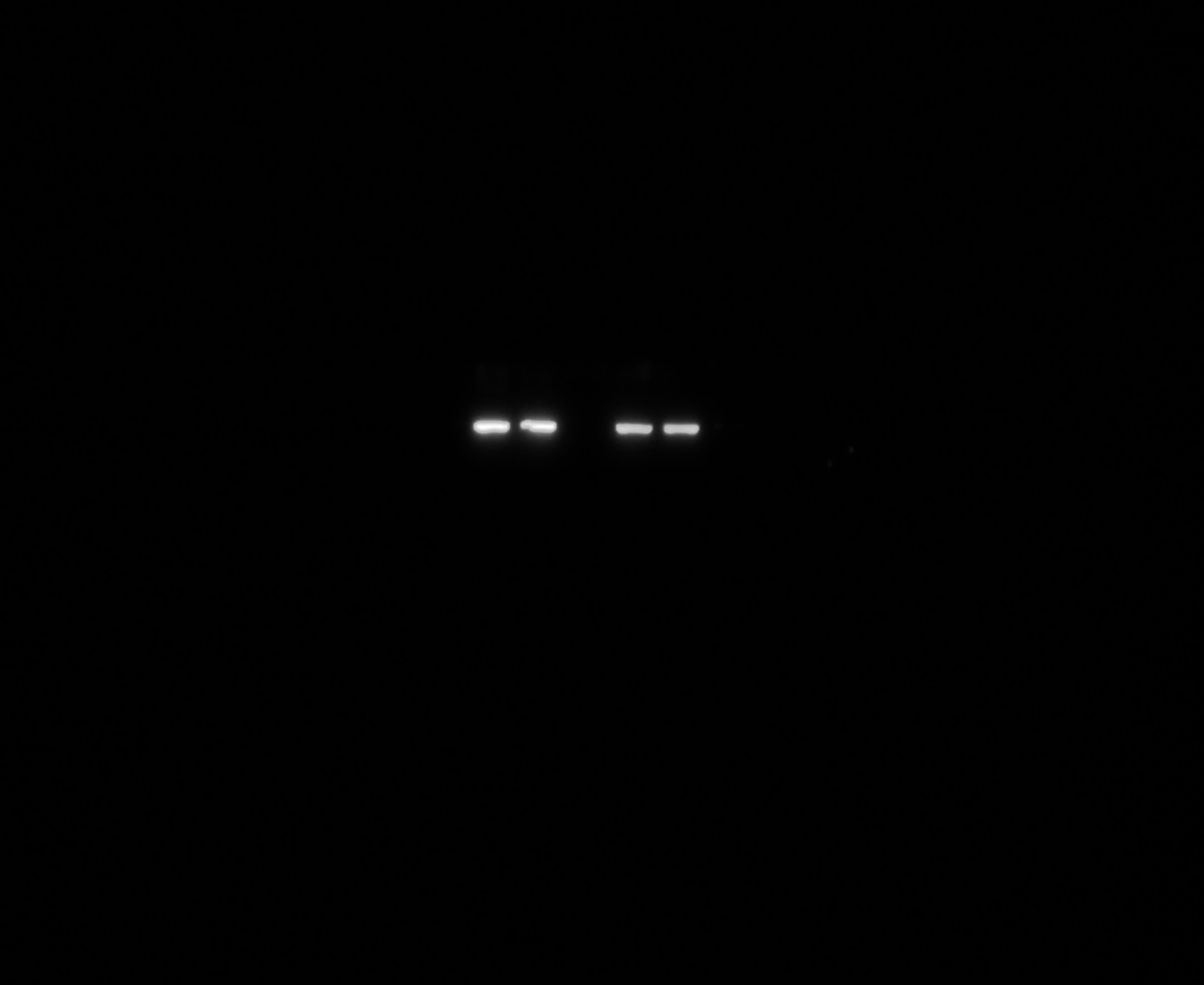

Supplement: Figure 6—figure supplement 1—source data 1. [file elife-79116-fig6-figsupp1-data1.zip › Figure 6-figure supplement 1-source data 1/Unedited/Fig6-supple 1A-TUBULIN antibody (For WT, 10M).Tif]

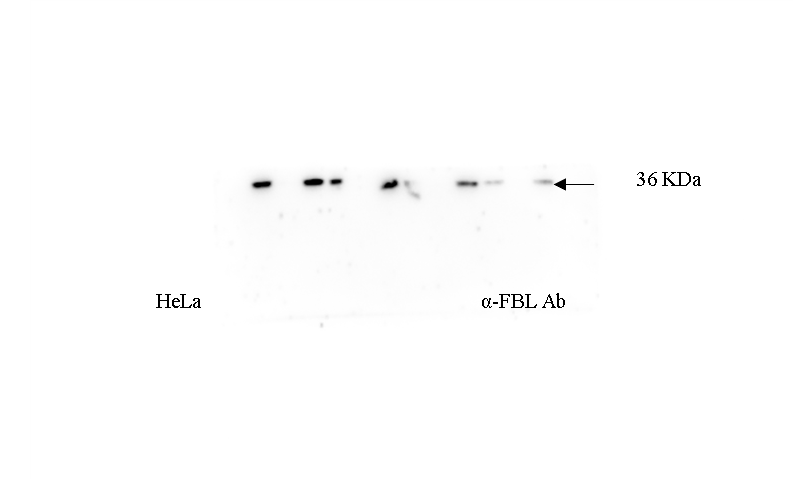

Supplement: Figure 7—source data 1. [file elife-79116-fig7-data1.zip › Figure 7-source data 1/+Label/Fig 7A-FBL antibody (For HeLa mock, 0.01 mM, 0.03 mM, 0.1 mM NaAsO2, 6 h ).tif]

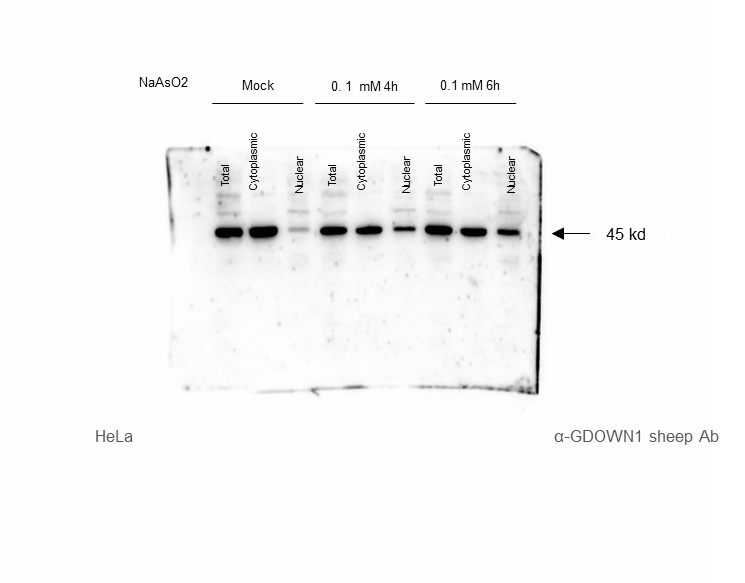

Supplement: Figure 7—source data 1. [file elife-79116-fig7-data1.zip › Figure 7-source data 1/+Label/Fig 7A-GDOWN1 antibody (For HeLa mock, 0.1 mM NaAsO2, 4 h, 6 h).tif]

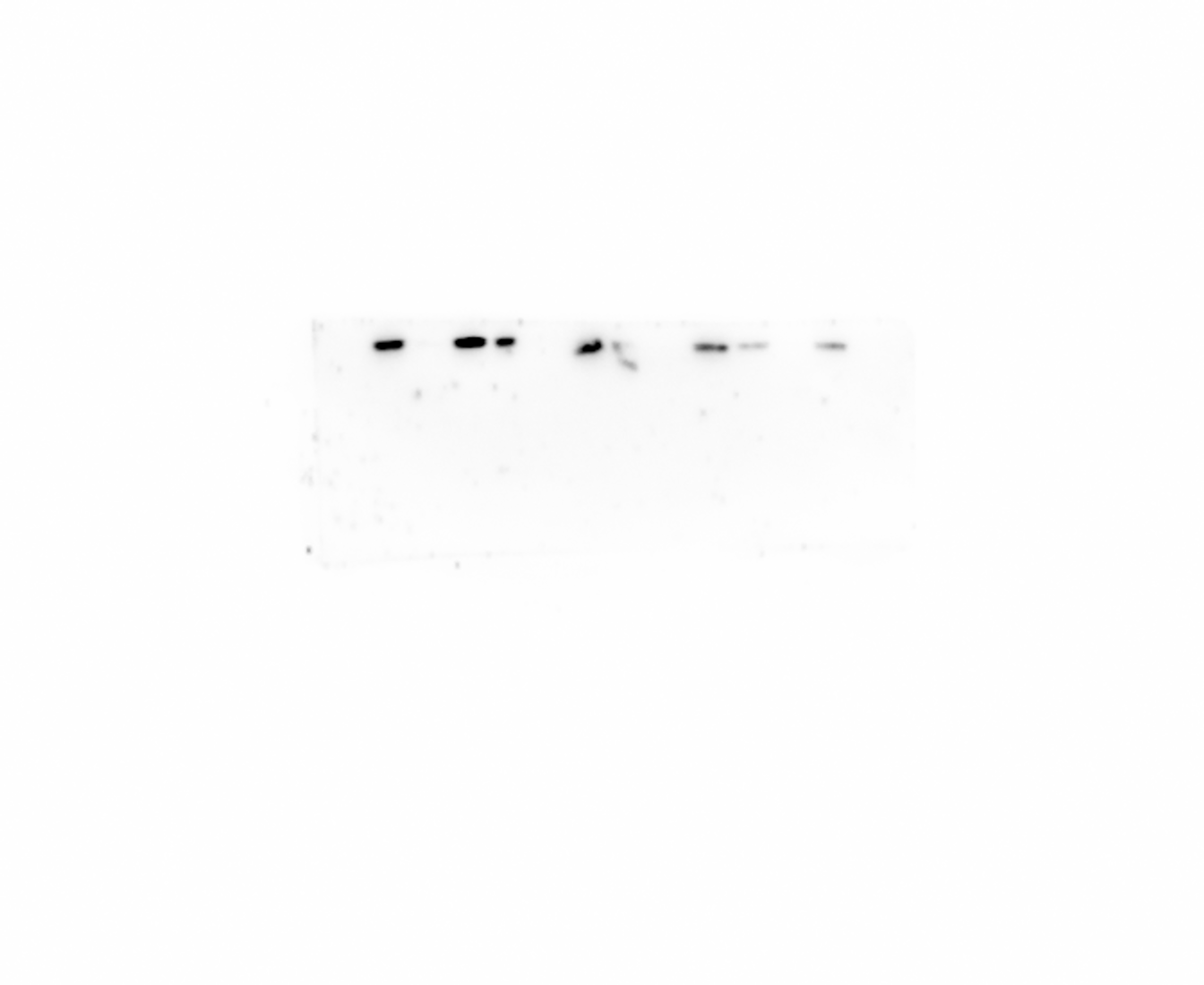

Supplement: Figure 7—source data 1. [file elife-79116-fig7-data1.zip › Figure 7-source data 1/Unedited/Fig 7A-FBL antibody (For HeLa mock, 0.01 mM, 0.03 mM, 0.1 mM NaAsO2, 6 h ).Tif]

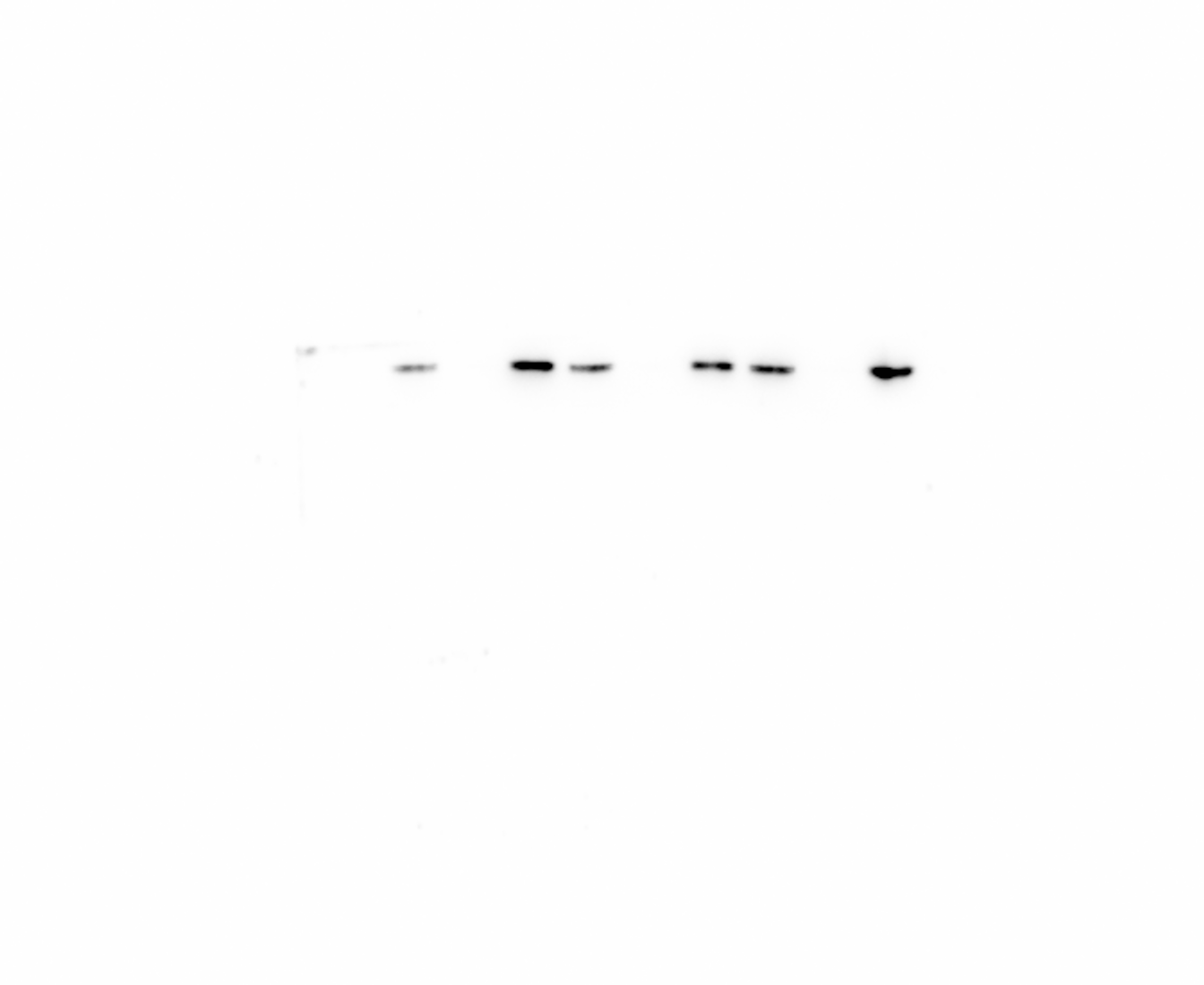

Supplement: Figure 7—source data 1. [file elife-79116-fig7-data1.zip › Figure 7-source data 1/Unedited/Fig 7A-FBL antibody (For HeLa mock, 0.1 mM NaAsO2 4 h 6 h).Tif.Tif]

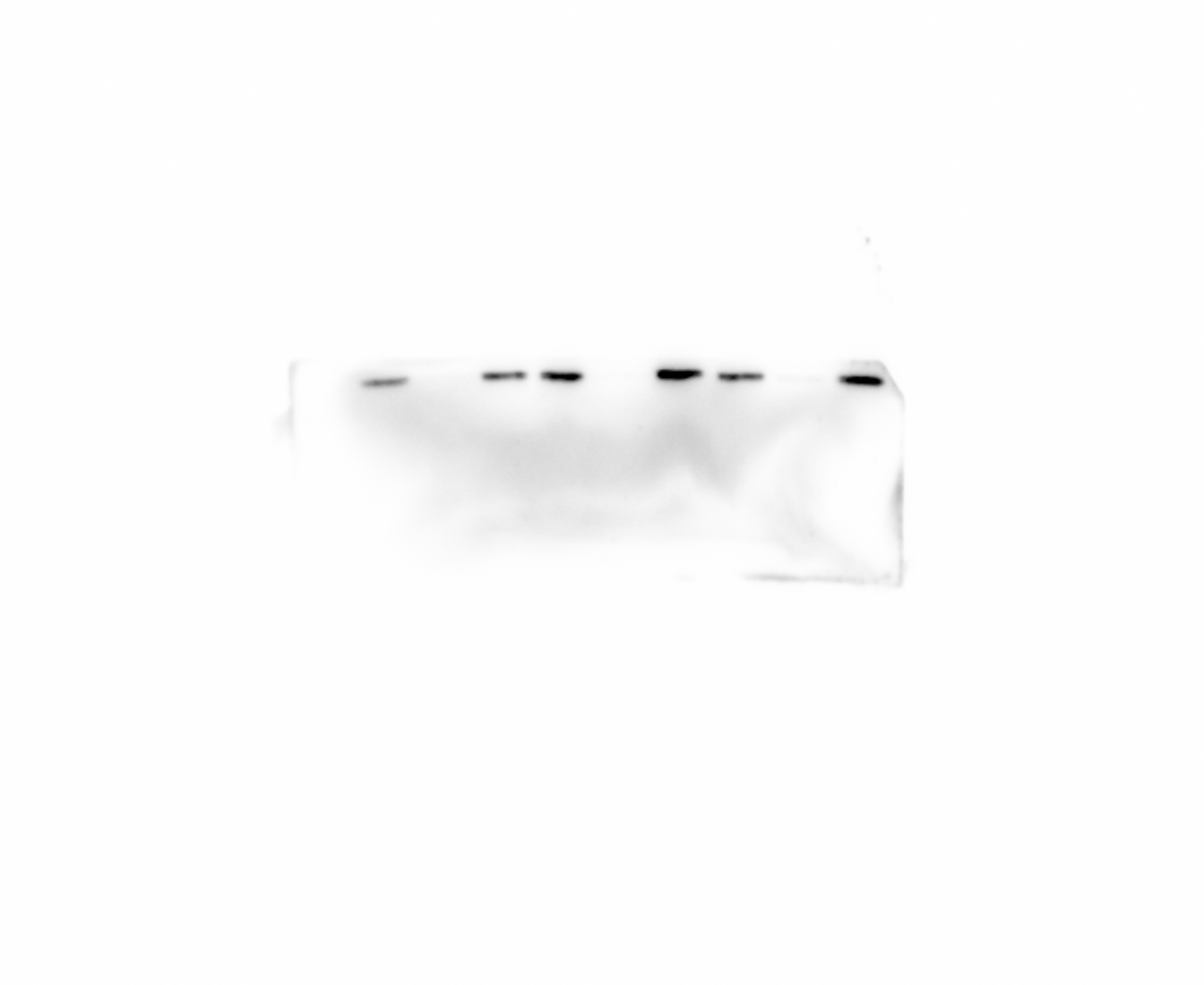

Supplement: Figure 7—source data 1. [file elife-79116-fig7-data1.zip › Figure 7-source data 1/Unedited/Fig 7A-FBL antibody (For HeLa mock, 0.1 mM NaAsO2. 4 h, 6 h and recover 24 h).Tif]

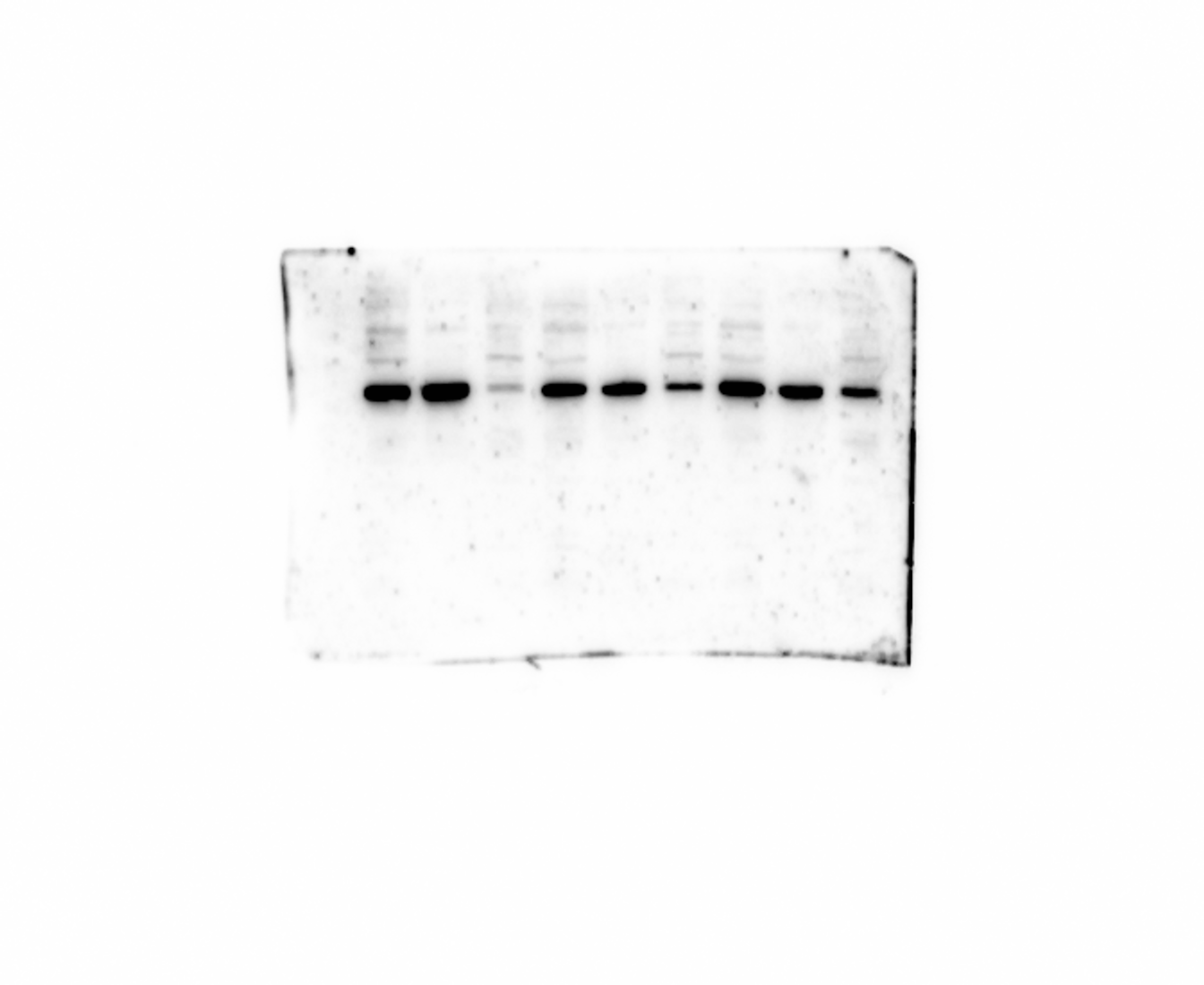

Supplement: Figure 7—source data 1. [file elife-79116-fig7-data1.zip › Figure 7-source data 1/Unedited/Fig 7A-GDOWN1 antibody (For HeLa mock, 0.1 mM NaAsO2, 4 h, 6 h).Tif]

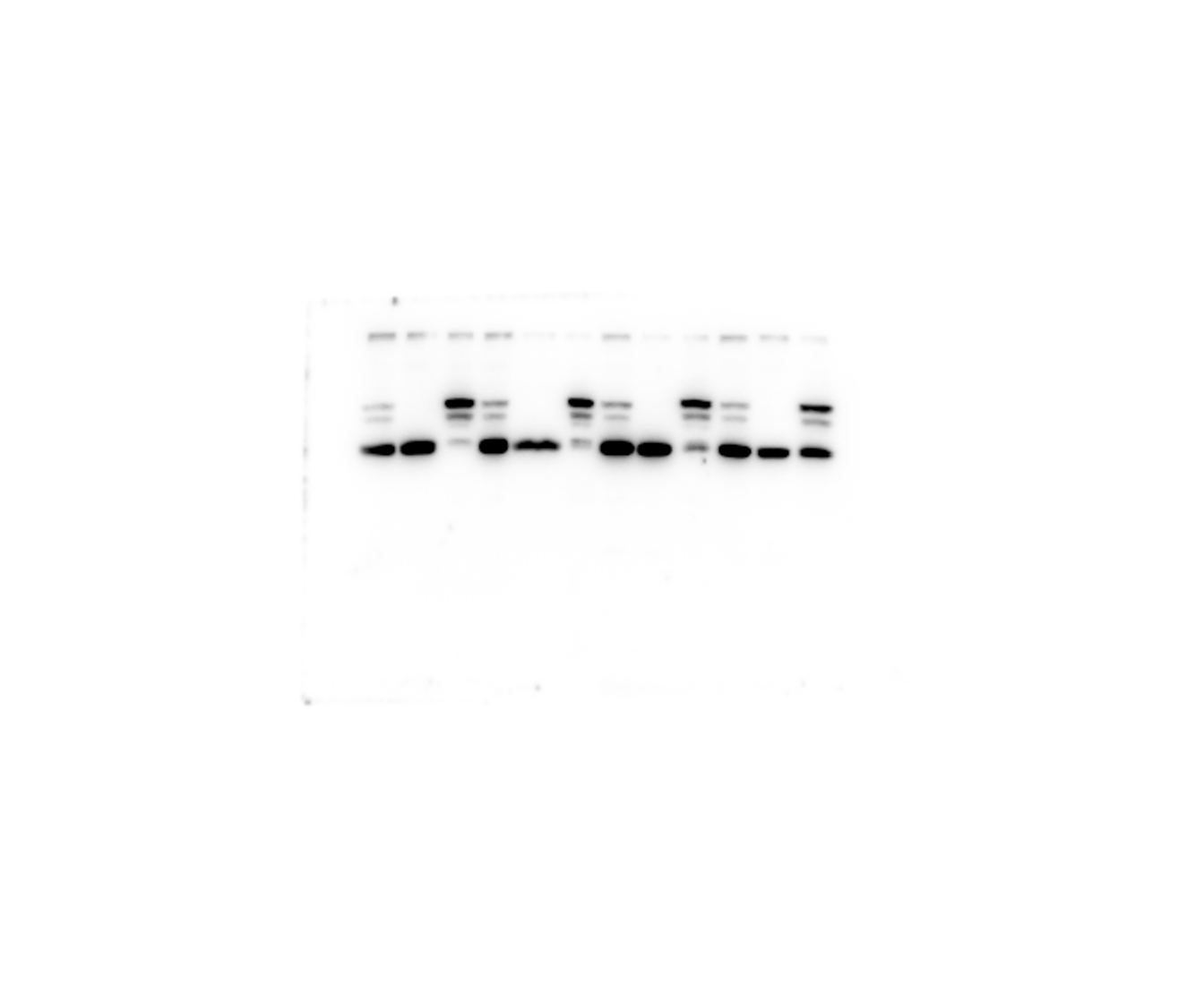

Supplement: Figure 7—source data 1. [file elife-79116-fig7-data1.zip › Figure 7-source data 1/Unedited/Fig 7A-GDOWN1 antibody (For HeLa mock, 0.01 mM, 0.03mM, 0.1mM NaAsO2, 6 h ).Tif]

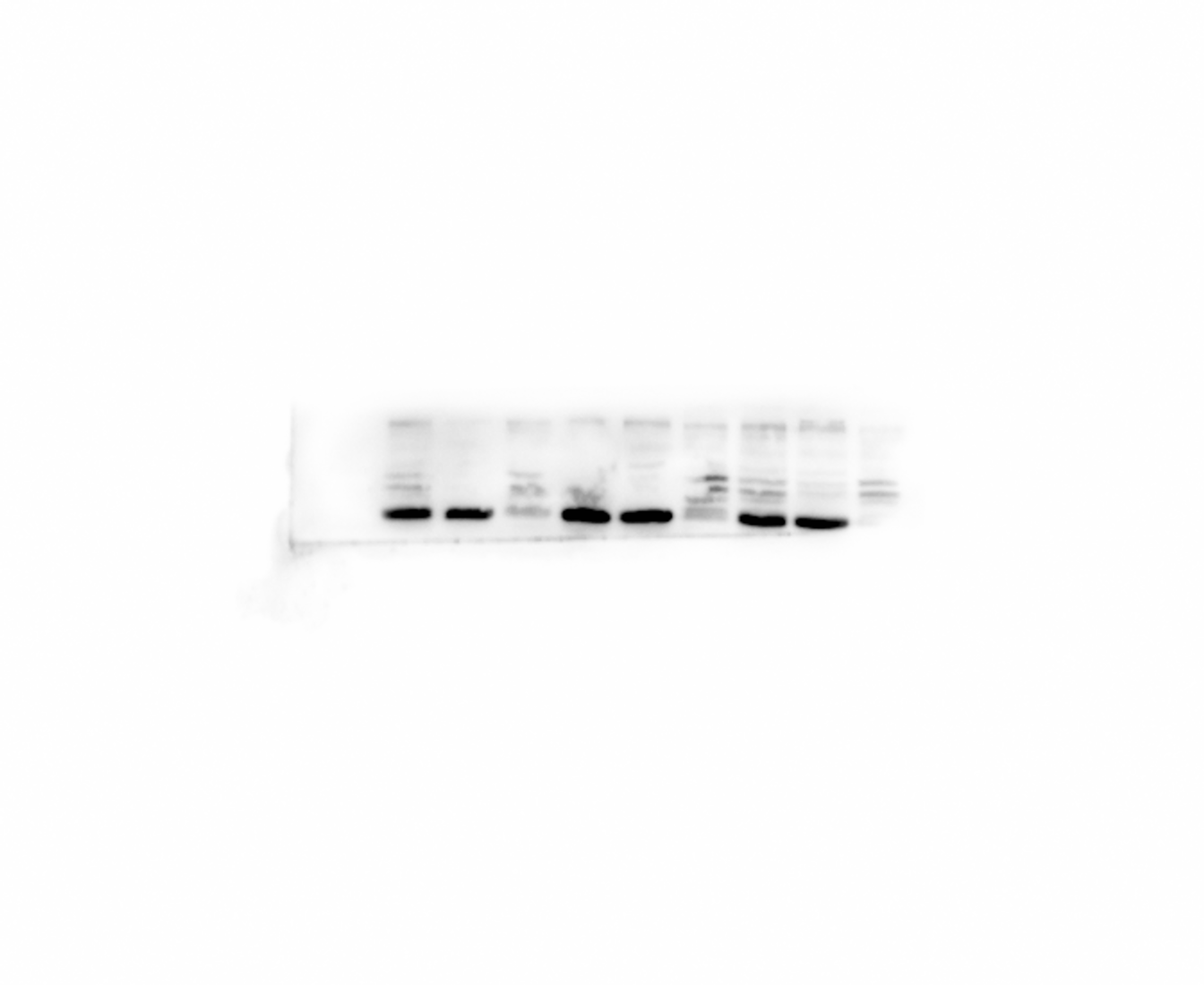

Supplement: Figure 7—source data 1. [file elife-79116-fig7-data1.zip › Figure 7-source data 1/Unedited/Fig 7A-GDOWN1 antibody(For HeLa mock, 0.1 mM NaAsO2, 4 h, 6 h and recover 24 h).Tif]

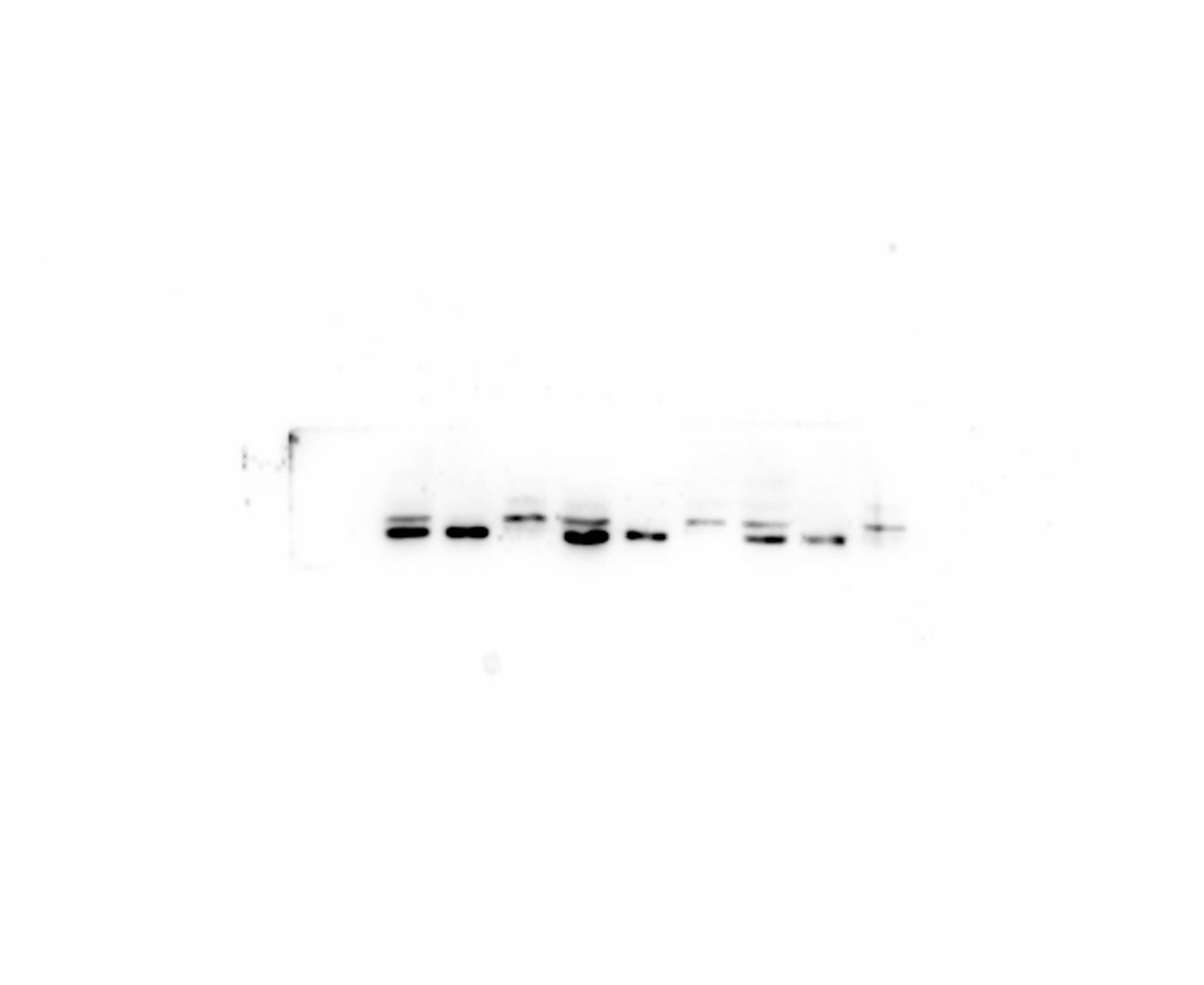

Supplement: Figure 7—source data 1. [file elife-79116-fig7-data1.zip › Figure 7-source data 1/Unedited/Fig 7A-TUBULIN antibody (For HeLa mock, 0.1mM NaAsO2 4 h 6 h).Tif]

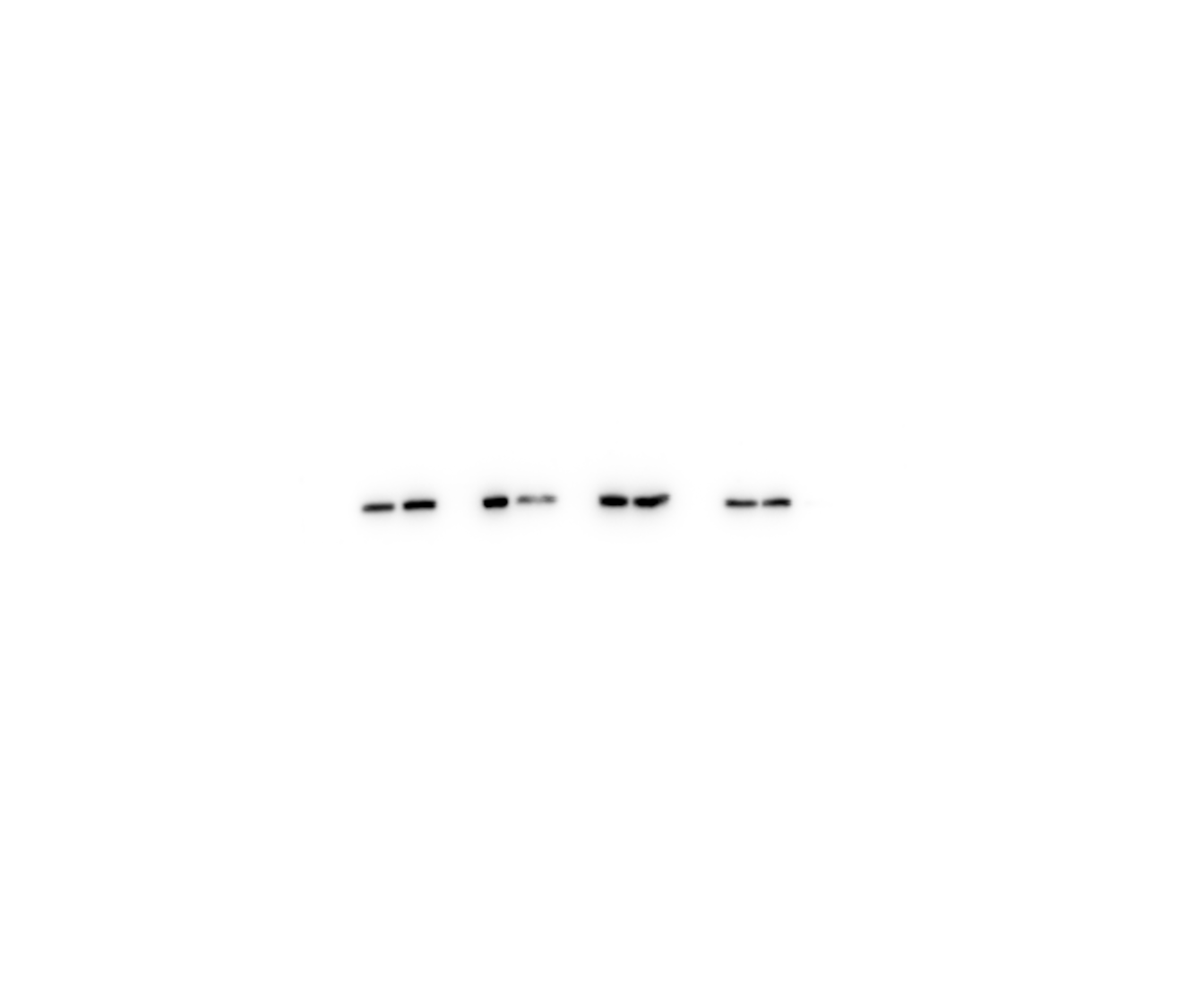

Supplement: Figure 7—source data 1. [file elife-79116-fig7-data1.zip › Figure 7-source data 1/Unedited/Fig 7A-TUBULIN antibody (For HeLa mock, 0.01 mM, 0.03 mM, 0.1 mM NaAsO2, 6 h ).Tif]

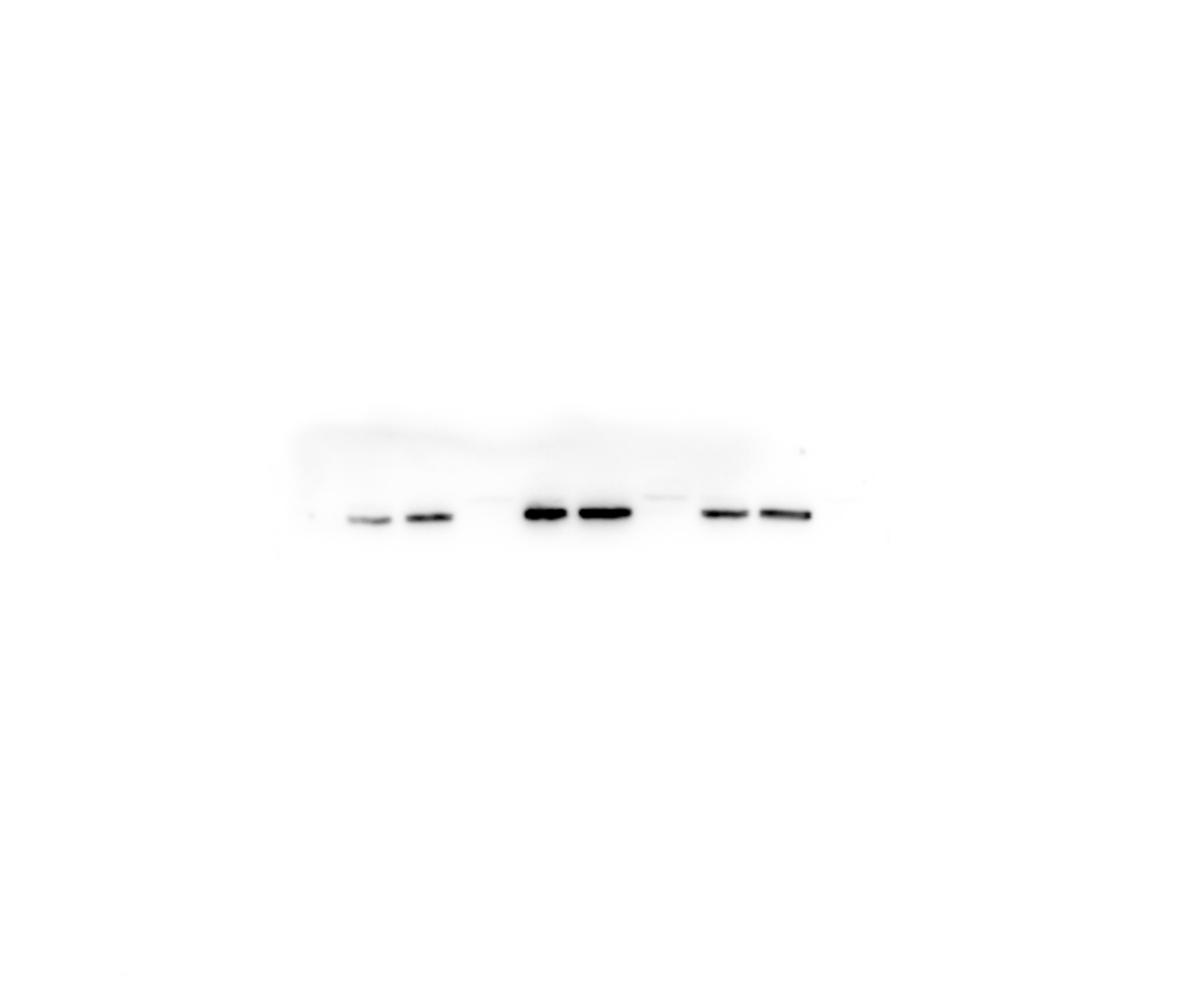

Supplement: Figure 7—source data 1. [file elife-79116-fig7-data1.zip › Figure 7-source data 1/Unedited/Fig 7A-TUBULIN antibody(For HeLa mock, 0.1 mM NaAsO2, 4 h 6 h and recover 24 h).Tif]

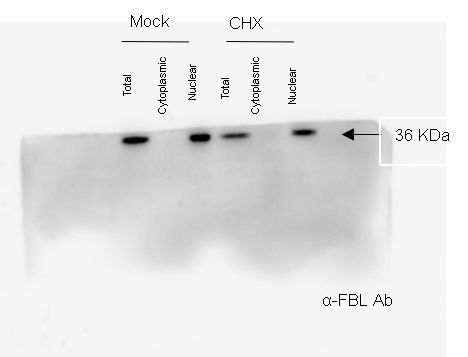

Supplement: Figure 7—figure supplement 1—source data 1. [file elife-79116-fig7-figsupp1-data1.zip › Figure 7-figure supplement 1-source data 1/+Label/Fig7-Fig supplement 1A -FBL antibody(for HeLa mock +250mg per ml 12 h CHX ).tif]

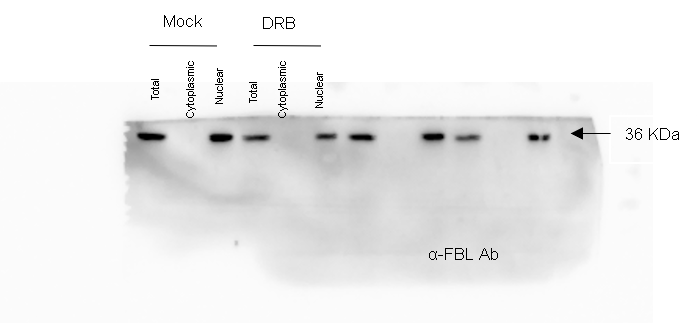

Supplement: Figure 7—figure supplement 1—source data 1. [file elife-79116-fig7-figsupp1-data1.zip › Figure 7-figure supplement 1-source data 1/+Label/Fig7-Fig supplement 1A -FBL antibody(for HeLa mock +500uM 6 h DRB ).tif]

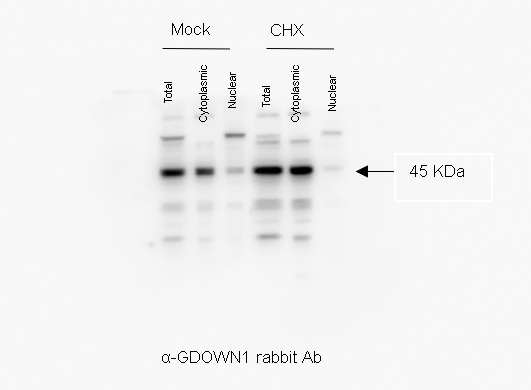

Supplement: Figure 7—figure supplement 1—source data 1. [file elife-79116-fig7-figsupp1-data1.zip › Figure 7-figure supplement 1-source data 1/+Label/Fig7-Fig supplement 1A -GDOWN1 antibody(for HeLa mock +250mg per ml 12 h CHX ).tif]

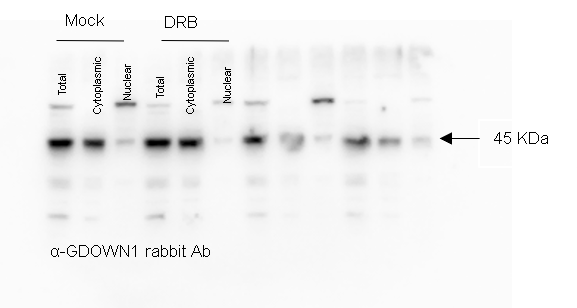

Supplement: Figure 7—figure supplement 1—source data 1. [file elife-79116-fig7-figsupp1-data1.zip › Figure 7-figure supplement 1-source data 1/+Label/Fig7-Fig supplement 1A -GDOWN1 antibody(for HeLa mock +500uM 6 h DRB ).tif]

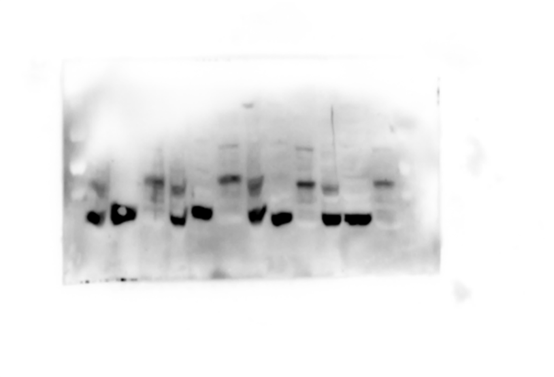

Supplement: Figure 7—figure supplement 1—source data 1. [file elife-79116-fig7-figsupp1-data1.zip › Figure 7-figure supplement 1-source data 1/+Label/Fig7-Fig supplement 1A -GDOWN1 antibody(for HeLa mock +CPT+Doxorubicin ).tif]

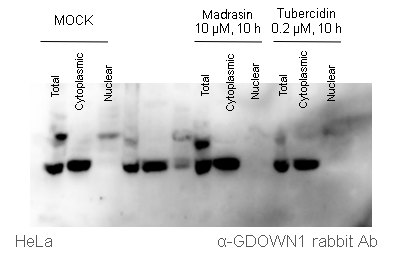

Supplement: Figure 7—figure supplement 1—source data 1. [file elife-79116-fig7-figsupp1-data1.zip › Figure 7-figure supplement 1-source data 1/+Label/Fig7-Fig supplement 1A -GDOWN1 antibody(for HeLa mock +Madrasin+Tubercidin ).tif]

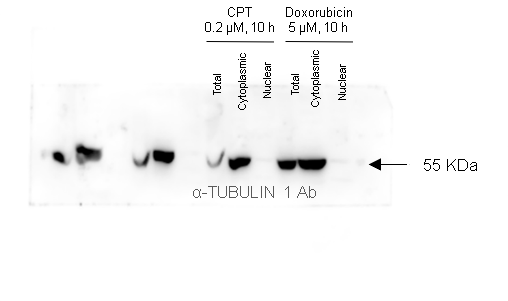

Supplement: Figure 7—figure supplement 1—source data 1. [file elife-79116-fig7-figsupp1-data1.zip › Figure 7-figure supplement 1-source data 1/+Label/Fig7-Fig supplement 1A -TUBULIN antibody(for HeLa mock +CPT+Doxorubicin ).tif]

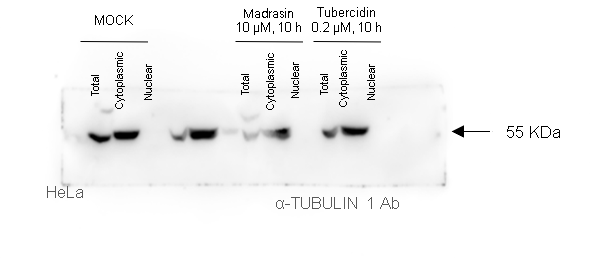

Supplement: Figure 7—figure supplement 1—source data 1. [file elife-79116-fig7-figsupp1-data1.zip › Figure 7-figure supplement 1-source data 1/+Label/Fig7-Fig supplement 1A -TUBULIN antibody(for HeLa mock +Madrasin+Tubercidin ).tif]

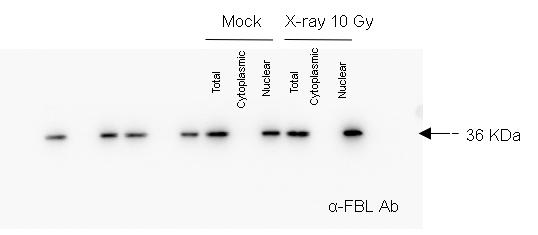

Supplement: Figure 7—figure supplement 1—source data 1. [file elife-79116-fig7-figsupp1-data1.zip › Figure 7-figure supplement 1-source data 1/+Label/Fig7-Fig supplement 1A-FBL antibody(for HeLa mock +X-ray 10Gy ).tif]

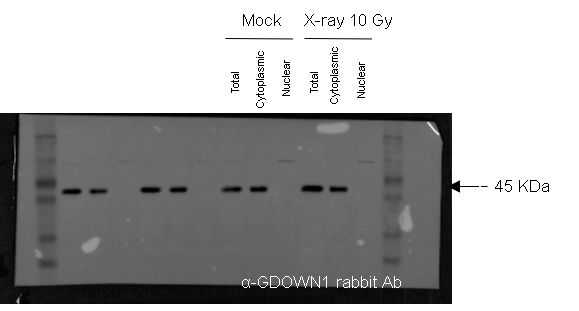

Supplement: Figure 7—figure supplement 1—source data 1. [file elife-79116-fig7-figsupp1-data1.zip › Figure 7-figure supplement 1-source data 1/+Label/Fig7-Fig supplement 1A-GDOWN1 antibody(for HeLa mock +X-ray 10Gy ).tif]

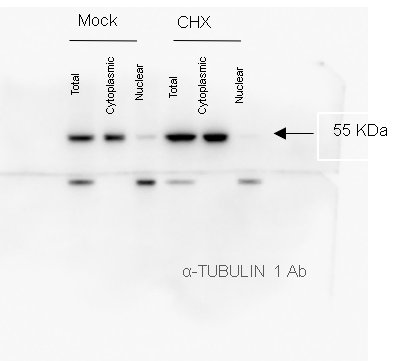

Supplement: Figure 7—figure supplement 1—source data 1. [file elife-79116-fig7-figsupp1-data1.zip › Figure 7-figure supplement 1-source data 1/+Label/Fig7-Fig supplement 1A-TUBULIN antibody(for HeLa mock +250mg per ml 12 h CHX ).tif]

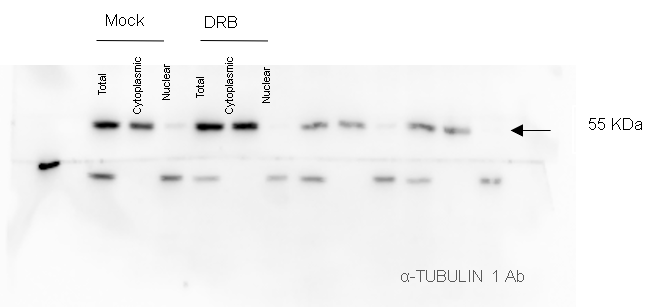

Supplement: Figure 7—figure supplement 1—source data 1. [file elife-79116-fig7-figsupp1-data1.zip › Figure 7-figure supplement 1-source data 1/+Label/Fig7-Fig supplement 1A-TUBULIN antibody(for HeLa mock +500uM 6 h DRB ).tif]

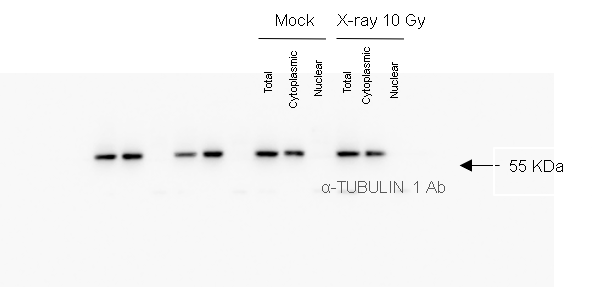

Supplement: Figure 7—figure supplement 1—source data 1. [file elife-79116-fig7-figsupp1-data1.zip › Figure 7-figure supplement 1-source data 1/+Label/Fig7-Fig supplement 1A-TUBULIN antibody(for HeLa mock +X-ray 10Gy ).tif]

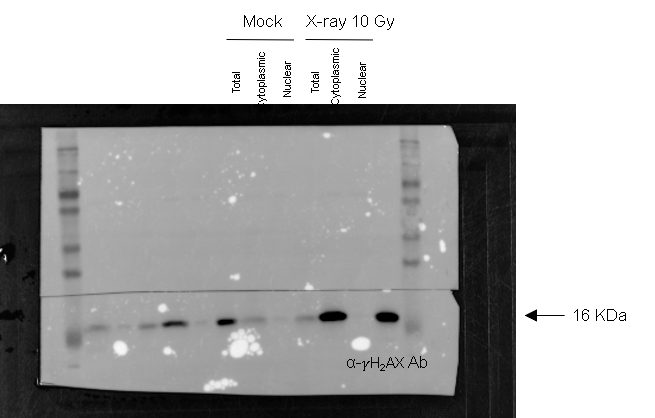

Supplement: Figure 7—figure supplement 1—source data 1. [file elife-79116-fig7-figsupp1-data1.zip › Figure 7-figure supplement 1-source data 1/+Label/Fig7-Fig supplement 1A-a├H2Ax antibody(for HeLa mock +X-ray 10Gy ).tif]

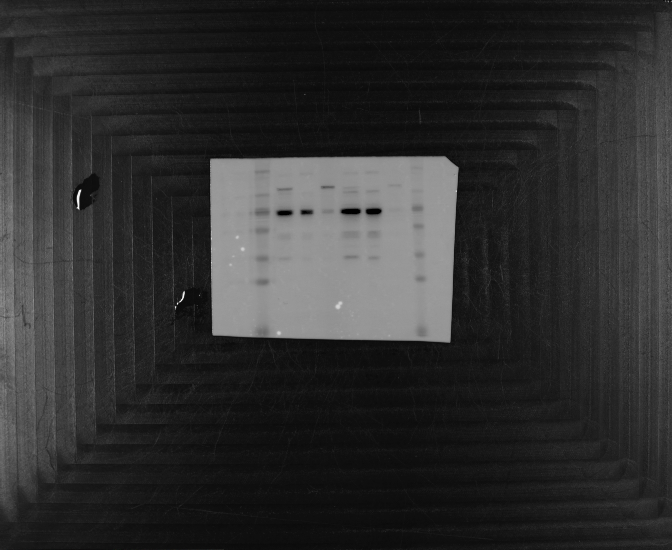

Supplement: Figure 7—figure supplement 1—source data 1. [file elife-79116-fig7-figsupp1-data1.zip › Figure 7-figure supplement 1-source data 1/Unedited/Fig7-Fig supplement 1A -GDOWN1 antibody(for HeLa mock +250mg per ml 12 h CHX ).Tif]

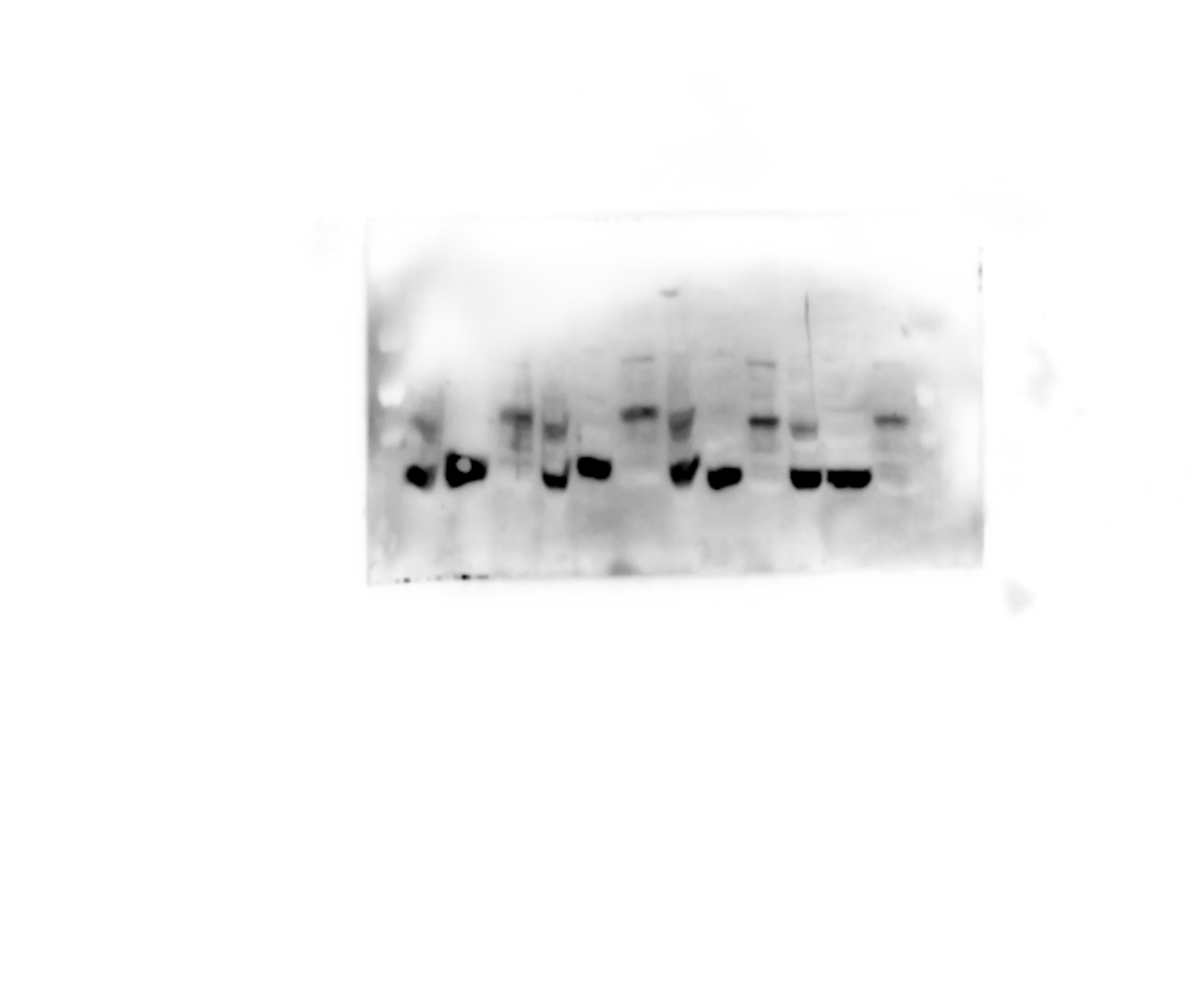

Supplement: Figure 7—figure supplement 1—source data 1. [file elife-79116-fig7-figsupp1-data1.zip › Figure 7-figure supplement 1-source data 1/Unedited/Fig7-Fig supplement 1A -GDOWN1 antibody(for HeLa mock +CPT+Doxorubicin ).Tif]

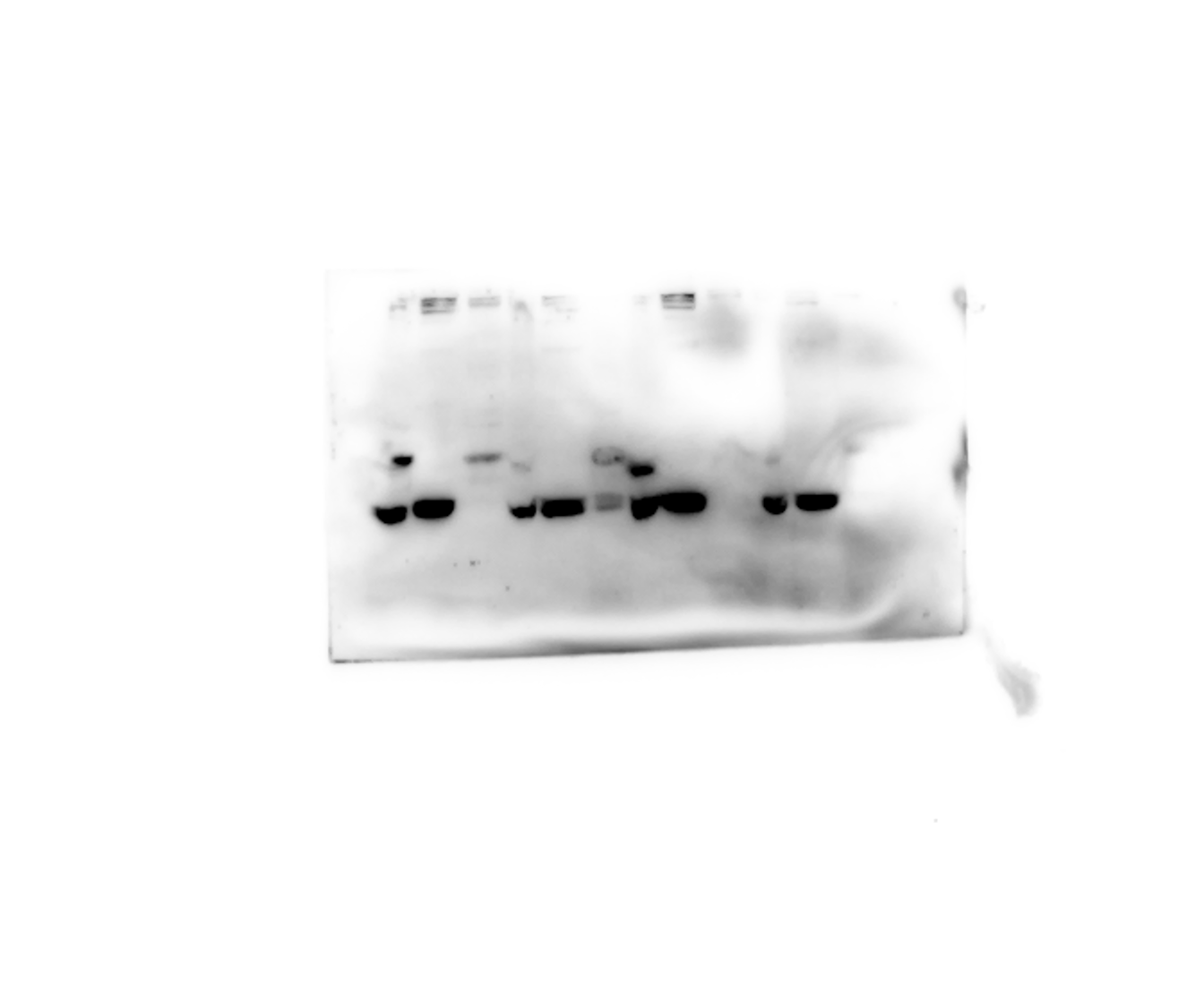

Supplement: Figure 7—figure supplement 1—source data 1. [file elife-79116-fig7-figsupp1-data1.zip › Figure 7-figure supplement 1-source data 1/Unedited/Fig7-Fig supplement 1A -GDOWN1 antibody(for HeLa mock +Madrasin+Tubercidin ).Tif]

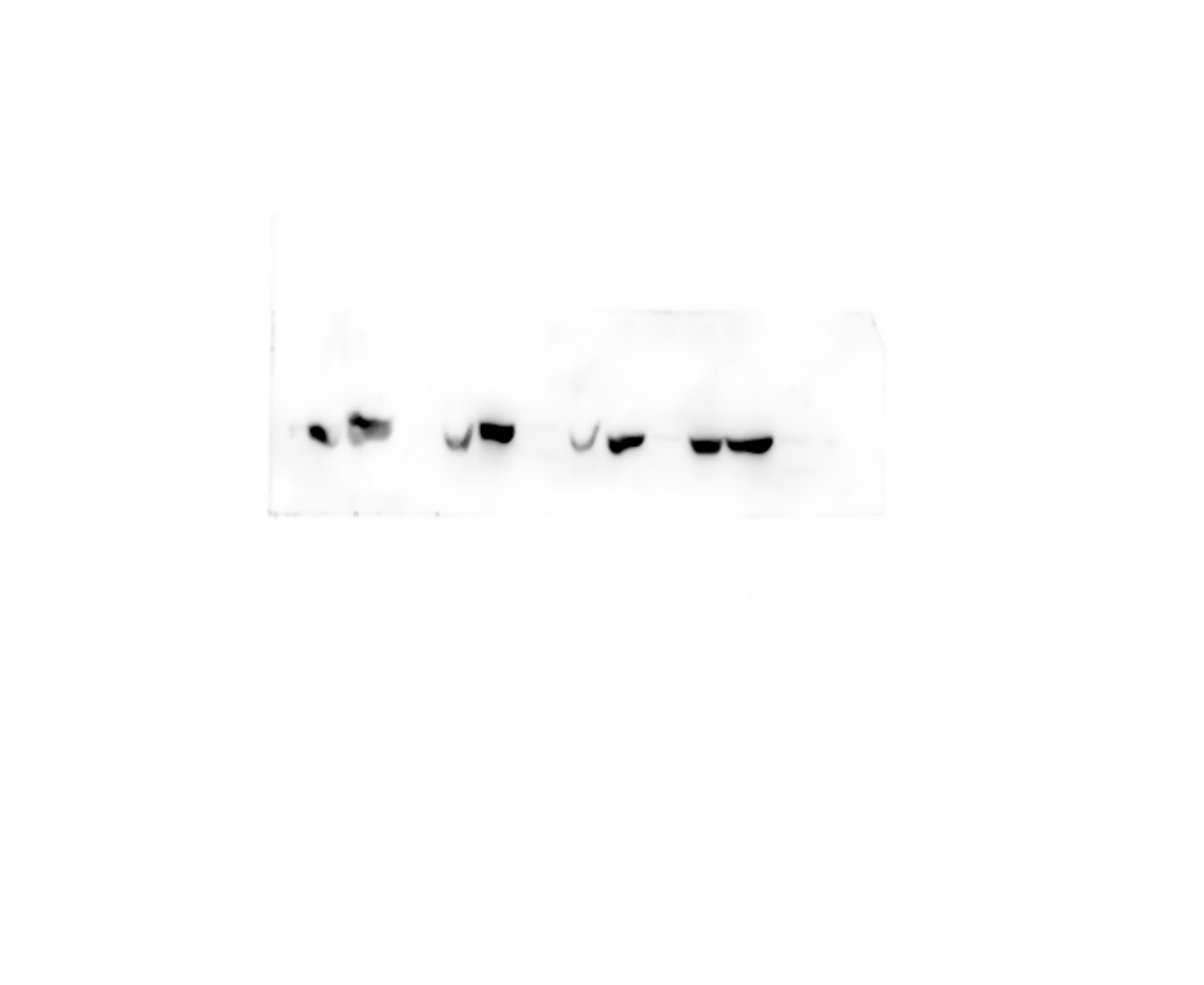

Supplement: Figure 7—figure supplement 1—source data 1. [file elife-79116-fig7-figsupp1-data1.zip › Figure 7-figure supplement 1-source data 1/Unedited/Fig7-Fig supplement 1A -TUBULIN antibody(for HeLa mock +CPT+Doxorubicin ).Tif]

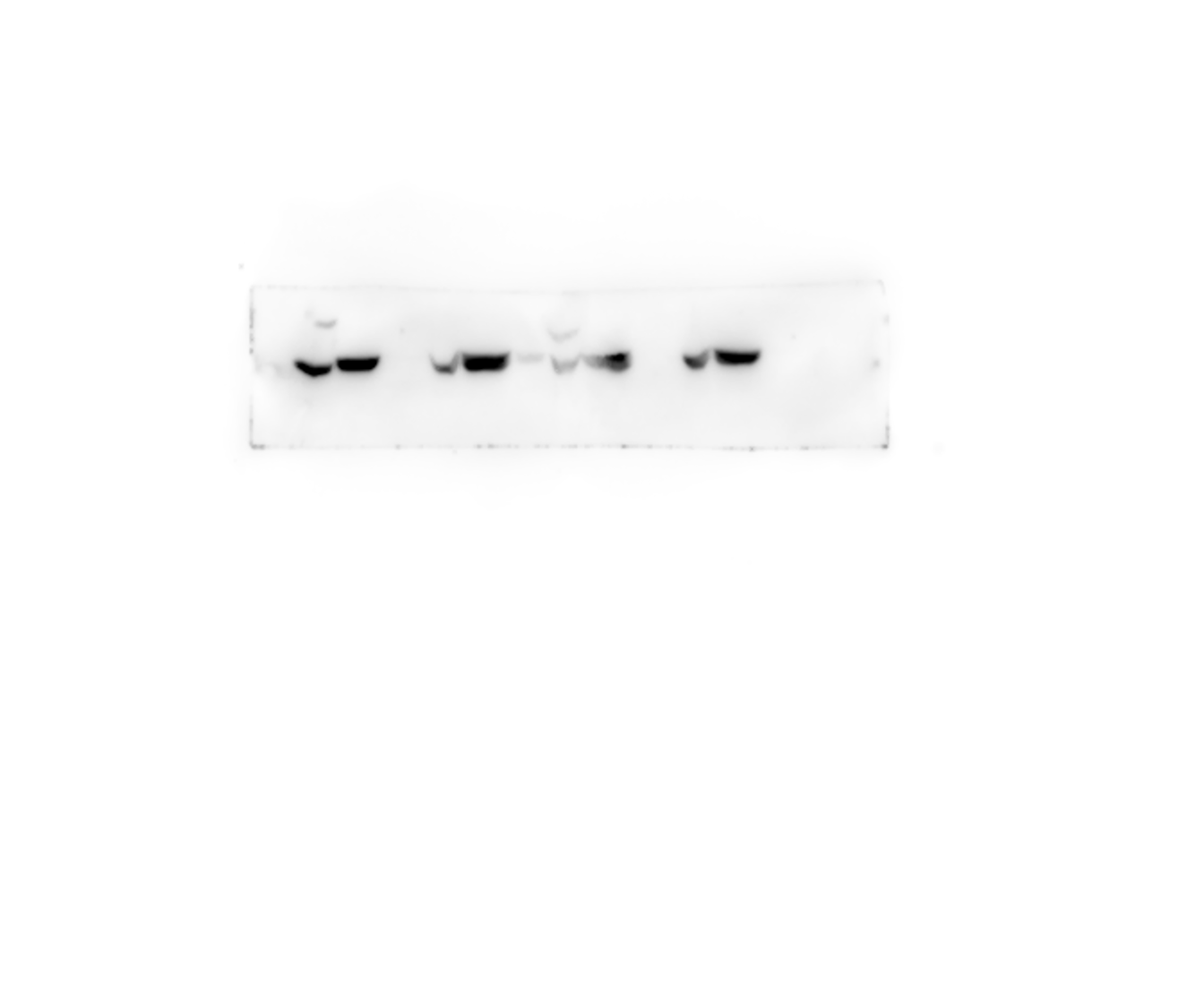

Supplement: Figure 7—figure supplement 1—source data 1. [file elife-79116-fig7-figsupp1-data1.zip › Figure 7-figure supplement 1-source data 1/Unedited/Fig7-Fig supplement 1A -TUBULIN antibody(for HeLa mock +Madrasin+Tubercidin ).Tif]

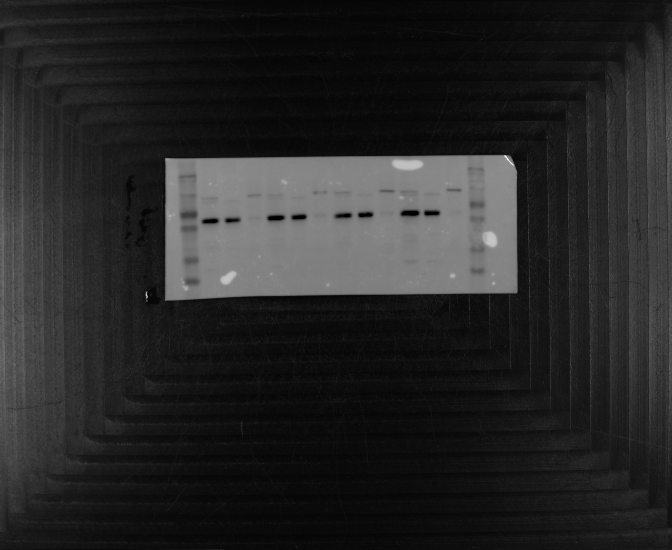

Supplement: Figure 7—figure supplement 1—source data 1. [file elife-79116-fig7-figsupp1-data1.zip › Figure 7-figure supplement 1-source data 1/Unedited/Fig7-Fig supplement 1A-GDOWN1 antibody(for HeLa mock +X-ray 10Gy ).Tif]

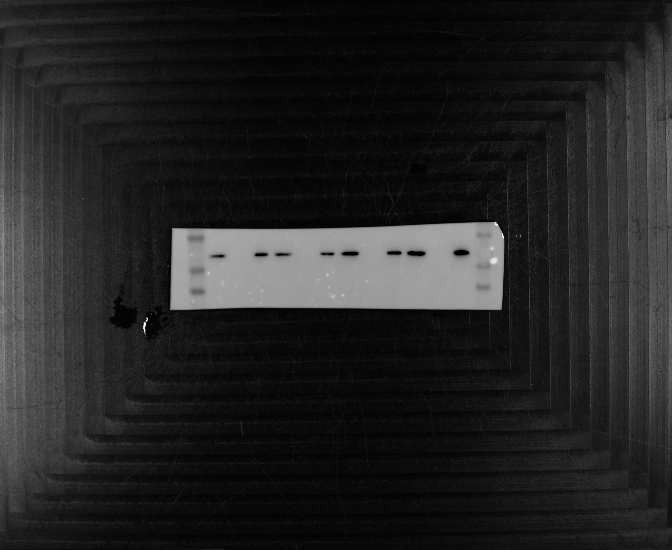

Supplement: Figure 7—figure supplement 1—source data 1. [file elife-79116-fig7-figsupp1-data1.zip › Figure 7-figure supplement 1-source data 1/Unedited/Fig7-Fig supplement 1A-TUBULIN antibody(for HeLa mock +X-ray 10Gy ).Tif]

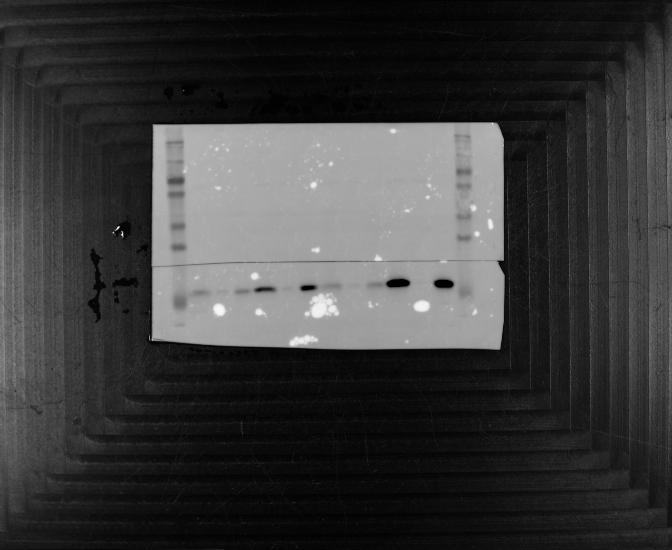

Supplement: Figure 7—figure supplement 1—source data 1. [file elife-79116-fig7-figsupp1-data1.zip › Figure 7-figure supplement 1-source data 1/Unedited/Fig7-Fig supplement 1A-a├H2Ax antibody(for HeLa mock +X-ray 10Gy ).Tif]
